# Supplementary material for: Estimating statistical significance of local protein profile-profile alignments
Source: BMC Bioinformatics. 2019 Aug 13;20:419. doi: 10.1186/s12859-019-2913-3 (PMC6693267; doi:10.1186/s12859-019-2913-3)
Supplement: Supplementary file 1 — Supplementary Materials. Methodological details, derivations, evaluation description, additional simulation and application results. (PDF 5,053 kb) [file 12859_2019_2913_MOESM1_ESM.pdf]

Estimating statistical significance of local protein profile-profile alignments

Supplementary Materials

Mindaugas Margelevičius

Institute of Biotechnology, Life Sciences Center, Vilnius University, Vilnius, Lithuania

mindaugas.margelevicius@bti.vu.lt

Contents

S1 Previous research **2**
S1.1 Statistics of gapped sequence alignments 2
S1.2 Distribution of profile-profile alignment scores 4

S2 Terminology **5**
S2.1 Effective number of observations 5
S2.2 Source profiles 5
S2.3 Reference profiles 5

S3 Preliminary findings **6**
S3.1 Alignment scores of real unrelated profiles 6
S3.2 Profile simulation issues 7

S4 Methodology **9**
S4.1 Profile simulation 9
S4.2 Comparison of simulated profiles 12
S4.3 Compositional similarity 12
S4.4 Prediction of statistical parameters 13
S4.5 Combining dependent p-values 14

S5 Evaluation **15**
S5.1 Model fitting and goodness-of-fit tests . . . 15
S5.2 Assessing statistical accuracy . . . 16
S5.3 Assessing profile-profile alignment performance . . . 16
S5.3.1 Datasets . . . 16
S5.3.2 Profile construction . . . 17
S5.3.3 Profile-profile alignment methods . . . 17
S5.3.4 Implementation of the algorithm proposed previously . . . 17
S5.3.5 Sensitivity evaluation . . . 17
S5.3.6 Alignment quality evaluation . . . 18

S6 Simulation results **18**
S6.1 Alignment scores of profiles generated using $S = 1012$ seed profiles . . . 18
S6.2 Alignment scores of profiles generated using $S = 1$ seed profile . . . 20
S6.3 Correlation between the estimates of the EVD parameters . . . 21
S6.4 Distribution of the number of positive substitution scores . . . 21
S6.5 Optimal adjustment parameters . . . 23

S7 Application results **23**
S7.1 Improvement in high-quality alignment rate . . . 23
S7.2 Statistical analysis with respect to false positives . . . 25
S7.3 Application to pairwise profile HMM alignments . . . 27

S8 Software and data availability **28**

A Appendix: Conditional mean estimators for the EVD parameters **31**
A.1 Conditional distribution for the parameters . . . 31
A.2 Prior distribution for the parameters . . . 31
A.2.1 Location parameter . . . 32
A.2.2 Scale parameter . . . 32
A.3 Conditional mean estimators . . . 33
A.3.1 Location parameter . . . 33
A.3.2 Scale parameter . . . 34

References **35**

S1 Previous research

There are many factors affecting the distribution of alignment scores. We overview them along with existing solutions to take them into account. We also discuss how these factors relate to the distribution of profile-profile alignment scores.

S1.1 Statistics of gapped sequence alignments

Local alignment score for random sequences grows logarithmically with the total size of the search space mn (see Eq (1) in the main text) (Waterman, 1994; Zhang, 1995), implying the same growth rate for the noise level (Spang and Vingron, 2001). Thus, even an error of a few

percents of the statistical parameters causes an increase of several times in the error in estimates of statistical significance. Accordingly, various solutions have been proposed to estimate the statistical parameters accurately (Karlin and Altschul, 1993; Karlin, 1994; Neuhauser, 1994; Mott and Tribe, 1999; Storey and Siegmund, 2001; Olsen *et al.*, 1999; Waterman and Vingron, 1994; Bundschuh, 2000, 2002a; Chia and Bundschuh, 2006; Yu and Hwa, 2001; Altschul *et al.*, 2001; Bundschuh, 2002b).

While many statistical techniques provide asymptotic estimates (in the limit of infinitely long sequences) for the statistical parameters, practical application requires defining the dependence of the statistical parameters on the lengths of sequences compared. Finite sequence lengths limit the extension of alignments starting near the end (edge) of a sequence, thus affecting the distribution of alignment scores (Altschul and Gish, 1996; Spang and Vingron, 1998; Altschul *et al.*, 2001). For short sequences, finite-length-induced edge effects can cause substantial changes in statistical parameter values.

In fact, edge effects always manifest in the far right tail of the score distribution (Hartmann, 2002; Wolfsheimer *et al.*, 2007; Newberg, 2008), as all real or simulated sequences are of finite length (here we consider high-scoring local alignments). However, the events of the alignment length approaching the length of sequences are of limited importance. Unless short sequences are compared, such events are extremely rare and the probability for them to occur is infinitesimal. Thus, in general, the distribution defined precisely on an interval of scores for which the expected alignment length is much less than the length of sequences suffices for practical purposes. Note that edge effects still appear for the reasons described above.

Another important factor that affects values of the statistical parameters is the amino acid composition of sequences. A compositional similarity between unrelated sequences causes an overestimate of statistical significance (Mott, 1992, 2000) because of an underlying process of sequence composition not accounted for by a null model of random sequences/alignments. Alignment scores of sequences with a similarly biased composition produce an elongated right tail in the distribution (1) (main text), implying a smaller value of the inverse scale parameter λ.

A corresponding change in the distribution follows when gaps are disallowed. Since λ can be calculated numerically for ungapped alignments, the λ computed with the modification of using the observed residue frequencies of two sequences as a null model corresponds to a measure of compositional bias. Using the computed λ in estimating the statistical significance of gapped alignments has yielded more accurate alignment statistics (Mott, 1992; Yu *et al.*, 2006). In this study, we develop an approach that takes into consideration compositional similarity based on calculated λ.

Other procedures for taking compositional bias into consideration exist. However, for example, scaling an amino acid substitution (score) matrix to obtain a reference value for λ calculated in the context of standard residue frequencies (known as composition-based statistics) (Altschul *et al.*, 1997; Schäffer *et al.*, 2001) alters the substitution scores and can lead to decreased sensitivity to related proteins (Yu *et al.*, 2006). A different modification of substitution scores such that target frequencies (characterizing the distribution of aligned amino acid pairs) are consistent with the observed residue frequencies of two sequences and close to the target frequencies inherent in a reference substitution matrix (Yu *et al.*, 2003) is beneficial only under certain conditions (Altschul *et al.*, 2005).

On the other hand, strategies suggesting a separate estimation of statistical parameters for each pair of sequences and thus implicitly taking account of compositional bias without reference to distributions (Metzler, 2006; Poleksic *et al.*, 2005) may be inaccurate. Processing of suboptimal alignments of two sequences introduces statistical bias into estimates of statistical parameters (Altschul *et al.*, 2001). Correcting for the statistical bias unknown and potentially different for

each pair of compositionally biased sequences may even be prohibitive for short sequences. A different strategy—estimating statistical parameters each time during a database search (Pearson, 1998; Bailey and Gribskov, 2002)—confronts such challenges as differentiation between related and unrelated sequences and scarcity of data (when avoiding averaging over the values of sequence attributes, e.g., length and composition).

S1.2 Distribution of profile-profile alignment scores

The statistical theory for ungapped sequence alignments pertains to profile-to-sequence alignments, as the profile represents a position-specific score matrix constructed for a particular multiple sequence alignment (MSA) using the same principles for building scores as the construction of standard substitution matrices adheres to (Altschul *et al.*, 1997). For example, an alignment between a profile describing a single sequence and another sequence corresponds to that of the two sequences using a standard substitution matrix.

The convergence of ungapped alignment score to the EVD for a special case of profile construction has been proved theoretically (Goldstein and Waterman, 1994). Further, the scores of profile-to-sequence alignments with affine gap costs have been empirically demonstrated to follow an EVD (Altschul *et al.*, 1997). It has also been shown that the EVD closely approximates the distribution of scores produced by aligning to sequences both the profile running the hybrid algorithm (Yu *et al.*, 2002) and the profile HMM (Viterbi algorithm) (Eddy, 2008). Hence, the well-established statistical theory and the experimental results conveys all the considerations related to sequence alignment (e.g., corrections for edge effects and compositional bias) to profile-to-sequence alignment.

A different picture, as it may seem, emerges in pairwise profile alignment. The probabilities of amino acids characterize each profile position, and not every profile-profile scoring function leads to an unambiguous characterization of profile-profile alignment score. However, typically, the similarity score of two profile positions takes into account the probability for a pair of positions to appear in an alignment by chance and is implicitly log-odds. The similarity scores, therefore, can be scaled so that the corresponding (implicit) target frequencies (Karlin and Altschul, 1990; Altschul, 1993) become a valid probability distribution over all pairs of positions. Evidence suggests that scores of a type of log-odds are statistically most powerful for recognizing alignments whose aligned pairs are defined by the target distribution the scores entail (Meng *et al.*, 2011). Log-odds scores immediately satisfy the conditions of negative expected score with at least one positive (Karlin, 2005; Meng *et al.*, 2011), and their maximal sum can be characterized by the statistics of local alignment (see Eq (1)). Hence, given an implicit target distribution different from a random distribution and gap penalties that ensure negative expected score per aligned pair, profile-profile alignment score should obey the EVD.

Indeed, this has been confirmed empirically (Sadreyev and Grishin, 2003; Poleksic, 2009; Margelevičius and Venclovas, 2010). Yet, no clear answer to what and whether one type of distribution governs profile-profile alignment score exists. A distribution called 'power EVD', or PEVD, has been proposed as a model for the distribution of scores obtained by aligning profiles generated by a process leading to a more realistic representation of unrelated proteins (Sadreyev and Grishin, 2008). Still, all this progress has been surrounded by some limitations. And none of the studies included the scoring of secondary structures (SSs) predicted for profiles and analyzed its impact on the distribution of alignment scores.

S2 Terminology

S2.1 Effective number of observations

The effective number of observations (ENO) that a profile represents is defined as the median of the exponential functions of entropy values, $\exp(-\sum_a f_{ia} \log f_{ia})$, calculated at each profile position (Pei and Grishin, 2001), where f_{ia} represents the observed frequency of amino acid a at profile position i. The ENO ranges from 1 to 20. However, ENO values greater than or equal to 16 indicate high sequence diversity of the multiple sequence alignment (MSA) used to construct the profile and are highly uncommon for real profiles.

S2.2 Source profiles

Profiles constructed for real sequences and used to generate random profiles are referred to as source profiles. The algorithms of generating profiles that are based on assembling fragments of source profiles require the source profiles to be unrelated. A diverse set of unrelated profiles representing source profiles was prepared as follows.

First, UniRef50 database (Suzek *et al.*, 2015) sequences (2013) were clustered at 20% sequence identity using the BLASTclust utility from the NCBI BLAST software suite (Altschul *et al.*, 1997) with a length coverage threshold of 0.7 applied to either of two sequences being compared and soft masking of low complexity regions. Sequences that did not form clusters and whose low complexity regions (Wootton and Federhen, 1996) constituted less than 20% of the sequence length were retained. Then, input MSAs for profile construction were obtained by running for each resulting sequence HMMER3 (Eddy, 2011) for two iterations using a sequence inclusion threshold of 0.001 against the UniRef50 database (2015). To obtain a sufficient number of profiles of a larger ENO, for some sequences, the number of iterations and the E-value threshold for sequence inclusion gradually increased up to 6 and 0.5, respectively. The final set of source profiles contained medium-length profiles of an ENO ranging from 2 to 14 in multiples of 2. The number of the source profiles varied from 1307 (ENO $n = 14$) to 2647 ($n = 6$) and the length from 333 ± 68 ($n = 12$) to 392 ± 6 ($n = 2$).

S2.3 Reference profiles

Reference profiles are profiles constructed for real unrelated sequences. The distribution of scores obtained by aligning reference profiles provides a reference for the distribution of alignment scores of simulated profiles and hence an indication of how closely simulated profiles represent real unrelated profiles (profiles constructed from real MSAs).

In contrast to source profiles used to generate random profiles, reference profiles are compared directly to create a distribution of alignment scores. Therefore, even weak homology is not supposed to be shared among reference profiles. Below is the description of the procedure for computing and selecting reference profiles.

The diverse set of sequences filtered to 20% sequence identity and used in the procedure for computing the source profiles were further clustered using BLASTclust with a length coverage threshold of 0.125 applied to either of two sequences being compared. MSAs and corresponding profile HMMs for the resulting sequences that did not form clusters and did not contain low complexity regions were obtained by running HMMER3 for two iterations using a sequence inclusion threshold of 0.001 against the UniRef50 database (2015). Searches of some sequences

with an E-value threshold for sequence inclusion of 0.01 were performed to increase the number of COMER profiles of a larger ENO.

We retained each COMER profile constructed for an MSA for which HMMER3 search with the corresponding HMM against the resulting set of clustered sequences did not retrieve, with an E-value less than 10, any other sequences than that for which the MSA was built. The final reference profiles were grouped into bins of a certain ENO and length. The ENO values were 2, 4, 6, and 8. The length values were 100, 200, 400, and 600. Profile lengths were allowed to vary within 5% in each bin. Bins containing less than 50 profiles were not considered.

To further exclude possibly related profile pairs, we removed from consideration the profile pair aligned with the highest score in the alignment list obtained by searching a reference profile from a bin against the profiles in another bin.

S3 Preliminary findings

S3.1 Alignment scores of real unrelated profiles

The distributions of alignment scores of real unrelated profiles (constructed from MSAs of real sequences) were obtained by aligning with COMER Margelevičius (2016) the reference profiles (Section S2.3) grouped into bins of profile ENO and length. Aligning the reference profiles from one bin against the reference profiles in the other bins gave rise to the distributions for different pair values of profile ENO and length. Distributions for different values of profile length and compositional similarity were similarly obtained.

The resulting distributions along with EVD fits are shown in Figure S1. Figure S2 provides some examples (A and B) where an EVD fits the data well in the upper tail. It also shows an example (C) where a goodness-of-fit test (see Section S5.1) rejects the null hypothesis, but the shape of the distribution matches the EVD despite some outliers, which may correspond to related profile pairs.

Figure S1. **Distributions of alignment scores of real unrelated profiles for different pair values of profile ENO and length.** The red and the green curves represent a maximum likelihood fit of the EVD and the generalized EVD to the data, respectively. P-values of the tests of goodness of fit of the EVD to the data are shown. N, the number of alignment scores. (*Please find the figure among Additional files.*)

Overall, the tests of goodness of fit of the EVD in the right tail of an alignment score distribution (Figure S1, Table S1) show the appropriateness of the EVD to describe the distribution of profile-profile alignment scores. Yet, the distribution of the p-values of the goodness-of-fit tests (Figure S2D) is not uniform as would be expected in the case of sampling many times a large number of alignment scores from an EVD. However, in the context of aligning real profiles and performing only 53 goodness-of-fit tests (for each plot in Figure S1) for a limited number of produced alignment scores, the distribution of p-values in Figure S2D is dispersed so that 85% of the tests do not reject the null hypothesis. We therefore assume that the distribution of alignment scores of real unrelated profiles can be approximated by the EVD.

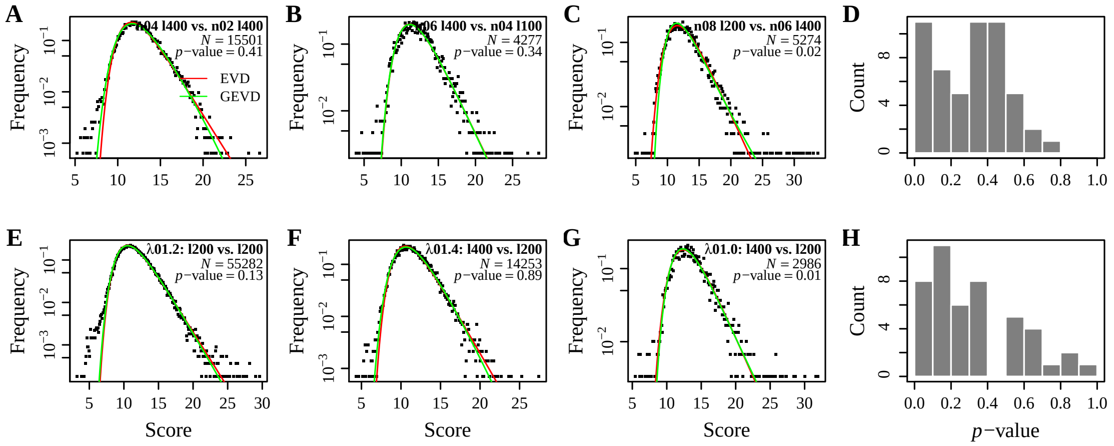

Figure S2. **Distribution of alignment scores of real unrelated profiles.** Panels (A–C) plot on a log scale three distributions obtained from aligning reference profiles of ENO n and length l against reference profiles with different values of n and l. Panel (D) displays the distribution of the p-values of upper-tail Anderson-Darling goodness-of-fit tests. (C) represents an example where the goodness-of-fit test rejects the null hypothesis in the presence of outliers, but the shape of the alignment score distribution matches the EVD. (E–G): Alignment score distributions for real profiles of specified mutual compositional similarity λ and length l. (G) shows the distribution with outliers, similar as shown in (C), for which goodness-of-fit p-value is the lowest. Figure S3 and Table S2 show all distributions and the results of the goodness-of-fit tests for different values of λ and l. (H): The distribution of the p-values of goodness-of-fit tests for alignment scores obtained from aligning profiles with different compositional similarity and length. The red and the green curves represent a maximum likelihood fit of the EVD and the generalized EVD (Kotz and Nadarajah, 2000) (GEVD, for illustration only) to the data, respectively. The GEVD fits illustrate the curvature of the empirical distributions. N, the number of alignment scores.

Table S1. **Goodness of fit of the EVD to the distribution of alignment scores of real unrelated profiles.** Distribution represents distributions obtained from aligning reference profiles of ENO n and length l against reference profiles with different values of n and l. The table reports the estimates and their standard errors (SE) for the location and scale parameters of the EVD for each distribution of alignment scores. N is the number of alignment scores. AD_{up} is the supremum class upper-tail Anderson-Darling statistic. The p-value of statistic AD_{up} was computed by Monte Carlo simulation with 100 samples. (*Please find the table among Additional files.*)

Figure S3. **Distributions of alignment scores obtained from aligning pairs of real profiles with mutual compositional similarity λ and different values of length l.** The red and the green curves represent a maximum likelihood fit of the EVD and the generalized EVD to the data, respectively. N, the number of alignment scores. (*Please find the figure among Additional files.*)

S3.2 Profile simulation issues

It has been shown (Sadreyev and Grishin, 2008) that generating random profiles by a procedure leading to their realistic representation, obtaining and using statistics from the distribution of their alignment scores improve remote homology detection. However, characterizing the distribution of profile-profile alignment scores, where calculated similarities between predicted SSs and

Table S2. **Goodness of fit of the EVD to the distribution of alignment scores of real unrelated profiles.** Distribution represents distributions obtained from aligning pairs of real profiles with mutual compositional similarity λ and different values of length l. The table reports the estimates and their standard errors (SE) for the location and scale parameters of the EVD for each distribution of alignment scores. N is the number of alignment scores. The p-value of statistic AD_{up} was computed by Monte Carlo simulation with 100 samples. (*Please find the table among Additional files.*)

sequence contexts (Margelevičius, 2016, 2018) contribute to an alignment score, can be ambiguous.

For example, consider the procedure of randomly generating unrelated profiles, by which a generated profile corresponds to a series of variable-length profile fragments sampled randomly, concatenated and cut to match the order and lengths of SS segments predicted for real profiles (Sadreyev and Grishin, 2008). Applied to profiles constructed for unrelated sequences from the UniRef50 database (Suzek *et al.*, 2015) (Section S2), it leads to alignment scores whose distribution's right tail becomes heavier and increasingly deviates from an EVD with increasing profile length and ENO (Figure S4A and D). The right tail of the distribution of alignment scores becomes even heavier when generated profiles consist of randomly sampled fragments preserving only the order of SS segments predicted for real profiles (Figure S4B and E).

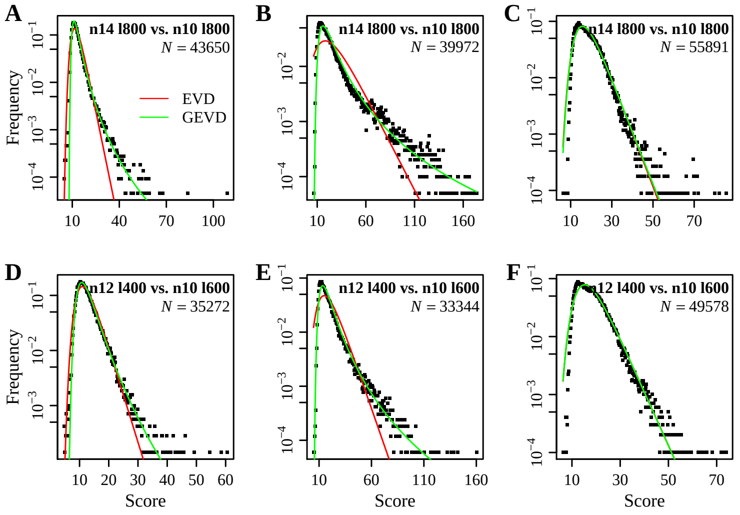

Figure S4. **Distribution of alignment scores of profiles generated following SS predictions for real profiles.** Results under two settings for profile comparison are shown. (A–C) show distributions obtained by comparing profiles of an effective number of observations $n = 14$ and a length $l = 800$ against those with $n = 10$ and $l = 800$. Profiles with $n = 12$ and $l = 400$ were compared against those with $n = 10$ and $l = 600$ to obtain distributions shown in (D–F). (A,D): Profiles were compiled of real profile fragments sampled randomly, concatenated and cut to match the SS types and the lengths of SS segments predicted for real profiles. (B,E): Profiles were compiled as in (A,D) but with no constraints imposed on fragment lengths. (C,F): Profiles were compiled as in (B,E) except that long profile fragments corresponding to SS type C had been randomly cropped not to exceed the median length of predicted coils. The red and the green curves represent a maximum likelihood fit of the EVD and the generalized EVD (to illustrate the curvature of the empirical distributions) to the data, respectively. N, the number of alignment scores.

These findings suggest two conclusions. First, the alignment of profiles generated following SS predictions of real profiles leads to a distribution of scores that diverges from an EVD as the length and ENO of the generated profiles increase.

Here we note differences from the original procedure (Sadreyev and Grishin, 2008). It used Pfam (Finn *et al.*, 2016) MSAs for the construction of profiles representing a population of fragments, and it produced alignment scores that did not include similarities between predicted SSs and other derived measures. Although a new type of alignment score distribution, PEVD, was introduced, alignment scores produced here distribute differently because of, most importantly, the inclusion of similarity scores between predicted SSs.

Second, the distribution of alignment scores strongly depends on an algorithm for compiling profile fragments into a profile. This point is additionally demonstrated by the following illustration.

A large proportion of the profile length (about half on average) is usually predicted to be a coil (C), and similarity between long coils, for example, can lead to a high-scoring alignment. Figure S4C and F show that in comparison with the results shown in Figure S4B and E, the number of high-scoring alignments decreased considerably when long (>10) profile fragments corresponding to SS type C were randomly cropped not to exceed the median length (6) of coils.

Evidently, profile fragments of the same SS type increase the probability of producing a high-scoring alignment between them (Figure S4A and D). As profile length and ENO increase, alignment scores can result in a distribution that resembles a mixture of distributions and does not represent a distribution obtained by aligning unrelated profiles.

To reduce correlations between randomly sampled variable-length profile fragments, we propose an algorithm by which unrelated profiles are generated by randomly sampling and concatenating fixed-length fragments regardless of SS predictions the fragments entail. A random sampling of fixed-length fragments independently of profile qualities reduces the odds for two generated profiles to share similar long segments, provided the length of fragments is not large.

S4 Methodology

In this section, we provide details of the methodology developed for estimating the statistical significance of profile-profile alignments. We detail the algorithm developed for generating random profiles such that the distribution of their alignment scores resembles that obtained for real profiles. Next, we describe the characterization of the distribution of alignment scores of random profiles, dependent upon profile attributes (length and ENO) and compositional similarity between profiles. We also describe the calculation of the combined statistical significance of alignment score and the normalized number of positive substitution scores.

S4.1 Profile simulation

It is well established that the parameters of the distribution of sequence alignment scores depend on the lengths of the sequences being compared. The distribution of profile-profile alignment scores depends not only on profile lengths but also on ENOs. ENO summarizes in one number the observed frequencies of the amino acids per profile position and effectively represents the number of observations. Therefore, given that a profile-profile substitution score at each pair of profile positions depends on the observed frequencies and consequently on the number of observations,

the distribution of alignment scores depends on profile ENOs, which has been demonstrated empirically (Sadreyev and Grishin, 2008).

To investigate the impact of the profile length and ENO on the distribution of alignment scores, the presence of unrelated profiles of different lengths and ENOs is a necessary prerequisite. The steps for generating random profiles of all lengths $l \in \mathsf{L}$ and ENOs $n \in \mathsf{N}$ are described in Algorithm S1, where $\mathsf{L} = \{50, 100, 200, 400, 600, 800\}$ and $\mathsf{N} = \{2, 4, 6, \ldots, 14\}$ denote the sets of profile length and ENO values, respectively, used in this study. (Algorithm S1 differs from Algorithm 1 of the main text by specifying the generation of profiles of all $l \in \mathsf{L}$ and $n \in \mathsf{N}$ and is given for consistency.)

Algorithm S1 Generating random profiles given noise level r and fragment length s

---

Input: S sequences.
Output: R random profiles for each $n \in \mathsf{N}$ and $l \in \mathsf{L}$.

1. Make profiles (seeds) for S diverse sequences chosen at random for which profiles are obtained to be of a sufficiently large ENO (e.g., 12).
2. Using each of the S profiles as a model and Algorithm S2, generate M MSAs with noise level r for each ENO $n \in \mathsf{N}$, i.e., characterized by an ENO n.
3. Using each set of $S \times M$ generated MSAs with ENO $n \in \mathsf{N}$ as source MSAs, generate a set of R random MSAs for each length $l \in \mathsf{L}$ in the following way:
   (a) choose a number j at random uniformly between 1 and $S \times M$;
   (b) randomly select a fragment of length s of source MSA j;
   (c) copy and add the selected fragment to a random MSA being generated;
   (d) repeat steps (a)–(c) until the length of the random MSA becomes l.
4. Construct profiles from the random MSAs generated in quantities of R for each $n \in \mathsf{N}$ and $l \in \mathsf{L}$.

Algorithm S1 (Algorithm 1 of the main text) specifies that R random profiles for each ENO n and length l are generated using $S \times M$ MSAs produced with added noise by the profile models from the diverse set of S real unrelated profiles (see Section S2.2 for a description of the procedure for obtaining unrelated profiles).

The diverse set of S unrelated profiles represents a random sample drawn from a large population of profiles and thus ensures that R random profiles generated for each ENO n and length l combine features (fragments) characteristic to real profiles (sequence families). Each set of M MSAs produced using one of the S profile models augments the dataset used to generate R random profiles and can be viewed as a parametric bootstrap sample. In fact, S collections, each of which contains M MSAs, represent different subsamples in the context of the procedure of the bag of multiple bootstraps (Kleiner *et al.*, 2014; Liang *et al.*, 2016). The exception is that the parameters of the alignment score distribution are estimated based on all of the subsamples instead of averaging estimates computed for each subsample. Both estimators are consistent (Liang *et al.*, 2016; Wang *et al.*, 2017).

A large number S of seed profiles ensure required diversity, and M can be small. M, however, should increase for decreasing values of S. We show in the main text (Section 2.4) that the combinations of large S and small M and vice versa lead to similar distributions of scores produced

by aligning randomly generated profiles. Even a single seed profile, $S = 1$, provides a diverse set of M MSAs through adding noise to the produced MSAs.

The noise level r determines the extent to which M MSAs vary around the profile used to produce the MSAs. Since the same set of seed profiles are used to produce M MSAs for each ENO $n \in \mathsf{N}$, generating MSAs by Algorithm S2 requires a smaller number of iterations when using seed profiles of a sufficiently large ENO (step 1 of Algorithm S1). The scale matrices $\boldsymbol{\Sigma}_\tau$ and $\boldsymbol{\Sigma}_\mathrm{e}$ in Algorithm S2 control variability of the elements of random vectors. We set $\boldsymbol{\Sigma}_\tau$ and $\boldsymbol{\Sigma}_\mathrm{e}$ to the matrices with diagonal entries 1 and off-diagonal entries 0.5.

Along with the fragment length s, the noise level r also determines the degree of similarity that randomly sampled fragments share across R generated profiles. In the main text, we discuss how to optimize r and s so that alignment scores of R generated profiles follow the same type of distribution as do alignment scores of real unrelated profiles and that the divergence between these distributions is minimized.

Algorithm S2 Generating an MSA of ENO n with noise level r using a profile model

---

Input: A profile model.
Output: A simulated MSA.

1. Add random noise to the profile by applying the following steps for each profile position i:
   (a) generate a three-dimensional multivariate logistic normal random variable $\boldsymbol{\tau}_x$ with location vector $\mathbf{0}$ and scale matrix $\boldsymbol{\Sigma}_\tau$, representing transition probabilities from state $x = \mathrm{M, I, D}$ (match, insert, and delete states, respectively);
   (b) combine $\boldsymbol{\tau}_x$ with the profile's transition probabilities, $\boldsymbol{\tau}_{ix}$, to obtain new transition probabilities at position i for state $x = \mathrm{M, I, D}$: $r\boldsymbol{\tau}_x + (1-r)\boldsymbol{\tau}_{ix}$ ($r \in [0,1]$);
   (c) generate an A-dimensional ($A = 19$) multivariate logistic normal random variable $\boldsymbol{e}$ with location vector $\mathbf{0}$ and scale matrix $\boldsymbol{\Sigma}_\mathrm{e}$, representing amino acid target frequencies;
   (d) combine $\boldsymbol{e}$ with the profile's target frequencies, $\boldsymbol{e}_i$, to obtain new target frequencies at position i: $r\boldsymbol{e} + (1-r)\boldsymbol{e}_i$.
2. Generate an MSA of k sequences such that its ENO $n' = n$:
   (a) sample a sequence of states from the distributions of transition probabilities and a corresponding amino acid sequence from the distributions of target frequencies of the profile model with added noise;
   (b) repeat step (2a) k times;
   (c) incorporate the sampled amino acid sequences into the MSA based on the sequence of states available for each amino acid sequence;
   (d) calculate the ENO n' of the resultant MSA;
   (e) increase (if $n' < n$) or decrease k (if $n' > n$) according to the binary search algorithm;
   (f) repeat steps (2a)–(2e) until $n' = n$ or stop if a maximum number of iterations is reached.

S4.2 Comparison of simulated profiles

The distributions of alignment scores of profiles simulated with noise level r and fragment length s were obtained by aligning 250 simulated profiles of ENO $n_1 \in \mathsf{N}$ and length $l_1 \in \mathsf{L}$ against 250 simulated profiles of ENO $n_2 \in \mathsf{N}$ ($n_2 \neq n_1$) and length $l_2 \in \mathsf{L}$. In this way, the distributions of alignment scores between two sets of profiles, characterized by different values of profile attributes (n_1, l_1) and (n_2, l_2), were obtained. Note that profiles of the same ENO were not compared with one another because the same set of $S \times M$ source MSAs used to generate all profiles of the same ENO (Algorithm S1) implies dependence between profiles of the same ENO and cause greater similarity among them.

The distributions of the scores of alignments between profiles of length $l_1 \in \mathsf{L}$ and profiles of length $l_2 \in \mathsf{L}$ with mutual compositional similarity λ_u (Section S4.3) were obtained from the alignments produced as described above.

S4.3 Compositional similarity

The statistical theory for sequence and profile-to-sequence alignments provides the limiting distribution of alignment scores (Eq 1, main text), where the statistical parameter $\lambda_\mathrm{u} \equiv \lambda$ has several related meanings. In addition to determining the scale of the distribution of ungapped alignment score, it also represents a scale factor that allows calculating the target frequencies implicit in the substitution matrix (Karlin and Altschul, 1990; Altschul *et al.*, 1997), it provides a reference value for rescaling the substitution matrix so that reference statistical parameters can be applied in the presence of compositionally biased sequences (Altschul *et al.*, 1997; Schäffer *et al.*, 2001), and it itself is a measure of compositional similarity (Yu *et al.*, 2006). It is easy to see that the value of λ_u found as the positive solution to

$$\sum_k p(s_k) \exp(\lambda_\mathrm{u} s_k) = 1, \tag{S4.1}$$

where $\{s_k\}_k$ represent different values of scores in the substitution matrix and $p(s_k)$ is the probability of s_k, will decrease as the number of positive substitution scores increases ($p(s_k)$ increases for all $k : s_k > 0$). Low values of λ_u may, therefore, indicate the possibility for a high-scoring alignment to occur by chance due to compositionally biased regions in two sequences.

The parameter λ_u calculated for a pair of profiles (Sadreyev and Grishin, 2003; Margelevičius and Venclovas, 2010) has the same dependence on composition. We take into account compositional similarity between profiles by specifying the dependence of the distribution of profile-profile alignment scores on λ_u and the lengths of profiles being compared. In other words, in addition to having obtained an alignment score distribution for profiles characterized by two sets of attributes (n_1, l_1) and (n_2, l_2), we also obtain the distribution of the alignment scores of the pairs of simulated profiles of lengths l_1 and l_2 whose λ_u calculated and rounded to the nearest multiple of 0.1 is equal to a specified value.

We combine the statistical parameters of these two distributions as follows. Let A and B index two distributions, where the first is obtained for profiles described by the set of attributes $\{n_1, l_1; n_2, l_2\}$ and the other for profiles characterized by the set of attributes $\{\lambda_\mathrm{u}; l_1; l_2\}$. Assume that distributions A and B belong to the family of EVDs. Let $\hat{\mu}^\mathrm{A}$ and $\hat{\sigma}^\mathrm{A}$ respectively denote the estimates of the location and scale parameters of the EVD corresponding to distribution A. Similarly, let $\hat{\mu}^\mathrm{B}$ and $\hat{\sigma}^\mathrm{B}$ be the estimates of the statistical parameters of distribution B. Then, the conditional mean estimator of the location parameter given two sets of parameters specifying

its distribution in settings A and B is (see Appendix A for a proof)

$$\hat{\mu} = a\hat{\mu}^{\mathrm{A}} + (1-a)\hat{\mu}^{\mathrm{B}} \quad (0 < a < 1), \tag{S4.2}$$

and the corresponding conditional mean estimator of the scale parameter is

$$\hat{\sigma} = b\hat{\sigma}^{\mathrm{A}} + (1-b)\hat{\sigma}^{\mathrm{B}} \quad (0 < b < 1), \tag{S4.3}$$

where a and b are parameters that depend on the statistical parameters specifying the distributions of the location and scale parameters, respectively, in settings A and B (see Appendix A for details).

An alternative measure of compositional similarity is an exponential function of the divergence between two profiles measured by a symmetrized Kullback-Leibler divergence, $c_1 \exp(-c_2[D_{\mathrm{KL}}(Q\|P) + D_{\mathrm{KL}}(P\|Q)])$, where c_1 and c_2 are constants and P and Q represent the average vectors of target frequencies calculated for the two profiles. However, it represents a rather generalized measure, which has been found to be not as effective as λ_{u}. The approach of combining the statistical parameters described above has also been found to be more stable than modeling λ_{u} with a gamma distribution given the set of profile attributes $\{n_1, l_1; n_2, l_2\}$.

S4.4 Prediction of statistical parameters

The distributions of alignment scores of simulated profiles are obtained for given values of profile ENOs $n_1, n_2 \in \mathsf{N}$ and lengths $l_1, l_2 \in \mathsf{L}$ and discretized values of λ_{u}. The statistical parameters of distributions for any values of n_1 and n_2, l_1 and l_2, and λ_{u} are predicted using an artificial neural network (NN) model trained on the estimates of statistical parameters obtained from the observed distributions. Thus, under the assumption that distributions A and B (see Section S4.3) are EVDs, trained NNs predict μ^{A} and σ^{A} based on the profile attributes $\{n_1, l_1; n_2, l_2\}$ and predict μ^{B} and σ^{B} based on profile compositional similarity, $\{\lambda_{\mathrm{u}}; l_1; l_2\}$.

Note that the parameters a and b in (S4.2) and (S4.3) can be calculated directly if an NN architecture allows for prediction of the mean and variance parameters for μ^{A} and μ^{B} and the shape and scale parameters for σ^{A} and σ^{B} (see Appendix A). However, a and b values calculated in this way will vary across different input values of $\{n_1, l_1; n_2, l_2\}$ and $\{\lambda_{\mathrm{u}}; l_1; l_2\}$ and depend on the amount of data available for different profile attributes.

We, therefore, assume that predictions of the trained NNs correspond to estimates of the mean values of the statistical parameters (see Appendix A) and optimize a by minimizing the mean absolute error (MAE)

$$\frac{1}{2C} \sum_{\substack{n_1,n_2 \in \mathsf{N} \\ n_2 < n_1}} \sum_{l_1,l_2 \in \mathsf{L}} \sum_{\lambda_{\mathrm{u}}} \Big[|\hat{\hat{\mu}}(\lambda_{\mathrm{u}}; n_1, l_1; n_2, l_2) - \hat{\mu}^{\mathrm{A}}(n_1, l_1; n_2, l_2)| + \\ |\hat{\hat{\mu}}(\lambda_{\mathrm{u}}; n_1, l_1; n_2, l_2) - \hat{\mu}^{\mathrm{B}}(\lambda_{\mathrm{u}}; l_1; l_2)| \Big], \tag{S4.4}$$

where the last sum runs over discretized values of λ_{u}, C is the total number of terms in the three sums, $\hat{\hat{\mu}}(\cdot)$ is the conditional mean estimate calculated by (S4.2),

$$\hat{\hat{\mu}}(\lambda_{\mathrm{u}}; n_1, l_1; n_2, l_2) = a\hat{\hat{\mu}}^{\mathrm{A}}(n_1, l_1; n_2, l_2) + (1-a)\hat{\hat{\mu}}^{\mathrm{B}}(\lambda_{\mathrm{u}}; l_1; l_2), \tag{S4.5}$$

$\hat{\mu}^{\mathrm{A}}(\cdot)$ and $\hat{\mu}^{\mathrm{B}}(\cdot)$ denote the estimates of the location parameters of distributions A and B, and $\hat{\hat{\mu}}^{\mathrm{A}}(\cdot)$ and $\hat{\hat{\mu}}^{\mathrm{B}}(\cdot)$ denote their NN predictions. a and similarly optimized b determine the weights,

independent of profile attributes, with which statistical parameter estimates obtained for distributions A and B contribute to a conditional mean estimate (Section 2.4 of the main text).

We used the FANN C implementation of the NN model (Nissen, 2012) for training. However, we modified the FANN source code to add the L_2 regularization term to the mean squared error (MSE) function. The regularizer results from placing a normal prior on the NN weights and prevents overfitting (Bishop, 2006). A regularization coefficient of 10^{-6} was used for training an NN model with one hidden layer and four hidden units having hyperbolic tangent activation functions. Regularization and such an NN architecture reduced the model complexity.

Optimal NN weights were found after 100 sessions of training with 50 thousand epochs (iterations). At the beginning of each session, weights were randomly initialized to values in the range $(-0.1, 0.1)$. Figure S5 shows the mean and standard deviation of the MSE of predictors for alignment scores of profiles generated using $S = 1012$ seed profiles with a noise level $r = 0.03$ and a fragment length $s = 9$.

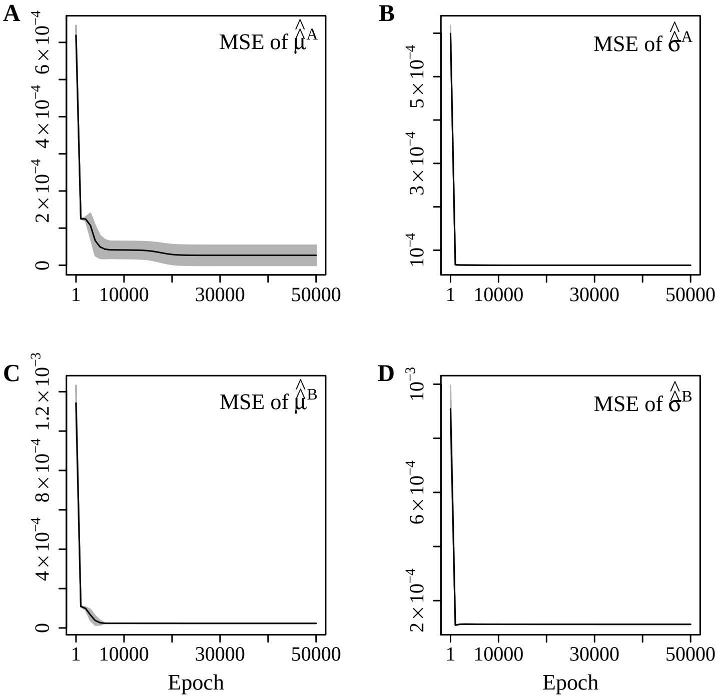

Figure S5. **MSE of the $\hat{\mu}^A$ (A), $\hat{\sigma}^A$ (B), $\hat{\mu}^B$ (C), and $\hat{\sigma}^B$ (D) predictors for alignment scores of profiles generated using $S = 1012$ seed profiles with a noise level $r = 0.03$ and a fragment length $s = 9$.** The solid line represents the mean, and the shaded area represents one standard deviation.

S4.5 Combining dependent p-values

We estimate the statistical significance of profile-profile alignments by combining the significance of the alignment score and the normalized number of positive substitution scores ω_n in the alignment. Both the number of positive substitution scores and the derived statistic ω_n correlate

with the alignment score. To take into account dependence between these measures, we estimate combined statistical significance using the empirical Brown's method (Poole *et al.*, 2016):

$$P = 1 - F_{2y}(\phi/f), \tag{S4.6}$$

where $F_{2y}(\cdot)$ is the distribution function of a chi-squared variable with $2y$ degrees of freedom, $\phi = -2(\log P_\mathrm{a} + \log P_\mathrm{o})$, P_a and P_o are the p-values of the alignment score and ω_n, respectively, $y = (\mathrm{E}(\phi))^2/\,\mathrm{var}(\phi)$, and $f = \mathrm{var}(\phi)/(2\mathrm{E}(\phi))$. The variance $\mathrm{var}(\phi)$ is empirically estimated from the data.

S5 Evaluation

S5.1 Model fitting and goodness-of-fit tests

Describing the behavior of the right tail of alignment score distributions is sufficient for practical purposes. Therefore, we fit models (EVD, GEVD) in the right tail of an alignment score distribution and estimate the model parameters using the maximum likelihood method.

To test the goodness of fit of a model in the right tail, we apply the supremum class upper tail Anderson-Darling test for left-truncated data (Chernobai *et al.*, 2015), where the test statistic AD_up assigns more weight on observations in the right tail of a distribution. However, the weight $\psi(x) = (1 - \hat{F}_\theta(x))^{-1}$, where $\hat{F}_\theta(x)$ is the estimated distribution function, implies that AD_up is bounded from below by $\sqrt{N}$, the square root of the number of observations. Hence, the p-value of the AD_up statistic does not reach high values (see Figure S6A and D), and the distribution of p-values cannot be tested for uniformity under the assumption of the null hypothesis.

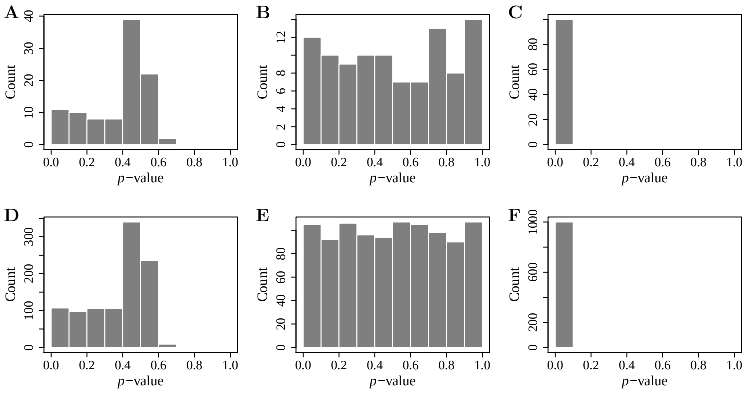

Figure S6. **Distribution of p-values when the null hypothesis is true (A,B,D,E) and false (C,F).** The figure shows the results of the supremum class upper-tail Anderson-Darling test applied to 100 (A,B,C) and 1000 (D,E,F) random samples of size $N = 10\,000$ drawn from the standard normal distribution. (A,D): Results obtained using the original AD_up statistic. (B,E): Results obtained using the modified AD_up statistic. When the null hypothesis is true (B,E), the modified statistic leads to a uniform distribution for p-values as the number of data samples increases. (C,F): The mean of the null distribution differs by 1. Both statistics lead to the same distribution. p-values were obtained by Monte Carlo simulation with 100 samples.

To resolve this issue, we modified the AD_up statistic by excluding from the calculation the last order statistic $x_{(N)}$ of a data sample, which leads to the boundedness of AD_up from below.

Unless N is very small or $x_{(N)}$ represents an extremely high outlier, this modification does not affect the supremum values of AD_{up} that indicate a deviation from the null hypothesis. It also ensures that the p-value is uniform when the null hypothesis is true (Figure S6B and E).

We use Monte Carlo simulation with 100 samples to compute p-values (Chernobai *et al.*, 2015; Wolter, 2012).

S5.2 Assessing statistical accuracy

The comparison of the profiles constructed for 5000 simulated Pfam (Finn *et al.*, 2016) (v30.0) families against the database of simulated profiles representing 4931 SCOPe (Fox *et al.*, 2013) (v2.03) domains constitutes the basis for assessing statistical accuracy. The 5000 Pfam families were selected at random, while the 4931 SCOPe domains formed the training dataset (Section S5.3.1). Every Pfam family represented the source MSA ($S = 1$, $M = 1$) in step 3 of Algorithm S1 (Algorithm 1 of the main text) used to generate one random profile ($R = 1$) based on the Pfam family. Whereas every real profile constructed for each of the 4931 SCOPe domains (Section S5.3.2) represented the seed profile ($S = 1$, $M = 1$) in step 2 of Algorithm S1 used to generate one random profile ($R = 1$) based on the family that the profile built for the SCOPe domain described.

Based on the results reported in the main text, random profiles were generated using the Pfam families or SCOPe profiles as source data with a fragment length $s = 9$. However, there is an important distinction between the process of generating profiles for assessing statistical accuracy and that for the simulation study (Section 2.4 of the main text). In the latter case, the aim is to generate a diverse set of random profiles for the characterization of the distribution of their alignment scores, using a large number of seed profiles ($S \gg 1$) or generating randomly many source MSAs ($M \gg 1$). Yet profiles used for assessing statistical accuracy have to preserve the attributes (ENO and length) and the composition of each of the Pfam families and the profiles constructed for the SCOPe domains. The properties of each real sequence family or profile were mimicked using only one seed profile ($S = 1$) or source MSA ($M = 1$), while a noise level of $r = 0.05$ ensured that the generated random profiles did not share significant similarities.

S5.3 Assessing profile-profile alignment performance

S5.3.1 Datasets The SCOPe database (Fox *et al.*, 2013) (v2.03) of protein domain sequences and structures, filtered to 20% sequence identity, served as the basis for the evaluation of sensitivity and alignment quality. Class g of small proteins was not considered. The domains were divided into training and test datasets by SCOPe folds.

The domains of every second fold, 4931 domains of 546 folds in total, were assigned to the training dataset. The 4900 domains of the remaining 547 folds were assigned to the test dataset.

Profiles constructed for the domains of the training dataset (Section S5.3.2) served as seeds for generating random profiles for assessing statistical accuracy (Section S5.2). A smaller dataset, every fourth fold of the training dataset (1112 domains), was used to evaluate the effect of the adjustment parameters on sensitivity and high-quality alignment rate.

The evaluation of the performance of the COMER method (Margelevičius, 2016, 2018) implementing a new method for estimation of statistical significance and benchmark tests on unseen SCOPe folds were performed using the test dataset. 1722 queries that represented each superfamily but included every fourth domain of larger superfamilies were searched against all the

4900 representatives of the test set to generate results.

S5.3.2 Profile construction Profiles originated from two categories of MSAs. The MSA of the first category for a domain sequence was the result of running PSI-BLAST (Altschul *et al.*, 1997) (v2.2.28+) for six iterations using a sequence inclusion threshold of 10^{-5} and soft masking of low complexity regions against the UniRef50 sequence database (Suzek *et al.*, 2015). The second category of MSAs was obtained by running HHblits (Remmert *et al.*, 2012) for three iterations using default settings against the UniProt20 database of profile HMMs. The final MSAs contained only statistically significant matches. The HHblits MSAs were used to construct profiles for the sequences of the training dataset.

Secondary structure (SS) predictions used by COMER and HHsearch (Section S5.3.3) were calculated using PSIPRED (Jones, 1999).

S5.3.3 Profile-profile alignment methods The performance of the following profile-profile alignment methods was evaluated: a new version implementing a new statistical model and the previous version (v1.4.2) of the COMER method, the HHsearch method (Söding, 2005) (v3.0.0), FFAS (Jaroszewski *et al.*, 2011), and COMPASS (v3.1) (Sadreyev and Grishin, 2008).

If not stated otherwise, a default value of 0.35 for the HHsearch's option of posterior probability threshold for maximum accuracy alignment (-mact) was changed to 0.3. While not affecting sensitivity, the option assigned this value resulted in better alignment quality in both the local and global evaluation modes (Section S5.3.6).

COMPASS profiles were built from input MSAs preprocessed with the `prep_psiblastali` utility, as specified in COMPASS documentation.

The 90 000 top-ranked alignments of each method were evaluated. This number of alignments covered the full range of statistically significant alignments of each method.

S5.3.4 Implementation of the algorithm proposed previously The evaluation of profile-profile alignment methods included a COMER version (referred to as S&G'08) implementing the algorithm for estimating statistical significance proposed by Sadreyev and Grishin (2008).

Following this method, profiles of lengths $l \in \mathsf{L}$ ($\mathsf{L} = \{50, 100, 200, 400, 600, 800\}$) and ENOs $n \in \mathsf{N}$ ($\mathsf{N} = \{2, 4, 6, \ldots, 12\}$) were generated by randomly sampling and concatenating segments of profiles constructed for Pfam (v30.0) MSAs, where the segments matched the order and lengths of SS elements predicted by PSIPRED for the Pfam profiles. We used the generalized EVD (Kotz and Nadarajah, 2000) (GEVD) and the EVD to model the distribution of alignment scores of the generated profiles. The GEVD allows for flexible modeling of the tail behavior of the distribution, similarly as 'power EVD' introduced by Sadreyev and Grishin (2008). However, we obtained better results using the EVD and, therefore, the results of using the EVD are presented. To predict statistical parameters for profiles of any length and ENO, NNs (Section S4.4) trained on the estimates obtained from aligning the generated profiles were employed.

S5.3.5 Sensitivity evaluation Sensitivity to homologous proteins was evaluated using ROC analysis. A pair of aligned domains that belonged to the same SCOPe superfamily or shared statistically significant structural similarity (DALI (Holm *et al.*, 2008) Z-score ≥ 2) was considered a correct match, or true positive (TP). Aligned pairs that did not meet the above criteria but belonged to the same SCOPe fold were considered to have an unknown relationship and were ignored. Other aligned pairs were considered spurious, or false positives (FPs).

Plots of TPs against FPs were produced for top-ranked alignments sorted by reported statistical significance. Of two alignments between the same pair of domains, the one with higher statistical significance was retained. Alignments between identical domains were removed.

The sensitivity was also summarized using the ROC_n score, which is the normalized area under the ROC curve up to n FPs. The difference between two areas and its statistical significance was calculated as this difference divided by the standard error (Hanley and McNeil, 1983) estimated from 100 bootstrap replicates (Robin *et al.*, 2011).

S5.3.6 Alignment quality evaluation Reference-free evaluation of alignment quality (Margelevičius, 2016, 2018) was used. An alignment between two domains was considered to be of high quality if the most accurate structural model generated for one of the two domains, using the other domain as a template, was similar to the real structure. Structural models were generated using MODELLER (Šali and Blundell, 1993) (v9.4), while TM-score (Zhang and Skolnick, 2004) was used to evaluate the structural similarity between a model and the real structure. A high-quality alignment (HQA) has a TM-score ≥ 0.4, which indicates a statistically significant similarity. A TM-score < 0.4 implied an alignment of inferior quality (IQA). An alignment with a TM-score < 0.2, a characteristic value for a random pair, was assumed to be of low-quality (LQA).

Two evaluation modes—local and global—were used. The global mode evaluates the alignment with respect to the entire protein domain, whereas the local mode along the alignment extent. Both modes jointly constitute a comprehensive evaluation.

The alignment quality was also summarized using the ROC_n score calculated for the ROC curve up to n LQAs or IQAs. The statistical significance of the difference between two areas (ROC_n scores) was estimated using 100 bootstrap replicates (see also Section S5.3.5).

S6 Simulation results

S6.1 Alignment scores of profiles generated using $S = 1012$ seed profiles

Section 2.4 of the main text showed that using a fragment length $s = 9$ and a noise level $r = 0.03$ achieves the best balance between the distance between distributions obtained for real and simulated profiles and the goodness of fit to the data. Additional results are presented here.

Figure S7 shows three alignment score distributions for different values of profile attributes (A–C) and compositional similarity (E–G). All distributions and the results of the goodness-of-fit tests for different values of profile attributes are shown in Figure S8 and Table S3. Figure S9 and Table S4 show the results for different values of mutual compositional similarity.

The majority of the goodness-of-fit tests do not reject the null hypothesis (Figure S7D and H). However, the distribution of p-values is not uniform. The procedure of comparing simulated profiles (Section S4.2) accounts for this phenomenon. Profiles of a certain ENO and length are generated only once, and aligning them against all sets of profiles of different ENOs induces correlation between distributions. This correlation can be reduced by generating, using Algorithm S1 (Algorithm 1 of the main text), a new set of profiles before producing each distribution of alignment scores. Such a procedure, however, would considerably increase the computation time, and, therefore, is not applied in this study. Still, we note that for each distribution of alignment scores, one of two sets of random profiles was used only once.

Figure S7I–L and Figure S10 display the degree of match between the alignment score distributions obtained for real profiles and those with the parameters predicted based on the distributions

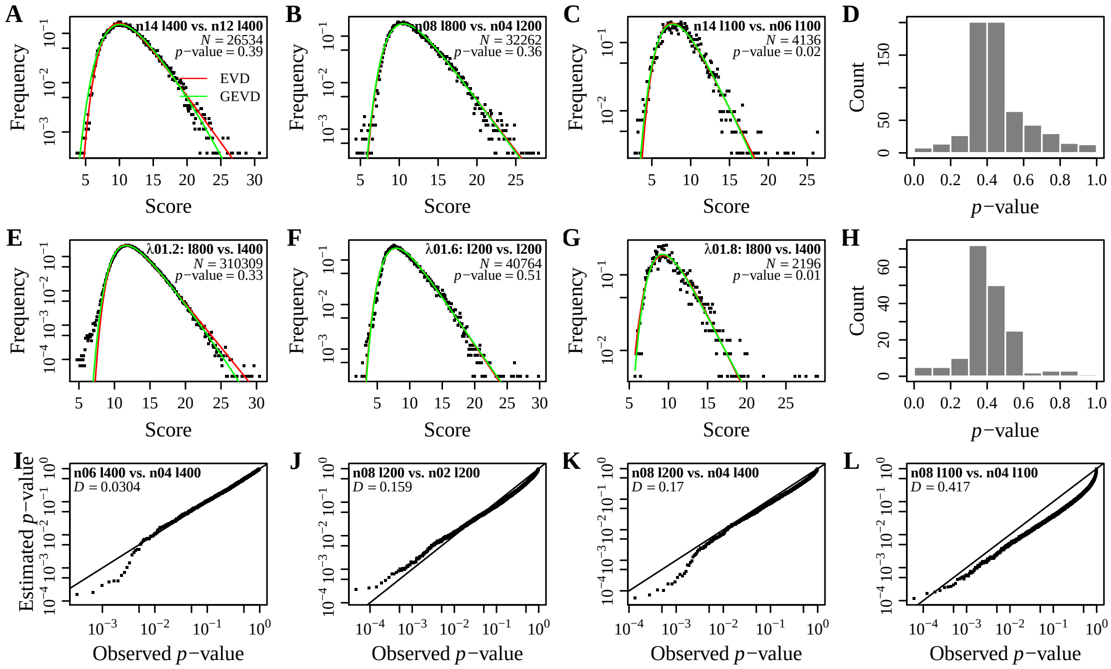

Figure S7. **Distributions for profiles generated using $S = 1012$ seed profiles with $s = 9$ and $r = 0.03$.** Panels (A–C) plot three distributions obtained from aligning simulated profiles of ENO n and length l against profiles with different values of n and l. (C) represents the distribution with some outliers that imply the lowest goodness-of-fit p-value, though it has the shape of an EVD. (D): The distribution of the p-values of goodness-of-fit tests for alignment scores obtained from aligning profiles with different attributes (ENO and length). (E–G) show three alignment score distributions for profiles of specified mutual compositional similarity λ and length l. Similarly as in (C), the distribution for which goodness-of-fit p-value is the lowest is shown in (G). (H): The distribution of the p-values of goodness-of-fit tests for alignment scores obtained from aligning profiles with different compositional similarity and length. (I–L): Observed p-values corresponding to the empirical distribution function obtained for real unrelated profiles against estimated p-values using predicted values of the statistical parameters. (I), (J,K) and (L) represent examples where the distance D between two distribution functions belong to the sets of smallest, average, and largest values, respectively. N, the number of alignment scores. (GEVD fits illustrate the curvature of the empirical distributions and is for illustration only.)

Figure S8. **Distributions of alignment scores of simulated profiles for different pair values of profile ENO and length.** Profiles were generated using $S = 1012$ seed profiles with a noise level $r = 0.03$ and a fragment length $s = 9$. The red and the green curves represent a maximum likelihood fit of the EVD and the GEVD to the data, respectively. N, the number of alignment scores. (*Please find the figure among Additional files.*)

of alignment scores of simulated profiles.

Table S3. **Goodness of fit of the EVD to the distribution of alignment scores of profiles generated using $S = 1012$ seed profiles with $s = 9$ and $r = 0.03$.** Distribution represents distributions obtained from aligning simulated profiles of ENO n and length l against simulated profiles with different values of n and l. The table reports the estimates and their standard errors (SE) for the location and scale parameters of the EVD for each distribution of alignment scores. N is the number of alignment scores. AD_{up} is the upper-tail Anderson-Darling statistic. The p-value of statistic AD_{up} was computed by Monte Carlo simulation with 100 samples. (*Please find the table among Additional files.*)

Figure S9. **Distributions of alignment scores obtained from aligning pairs of simulated profiles with mutual compositional similarity λ and different values of length l.** Profiles were generated using $S = 1012$ seed profiles with a noise level $r = 0.03$ and a fragment length $s = 9$. The red and the green curves represent a maximum likelihood fit of the EVD and the GEVD to the data, respectively. N, the number of alignment scores. (*Please find the figure among Additional files.*)

Table S4. **Goodness of fit of the EVD to the distribution of alignment scores of profiles generated using $S = 1012$ seed profiles with $s = 9$ and $r = 0.03$.** Distribution represents distributions obtained from aligning pairs of simulated profiles with mutual compositional similarity λ and different values of length l. The table reports the estimates and their standard errors (SE) for the location and scale parameters of the EVD for each distribution of alignment scores. N is the number of alignment scores. AD_{up} is the upper-tail Anderson-Darling statistic. The p-value of statistic AD_{up} was computed by Monte Carlo simulation with 100 samples. (*Please find the table among Additional files.*)

Figure S10. **Observed p-values corresponding to the empirical distribution function obtained for real unrelated profiles against estimated p-values using predicted values of the statistical parameters.** The predictions were made based on alignment score distributions for profiles generated using $S = 1012$ seed profiles with a noise level $r = 0.03$ and a fragment length $s = 9$. D is the two-sample KolmogorovSmirnov statistic. (*Please find the figure among Additional files.*)

S6.2 Alignment scores of profiles generated using $S = 1$ seed profile

In the previous subsection, we used a large number of seed profiles and one source MSA ($M = 1$) generated per seed profile to produce a diverse set of simulated profiles. This subsection shows that using only one seed profile for simulating profiles results in diversity of sampled fragments similar to that observed in profiles generated using many seed profiles, provided a large number of source MSAs are generated based on the seed profile. Simulation using one instead of many seed profiles facilitates profile simulation.

The results of employing one seed profile and the optimal fragment length ($s = 9$) and noise level ($r = 0.03$) exhibit a similar distance between distributions obtained for real and simulated profiles and goodness of fit to the data. Figure 2B of the main text shows that seeding from three different profiles representing different SCOPe classes provides the properties of simulated profiles, through adding noise to $M = 1000$ source MSAs, that lead to similar results. Figure S11 summarizes the results obtained using the seed profile constructed for SCOPe domain d1vr5a1. It shows distributions for different values of profile attributes and compositional similarity and also correspondence between distributions obtained for real and simulated profiles.

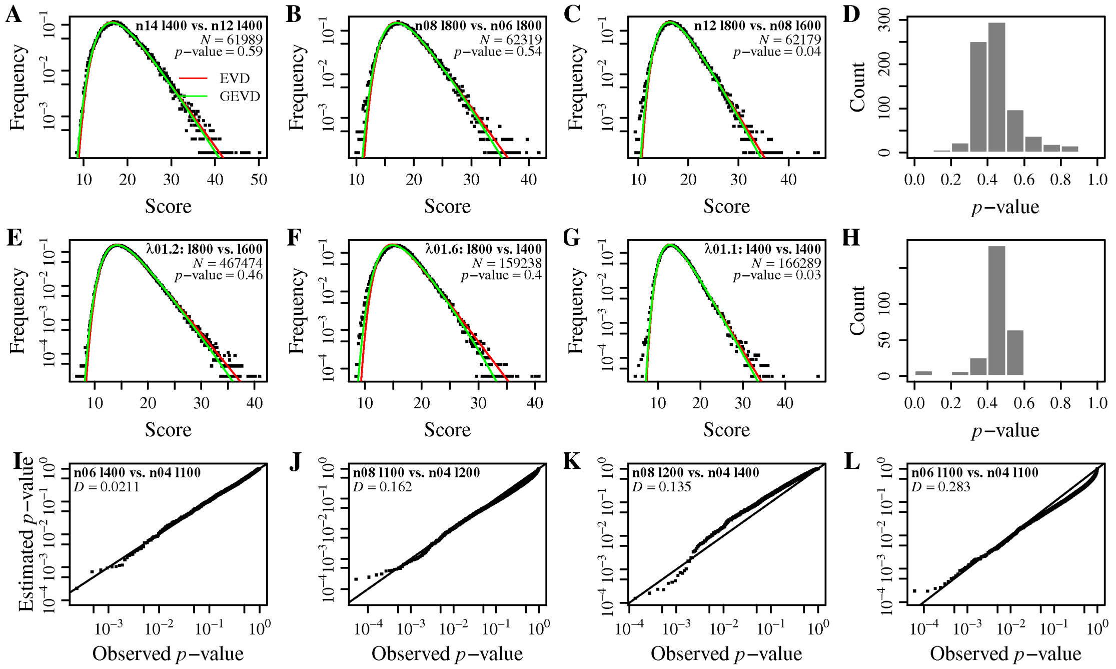

Figure S11. **Distributions for profiles generated using one seed profile ($S = 1$, $M = 1000$), d1vr5a1, with a noise level $r = 0.03$ and a fragment length $s = 9$.** Panels (A–C) plot three distributions obtained from aligning simulated profiles of ENO n and length l against profiles with different values of n and l. (C) represents the distribution with some outliers that imply the lowest goodness-of-fit p-value. (D): The distribution of the p-values of goodness-of-fit tests for alignment scores obtained from aligning profiles with different attributes (ENO and length). (E–G) show three alignment score distributions for profiles of specified mutual compositional similarity λ and length l. The distribution for which goodness-of-fit p-value is the lowest is shown in (G). (H): The distribution of the p-values of goodness-of-fit tests for alignment scores obtained from aligning profiles with different compositional similarity and length. (I–L): Observed p-values corresponding to the empirical distribution function obtained for real unrelated profiles against estimated p-values using predicted values of the statistical parameters. (I), (J,K) and (L) represent examples where the distance D between two distribution functions belong to the sets of smallest, average, and largest values, respectively. N, the number of alignment scores. (GEVD fits illustrate the curvature of the empirical distributions and is for illustration only.)

S6.3 Correlation between the estimates of the EVD parameters

Figure S12 shows the correlation between the estimates of the location and scale parameters obtained (Panel A) from aligning simulated profiles of length $l_1 \in \mathsf{L}$ and ENO $n_1 \in \mathsf{N}$ against profiles of length $l_2 \in \mathsf{L}$ and ENO $n_2 \in \mathsf{N}$ ($n_2 < n_1$) and (Panel B) from aligning profiles of length $l_1 \in \mathsf{L}$ against profiles of length $l_2 \in \mathsf{L}$ with discretized mutual compositional similarity λ_u.

S6.4 Distribution of the number of positive substitution scores

One of the criteria for judging whether two sequences are homologous and how closely related they are is the number of identical amino acid matches in the sequence alignment. A high

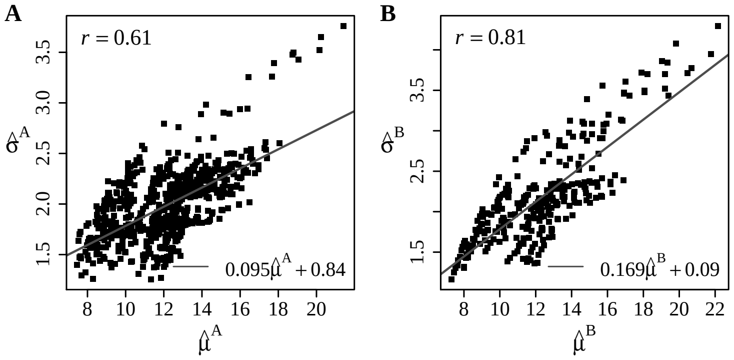

Figure S12. **Plots of the estimates of the scale parameters against those of the location parameters of the alignment score distributions obtained for profiles generated using a noise level $r = 0.03$ and a fragment length $s = 9$ and characterized by (A) ENO and length and (B) compositional similarity and length.** The line represents a linear fit to the data. r, Pearson's correlation coefficient.

proportion of identical matches increases the probability for two sequences to be homologous when the alignment covers a large proportion of the sequence lengths.

The corresponding criterion for profiles is the number of positive profile-profile substitution scores in the alignment. Specifying how the number of positive substitution scores is distributed for unrelated profiles is useful for estimating the statistical significance of profile-profile alignments.

Figure S13 and Table S5 show that the distribution (especially its right tail) of the number of positive substitution scores observed in alignments between unrelated profiles of different values of ENO and length can be accurately approximated by the negative binomial distribution (NBD).

Figure S13. **Distributions of the number of positive substitution scores observed in alignments of simulated profiles of different values of ENO and length.** Profiles were generated using $S = 1012$ seed profiles with a noise level $r = 0.03$ and a fragment length $s = 9$. The red points represent a least-squares fit of the NBD to the data. N, the number of alignments between simulated profiles. The p-values of the AD_{up} statistic were computed by Monte Carlo simulation with 100 samples. (*Please find the figure among Additional files.*)

Table S5. **Goodness of fit of the NBD to the distribution of the number of positive substitution scores observed in alignments of simulated profiles.** Distribution represents distributions obtained from aligning simulated profiles of ENO n and length l against simulated profiles with different values of n and l. Profiles were generated using $S = 1012$ seed profiles with a noise level $r = 0.03$ and a fragment length $s = 9$. The table reports the estimates and the Cramér–Rao lower bound on their standard errors (SE) for the shape (the number of non-positive scores) and probability (of having a positive score) parameters of the NBD for each distribution of the number of positive scores. N is the number of alignments between simulated profiles. AD_{up} is the upper-tail Anderson-Darling statistic. The p-value of statistic AD_{up} was computed by Monte Carlo simulation with 100 samples. (*Please find the table among Additional files.*)

Figure S14 shows that the normalized number of positive substitution scores ω_{n} introduced in Eq (7) of the main text is also approximately distributed as an NBD.

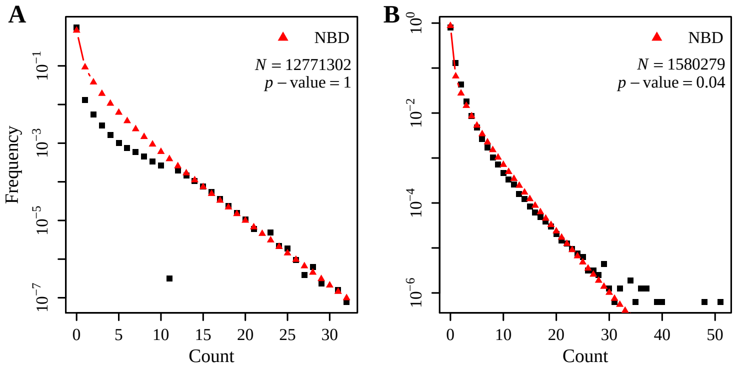

Figure S14. **Distribution of the ω_n statistic.** The red points represent a least-squares fit of the NBD to the data. N is the number of alignments between profiles. The p-values of the AD_{up} statistic (3573.7 and 18403.9 in A and B, respectively) were computed by Monte Carlo simulation with 100 samples. (A): Distribution of the ω_n statistic calculated from alignments of simulated profiles of different values of ENO and length. Profiles were generated using $S = 1012$ seed profiles with a noise level $r = 0.03$ and a fragment length $s = 9$. The outlier at $\omega_n = 11$ is due to the discreteness of the lengths of the simulated profiles, where the smallest increment in length was 50. (B): Distribution of the ω_n statistic calculated from alignments of real unrelated profiles (unaligned by DALI or DALI Z-score < 2) constructed for 4915 protein domains selected randomly from the SCOPe database (v2.03) filtered to 20% sequence identity.

S6.5 Optimal adjustment parameters

The weights a and b in the conditional mean estimators and the adjustment parameters $W = \{g_s, g_i, g_c, h_s, h_i, h_c\}$ in (5) and (6) of the main text are optimized with respect to statistical accuracy and alignment quality and sensitivity. The optimal values represent final values used in the application.

For assessing statistical accuracy, using COMER implementing the proposed method for estimating statistical significance, we search and align the profiles constructed for 5000 simulated Pfam (v30.0) sequence families against the database of simulated profiles representing 4931 SCOPe (v2.03) domains (Section S5.2). Then, we calculate the fraction of queries with a p-value reported by COMER for their best match less than the specified p-value. Statistical accuracy is also evaluated by calculating the maximum distance D_t between observations (fraction of queries) and theoretical predictions up to a significance level of 0.01. D_t thus corresponds to the Kolmogorov-Smirnov statistic for truncated data.

The evaluation of sensitivity and alignment quality (Sections S5.3.5 and S5.3.6) is based on a small subset of 1112 domains of the training dataset (Section S5.3.1).

The results are shown in Figures S15 and S16 and Table S6.

S7 Application results

S7.1 Improvement in high-quality alignment rate

This section provides additional results from alignment quality analysis. Table S7 and Figures S17 and S18 show the results of examining the number of HQAs as a function of the number of alignments of inferior quality (IQAs).

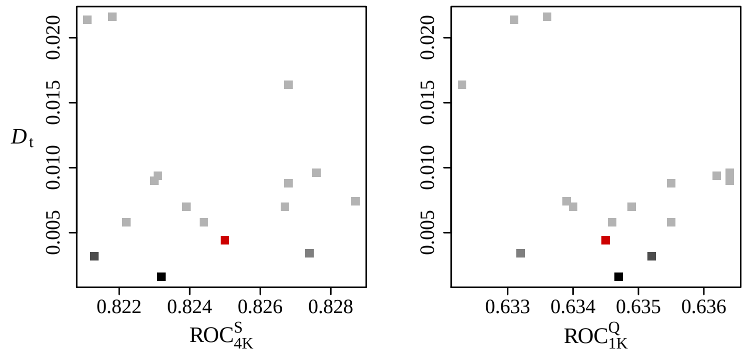

Figure S15. **Statistical accuracy against sensitivity and alignment quality for different values of the adjustment parameters.** D_t is the Kolmogorov-Smirnov statistic for the truncated distribution function of the fraction of queries with a p-value reported for their best match less than a specified value. ROC^S_{4K} and ROC^Q_{1K} are the normalized areas under the ROC curves up to 4000 false positives and up to 1000 low-quality alignments, respectively, and quantify the sensitivity and alignment quality of COMER alignments evaluated on the training dataset. Each point represents a particular set of values of the adjustment parameters. The red point represents a tradeoff between statistical accuracy and sensitivity and alignment quality. Three points of darker gray shade exhibit higher statistical accuracy but lower sensitivity or alignment quality.

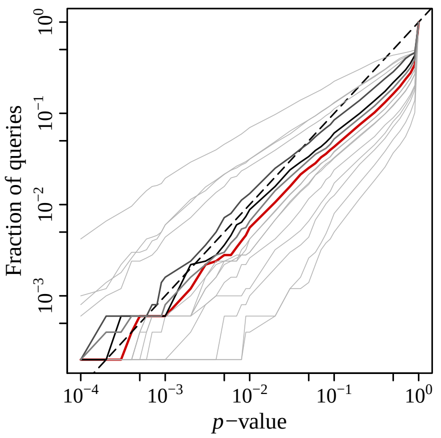

Figure S16. **Statistical accuracy of the COMER method.** The figure shows the results of comparing the profiles constructed for 5000 randomized Pfam families against the profiles constructed for 4931 randomized SCOPe domains. It plots the fraction of queries with a p-value reported for their best match less than the p-value indicated on the x-axis. The dashed line indicates the highest accuracy. Each solid line represents a particular set of values of the adjustment parameters. The color coding matches that of Figure S15: The red line represents a tradeoff between statistical accuracy and sensitivity and alignment quality. Three lines of darker gray shade exhibit higher statistical accuracy but correspond to lower sensitivity or alignment quality.

g_s	g_i	g_c	h_s	h_i	h_c	a	b	D_t (D)	$\text{ROC}^{\text{S}}_{\text{4K}}$	$\text{ROC}^{\text{Q}}_{\text{1K}}$
0.01	1.0	0.10	0.1	12	4	0.35	0.35	0.0164 (0.4452)	0.8268	0.6323
**0.05**	**1.0**	**0.10**	**0.1**	**12**	**4**	**0.35**	**0.35**	**0.0044 (0.5574)**	**0.8250**	**0.6345**
0.10	1.0	0.10	0.1	12	4	0.35	0.35	0.0090 (0.7200)	0.8230	0.6364
0.05	0.5	0.10	0.1	12	4	0.35	0.35	0.0216 (0.4290)	0.8218	0.6336
0.05	2.0	0.10	0.1	12	4	0.35	0.35	0.0096 (0.7982)	0.8276	0.6364
0.05	1.0	0.05	0.1	12	4	0.35	0.35	0.0598 (0.4060)	0.8273	0.6323
0.05	1.0	0.15	0.1	12	4	0.35	0.35	0.0094 (0.7612)	0.8231	0.6362
0.05	1.0	0.10	0.2	12	4	0.35	0.35	0.0058 (0.5980)	0.8244	0.6346
0.05	1.0	0.10	0.3	12	4	0.35	0.35	0.0070 (0.6340)	0.8239	0.6349
0.05	1.0	0.10	0.1	10	4	0.35	0.35	0.0016 (0.4694)	0.8232	0.6347
0.05	1.0	0.10	0.1	14	4	0.35	0.35	0.0070 (0.6292)	0.8267	0.6340
0.05	1.0	0.10	0.1	12	2	0.35	0.35	0.0032 (0.4408)	0.8213	0.6352
0.05	1.0	0.10	0.1	12	6	0.35	0.35	0.0074 (0.6966)	0.8287	0.6339
0.05	1.0	0.10	0.1	12	4	0.15	0.35	0.0058 (0.5980)	0.8222	0.6355
0.05	1.0	0.10	0.1	12	4	0.55	0.35	0.0034 (0.5082)	0.8274	0.6332
0.05	1.0	0.10	0.1	12	4	0.35	0.15	0.0214 (0.4324)	0.8211	0.6331
0.05	1.0	0.10	0.1	12	4	0.35	0.55	0.0088 (0.7054)	0.8268	0.6355

Table S6. **Dependence of the statistical accuracy and performance of the COMER method on the adjustment parameters.** D_t and D are the Kolmogorov-Smirnov statistics for the distribution function of the fraction of queries with the p-value reported for their best match less than a specified value. D_t is the statistic for truncated data. $\text{ROC}^{\text{S}}_{\text{4K}}$ is the normalized area under the ROC curve up to 4000 false positives (FPs) and quantifies the sensitivity of COMER alignments evaluated on the training dataset. $\text{ROC}^{\text{Q}}_{\text{1K}}$ is the normalized area under the ROC curve up to 1000 low-quality alignments (LQAs) and quantifies the quality of COMER alignments evaluated on the training set in the global mode. Alignment quality evaluated in the local mode correlates with that evaluated in the global mode and is not shown. Values highlighted in bold represent a tradeoff between statistical accuracy and sensitivity and alignment quality.

S7.2 Statistical analysis with respect to false positives

We provide statistical analysis with respect to false positives (FPs) found among the top-ranked alignments of the queries from the test dataset. We calculated the fraction of queries with a p-value reported by a profile-profile alignment method for the top-ranked FP less than the specified p-value. The p-value P for an HHsearch and COMPASS alignment was calculated from the reported E-value E using the equation $P = 1 - \exp(-E)$. The p-value for an FFAS alignment was calculated from the reported Z-score (Rychlewski *et al.*, 2000). However, we obtained a nearly flat curve for FFAS, and do not show it.

The results are shown in Figure S19. They are not unambiguous though. Classifying a pair of aligned protein domains as an FP (TP) depends on the presence or absence of an established evolutionary relationship and structural similarity between the protein domains (Section S5.3.5). Still, profile-profile local alignment methods may detect an evolutionary signal at a local scale, which becomes obscured at the global scale. For example, 58.8% (67/114; HHblits MSAs) and 41.0% (43/105; PSI-BLAST MSAs) of the top-ranked FPs found by the new version of the COMER method with a p-value ≤ 0.01 aligned with queries so that the similarity between a structural model and the real query structure was statistically significant when evaluated along the alignment extent. Hence, some of the statistically significant matches between queries and FPs represented in Figure S19 may indicate a strong evolutionary signal.

Input	Evaluation	COMER new			COMER new (alt.)		
		TPs (+%)	$\text{TPs}_{\text{v1.4}}$	FDR	TPs (+%)	$\text{TPs}_{\text{v1.4}}$	FDR
HHblits MSAs	Local	14102 (7.4)	13126	0.005	46312 (3.1)	44910	0.026
	Global	4960 (41.7)	3500	0.001	10366 (35.7)	7639	0.015
	Local (max ext)	13004 (28.6)	10114	0.008	11995 (18.6)	10114	0.008
	Global (max ext)	3700 (64.2)	2254	0.001	2971 (31.8)	2254	0.001
PSI-BLAST MSAs	Local	24435 (102.8)	12048	0.030	5701 (193.3)	1944	0.016
	Global	760 (91.9)	396	0.002	5120 (167.9)	1911	0.020
	Local (max ext)	760 (91.9)	396	0.002	5263 (187.1)	1833	0.016
	Global (max ext)	760 (91.9)	396	0.002	5125 (164.4)	1938	0.019

Table S7. **Improvement of the COMER method.** The results of the evaluation of the alignment quality (Local and Global evaluation modes) of versions of the COMER method using profiles constructed from HHblits and PSI-BLAST MSAs are shown. “max ext” denotes the evaluation of maximally extended COMER alignments. TPs stands for the number of high-quality alignments at a specified rate of alignments of inferior quality (FDR) for a new version of the COMER method. $\text{TPs}_{\text{v1.4}}$ represents the same number for the previous COMER version. The percentage improvement with respect to $\text{TPs}_{\text{v1.4}}$ is given in parentheses.

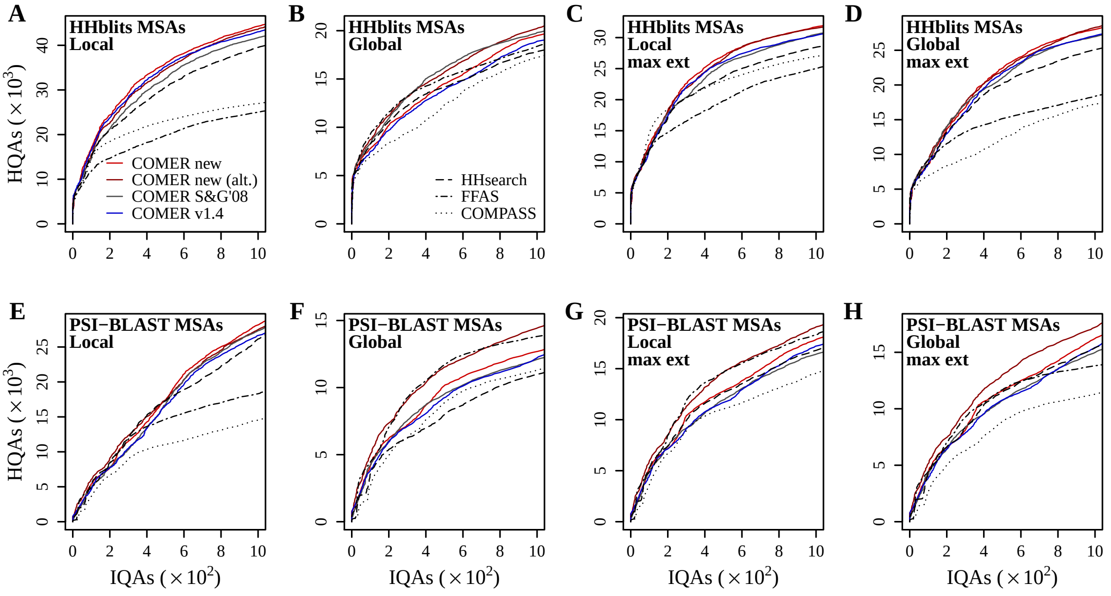

Figure S17. **Alignment quality of profile-profile alignment methods evaluated in the (A,C,E,G) local and (B,D,F,H) global evaluation modes.** Maximally extended (max ext) COMER and HHsearch alignments are evaluated in (C,D,G,H). Profiles were constructed from (A–D) HHblits and (E–H) PSI-BLAST MSAs. COMER v1.4 represents the previous version of the COMER method. COMER S&G’08 implements a statistical model based on previous research. HQA and IQA stand for high-quality alignment and alignment of inferior quality, respectively. The figure displays results for IQAs up to 1000. See Figure S18 for full range.

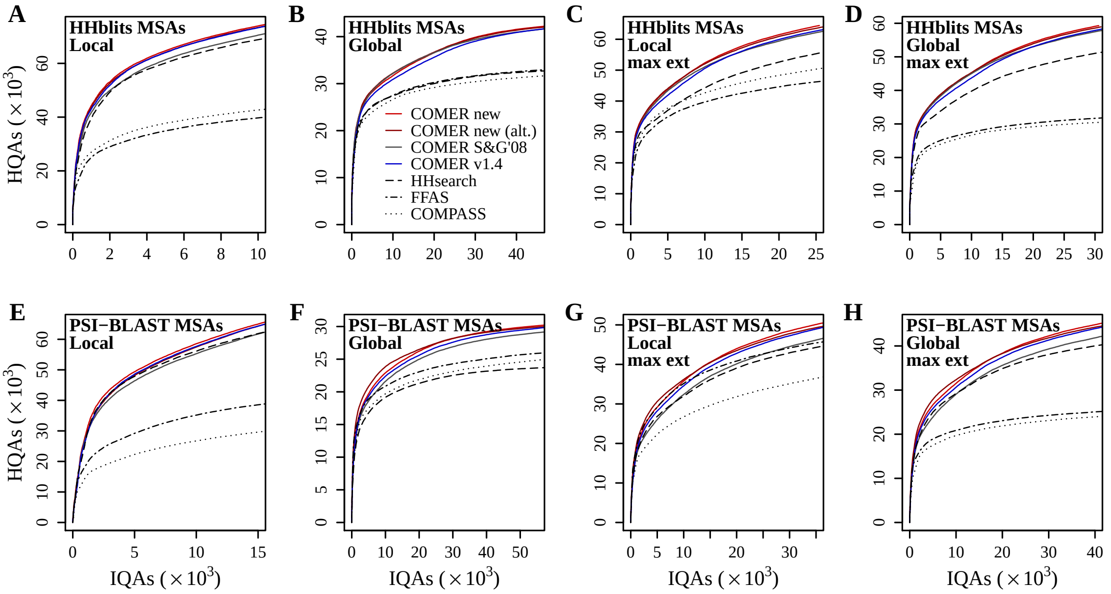

Figure S18. **Same as Figure S17 except that the results of all top-ranked alignments are displayed.**

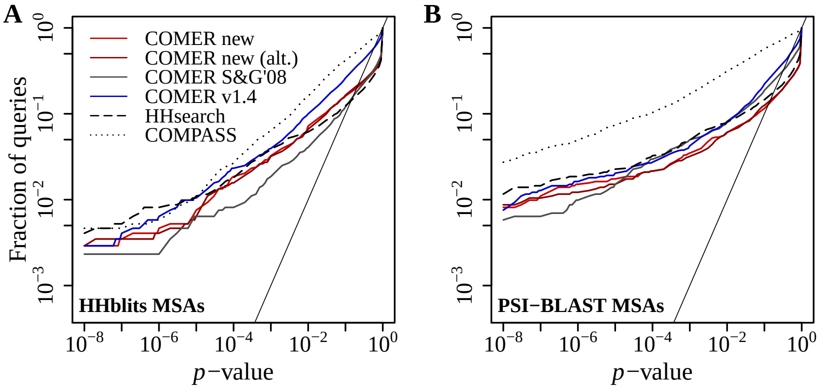

Figure S19. **Statistical accuracy of profile-profile alignment methods evaluated on the test dataset of real protein domains.** The figure plots the fraction of queries with a p-value reported for their top-ranked false positive less than the p-value indicated on the x-axis. The straight solid line represents expected values for unrelated matches. COMER new and new (alt.) represent the COMER method using two variants of the new statistical model. COMER S&G'08 implements a statistical model based on previous research. COMER v1.4 represents the previous version of the COMER method. The alignment methods used profiles constructed from (A) HHblits and (B) PSI-BLAST MSAs.

S7.3 Application to pairwise profile HMM alignments

In this section, we evaluate the effect of the methodology developed for estimating statistical significance when it is applied to pairwise profile HMM alignments produced by HHsearch.

For each alignment it produces, HHsearch separately reports the alignment score of profile HMMs and the similarity score of their SSs. The probability Prob expresses biological significance of a match with these scores (Söding *et al.*, 2015). However, the probability Prob is not easily interpretable from a statistical point of view. We, therefore, focus on the distribution of pairwise profile HMM alignment scores.

We applied the same steps to perform statistical inference that have been described in the main text and Section S4. 1. Random profile HMMs of different values of ENO and length were generated by Algorithm S1 using a fragment length $s = 9$ and a noise level $r = 0.03$. 2. The generated profile HMMs with different ENO values were aligned. 3. The EVD was fitted to each alignment score distribution obtained. 4. The artificial neural network models were trained to predict the statistical parameters based on profile attributes (ENO and length) and compositional similarity between profiles. 5. The adjustment parameters were optimized. 6. The NBD was fitted to the distribution of the normalized number of positive substitution scores.

There is one difference from what has been presented in the text. We could not calculate the parameter λ_{u}, a measure of compositional similarity between profiles, without modifying the HHsearch source code. Instead, we used an exponential function of a symmetrized Kullback-Leibler divergence (Section S4.3) to measure the compositional similarity between two profile HMMs in step 4. Although not being as effective as λ_{u}, it allows the application of the conditional mean estimator for combining independently predicted statistical parameters.

Next, we evaluated statistical accuracy and performance. When evaluating statistical accuracy, E-values reported by HHsearch were converted to p-values (more accurate than reported ones) using the equation $P = 1 - \exp(-E)$.

For performance evaluation, the 90 000 most significant HHsearch alignments sorted by probability Prob were re-ranked by reported E-value. These baseline HHsearch results were compared with the results obtained by re-estimating the statistical significance of the same 90 000 top-ranked HHsearch alignments using the new statistical model (Figure 3 in the main text).

The results are shown in Figure S20. The new statistical model improved statistical accuracy: $D_{\mathrm{t}} = 0.0042$ when statistical significance was re-estimated (HHsearch re-est.) as compared to $D_{\mathrm{t}} = 0.0086$ when using HHsearch E-values. Additionally, re-estimated statistical significance led to improved performance (Figure S20) with most improvements being statistically significant (Table S8). Improved sensitivity and HQA rate corresponded to an increase of up to 7.2% in the number of TPs and up to 55.3% in the number of HQAs, respectively (Table S9).

These results reveal the effectiveness of the developed methodology and improvements in statistical accuracy and performance with respect to a procedure employed by HHsearch for estimating statistical significance, which is already dependent upon profile ENO and length (Söding *et al.*, 2015). Still, the application of the methodology with restrictions illustrates that the purpose was only to demonstrate its effectiveness.

The results also show that HHsearch underestimates statistical significance. While this property can benefit iterative searches performed by HHblits, it can also lead to reduced sensitivity when conducting either one iteration of HHblits or an HHsearch search.

S8 Software and data availability

A new version (v1.5.1) of the COMER software that employs the implementation of the new method for the estimation of statistical significance is available at https://sourceforge.net/projects/comer. The COMER software is also available on Github at https://github.com/minmarg/comer and as a Docker image (https://hub.docker.com/r/minmar/comer). Option SSEMODEL allows the

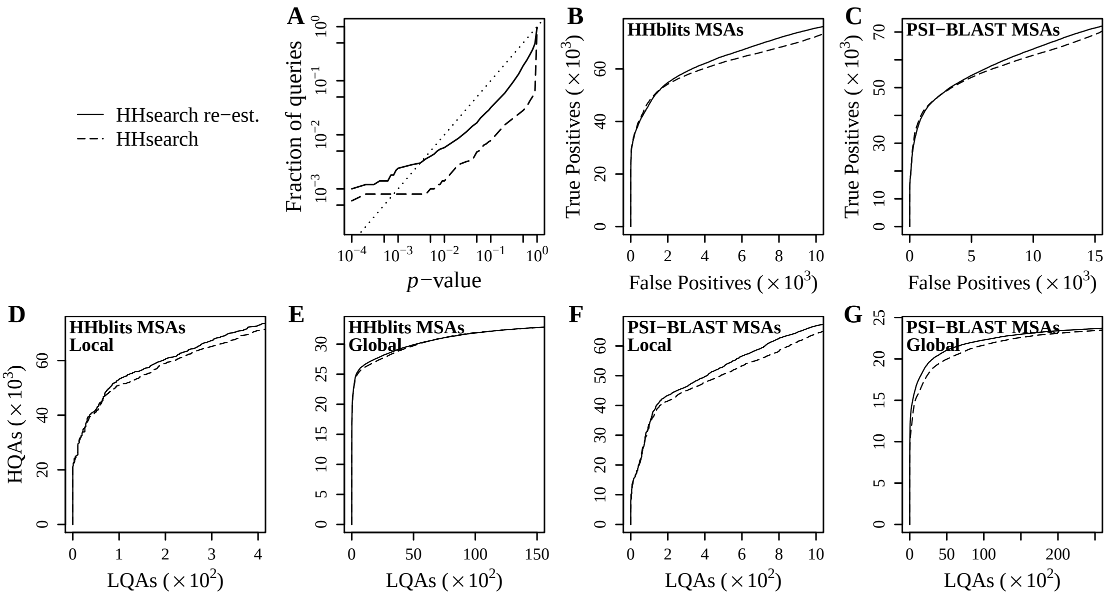

Figure S20. **Statistical accuracy (A) and performance (B–G) of the HHsearch method.** (A): The profile HMMs constructed for 5000 randomized Pfam families were compared to the profile HMMs constructed for 4931 randomized SCOPe domains to evaluate statistical accuracy (see Section S5.2 for details). (B,C): Sensitivity. (D–G): Alignment quality evaluated in the (D,F) local and (E,G) global evaluation modes. Profile HMMs were constructed from (B,D,E) HHblits and (C,F,G) PSI-BLAST MSAs. The solid line (HHsearch re-est.) represents the results obtained from re-estimating the statistical significance of the 90 000 top-ranked HHsearch alignments (B–G). HQA, high-quality alignment. LQA, low-quality alignment.

Input	Evaluation	x	HHsearch re-est.		HHsearch
			ROC$_x$	Z (p-value)	ROC$_x$
HHblits MSAs	Sensitivity	10000	0.761	10.0 ($<3\times10^{-16}$)	0.737
PSI-BLAST MSAs	Sensitivity	15000	0.701	7.0 (2.9×10^{-12})	0.684
HHblits MSAs	Local	400	0.708	2.2 (0.028)	0.686
	Global	15000	0.370	0.7 (0.501)	0.369
PSI-BLAST MSAs	Local	1000	0.622	4.3 (1.5×10^{-5})	0.592
	Global	25000	0.268	3.5 (5.3×10^{-4})	0.260

Table S8. **Area under the ROC curve and improvement of the HHsearch method.** The sensitivity and alignment quality (Local and Global evaluation modes) of the HHsearch method are evaluated. Profiles were constructed from HHblits and PSI-BLAST MSAs. ROC$_x$ is the ROC score calculated up to x false positives (FPs; Sensitivity) or low-quality alignments (LQAs; alignment quality in the Local and Global modes). Z is the difference between the areas (ROC$_x$ scores) obtained for differently ranked HHsearch alignments, divided by the estimated standard error. The statistical significance of Z is indicated in parentheses. HHsearch re-est. represents the results obtained from re-estimating the statistical significance of the 90 000 top-ranked HHsearch alignments.

Input	Evaluation	HHsearch		
		$\text{TPs}_{\text{re-est.}}$ (+%)	TPs	FDR
HHblits MSAs	Sensitivity	73448 (7.2)	68497	0.107
PSI-BLAST MSAs	Sensitivity	68158 (6.4)	64087	0.156
HHblits MSAs	Local	58832 (6.0)	55509	0.003
	Global	17770 (3.0)	17246	0.001
PSI-BLAST MSAs	Local	63297 (11.0)	57003	0.013
	Global	8217 (55.3)	5290	0.001

Table S9. **Effect of re-estimating the statistical significance of HHsearch alignments on sensitivity and HQA rate.** TPs stands for the number of true positives (Sensitivity) or high-quality alignments (in the Local and Global evaluation modes) at a specified false discovery rate (FDR) for the HHsearch method. $\text{TPs}_{\text{re-est.}}$ represents the same number having been given the re-estimated statistical significance of the 90 000 top-ranked HHsearch alignments. The percentage improvement with respect to TPs is given in parentheses. Profile HMMs were constructed from HHblits and PSI-BLAST MSAs.

user to choose between implemented statistical models. The software package contains programs to generate random MSAs and profiles. Other programs and scripts used in simulations and performance evaluation, dataset information and alignment data are available at https://sourceforge.net/projects/comer/files/comer-pub-data-1.05.

A Appendix: Conditional mean estimators for the EVD parameters

We define the conditional distributions of the EVD parameters in Section A.1. The required prior distributions for the parameters are obtained in Section A.2. Finally, Section A.3 derives conditional mean estimators for the EVD parameters.

A.1 Conditional distribution for the parameters

This section derives the conditional distribution of an EVD parameter given two sets of the parameters of the distribution that the EVD parameter is assumed to have (see Section A.3).

Let $\theta^{\mathrm{A}} = \theta^{\mathrm{A}}(D^{\mathrm{A}})$, $D^{\mathrm{A}} = \{n_1, l_1; n_2, l_2\}$, denote the set of parameters that characterize the distribution of an EVD parameter x (location or scale) estimated from the distribution of alignment scores obtained by aligning profiles of ENO n_1 and length l_1 against profiles of ENO n_2 and length l_2. Let further $\theta^{\mathrm{B}} = \theta^{\mathrm{B}}(D^{\mathrm{B}})$, $D^{\mathrm{B}} = \{c; l_1; l_2\}$, denote the set of parameters of the distribution of x estimated independently from the distribution of the scores of alignments between profiles of length l_1 and profiles of length l_2 whose mutual compositional similarity is c. We aim to find the conditional distribution $p(x|\theta^{\mathrm{A}}, \theta^{\mathrm{B}})$.

Using Bayes' theorem,

$$p(x|\theta^{\mathrm{A}}, \theta^{\mathrm{B}}) = \frac{p(\theta^{\mathrm{A}}, \theta^{\mathrm{B}}|x)p(x)}{\int p(\theta^{\mathrm{A}}, \theta^{\mathrm{B}}|x')p(x')dx'}. \tag{A.1}$$

Scores obtained by aligning profiles described by the set of attributes D^{A} integrate alignment scores of profile pairs with any mutual compositional similarity. On the other hand, alignment scores of profile pairs characterized by the set of attributes D^{B} include scores obtained for profiles of any ENO. Although profile lengths l_1 and l_2 common to both sets of attributes introduce dependency, two distributions of alignment scores associated with the two sets of attributes will be weakly correlated for the reasons discussed above. Therefore, we assume that θ^{A} and θ^{B} are conditionally independent given x:

$$p(\theta^{\mathrm{A}}, \theta^{\mathrm{B}}|x) = p(\theta^{\mathrm{A}}|x)p(\theta^{\mathrm{B}}|x).$$

We also have $p(\theta|x) = p(x|\theta)p(\theta)/p(x)$ for $\theta = \theta^{\mathrm{A}}, \theta^{\mathrm{B}}$. Then,

$$p(x|\theta^{\mathrm{A}}, \theta^{\mathrm{B}}) = \frac{p(x|\theta^{\mathrm{A}})p(\theta^{\mathrm{A}}) \times p(x|\theta^{\mathrm{B}})p(\theta^{\mathrm{B}})}{p(x) \int p(x'|\theta^{\mathrm{A}})p(\theta^{\mathrm{A}})p(x'|\theta^{\mathrm{B}})p(\theta^{\mathrm{B}})/p(x')dx'}$$

or

$$p(x|\theta^{\mathrm{A}}, \theta^{\mathrm{B}}) \propto \frac{p(x|\theta^{\mathrm{A}})p(x|\theta^{\mathrm{B}})}{p(x)}. \tag{A.2}$$

A.2 Prior distribution for the parameters

We assume noninformative priors for the EVD parameters. The Jeffreys prior is $p(x) \propto \sqrt{J(x)}$, where $J(x)$ is the Fisher information for x (Gelman *et al.*, 2004).

A.2.1 Location parameter $J(\mu)$ for the location parameter μ is $J(\mu) = \mathrm{E}\Big[\Big(\frac{d}{d\mu}\log p(y|\mu,\sigma)\Big)^2\Big|\mu\Big] = -\mathrm{E}\Big[\frac{d^2}{d\mu^2}\log p(y|\mu,\sigma)\Big|\mu\Big]$, where $p(y|\mu,\sigma)$ represents the density of the alignment score y distributed as an EVD with location μ and scale σ.

We have $\log p(y|\mu,\sigma) = -\log\sigma - \frac{y-\mu}{\sigma} - \exp(-\frac{y-\mu}{\sigma})$ and $\frac{d^2}{d\mu^2}\log p(y|\mu,\sigma) = -\frac{1}{\sigma^2}\exp(-\frac{y-\mu}{\sigma})$. Then, using the substitution $z = \exp(-\frac{y-\mu}{\sigma})$, we get

$$
\begin{aligned}
J(\mu) &= \frac{1}{\sigma^2}\int_{-\infty}^{\infty} e^{-\frac{y-\mu}{\sigma}}\sigma^{-1}e^{-\frac{y-\mu}{\sigma}}\exp\{-e^{-\frac{y-\mu}{\sigma}}\}dy \\
&= \frac{1}{\sigma^2}\int_0^{\infty} ze^{-z}dz = \frac{1}{\sigma^2}. \qquad (A.3)
\end{aligned}
$$

Hence,

$$
p(\mu) \propto 1. \qquad (A.4)
$$

A.2.2 Scale parameter The second derivative of the logarithm of the density with respect to the scale parameter σ is $\frac{d^2}{d\sigma^2}\log p(y|\mu,\sigma) = \frac{1}{\sigma^2} - \frac{2(y-\mu)}{\sigma^3} + \frac{2(y-\mu)}{\sigma^3}\exp(-\frac{y-\mu}{\sigma}) - \frac{(y-\mu)^2}{\sigma^4}\exp(-\frac{y-\mu}{\sigma})$. The Fisher information is then minus the sum of the expectations of each of these terms.

The first term is a constant. The expectation of the second term is

$$
\begin{aligned}
\mathrm{E}\Big[-\frac{2(y-\mu)}{\sigma^3}\Big|\sigma\Big] &= -\frac{2}{\sigma^2}\int_{-\infty}^{\infty}\underbrace{\exp\{-e^{-\frac{y-\mu}{\sigma}}\}}_{u}\underbrace{\frac{1}{\sigma}\frac{y-\mu}{\sigma}e^{-\frac{y-\mu}{\sigma}}dy}_{dv} \qquad (A.5) \\
&= -\frac{2}{\sigma^2}\Bigg[\Big(-\frac{y-\mu}{\sigma}-1\Big)e^{-\frac{y-\mu}{\sigma}}\exp\{-e^{-\frac{y-\mu}{\sigma}}\}\Big|_{-\infty}^{\infty} \qquad (A.6) \\
&\quad + \int_{-\infty}^{\infty}\Big(\frac{y-\mu}{\sigma}+1\Big)e^{-\frac{y-\mu}{\sigma}}\frac{1}{\sigma}e^{-\frac{y-\mu}{\sigma}}\exp\{-e^{-\frac{y-\mu}{\sigma}}\}dy\Bigg] \qquad (A.7) \\
&= \mathrm{E}\Big[-\frac{2(y-\mu)}{\sigma^3}e^{-\frac{y-\mu}{\sigma}}\Big|\sigma\Big] - \frac{2}{\sigma^2}. \qquad (A.8)
\end{aligned}
$$

The result follows from integration by parts applied to (A.5), where we obtain $v = (-\frac{y-\mu}{\sigma} - 1)\exp(-\frac{y-\mu}{\sigma})$. (A.6) equals 0, which follows from applying l'Hôpital's rule to the limits

$$
\lim_{y\to\infty}\frac{(\frac{y-\mu}{\sigma}+1)}{e^{\frac{y-\mu}{\sigma}}\exp\{e^{-\frac{y-\mu}{\sigma}}\}} = 0 \quad\text{and}\quad \lim_{y\to-\infty}\frac{(\frac{y-\mu}{\sigma}+1)e^{-\frac{y-\mu}{\sigma}}}{\exp\{e^{-\frac{y-\mu}{\sigma}}\}} = 0.
$$

The first term of (A.7) is the expectation $\mathrm{E}\Big[\frac{(y-\mu)}{\sigma}e^{-\frac{y-\mu}{\sigma}}\Big|\sigma\Big]$. The second term of (A.7) is equal to 1 by (A.3).

Using the substitution $z = \exp(-\frac{y-\mu}{\sigma})$, the expectation of the last term of $\frac{d^2}{d\sigma^2}\log p(y|\mu,\sigma)$ is

$$\begin{aligned} \mathrm{E}\Big[-\frac{(y-\mu)^2}{\sigma^4}e^{-\frac{y-\mu}{\sigma}}\Big|\sigma\Big] &= -\frac{1}{\sigma^2}\int_{-\infty}^{\infty}\frac{(y-\mu)^2}{\sigma^2}e^{-\frac{y-\mu}{\sigma}}\frac{1}{\sigma}e^{-\frac{y-\mu}{\sigma}}\exp\{-e^{-\frac{y-\mu}{\sigma}}\}dy \\ &= -\frac{1}{\sigma^2}\int_0^{\infty}\log^2(z)ze^{-z}dz \qquad \text{(A.9)} \\ &< -\frac{1}{\sigma^2}\int_0^{\infty}e^{(1-\delta)z}e^{-z}dz = -\frac{1}{\sigma^2}\frac{1}{\delta} \quad (0<\delta<1), \qquad \text{(A.10)} \end{aligned}$$

where δ is such that the inequality holds. Since the integral in (A.9) does not depend on σ and is finite, this expectation is proportional to $-\sigma^{-2}$.

The above result yields

$$J(\sigma) = -\frac{1}{\sigma^2} + \frac{2}{\sigma^2} + \mathrm{E}\Big[\frac{2(y-\mu)}{\sigma^3}e^{-\frac{y-\mu}{\sigma}}\Big|\sigma\Big] - \mathrm{E}\Big[\frac{2(y-\mu)}{\sigma^3}e^{-\frac{y-\mu}{\sigma}}\Big|\sigma\Big] + \frac{1}{\sigma^2}\mathrm{const.} \propto \frac{1}{\sigma^2}$$

and

$$p(\sigma) \propto \frac{1}{\sigma}. \qquad \text{(A.11)}$$

A.3 Conditional mean estimators

In this section, we derive conditional mean estimators for the EVD parameters based on the conditional distribution (A.2).

A.3.1 Location parameter We assume a normal distribution for the location parameter μ estimated from the distribution of alignment scores for profiles described by either set of attributes, D^{A} or D^{B}. Thus, $\mu|\theta_\mu^{\mathrm{A}} \sim \mathcal{N}(m^{\mathrm{A}}, (s^{\mathrm{A}})^2)$, where $\theta_\mu^{\mathrm{A}} \equiv \{m^{\mathrm{A}}, (s^{\mathrm{A}})^2\}$ represents mean m^{A} and variance $(s^{\mathrm{A}})^2$. A corresponding expression follows for $\mu|\theta_\mu^{\mathrm{B}}$.

From (A.2) and (A.4), the conditional distribution of μ is proportional to the product of the normal distributions $p(\mu|\theta_\mu^{\mathrm{A}})$ and $p(\mu|\theta_\mu^{\mathrm{B}})$, which is a normal distribution. To see this, it suffices to show that the sum of the exponents expresses in canonical form:

$$\frac{(\mu-m^{\mathrm{A}})^2}{(s^{\mathrm{A}})^2} + \frac{(\mu-m^{\mathrm{B}})^2}{(s^{\mathrm{B}})^2} = \left(\frac{(s^{\mathrm{A}})^2+(s^{\mathrm{B}})^2}{(s^{\mathrm{B}})^2(s^{\mathrm{A}})^2}\right)\left(\mu - \frac{(s^{\mathrm{B}})^2m^{\mathrm{A}}+(s^{\mathrm{A}})^2m^{\mathrm{B}}}{(s^{\mathrm{A}})^2+(s^{\mathrm{B}})^2}\right)^2 + \frac{(m^{\mathrm{A}}-m^{\mathrm{B}})^2}{(s^{\mathrm{A}})^2+(s^{\mathrm{B}})^2}.$$

Hence,

$$\mu|\theta_\mu^{\mathrm{A}},\theta_\mu^{\mathrm{B}} \sim \mathcal{N}\left(\frac{(s^{\mathrm{B}})^2m^{\mathrm{A}}+(s^{\mathrm{A}})^2m^{\mathrm{B}}}{(s^{\mathrm{A}})^2+(s^{\mathrm{B}})^2}, \frac{(s^{\mathrm{B}})^2(s^{\mathrm{A}})^2}{(s^{\mathrm{A}})^2+(s^{\mathrm{B}})^2}\right) \qquad \text{(A.12)}$$

and the conditional mean estimator

$$\hat{\mu} = a\hat{\mu}^{\mathrm{A}} + (1-a)\hat{\mu}^{\mathrm{B}} \quad (0<a<1), \qquad \text{(A.13)}$$

where $\hat{\mu}^{\mathrm{A}} \equiv m^{\mathrm{A}}$, $\hat{\mu}^{\mathrm{B}} \equiv m^{\mathrm{B}}$, and $a = (s^{\mathrm{B}})^2/\{(s^{\mathrm{A}})^2+(s^{\mathrm{B}})^2\}$.

A.3.2 Scale parameter We assume a gamma distribution for the scale parameter σ being estimated from the distribution of alignment scores for profiles described by either set of attributes, D^{A} or D^{B}: $\sigma|\theta_\sigma^{\mathrm{A}} \sim \mathrm{Gamma}(k^{\mathrm{A}}, q^{\mathrm{A}})$ with shape k^{A} and scale q^{A} and correspondingly, $\sigma|\theta_\sigma^{\mathrm{B}} \sim \mathrm{Gamma}(k^{\mathrm{B}}, q^{\mathrm{B}})$.

Substituting (A.11) and these gamma distributions into (A.2) gives a gamma distribution for σ conditional on $\theta_\sigma^{\mathrm{A}}$ and $\theta_\sigma^{\mathrm{B}}$:

$$\sigma|\theta_\sigma^{\mathrm{A}}, \theta_\sigma^{\mathrm{B}} \sim \mathrm{Gamma}\left(k^{\mathrm{A}} + k^{\mathrm{B}}, \frac{q^{\mathrm{B}} q^{\mathrm{A}}}{q^{\mathrm{A}} + q^{\mathrm{B}}}\right). \tag{A.14}$$

Let $\hat{\sigma}^{\mathrm{A}} = k^{\mathrm{A}} q^{\mathrm{A}}$ and $\hat{\sigma}^{\mathrm{B}} = k^{\mathrm{B}} q^{\mathrm{B}}$ denote the conditional means of σ given $\theta_\sigma^{\mathrm{A}}$ and of σ given $\theta_\sigma^{\mathrm{B}}$, respectively. Then, the conditional mean estimator of σ given $\theta_\sigma^{\mathrm{A}}$ and $\theta_\sigma^{\mathrm{B}}$ is

$$\left(\frac{\hat{\sigma}^{\mathrm{A}}}{q^{\mathrm{A}}} + \frac{\hat{\sigma}^{\mathrm{B}}}{q^{\mathrm{B}}}\right) \frac{q^{\mathrm{B}} q^{\mathrm{A}}}{q^{\mathrm{A}} + q^{\mathrm{B}}}$$

or

$$\hat{\sigma} = b\hat{\sigma}^{\mathrm{A}} + (1 - b)\hat{\sigma}^{\mathrm{B}} \quad (0 < b < 1), \tag{A.15}$$

where $b = q^{\mathrm{B}}/(q^{\mathrm{A}} + q^{\mathrm{B}})$.

References

Altschul, S. (1993). A protein alignment scoring system sensitive at all evolutionary distances. *J Mol Evol*, **36**(3), 290–300.

Altschul, S. and Gish, W. (1996). Local alignment statistics. *Methods Enzymol*, **266**, 460–480.

Altschul, S., Bundschuh, R., Olsen, R., and Hwa, T. (2001). The estimation of statistical parameters for local alignment score distributions. *Nucleic Acids Res*, **29**(2), 351–361.

Altschul, S., Wootton, J., Gertz, E., Agarwala, R., Morgulis, A., Schäffer, A., and Yu, Y. (2005). Protein database searches using compositionally adjusted substitution matrices. *FEBS J*, **272**(20), 5101–5109.

Altschul, S. F., Madden, T. L., Schäffer, A. A., Zhang, J., Zhang, Z., Miller, W., and Lipman, D. J. (1997). Gapped BLAST and PSI-BLAST: a new generation of protein database search programs. *Nucleic Acids Res*, **25**(17), 3389–3402.

Bailey, T. and Gribskov, M. (2002). Estimating and evaluating the statistics of gapped local-alignment scores. *J Comput Biol*, **9**(3), 575–593.

Bishop, C. (2006). *Pattern recognition and machine learning*. Springer, New York.

Bundschuh, R. (2000). An analytic approach to significance assessment in local sequence alignment with gaps. In R. Shamir, S. Miyano, S. Istrail, P. Pevzner, and M. Waterman, editors, *Proceedings of the fourth annual international conference on Computational molecular biology*, RECOMB '00, pages 86–95, New York. ACM.

Bundschuh, R. (2002a). Asymmetric exclusion process and extremal statistics of random sequences. *Phys Rev E Stat Nonlin Soft Matter Phys*, **65**(3), 031911.

Bundschuh, R. (2002b). Rapid significance estimation in local sequence alignment with gaps. *J Comput Biol*, **9**(2), 243–260.

Chernobai, A., Rachev, S., and Fabozzi, F. (2015). Composite goodness-of-fit tests for left-truncated loss samples. In C. Lee and J. Lee, editors, *Handbook of Financial Econometrics and Statistics*, pages 575–596. Springer, New York.

Chia, N. and Bundschuh, R. (2006). A practical approach to significance assessment in alignment with gaps. *J Comput Biol*, **13**(2), 429–441.

Eddy, S. (2008). A probabilistic model of local sequence alignment that simplifies statistical significance estimation. *PLoS Comput Biol*, **4**(5), e1000069.

Eddy, S. (2011). Accelerated profile HMM searches. *PLoS Comput Biol*, **7**(10), e1002195.

Finn, R., Coggill, P., Eberhardt, R., Eddy, S., Mistry, J., Mitchell, A., *et al.* (2016). The Pfam protein families database: towards a more sustainable future. *Nucleic Acids Res*, **44**(D1), D279–D285.

Fox, N., Brenner, S., and Chandonia, J. (2013). SCOPe: Structural classification of proteins–extended, integrating SCOP and ASTRAL data and classification of new structures. *Nucleic Acids Res*, **42**(D1), D304–D309.

Gelman, A., Carlin, J., Stern, H., and Rubin, D. (2004). *Bayesian Data Analysis*. Chapman & Hall/CRC, Boca Raton, 2nd edition.

Goldstein, L. and Waterman, M. (1994). Approximations to profile score distributions. *J Comput Biol*, **1**(2), 93–104.

Hanley, J. A. and McNeil, B. J. (1983). A method of comparing the areas under receiver operating characteristic curves derived from the same cases. *Radiology*, **148**(3), 839–843.

Hartmann, A. (2002). Sampling rare events: Statistics of local sequence alignments. *Phys Rev E Stat Nonlin Soft Matter Phys*, **65**(5), 056102.

Holm, L., Kääriäinen, S., Rosenström, P., and Schenkel, A. (2008). Searching protein structure databases with DaliLite v.3. *Bioinformatics*, **24**(23), 2780–2781.

Jaroszewski, L., Li, Z., Cai, X. H., Weber, C., and Godzik, A. (2011). FFAS server: novel features and applications. *Nucleic Acids Res*, **39**, W38–W44.

Jones, D. T. (1999). Protein secondary structure prediction based on position-specific scoring matrices. *J Mol Biol*, **292**(2), 195–202.

Karlin, S. (1994). Statistical studies of biomolecular sequences: Score-based methods. *Philos Trans R Soc Lond B Biol Sci*, **344**(1310), 391–402.

Karlin, S. (2005). Statistical signals in bioinformatics. *Proc Natl Acad Sci USA*, **102**(38), 13355–13362.

Karlin, S. and Altschul, S. (1990). Methods for assessing the statistical significance of molecular sequence features by using general scoring schemes. *Proc Natl Acad Sci USA*, **87**(6), 2264–2268.

Karlin, S. and Altschul, S. (1993). Applications and statistics for multiple high-scoring segments in molecular sequences. *Proc Natl Acad Sci USA*, **90**(12), 5873–5877.

Kleiner, A., Talwalkar, A., Sarkar, P., and Jordan, M. (2014). A scalable bootstrap for massive data. *J R Stat Soc Series B Stat Methodol*, **76**(4), 795–816.

Kotz, S. and Nadarajah, S. (2000). *Extreme value distributions: theory and applications*. Imperial College Press, London.

Liang, F., Kim, J., and Song, Q. (2016). A bootstrap Metropolis-Hastings algorithm for Bayesian analysis of big data. *Technometrics*, **58**(3), 304–318.

Margelevičius, M. (2016). Bayesian nonparametrics in protein remote homology search. *Bioinformatics*, **32**(18), 2744–2752.

Margelevičius, M. (2018). A low-complexity add-on score for protein remote homology search with COMER. *Bioinformatics*, **34**(12), 2037–2045.

Margelevičius, M. and Venclovas, Č. (2010). Detection of distant evolutionary relationships between protein families using theory of sequence profile-profile comparison. *BMC Bioinformatics*, **11**, 89.

Meng, L., Sun, F., Zhang, X., and Waterman, M. (2011). Sequence alignment as hypothesis testing. *J Comput Biol*, **18**(5), 677–691.

Metzler, D. (2006). Robust E-values for gapped local alignments. *J Comput Biol*, **13**(4), 882–896.

Mott, R. (1992). Maximum-likelihood estimation of the statistical distribution of smith-waterman local sequence similarity scores. *Bull Math Biol*, **54**(1), 59–75.

Mott, R. (2000). Accurate formula for P-values of gapped local sequence and profile alignments. *J Mol Biol*, **300**(3), 649–659.

Mott, R. and Tribe, R. (1999). Approximate statistics of gapped alignments. *J Comput Biol*, **6**(1), 91–112.

Neuhauser, C. (1994). A Poisson approximation for sequence comparisons with insertions and deletions. *Ann Stat*, **22**(3), 1603–1629.

Newberg, L. (2008). Significance of gapped sequence alignments. *J Comput Biol*, **15**(9), 1187–1194.

Nissen, S. (2012). *FANN: Fast Artificial Neural Network Library*.

Olsen, R., Bundschuh, R., and Hwa, T. (1999). Rapid assessment of extremal statistics for gapped local alignment. In T. Lengauer, R. Schneider, P. Bork, D. Brutlag, J. Glasgow, H.-W. Mewes, and R. Zimmer, editors, *Proceedings of the Seventh International Conference on Intelligent Systems for Molecular Biology*, ISMB '99, pages 211–222, Menlo Park. AAAI Press.

Pearson, W. (1998). Empirical statistical estimates for sequence similarity searches. *J Mol Biol*, **276**(1), 71–84.

Pei, J. and Grishin, N. (2001). AL2CO: calculation of positional conservation in a protein sequence alignment. *Bioinformatics*, **17**(8), 700–712.

Poleksic, A. (2009). Island method for estimating the statistical significance of profile-profile alignment scores. *BMC Bioinformatics*, **10**, 112.

Poleksic, A., Danzer, J., Hambly, K., and Debe, D. (2005). Convergent island statistics: a fast method for determining local alignment score significance. *Bioinformatics*, **21**(12), 2827–2831.

Poole, W., Gibbs, D., Shmulevich, I., Bernard, B., and Knijnenburg, T. (2016). Combining dependent p-values with an empirical adaptation of Brown's method. *Bioinformatics*, **32**(17), i430–i436.

Remmert, M., Biegert, A., Hauser, A., and Söding, J. (2012). HHblits: lightning-fast iterative protein sequence searching by HMM-HMM alignment. *Nat Methods*, **9**(2), 173–175.

Robin, X., Turck, N., Hainard, A., Tiberti, N., Lisacek, F., Sanchez, J. C., and Müller, M. (2011). pROC: an open-source package for R and S+ to analyze and compare ROC curves. *BMC Bioinformatics*, **12**, 77.

Rychlewski, L., Jaroszewski, L., Li, W., and Godzik, A. (2000). Comparison of sequence profiles. strategies for structural predictions using sequence information. *Protein Sci*, **9**(2), 232–241.

Sadreyev, R. and Grishin, N. (2003). Compass: a tool for comparison of multiple protein alignments with assessment of statistical significance. *J Mol Biol*, **326**(1), 317–336.

Sadreyev, R. and Grishin, N. (2008). Accurate statistical model of comparison between multiple sequence alignments. *Nucleic Acids Res*, **36**(7), 2240–2248.

Schäffer, A., Aravind, L., Madden, T., Shavirin, S., Spouge, J., Wolf, Y., *et al.* (2001). Improving the accuracy of PSI-BLAST protein database searches with composition-based statistics and other refinements. *Nucleic Acids Res*, **29**(14), 2994–3005.

Söding, J. (2005). Protein homology detection by HMM-HMM comparison. *Bioinformatics*, **21**(7), 951–960.

Söding, J., Meier, M., Steinegger, M., Remmert, M., Hauser, A., Angermüller, C., Meier, A., and Biegert, A. (2015). *HHsuite for sensitive protein sequence searching based on HMM-HMM alignment*.

Spang, R. and Vingron, M. (1998). Statistics of large-scale sequence searching. *Bioinformatics*, **14**(3), 279–284.

Spang, R. and Vingron, M. (2001). Limits of homology detection by pairwise sequence comparison. *Bioinformatics*, **17**(4), 338–342.

Storey, J. and Siegmund, D. (2001). Approximate p-values for local sequence alignments: numerical studies. *J Comput Biol*, **8**(5), 549–556.

Suzek, B., Wang, Y., Huang, H., McGarvey, P., Wu, C., and the UniProt Consortium (2015). UniRef clusters: a comprehensive and scalable alternative for improving sequence similarity searches. *Bioinformatics*, **31**(6), 926–932.

Šali, A. and Blundell, T. L. (1993). Comparative protein modelling by satisfaction of spatial restraints. *J Mol Biol*, **234**(3), 779–815.

Wang, H., Zhu, R., and Ma, P. (2017). Optimal subsampling for large sample logistic regression. *J Am Stat Assoc*.

Waterman, M. (1994). Estimating statistical significance of sequence alignments. *Philos Trans R Soc Lond B Biol Sci*, **344**(1310), 383–390.

Waterman, M. and Vingron, M. (1994). Rapid and accurate estimates of statistical significance for sequence data base searches. *Proc Natl Acad Sci USA*, **91**(11), 4625–4628.

Wolfsheimer, S., Burghardt, B., and Hartmann, A. (2007). Local sequence alignments statistics: deviations from gumbel statistics in the rare-event tail. *Algorithms Mol Biol*, **2**, 9.

Wolter, T. (2012). *truncgof: GoF tests allowing for left truncated data*.

Wootton, J. and Federhen, S. (1996). Analysis of compositionally biased regions in sequence databases. *Methods Enzymol*, **266**, 554–571.

Yu, Y. and Hwa, T. (2001). Statistical significance of probabilistic sequence alignment and related local hidden markov models. *J Comput Biol*, **8**(3), 249–282.

Yu, Y., Bundschuh, R., and Hwa, T. (2002). Hybrid alignment: high-performance with universal statistics. *Bioinformatics*, **18**(6), 864–872.

Yu, Y., Wootton, J., and Altschul, S. (2003). The compositional adjustment of amino acid substitution matrices. *Proc Natl Acad Sci USA*, **100**(26), 15688–15693.

Yu, Y., Gertz, E., Agarwala, R., Schäffer, A., and Altschul, S. (2006). Retrieval accuracy, statistical significance and compositional similarity in protein sequence database searches. *Nucleic Acids Res*, **34**(20), 5966–5973.

Zhang, Y. (1995). A limit theorem for matching random sequences allowing deletions. *Ann Appl Probab*, **5**(4), 1236–1240.

Zhang, Y. and Skolnick, J. (2004). Scoring function for automated assessment of protein structure template quality. *Proteins*, **57**(4), 702–710.
