# Supplementary figures and images for: Estimating statistical significance of local protein profile-profile alignments
Source: BMC Bioinformatics. 2019 Aug 13;20:419. doi: 10.1186/s12859-019-2913-3 (PMC6693267; doi:10.1186/s12859-019-2913-3)

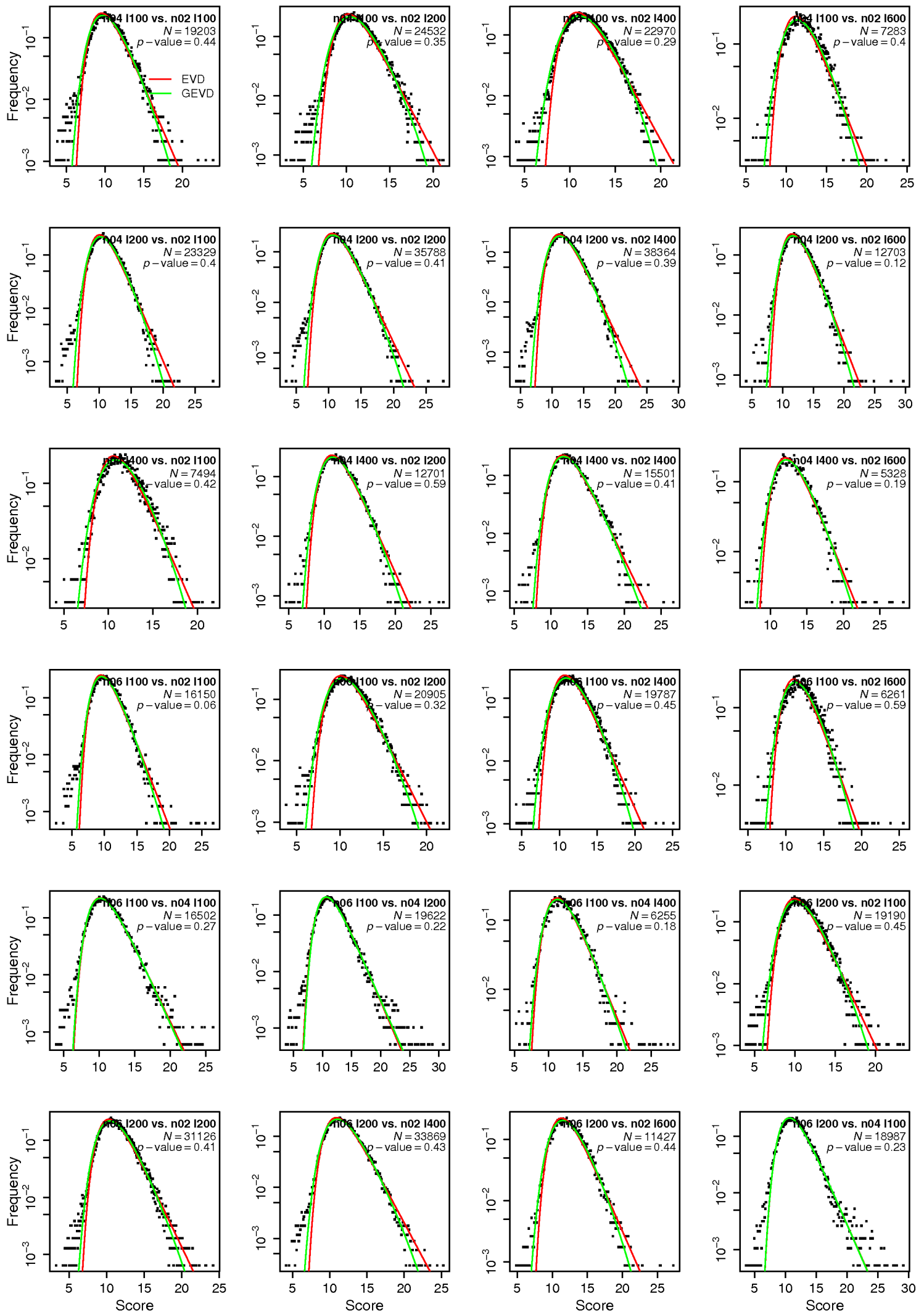

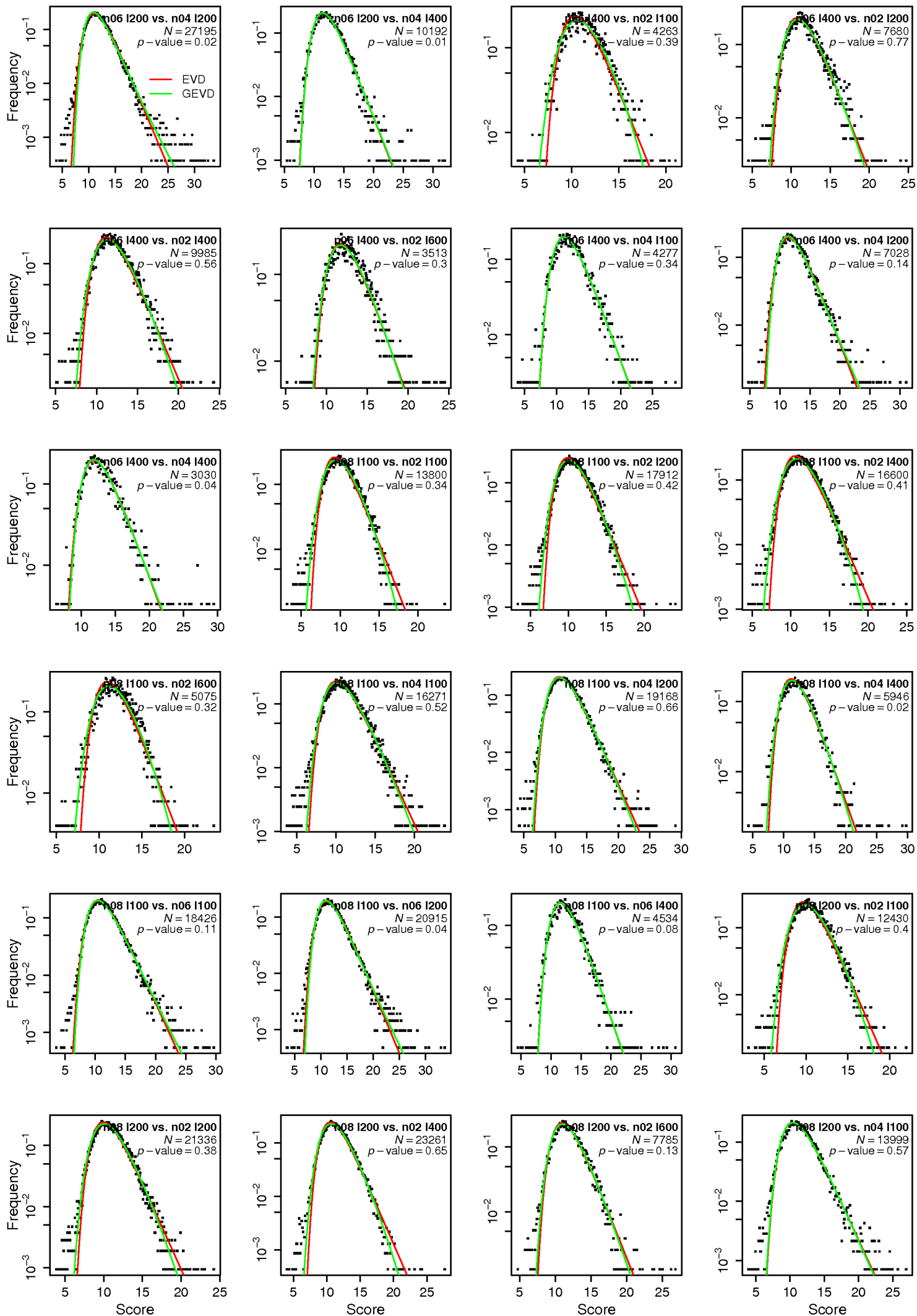

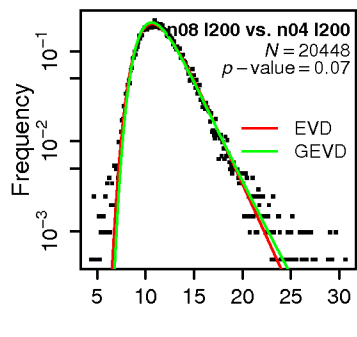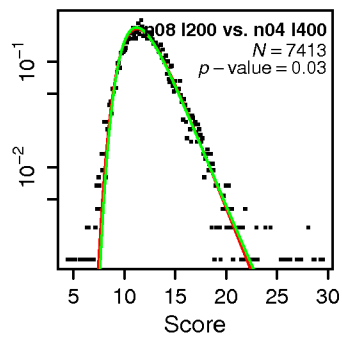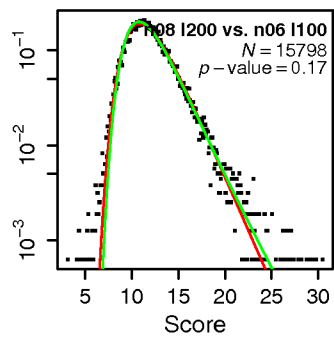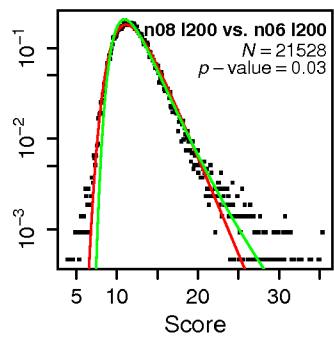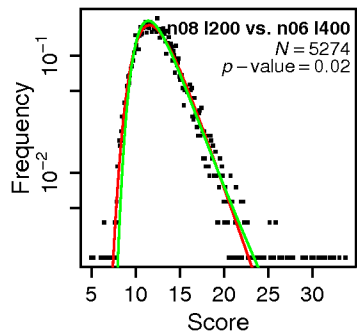

Supplement: Supplementary file 2 — Figure S1. Distributions of alignment scores of real unrelated profiles for different pair values of profile ENO and length. (PDF 579 kb) [file 12859_2019_2913_MOESM2_ESM.pdf]

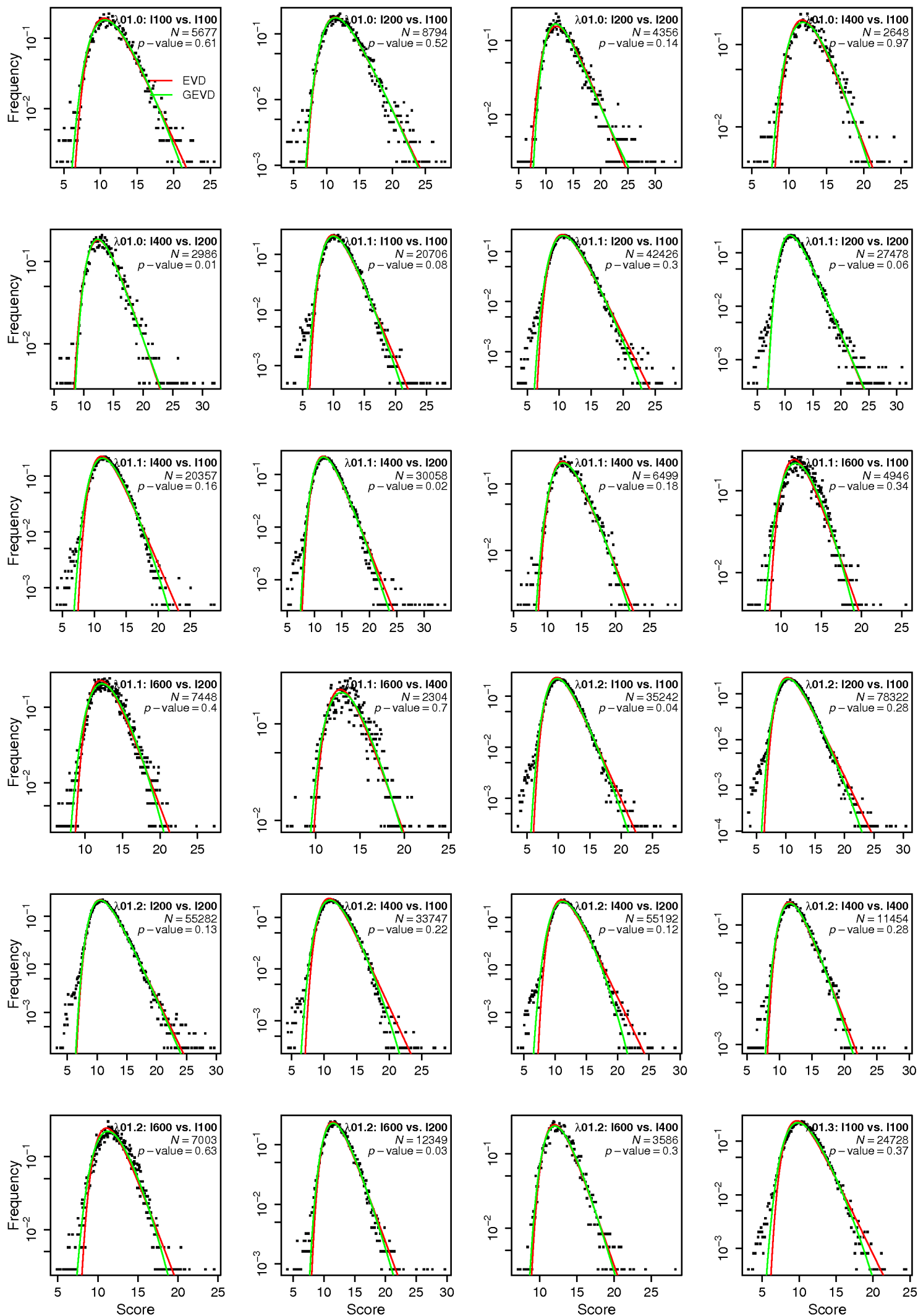

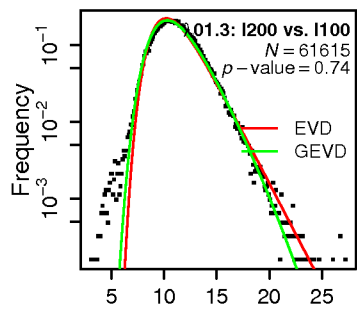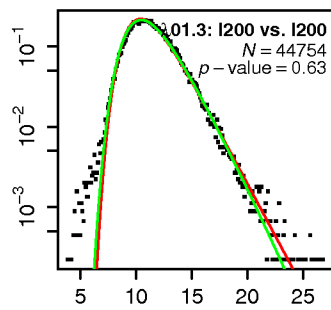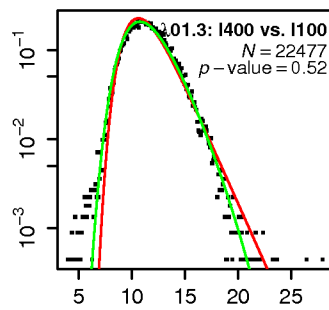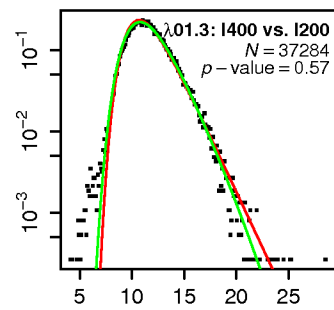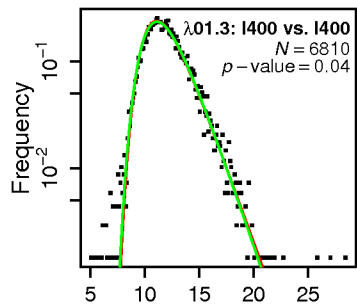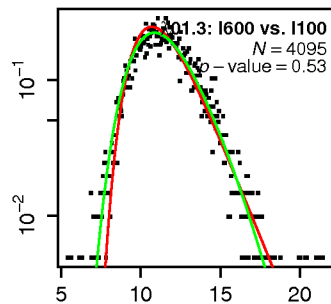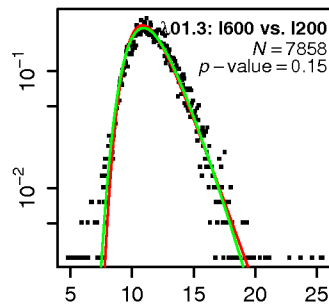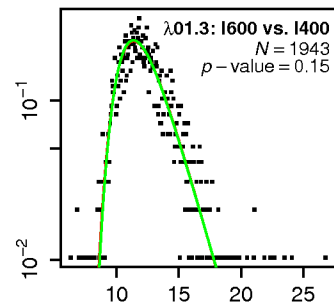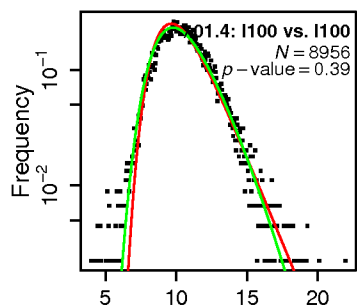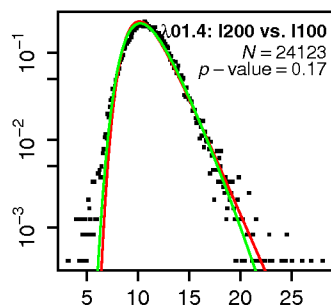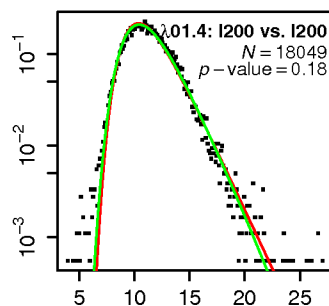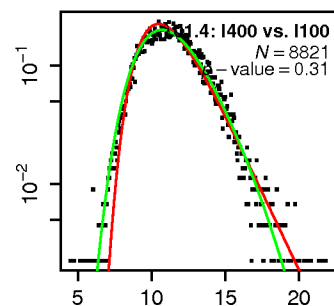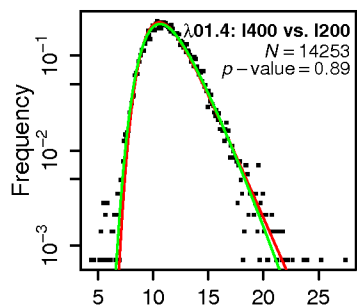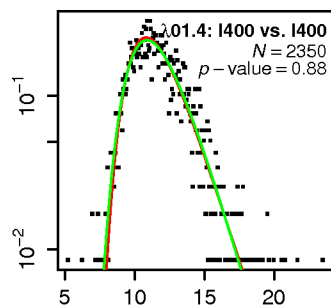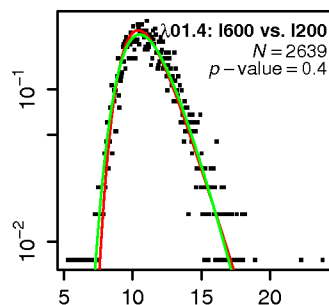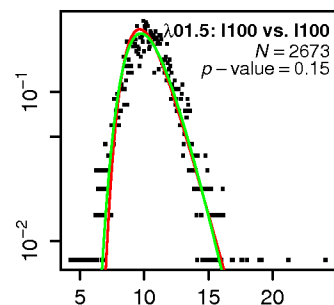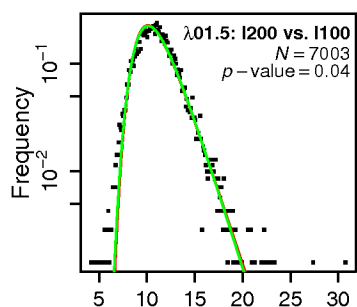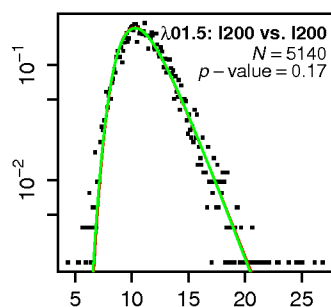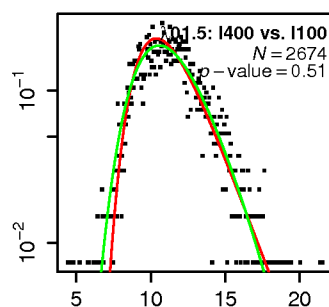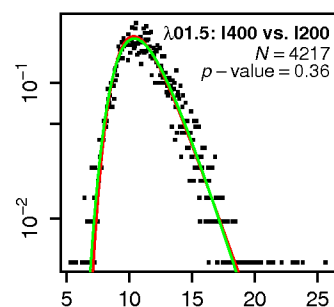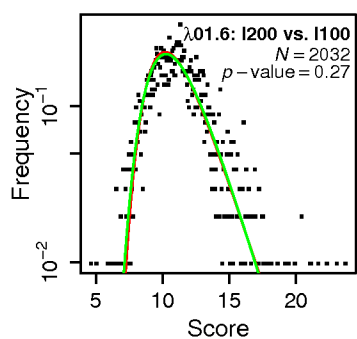

Supplement: Supplementary file 3 — Figure S3. Distributions of alignment scores obtained from aligning pairs of real profiles with mutual compositional similarity λ and different values of length l. (PDF 467 kb) [file 12859_2019_2913_MOESM3_ESM.pdf]

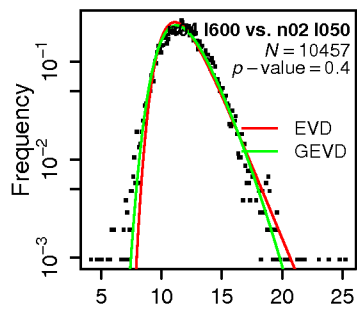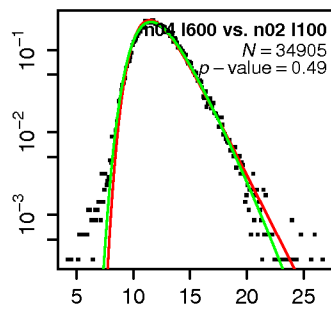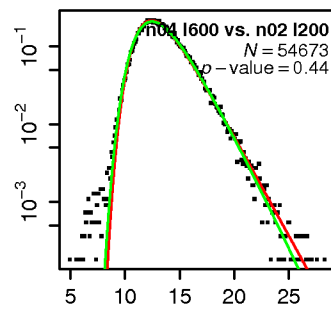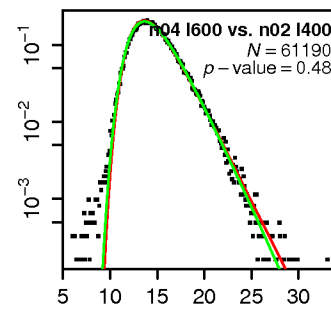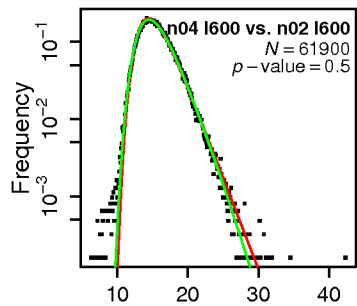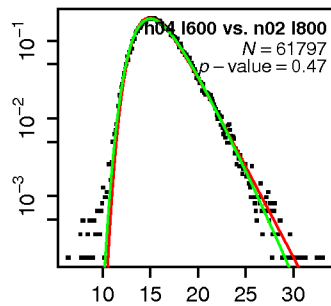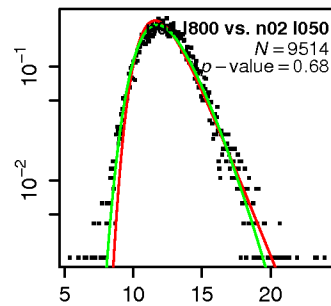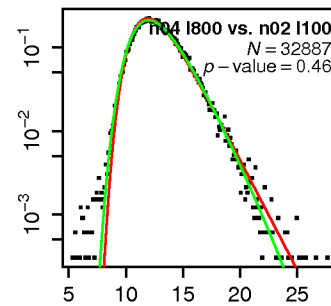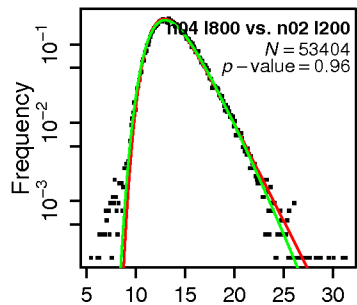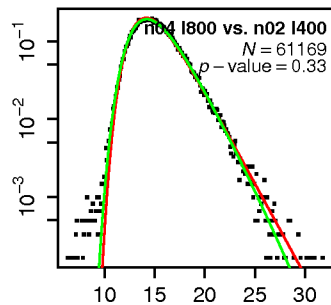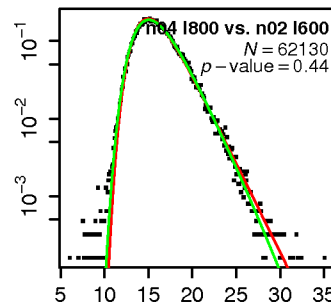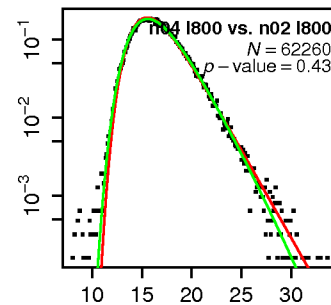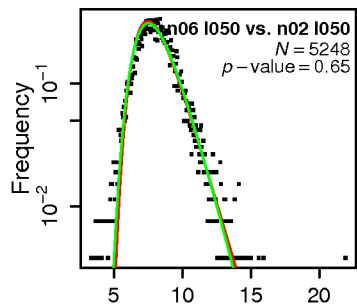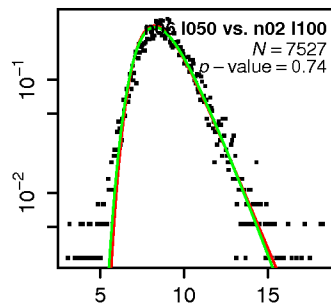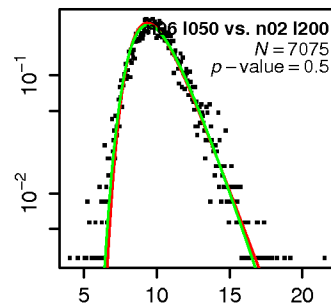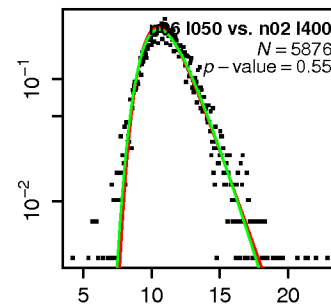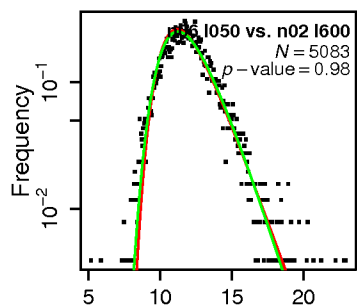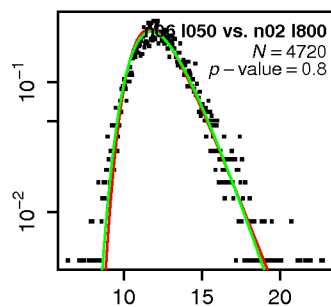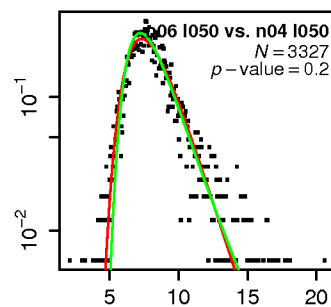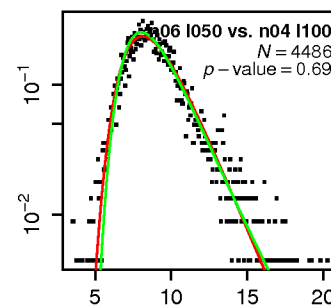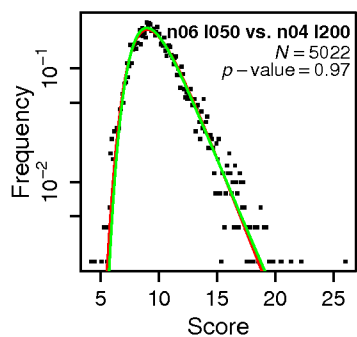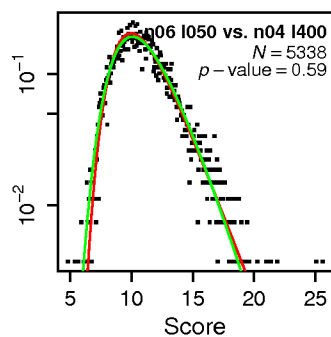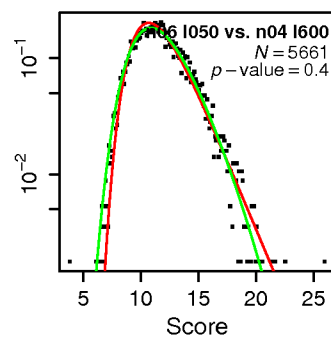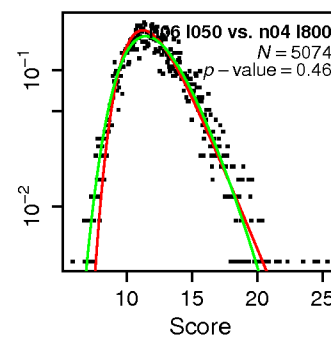

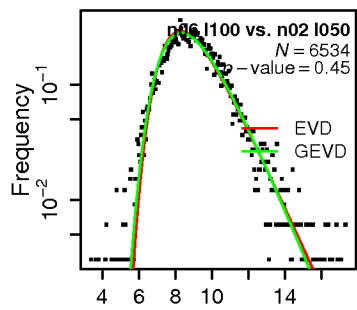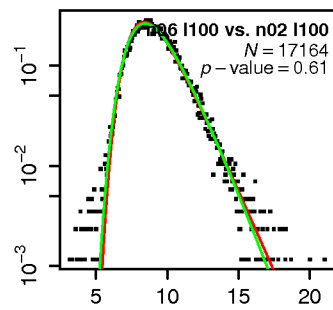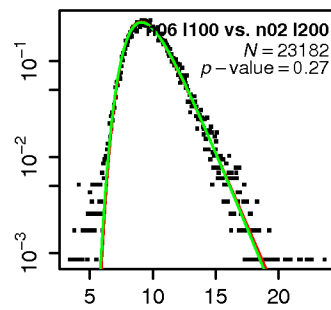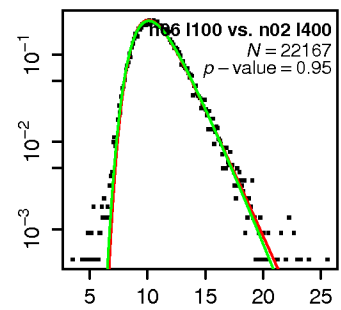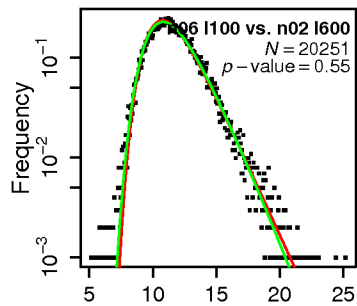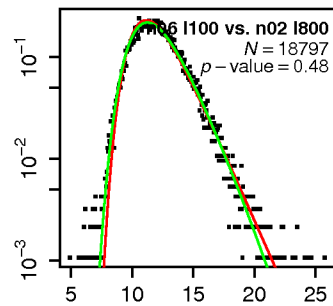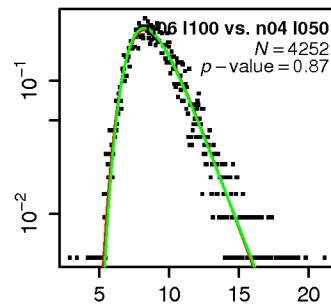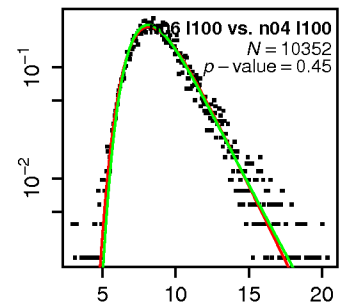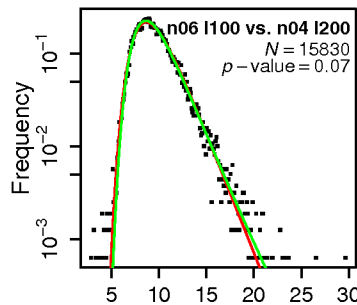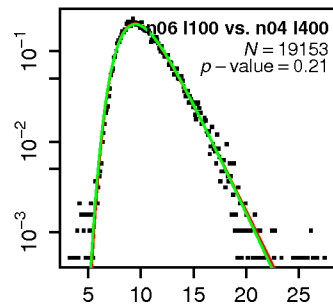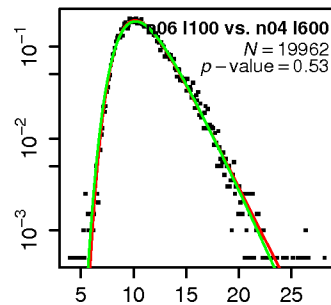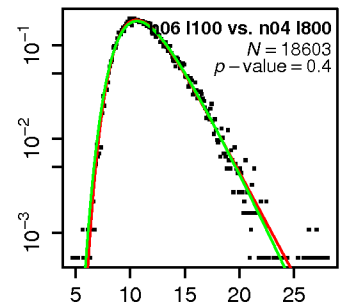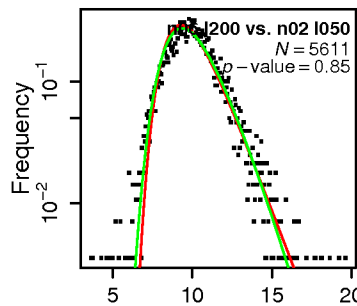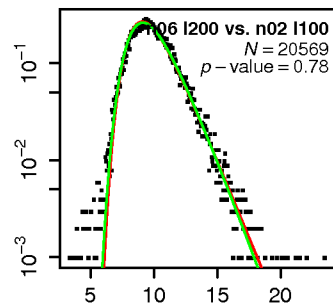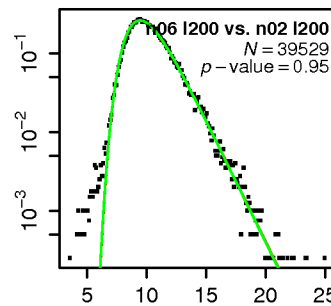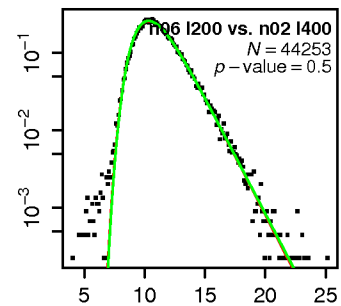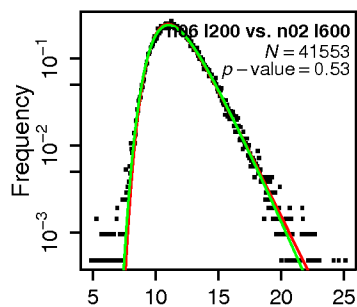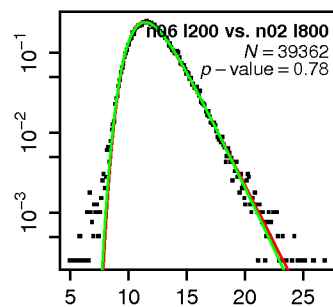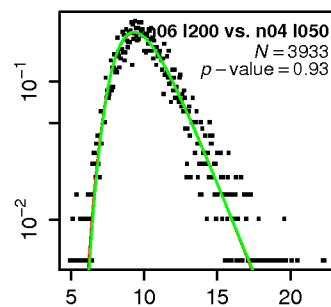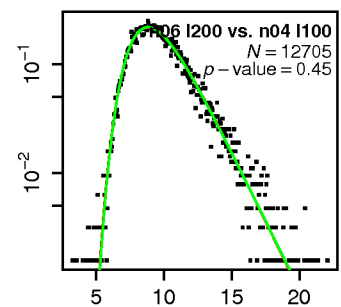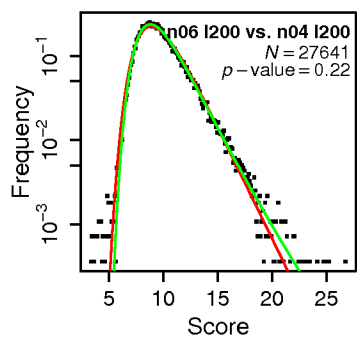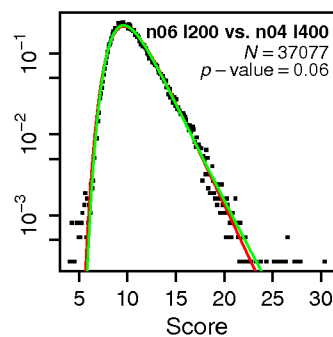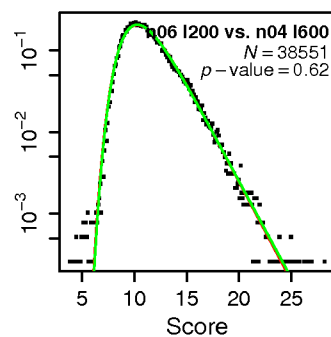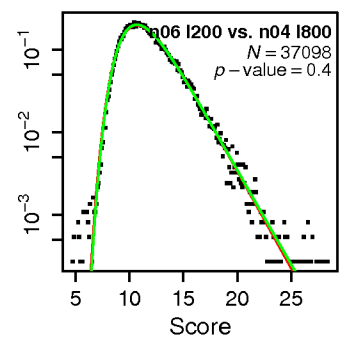

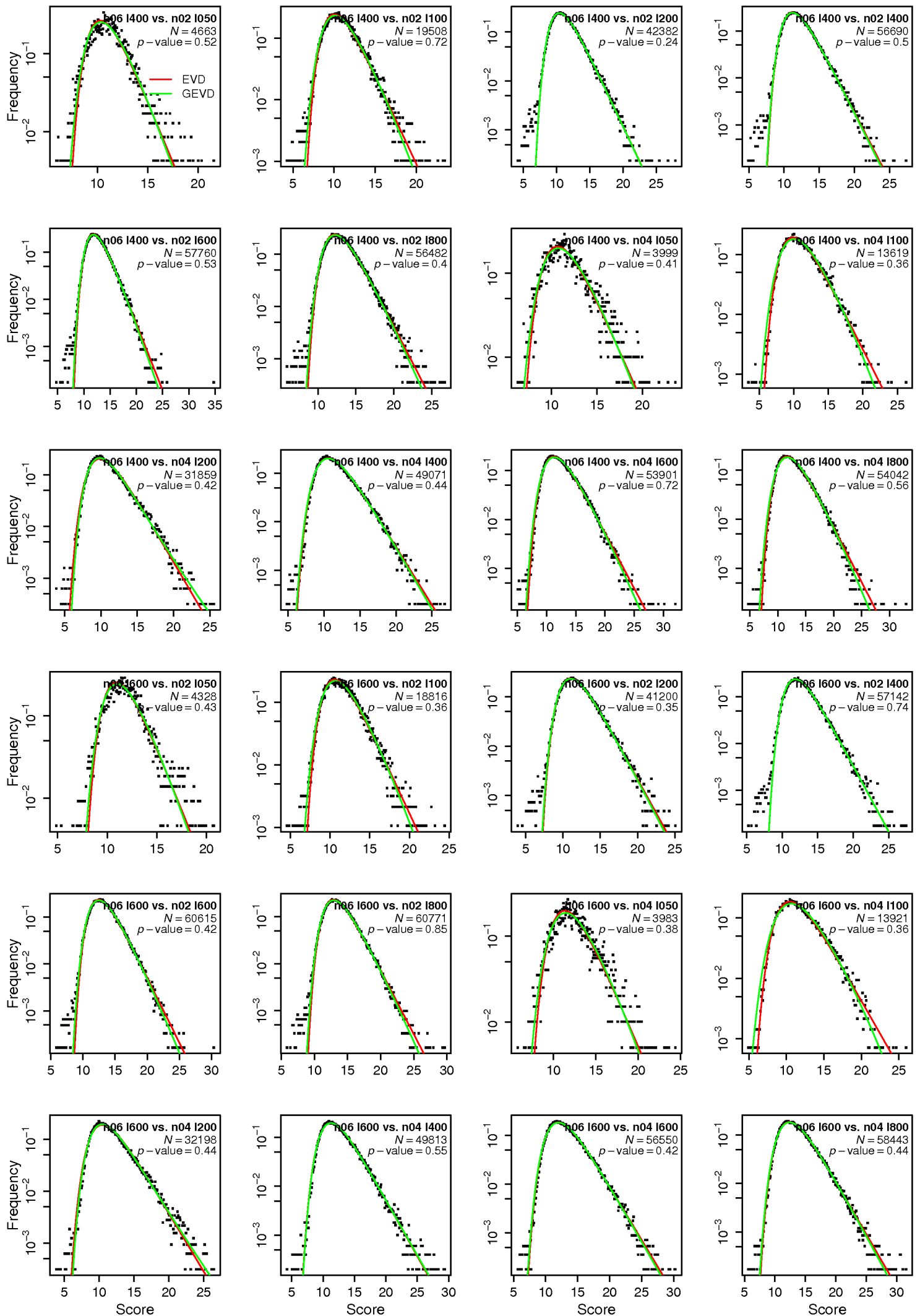



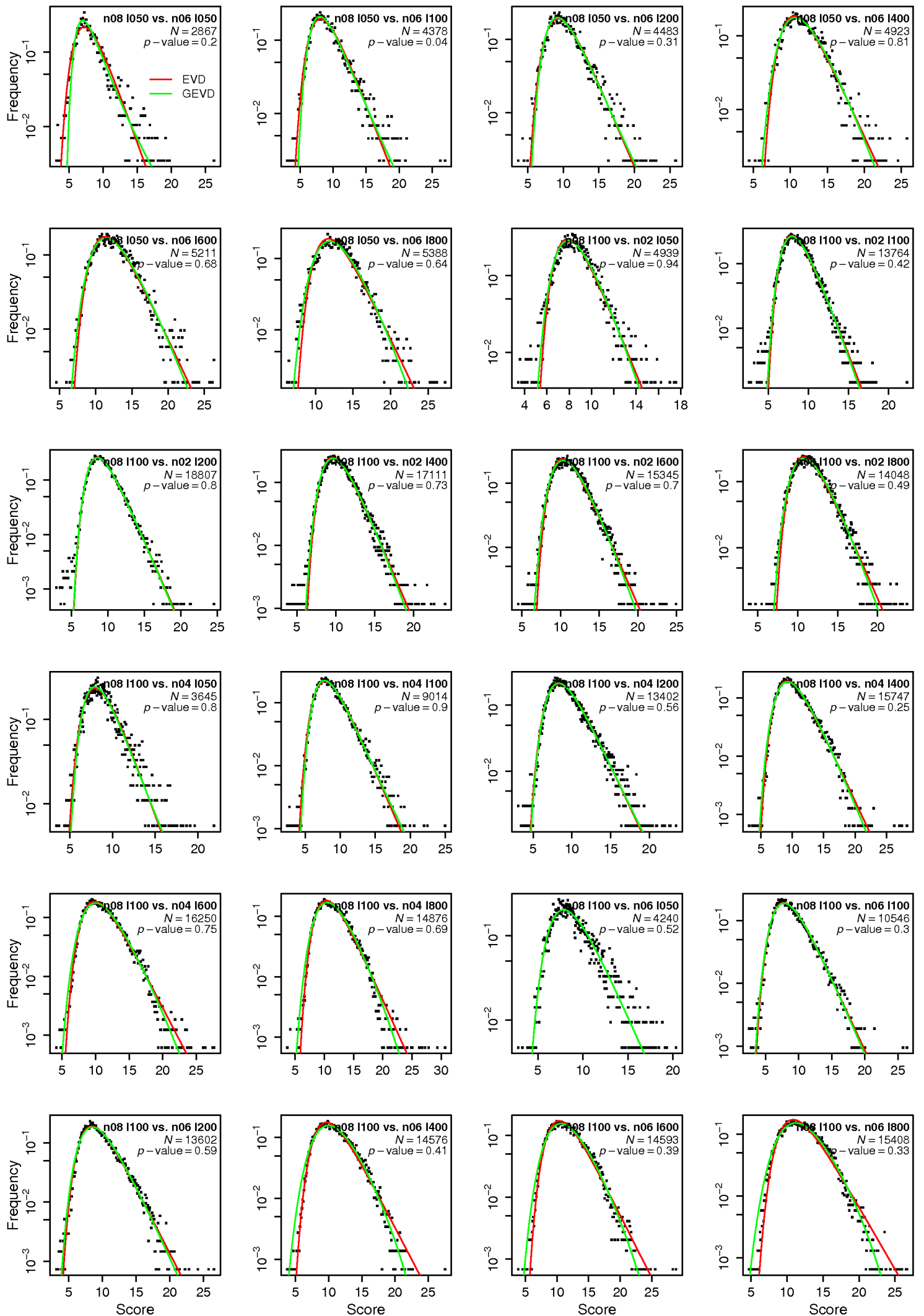

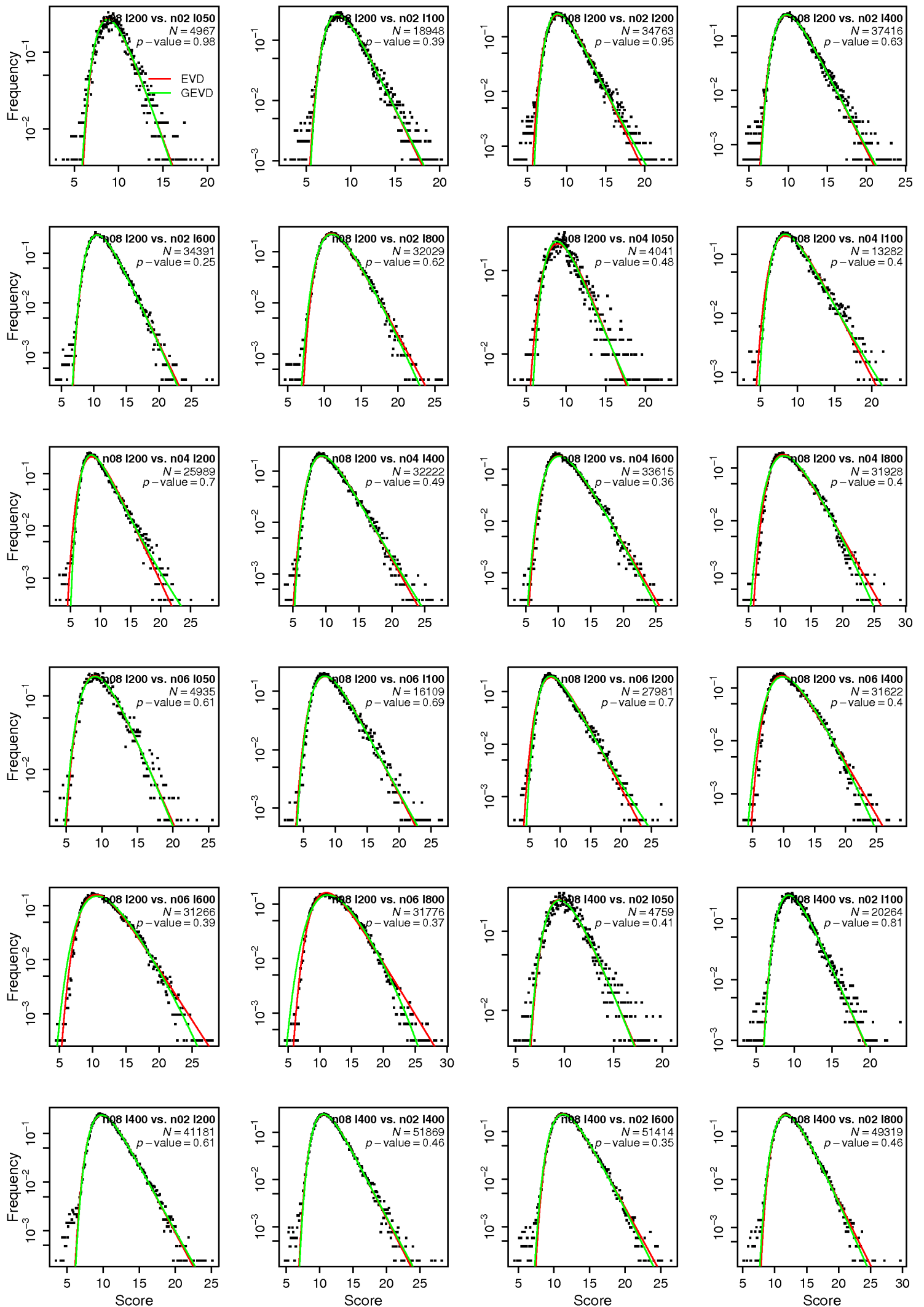

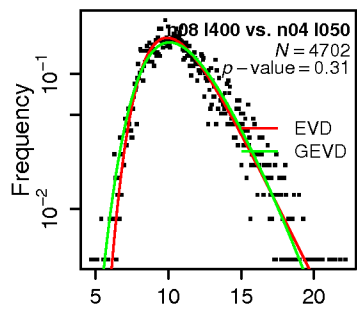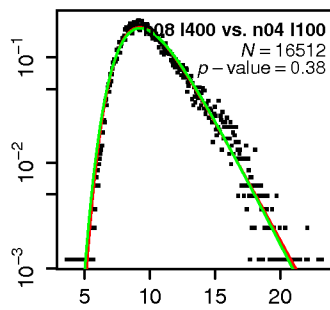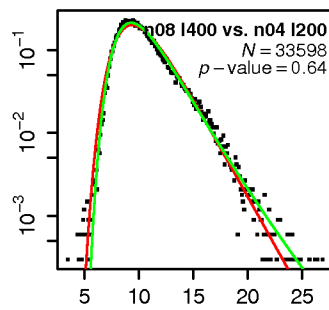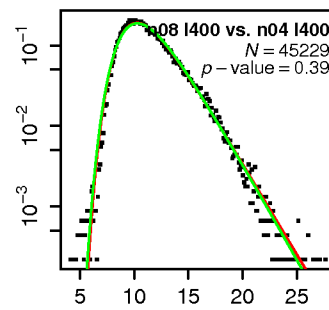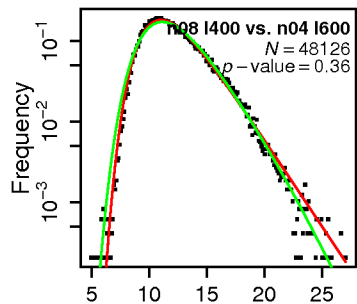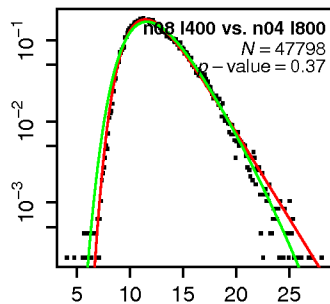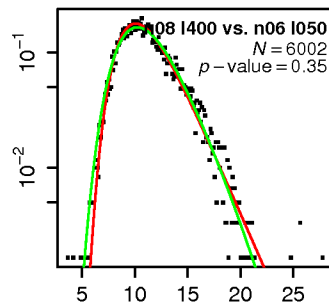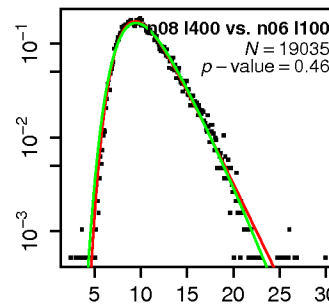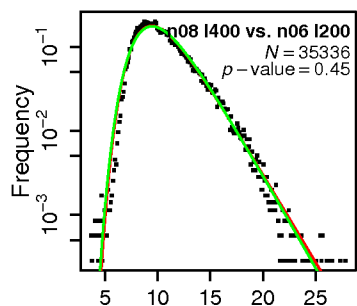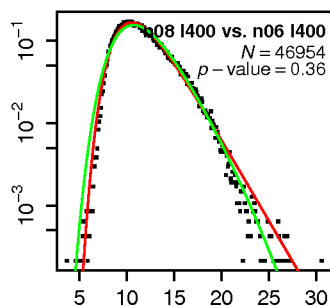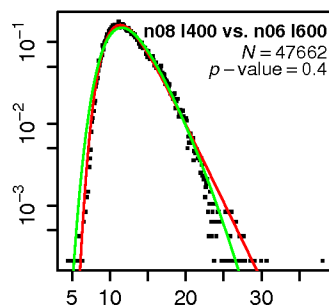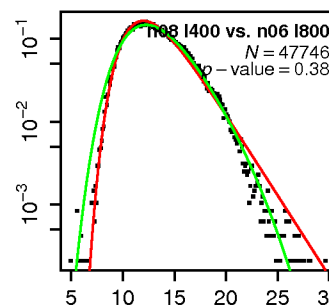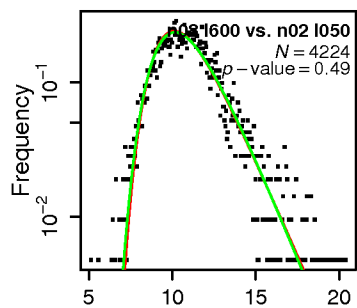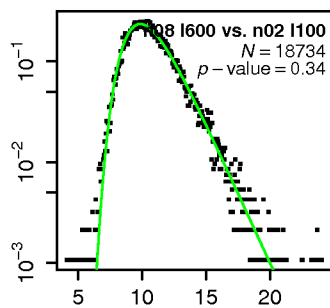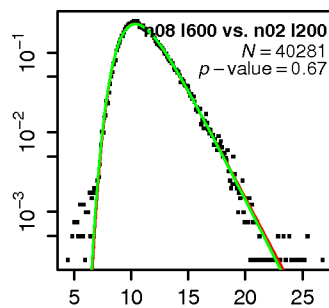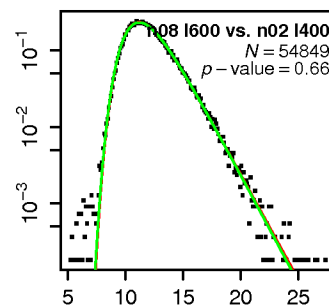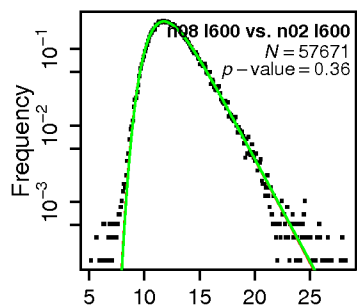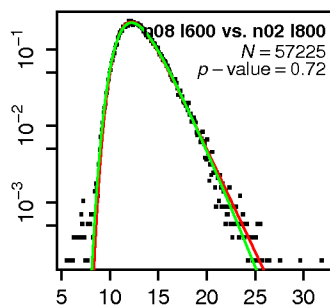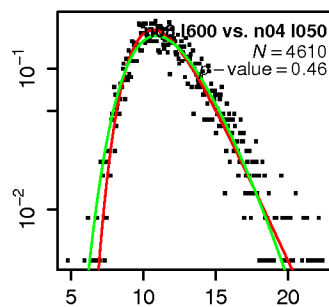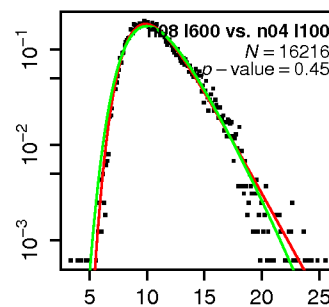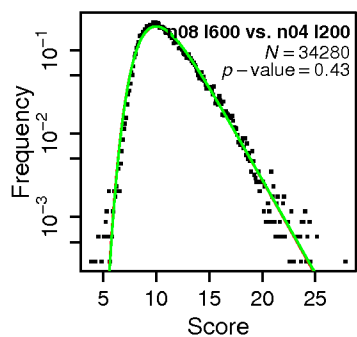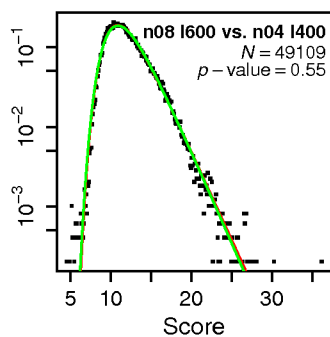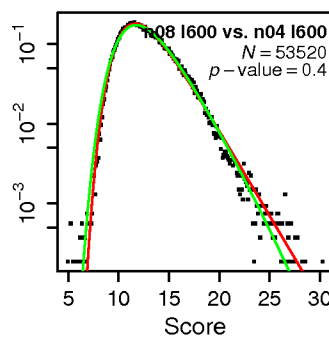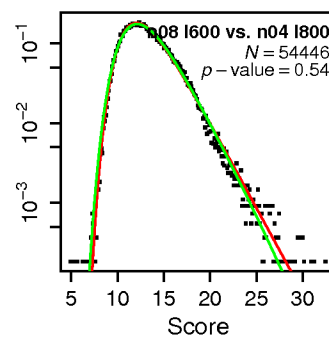

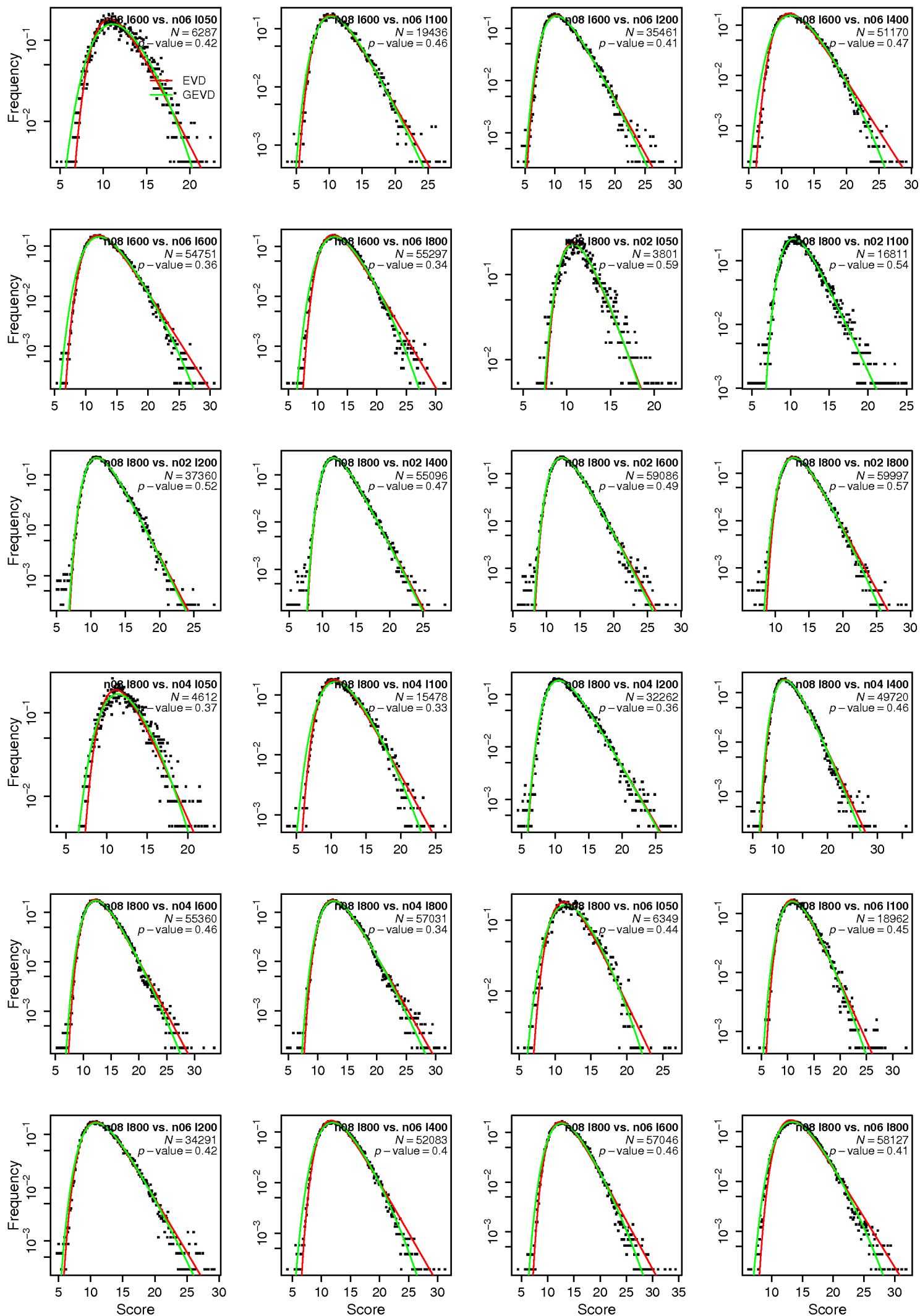



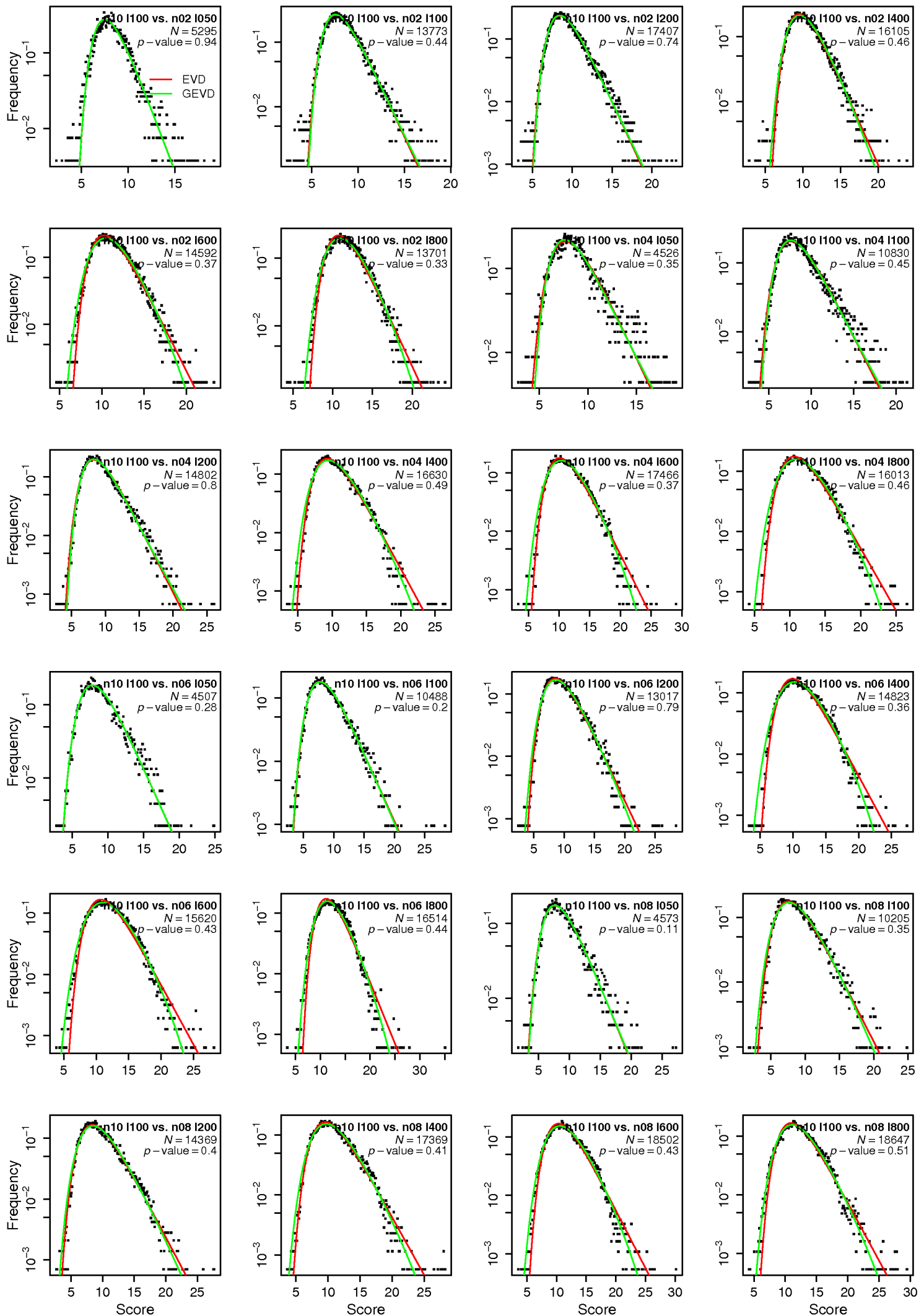

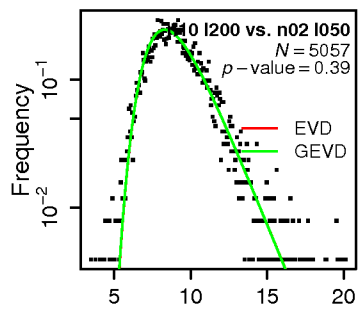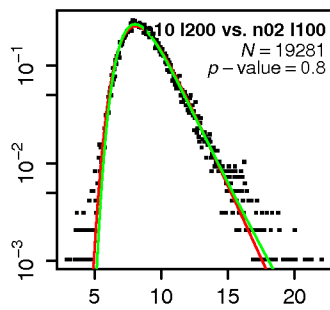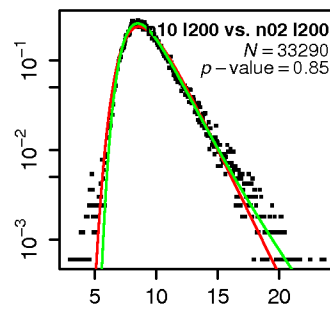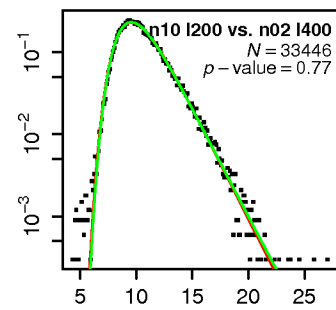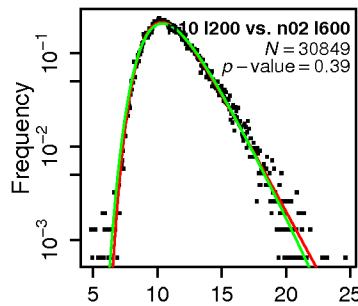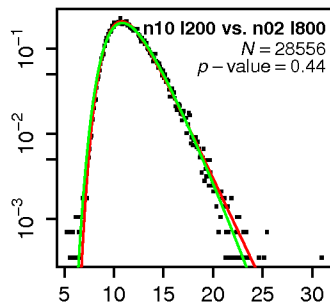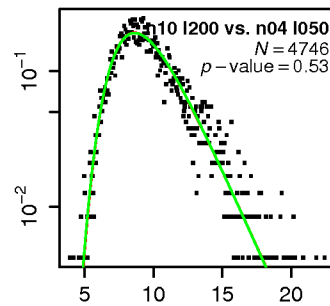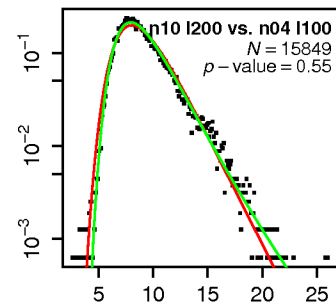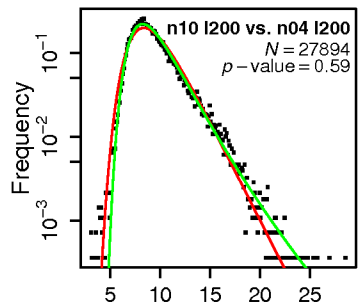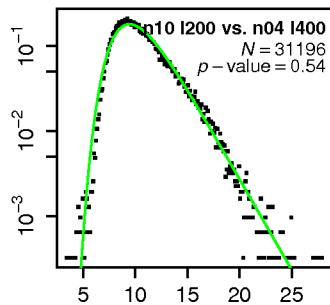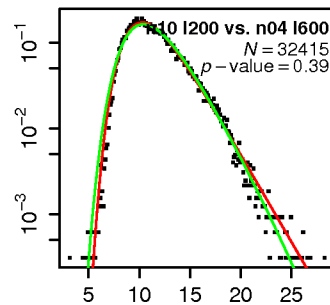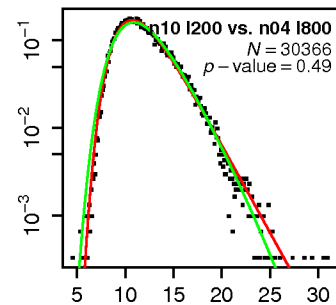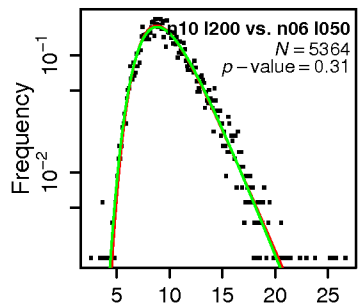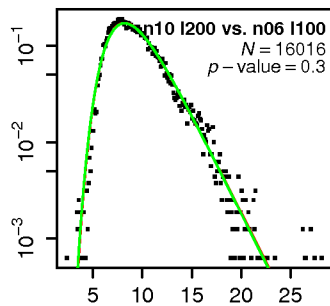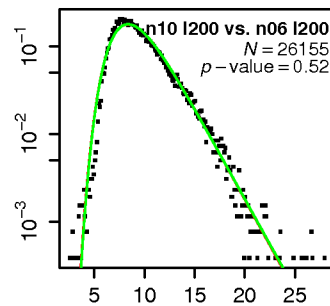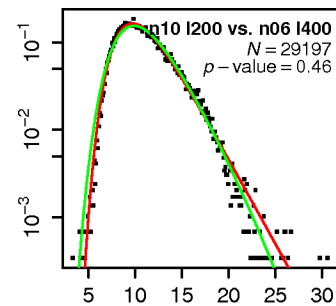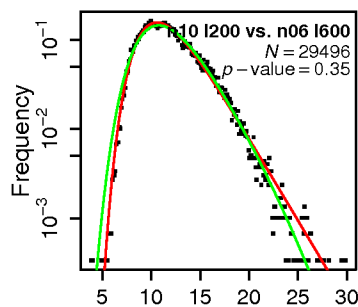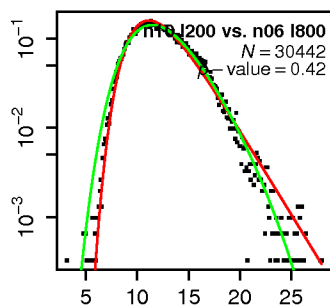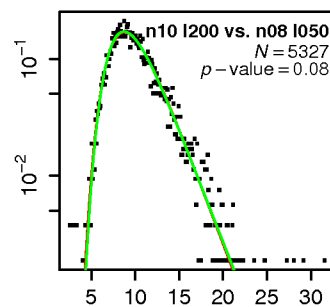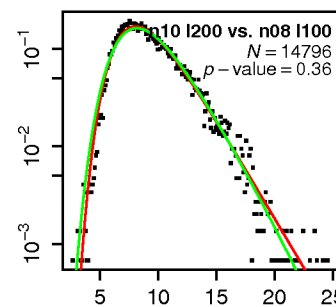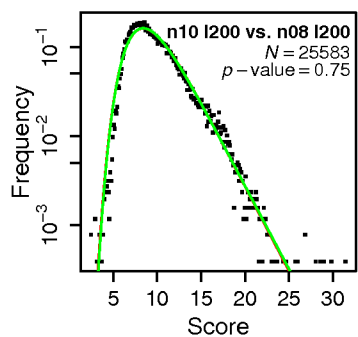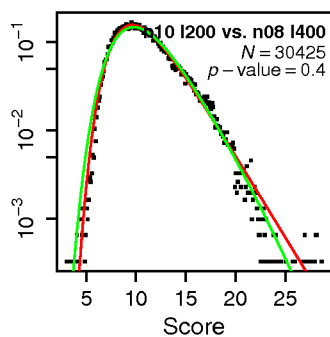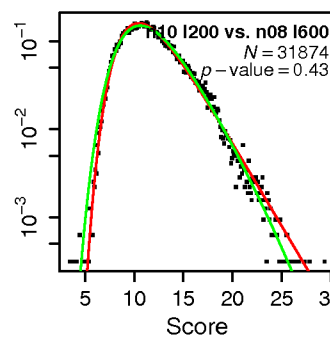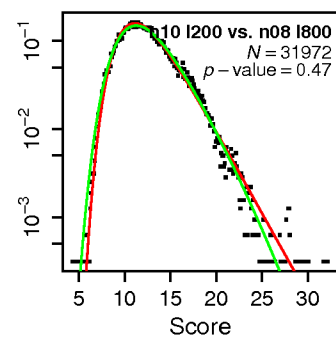

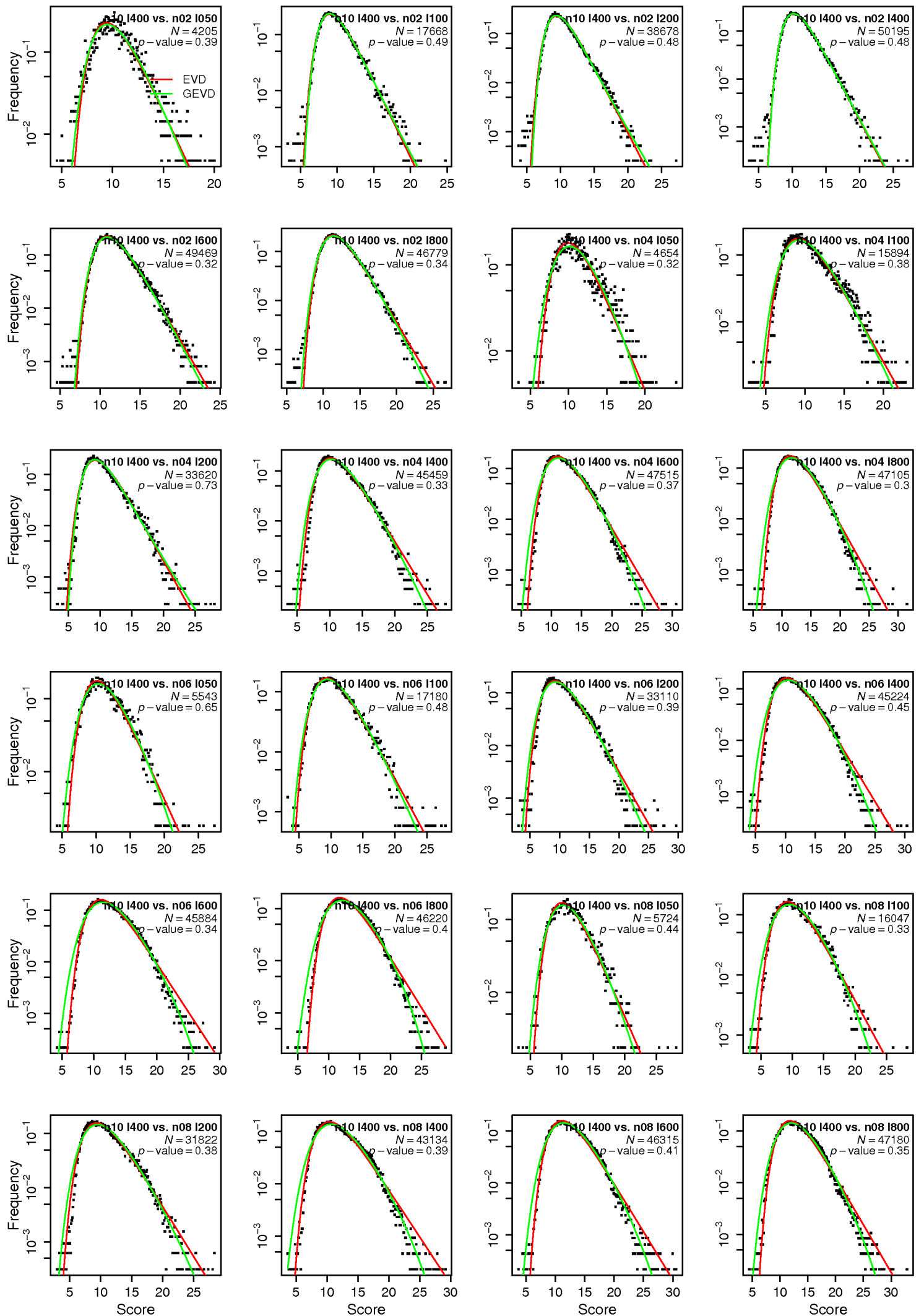

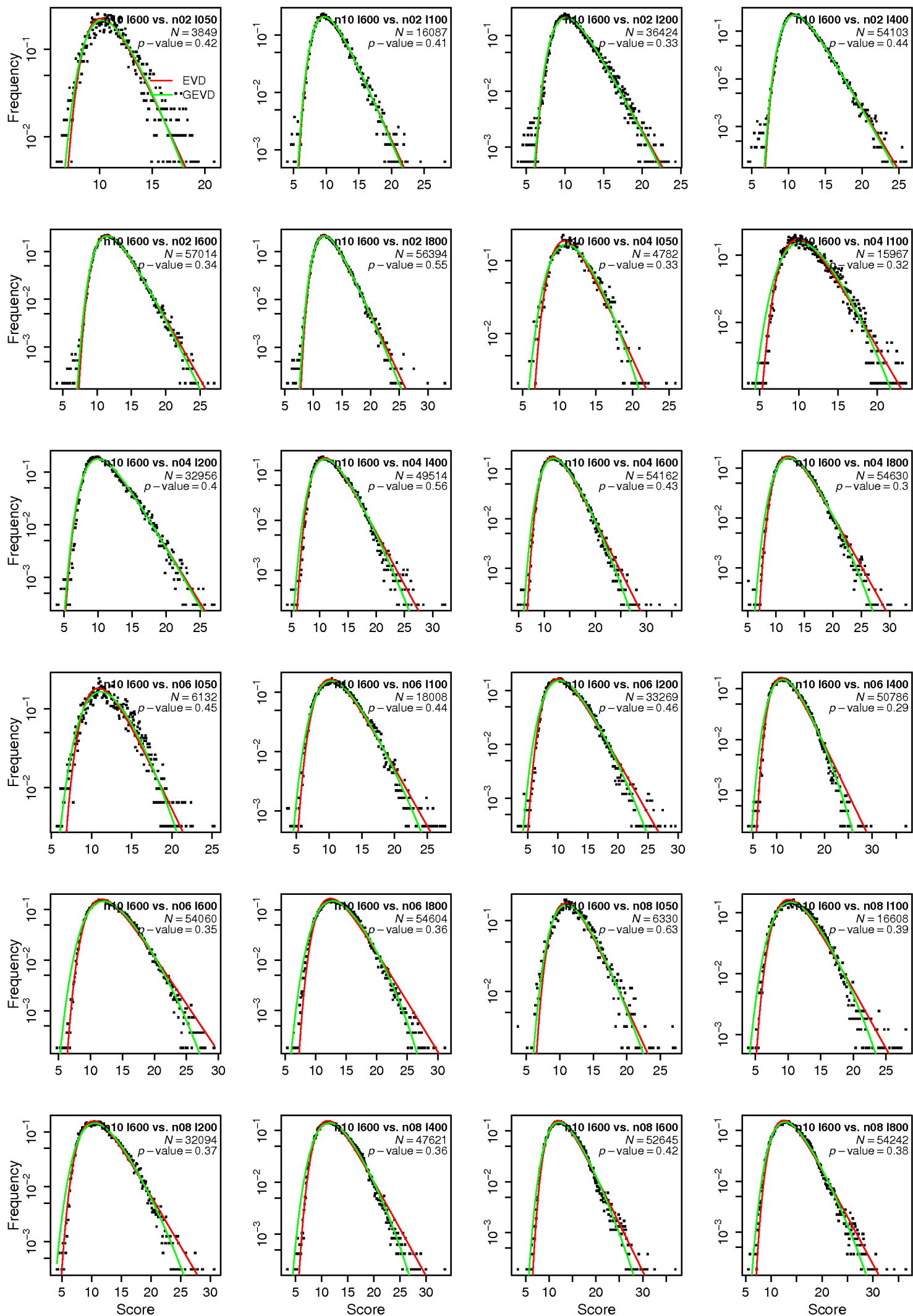

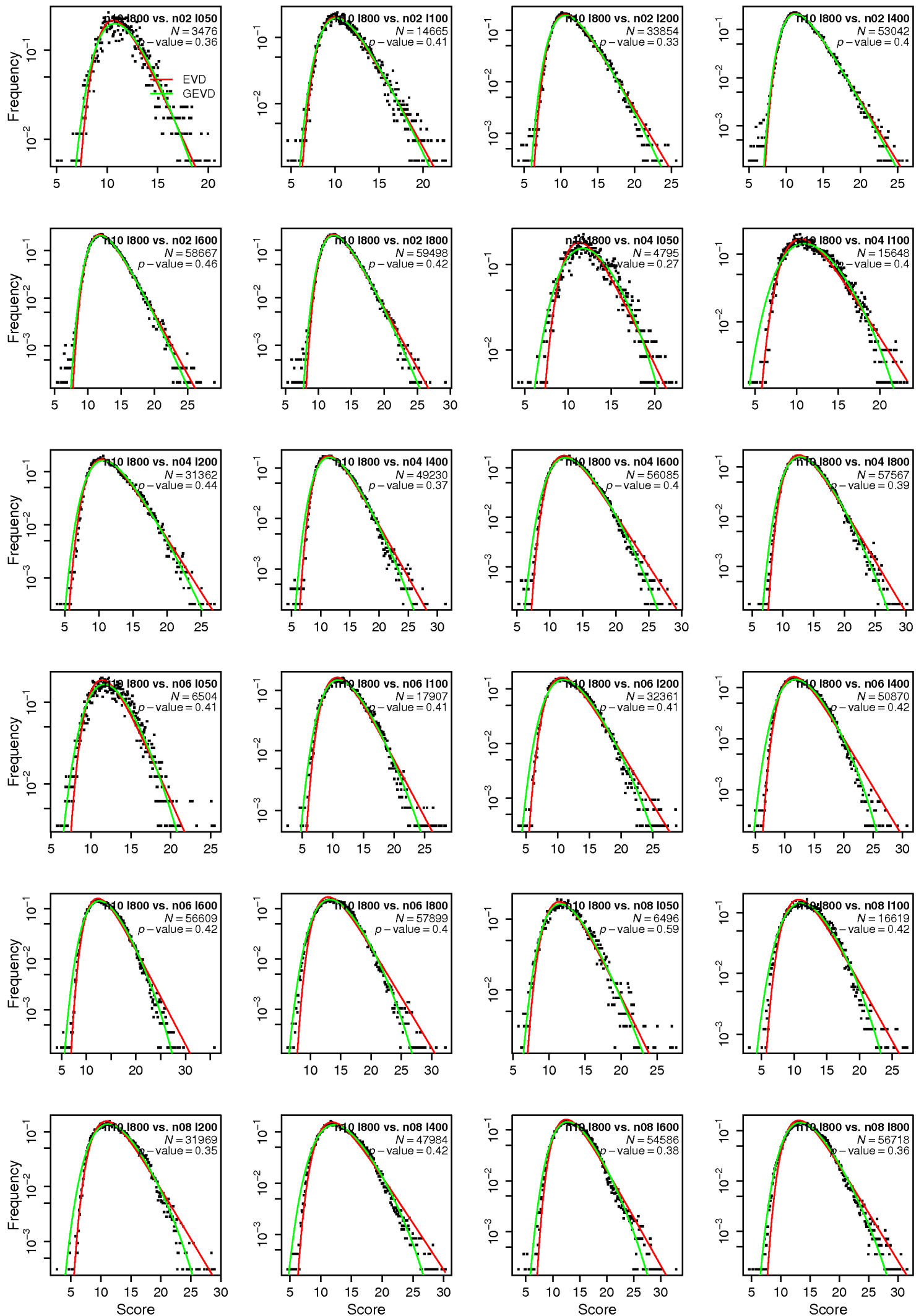

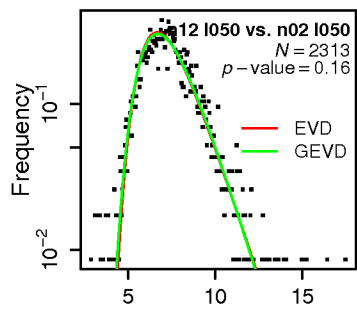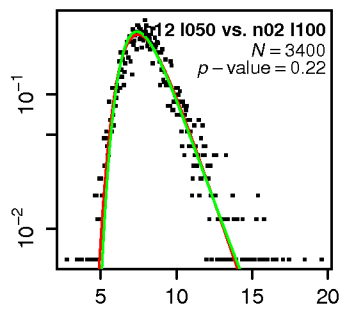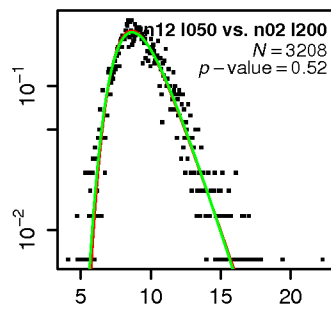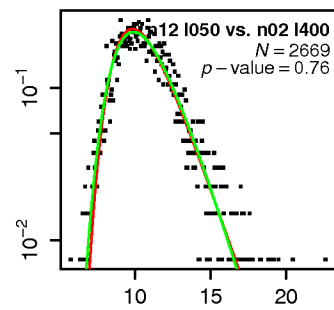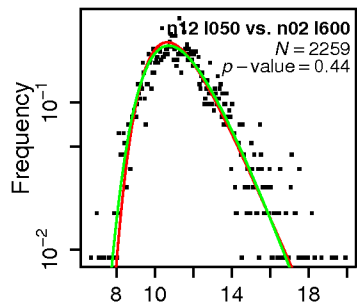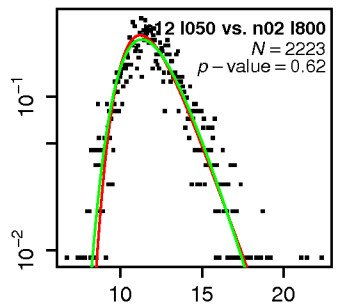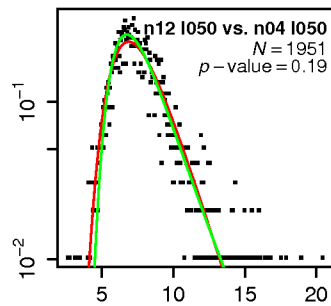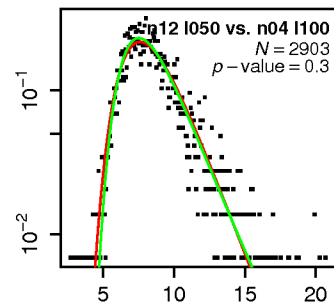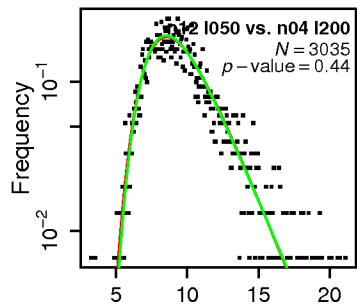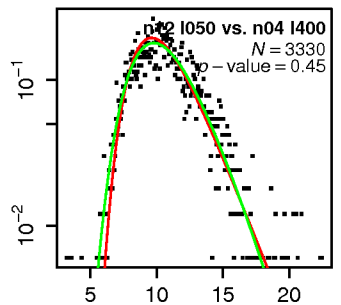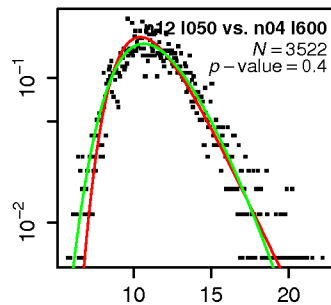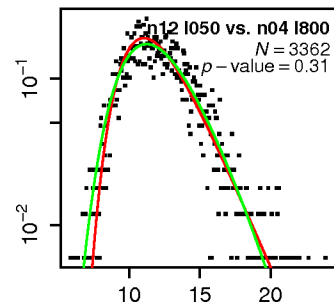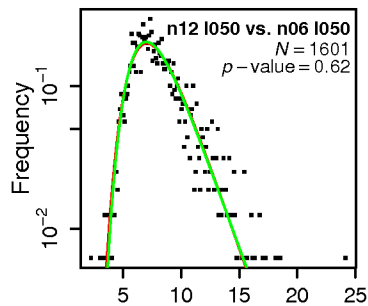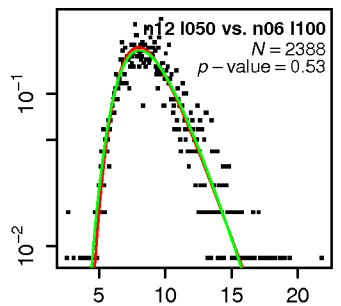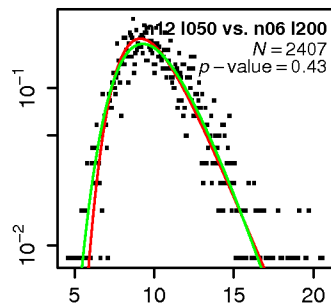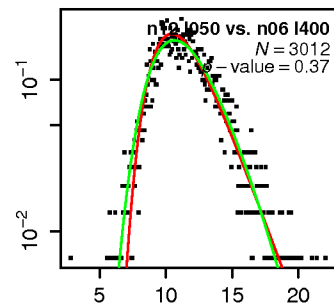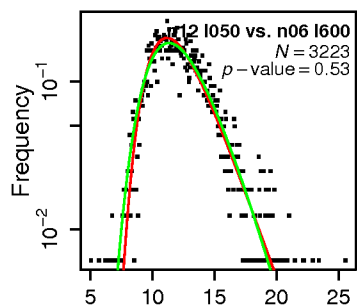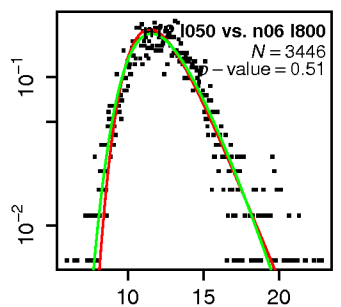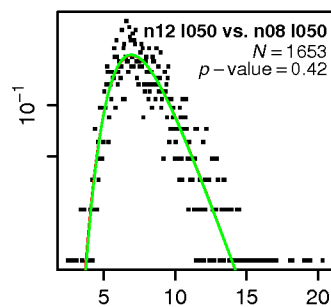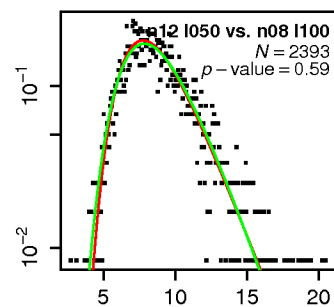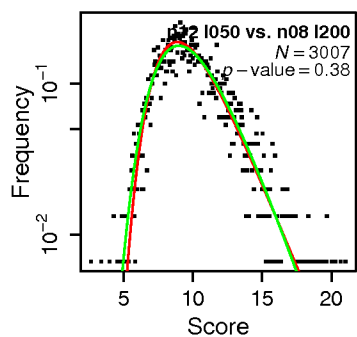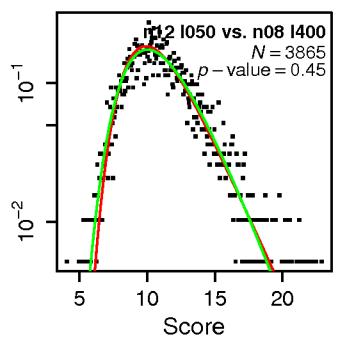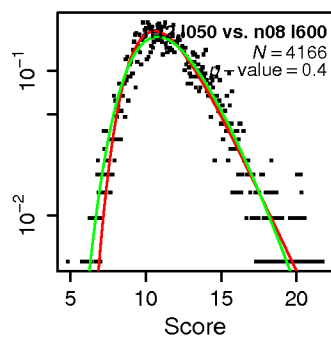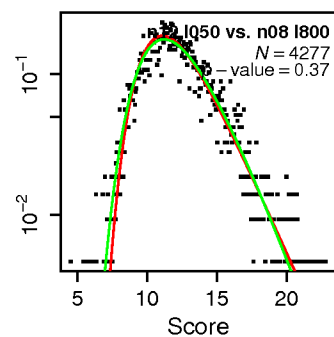

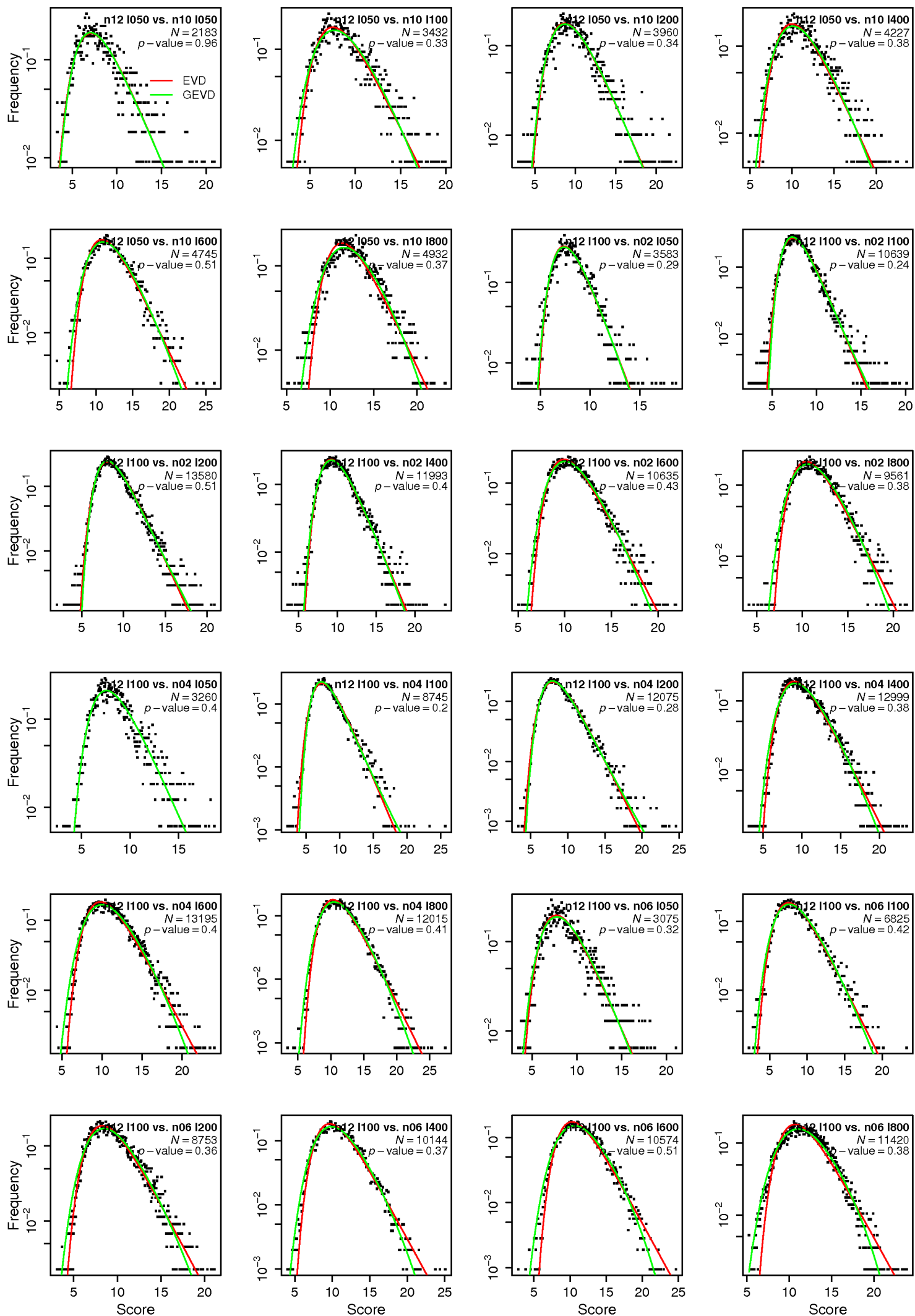

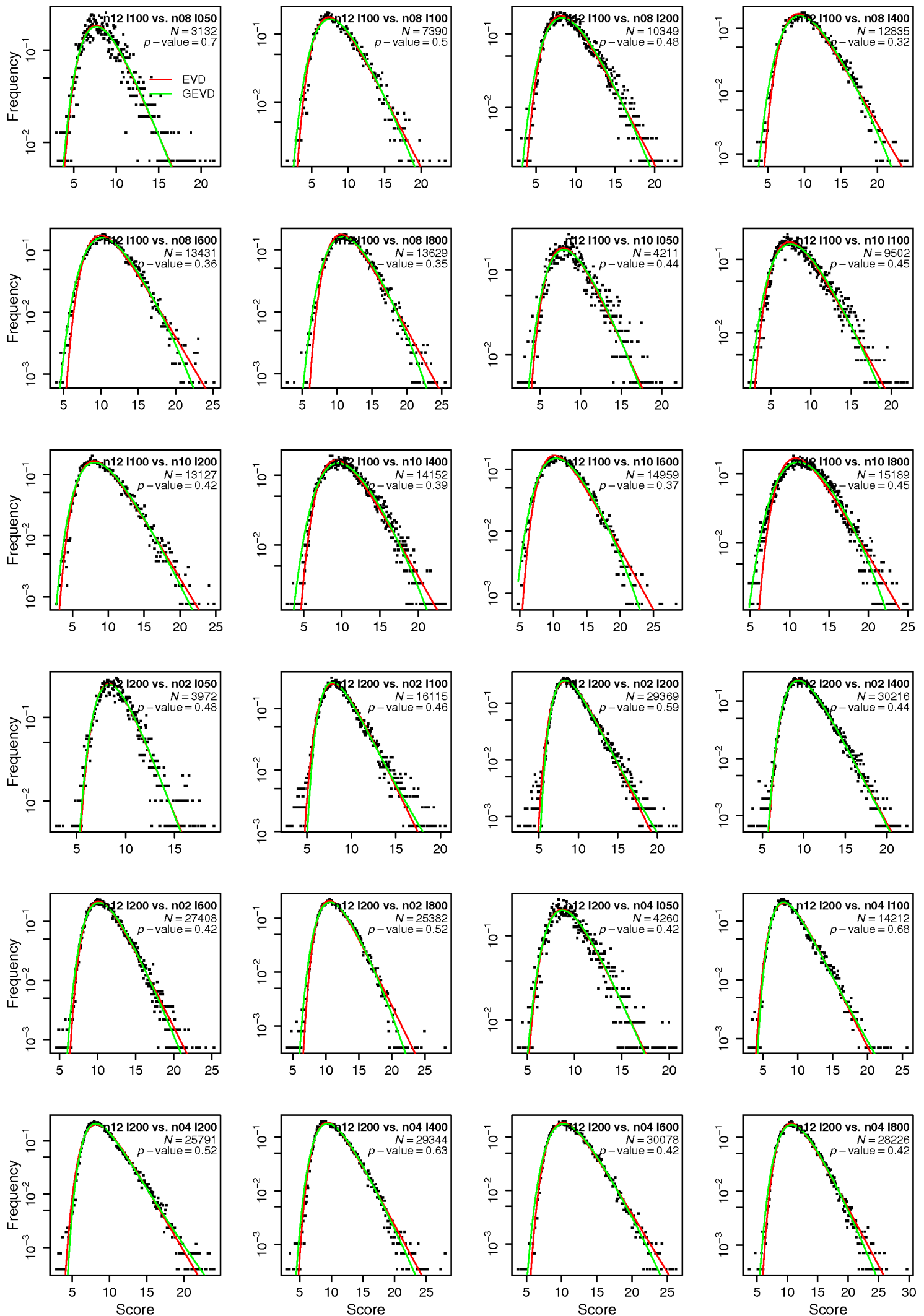

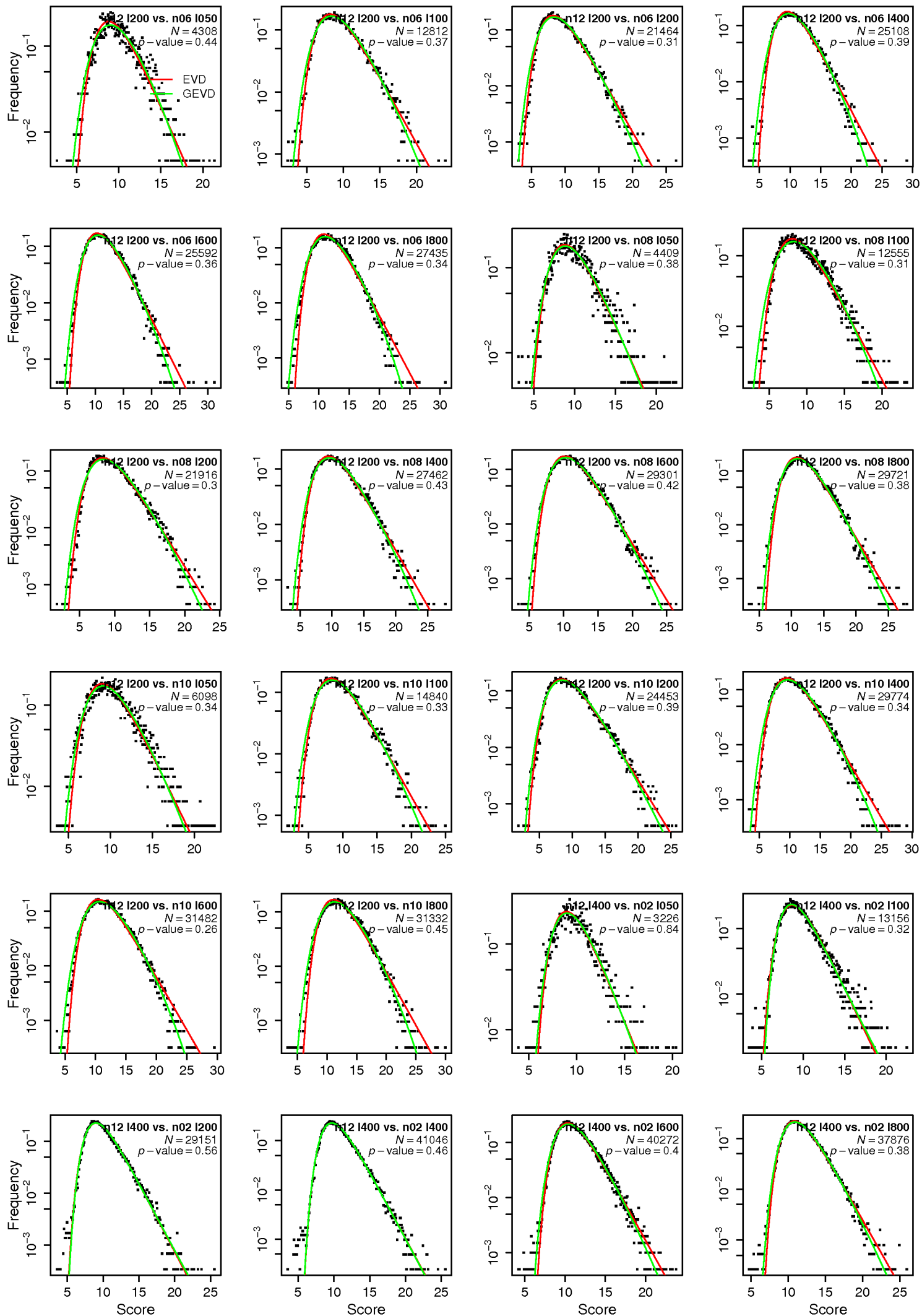

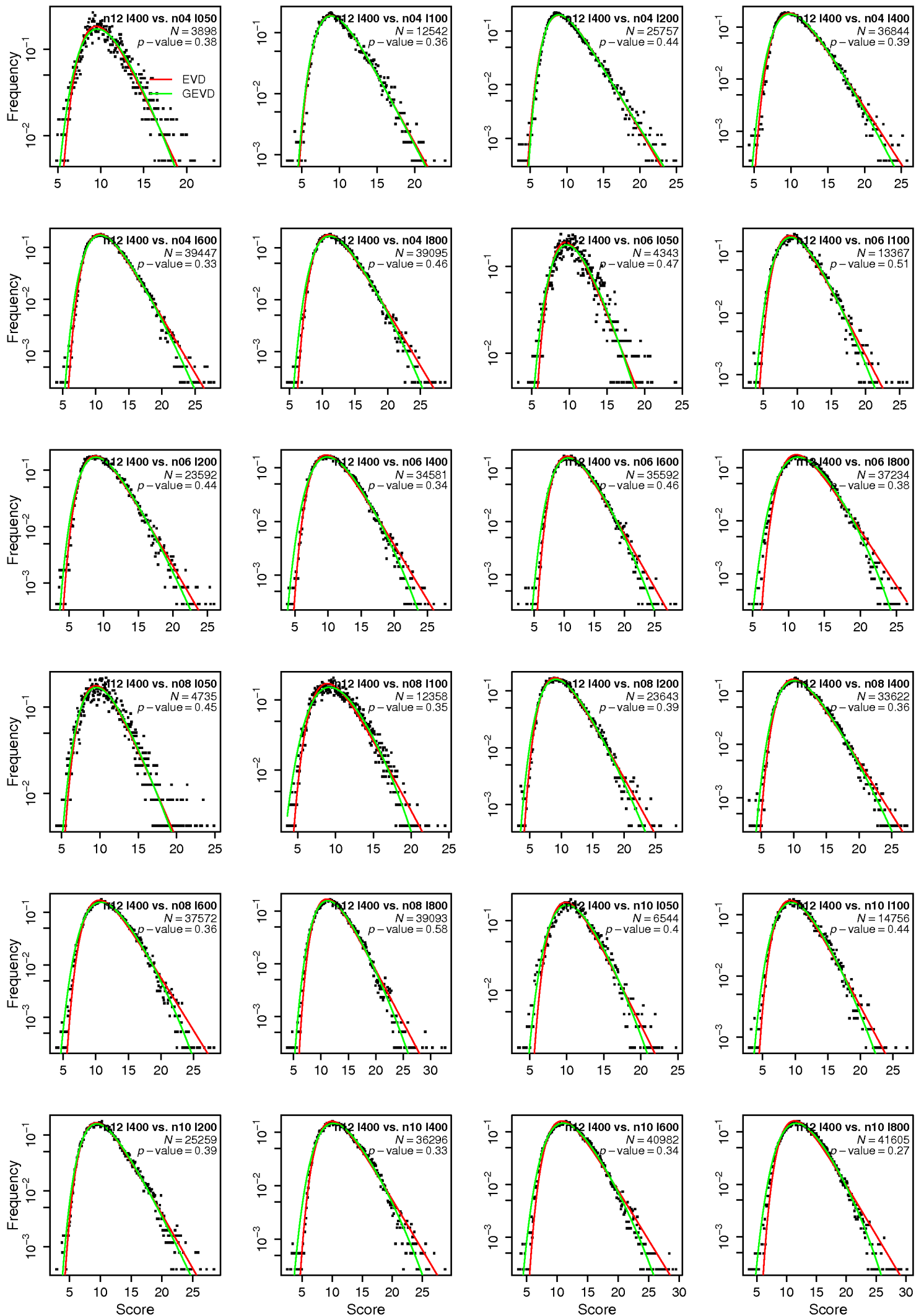

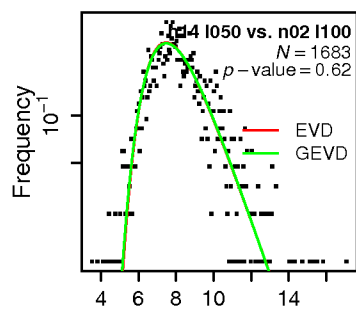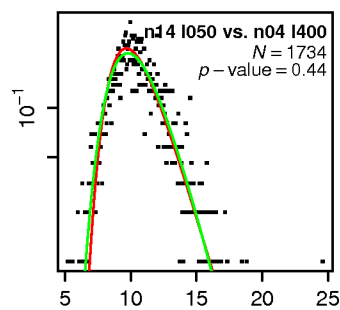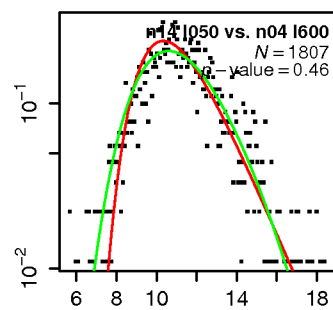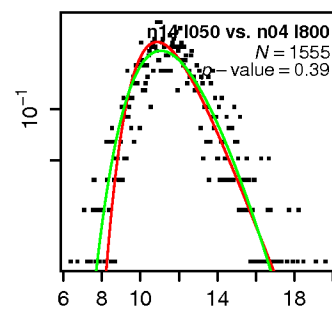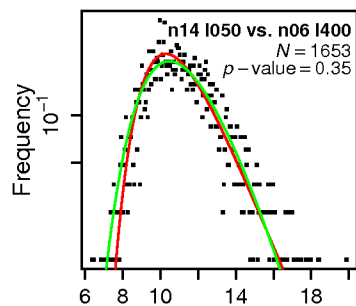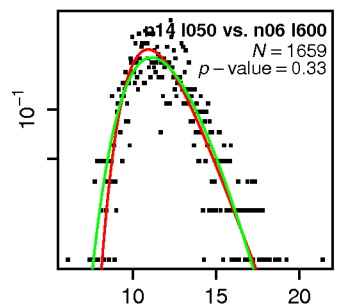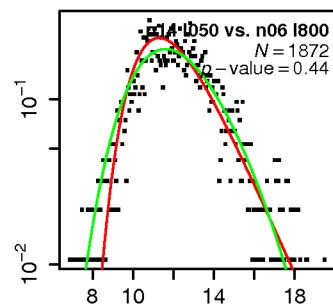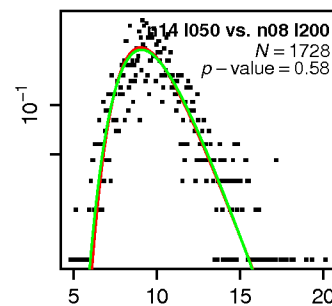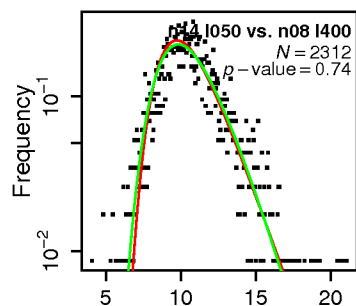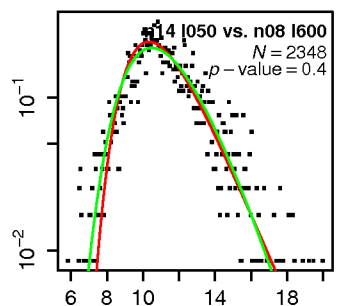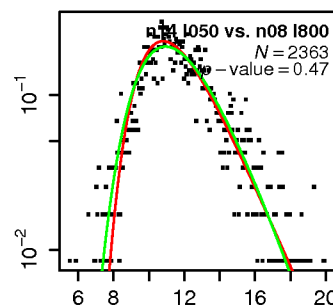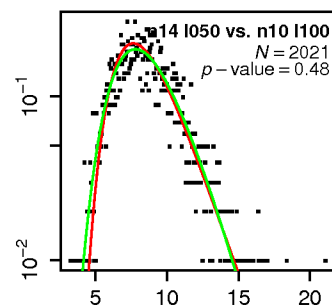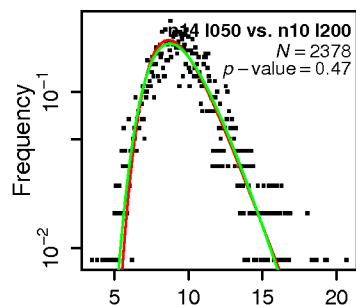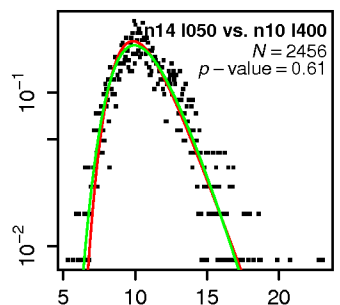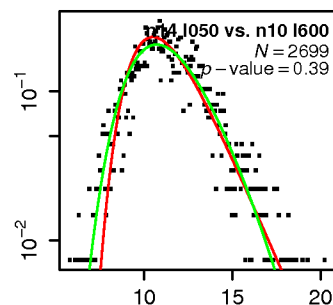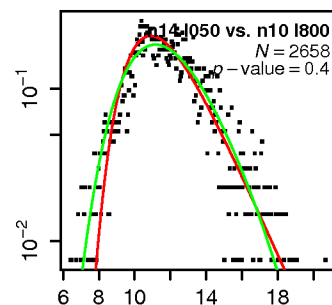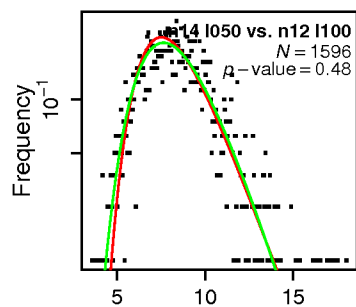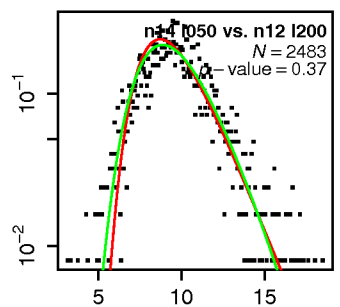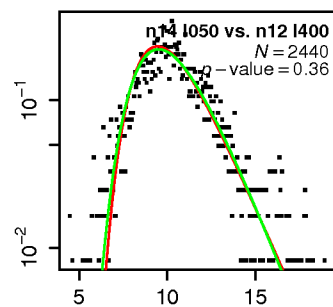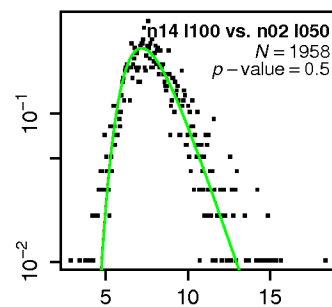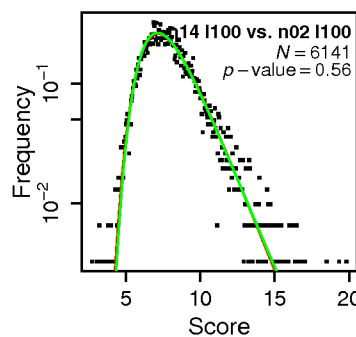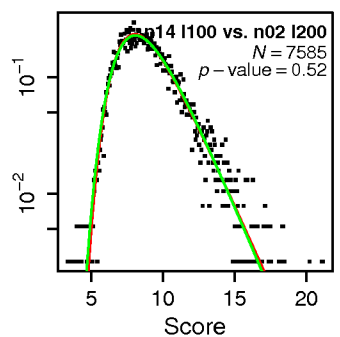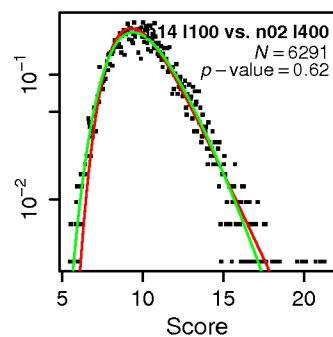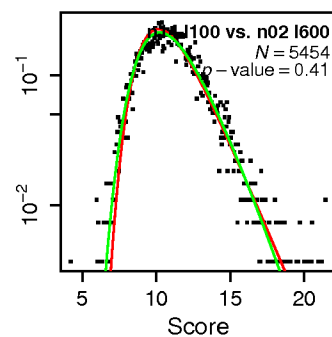

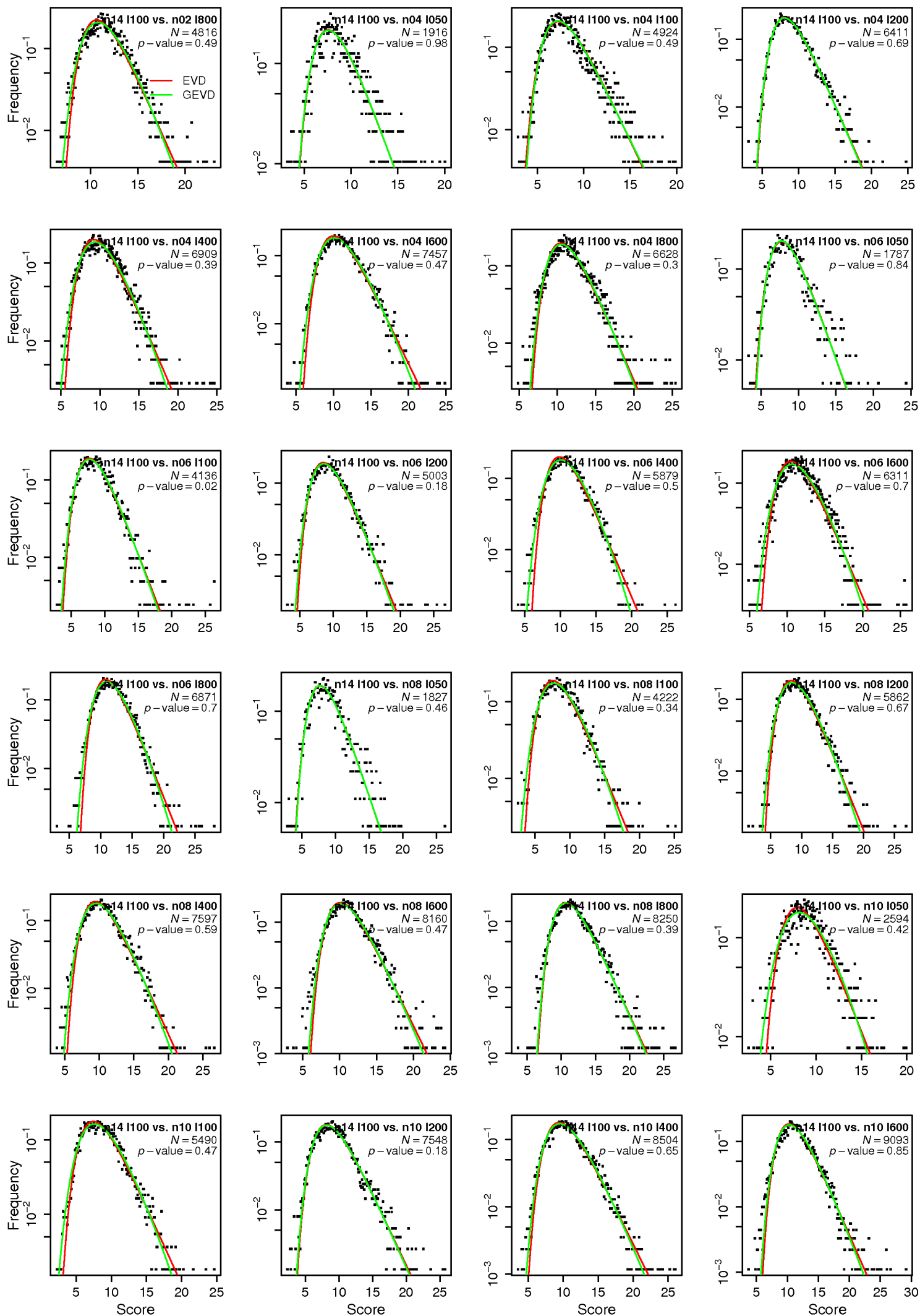

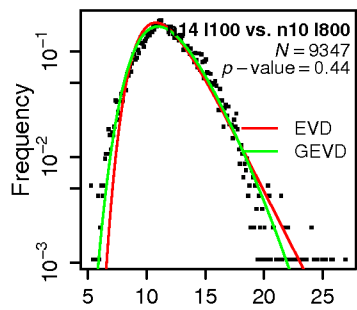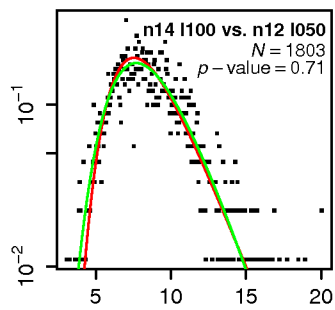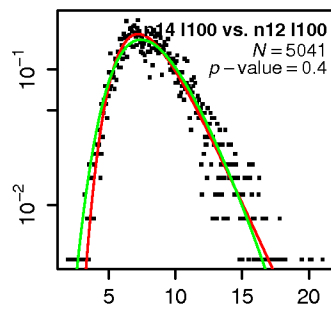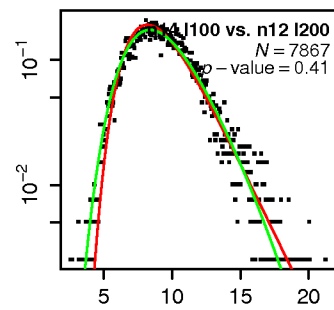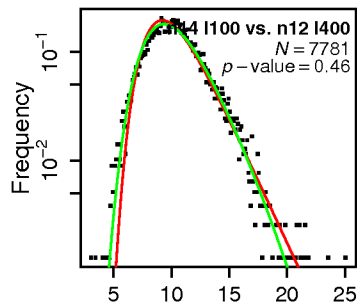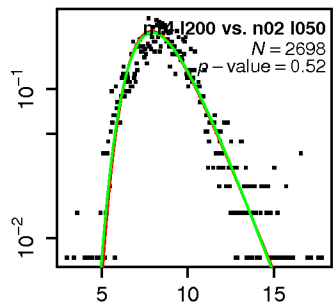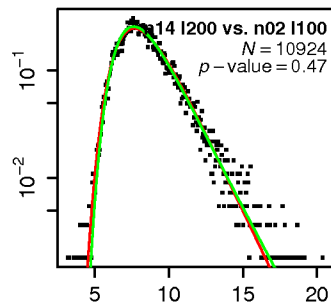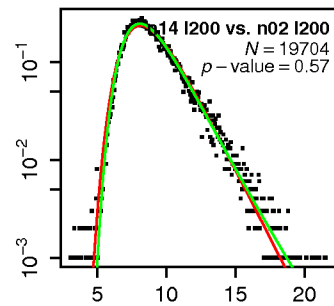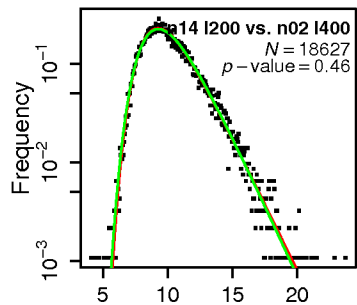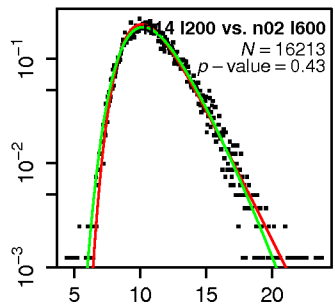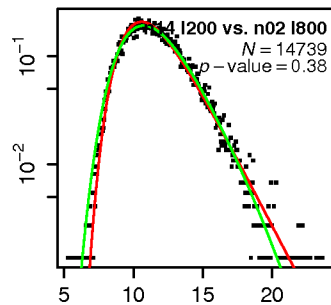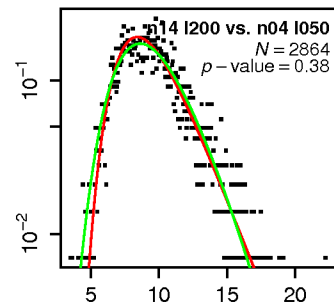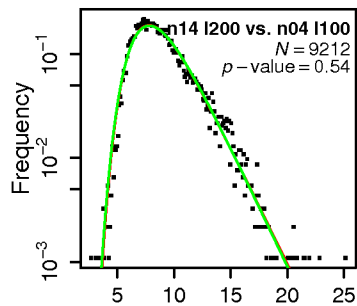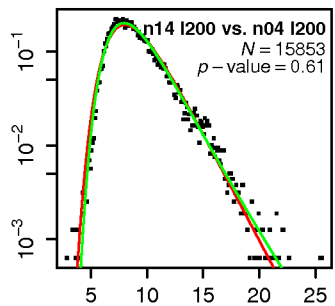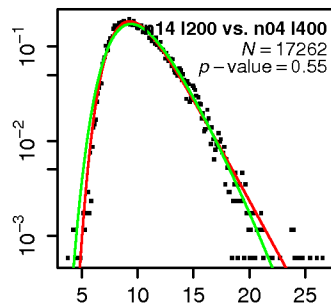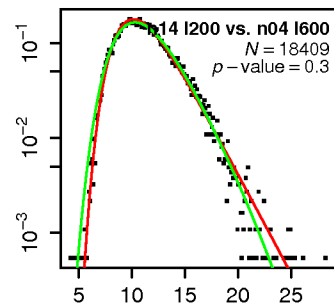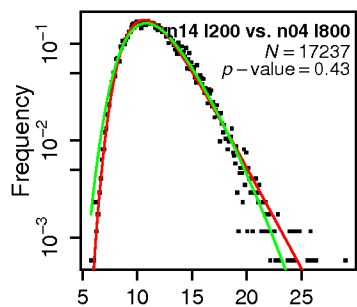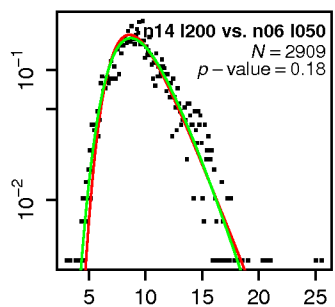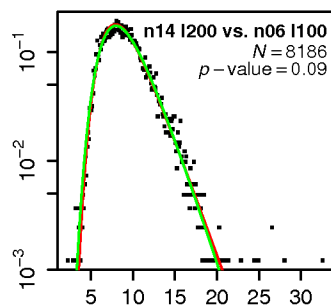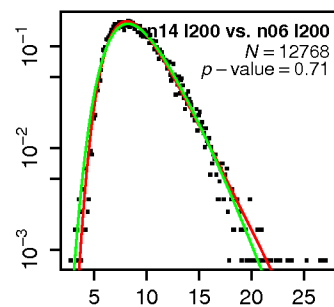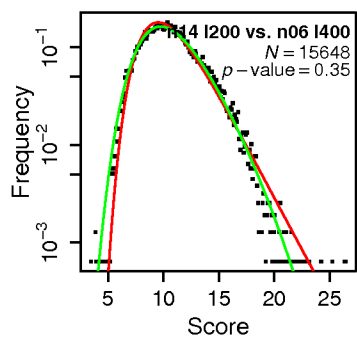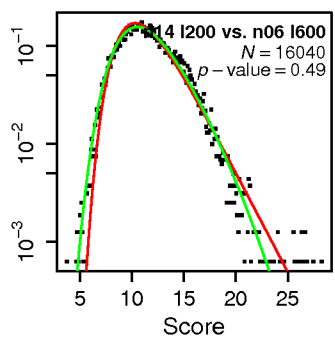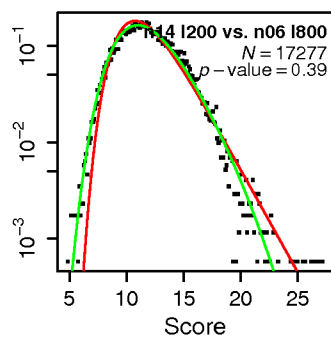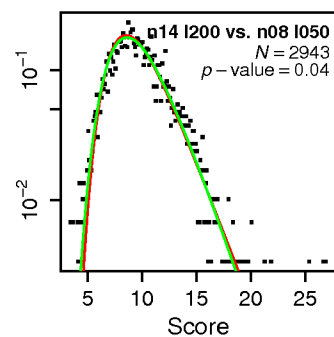

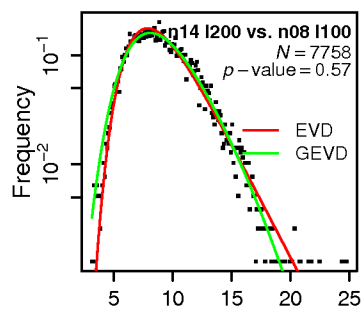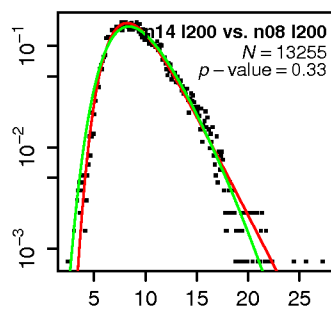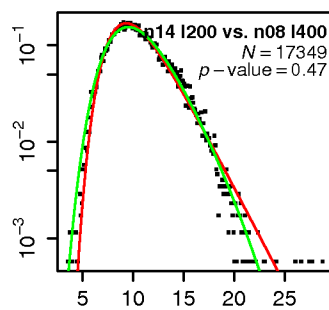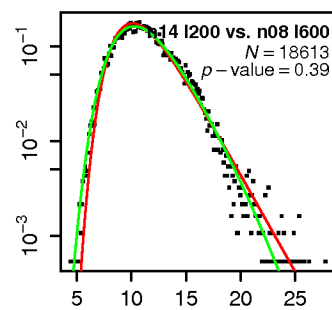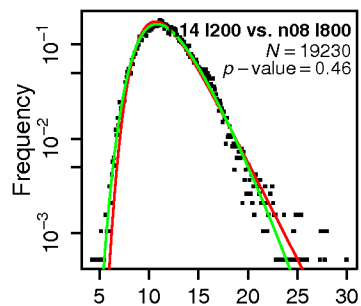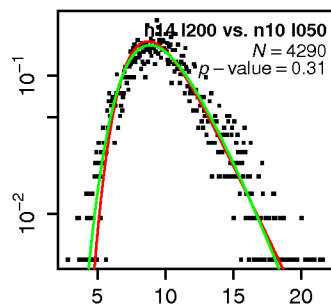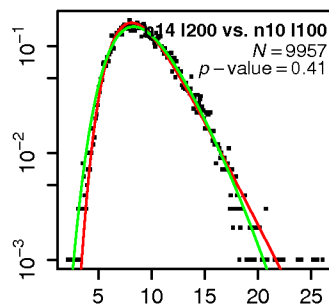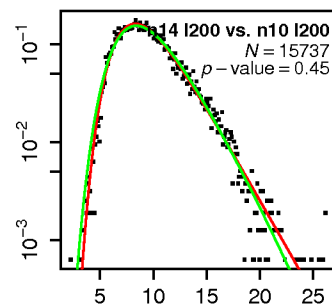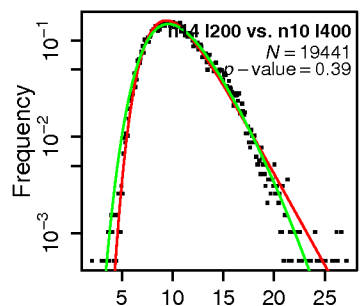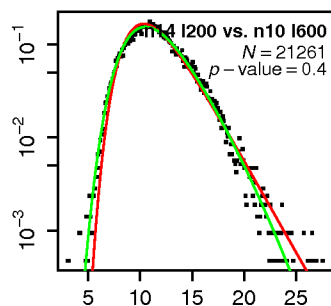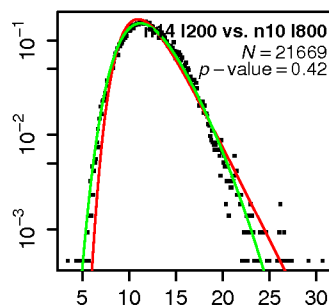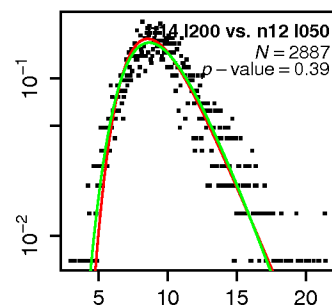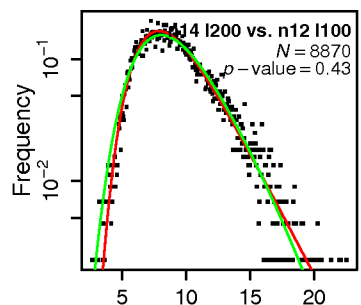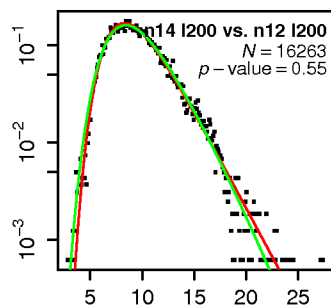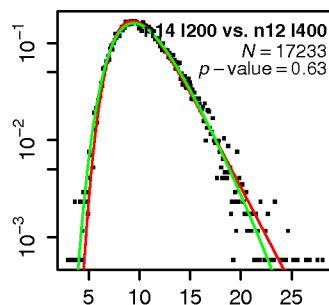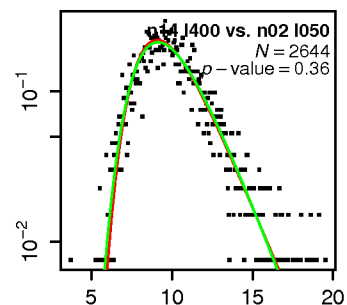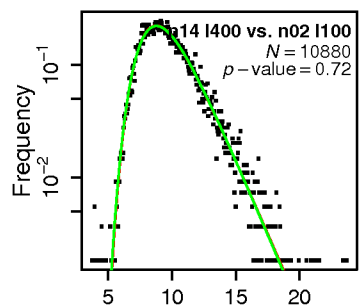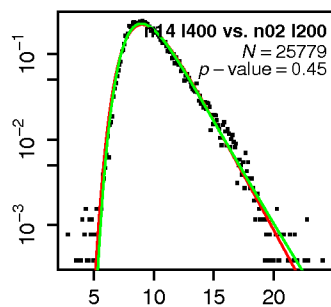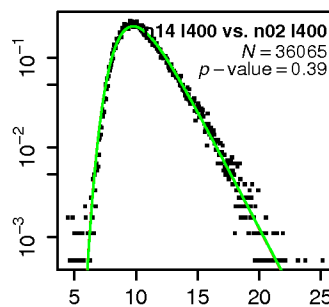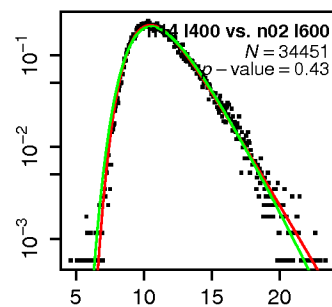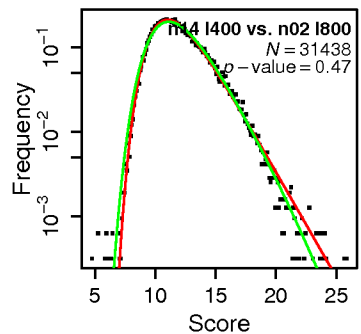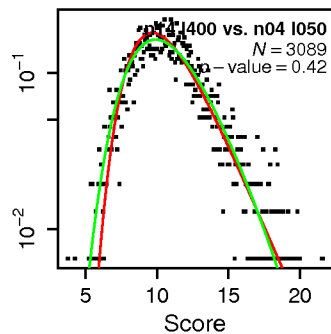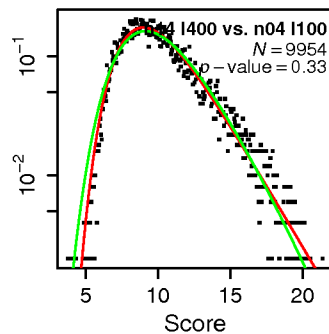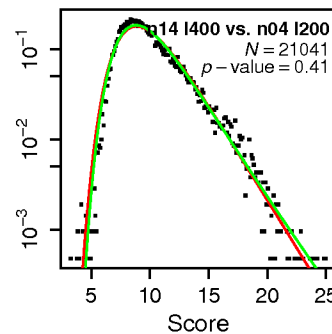

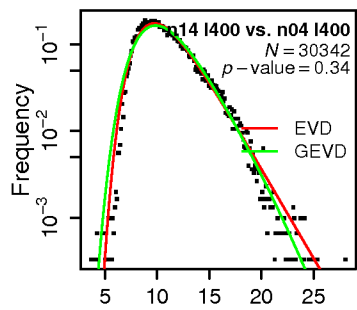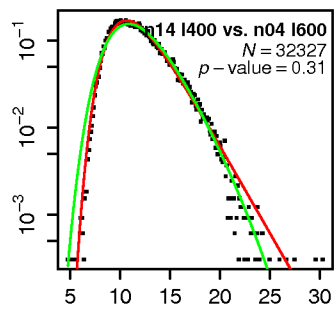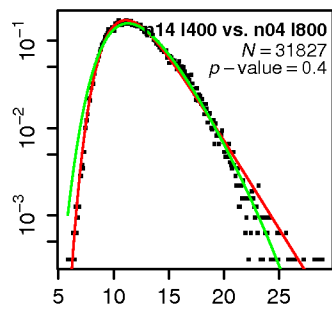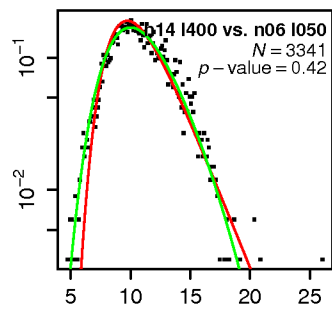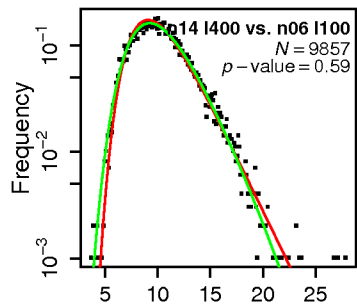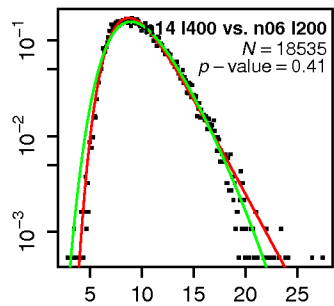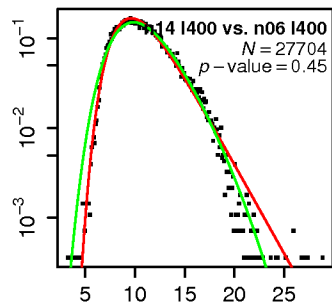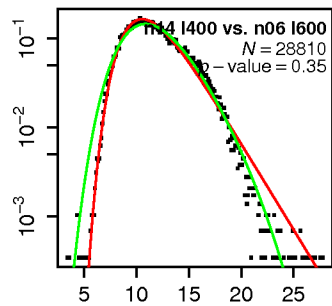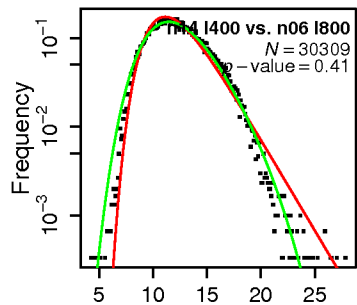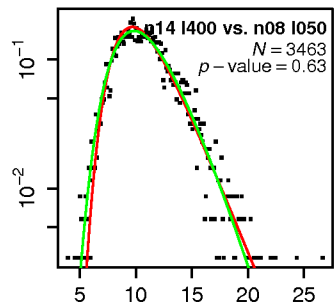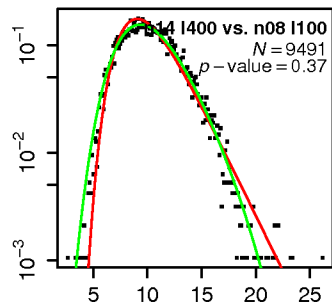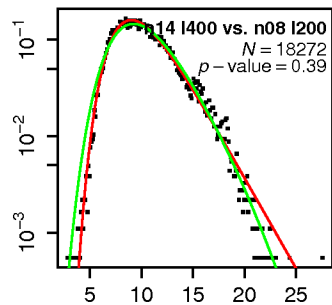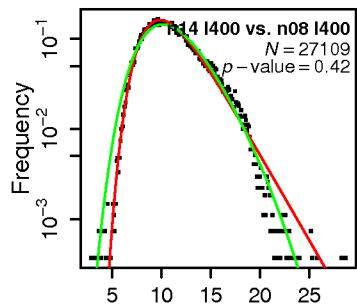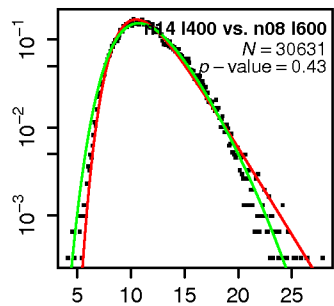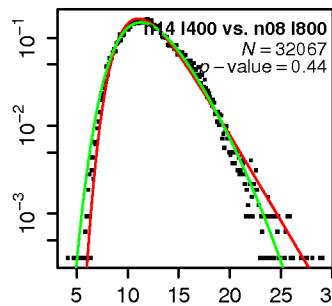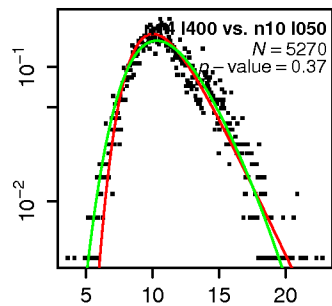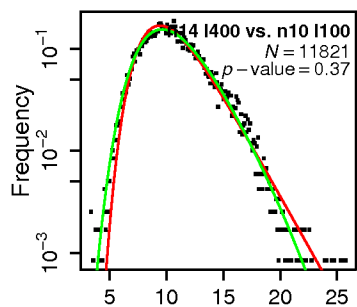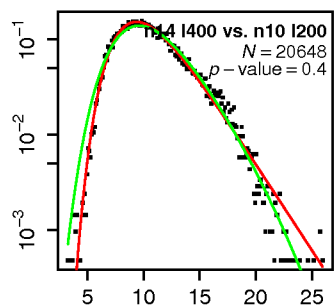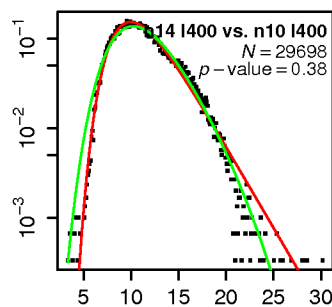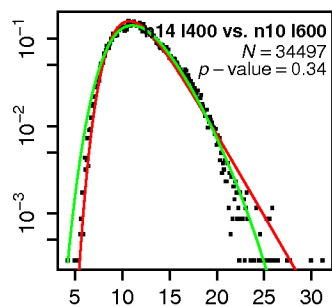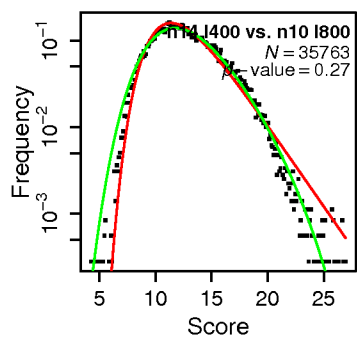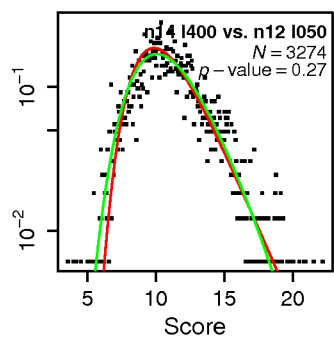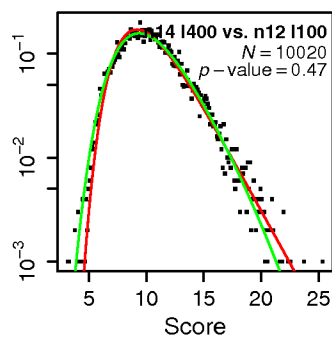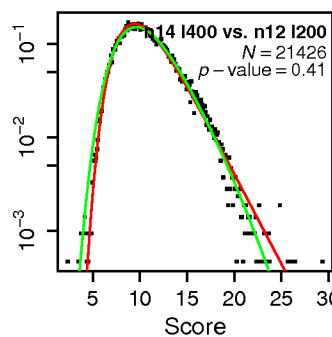

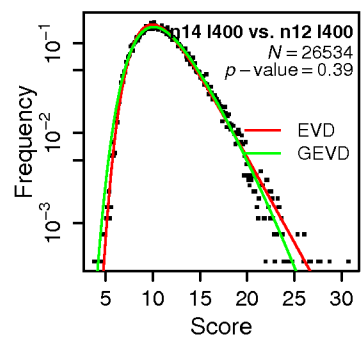

Supplement: Supplementary file 4 — Figure S8. Distributions of alignment scores of simulated profiles for different pair values of profile ENO and length. (PDF 6,262 kb) [file 12859_2019_2913_MOESM4_ESM.pdf]

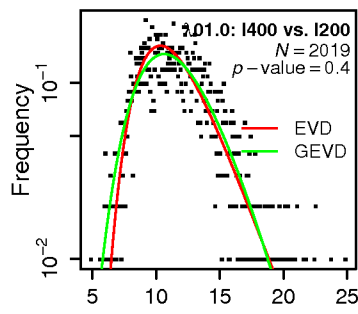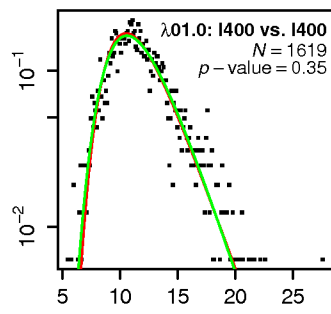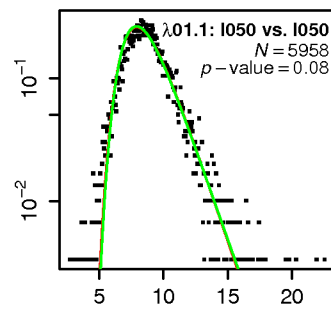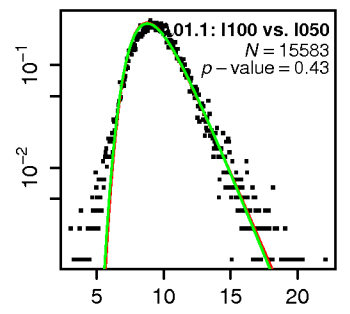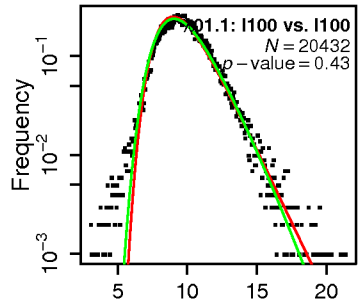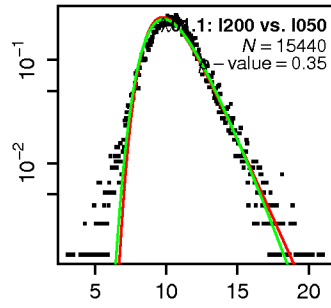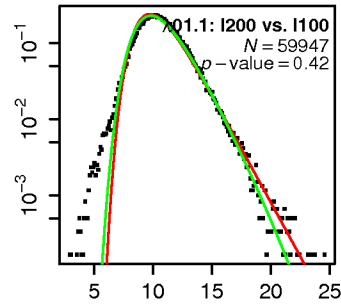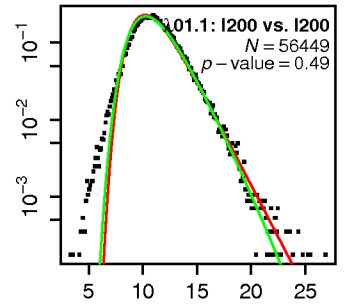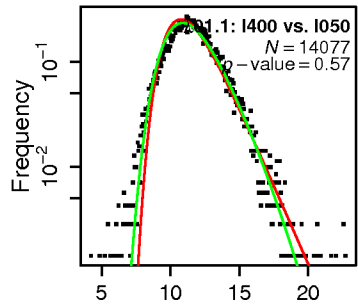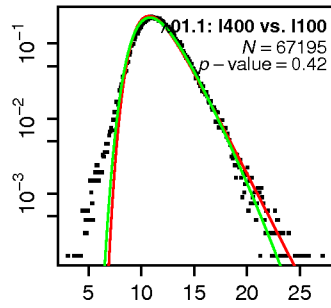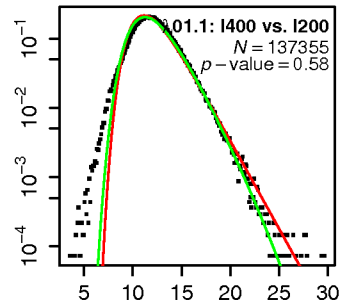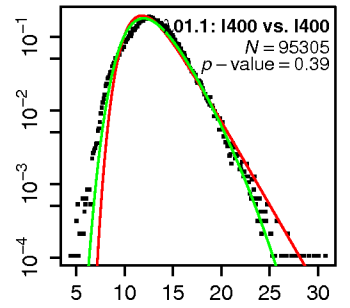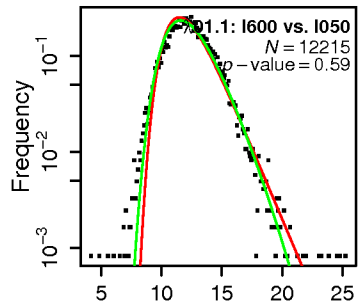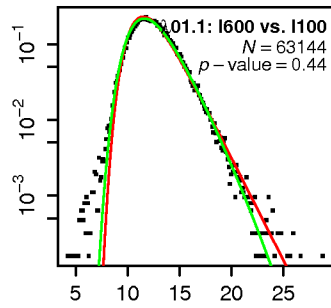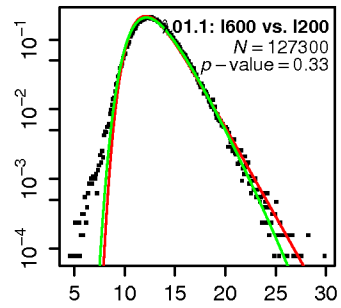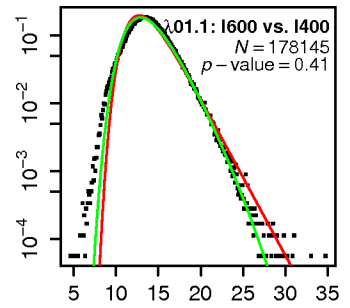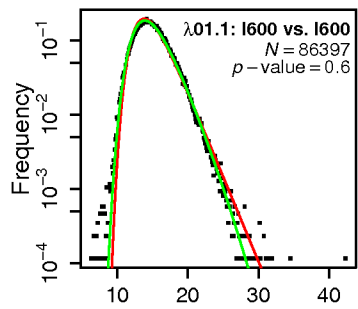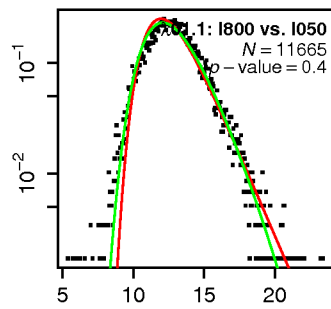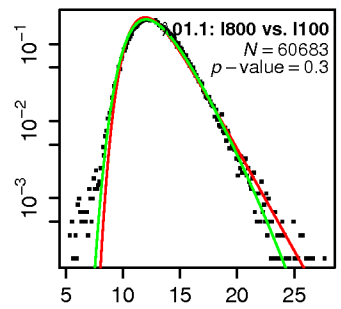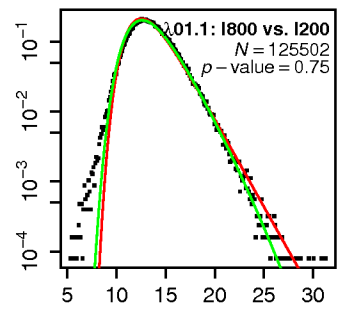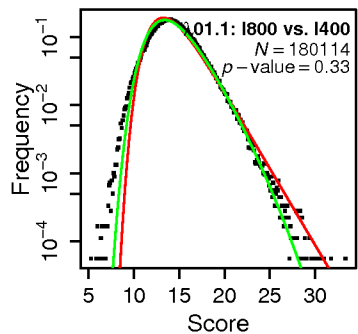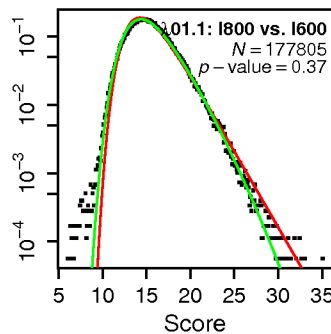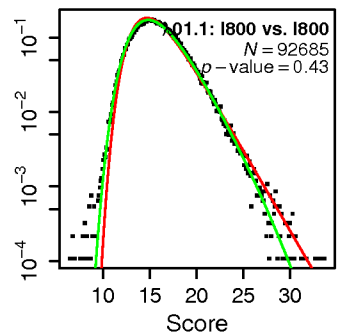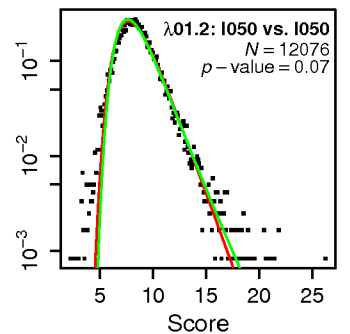

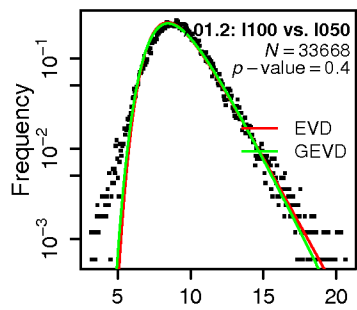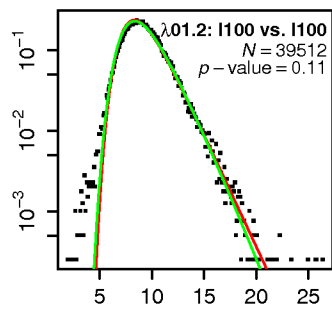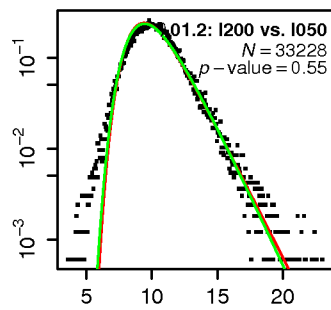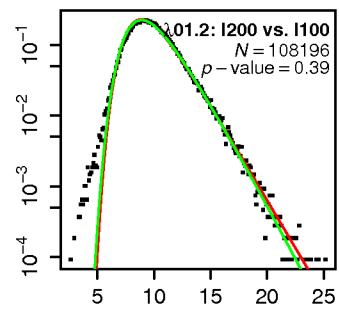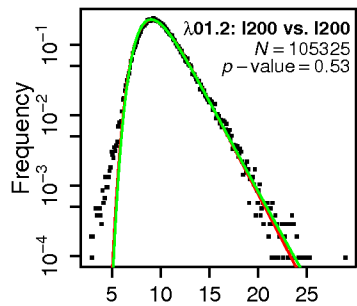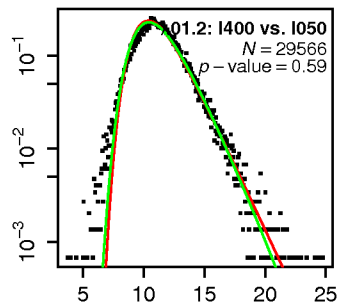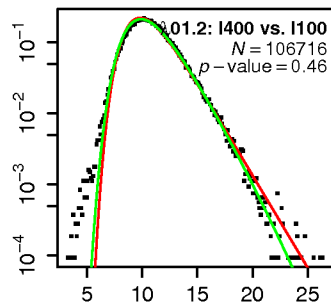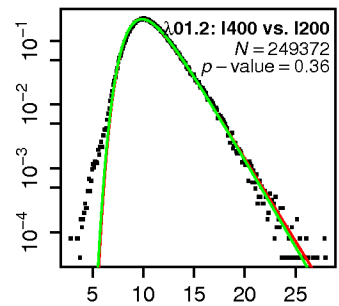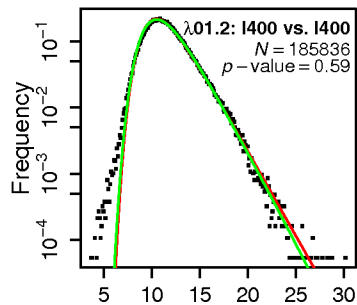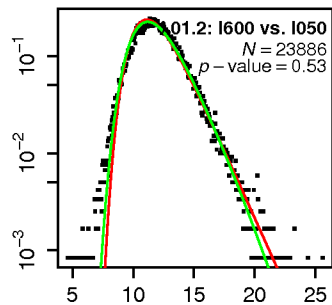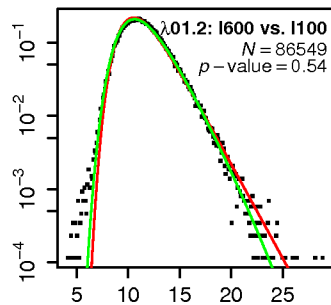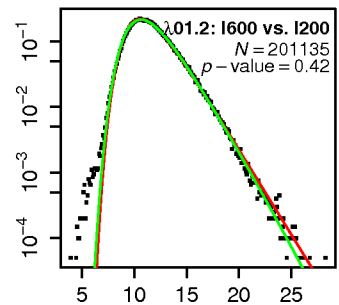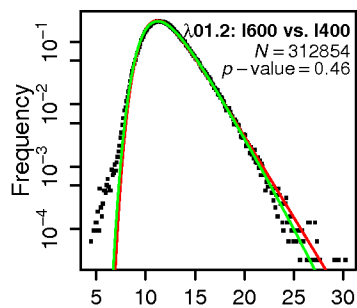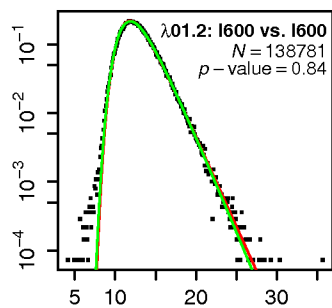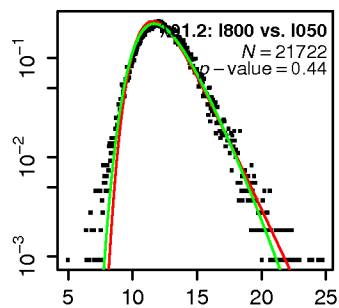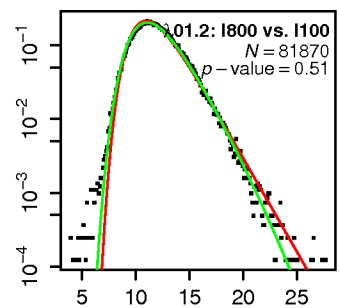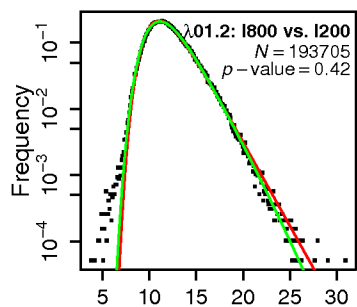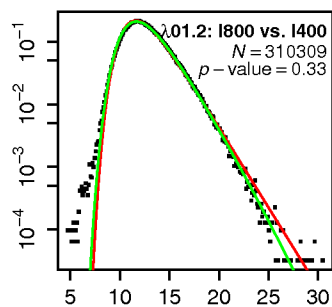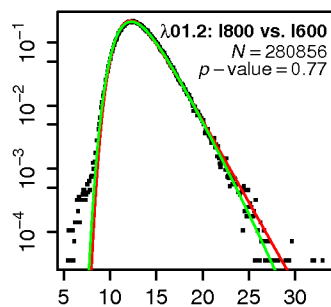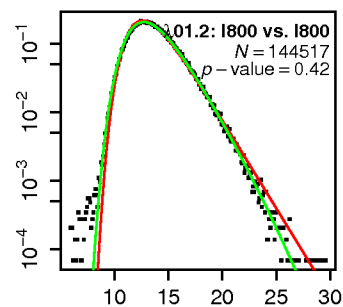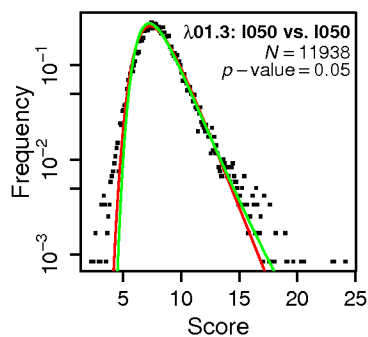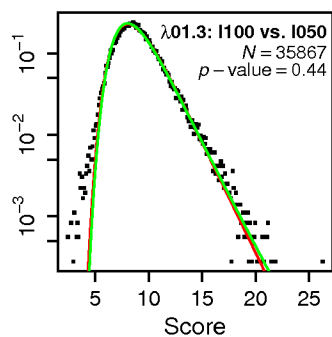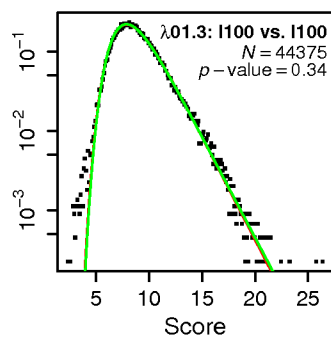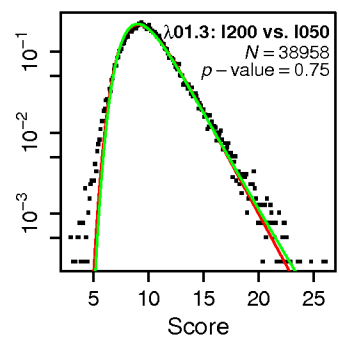

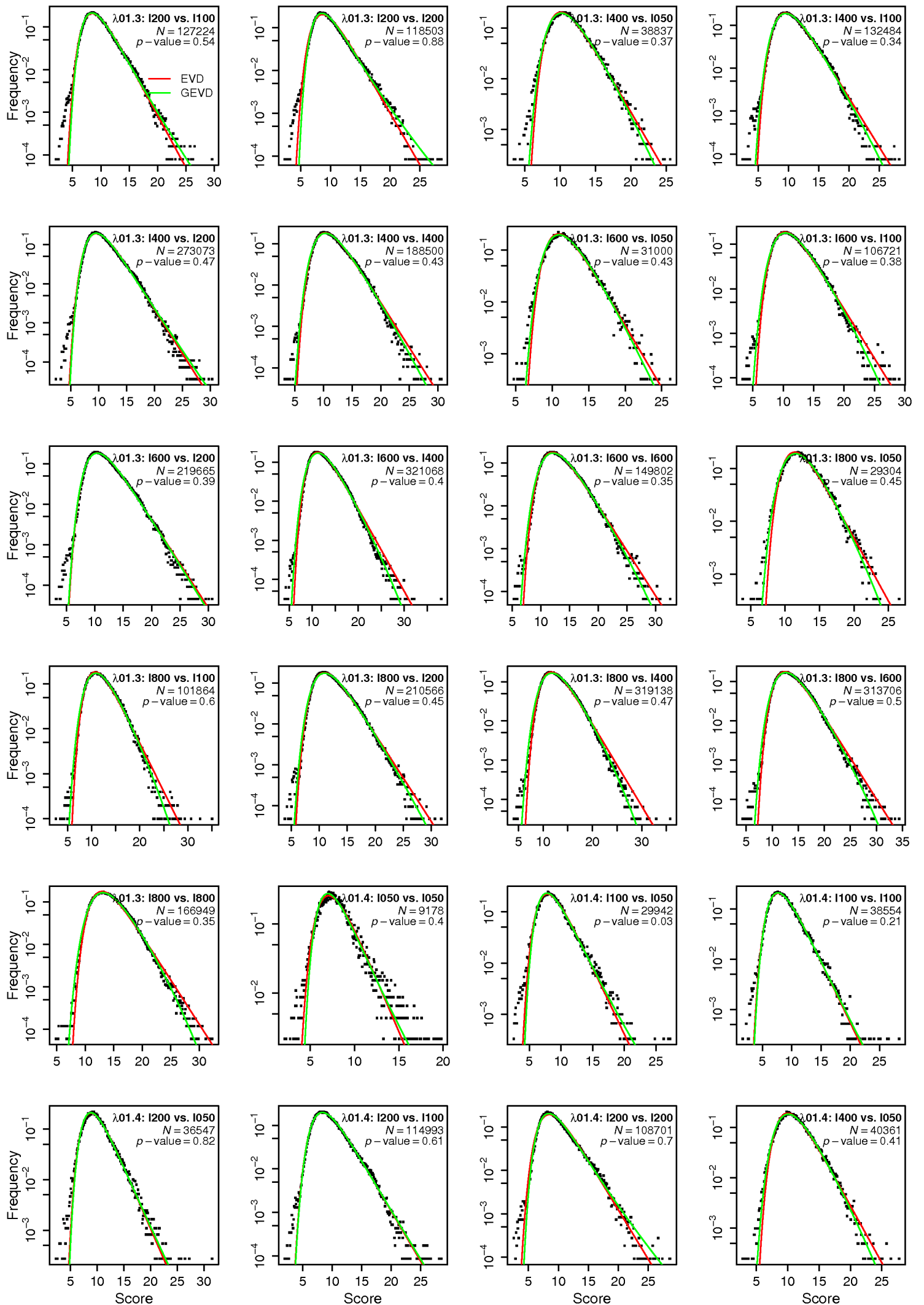

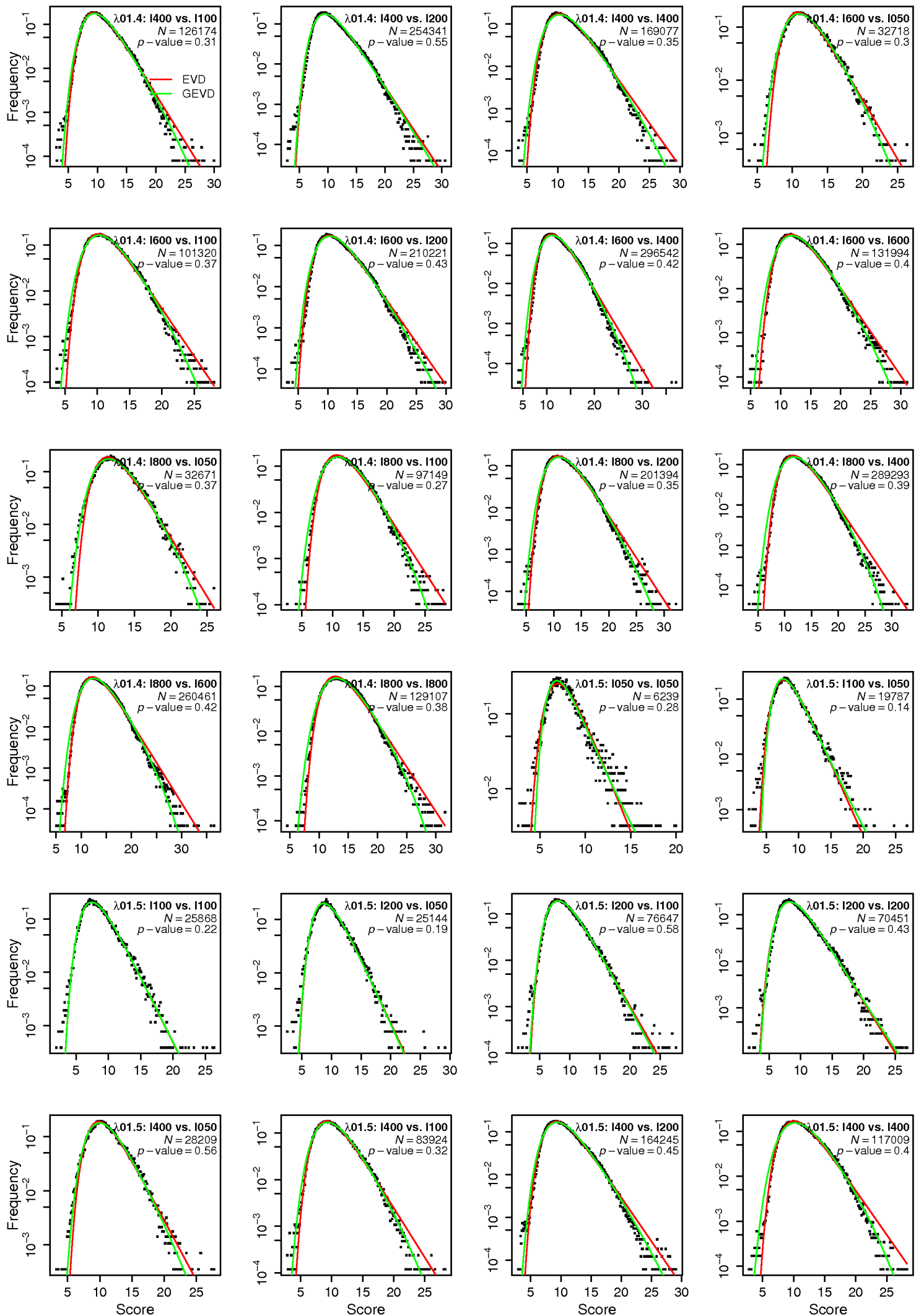

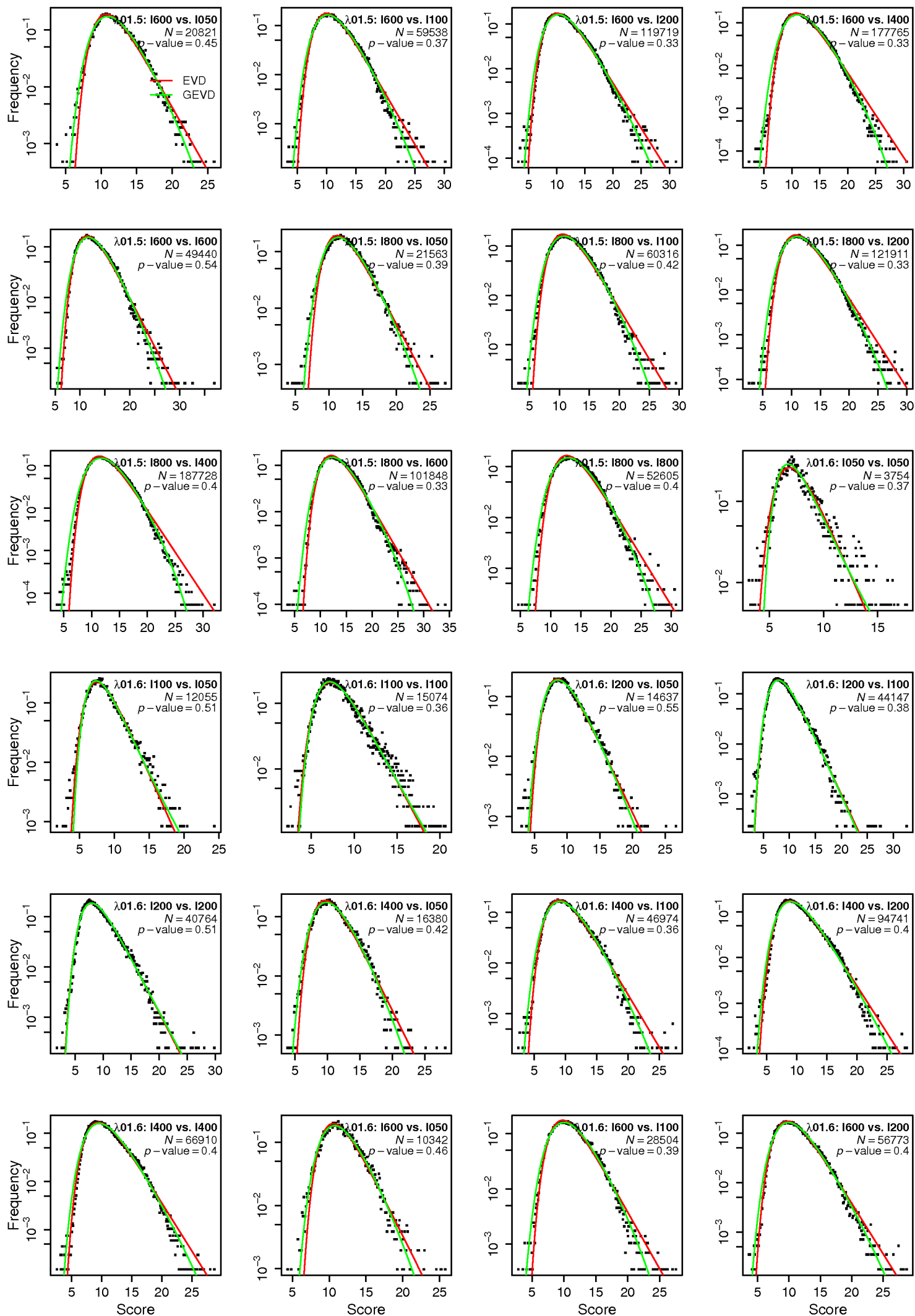

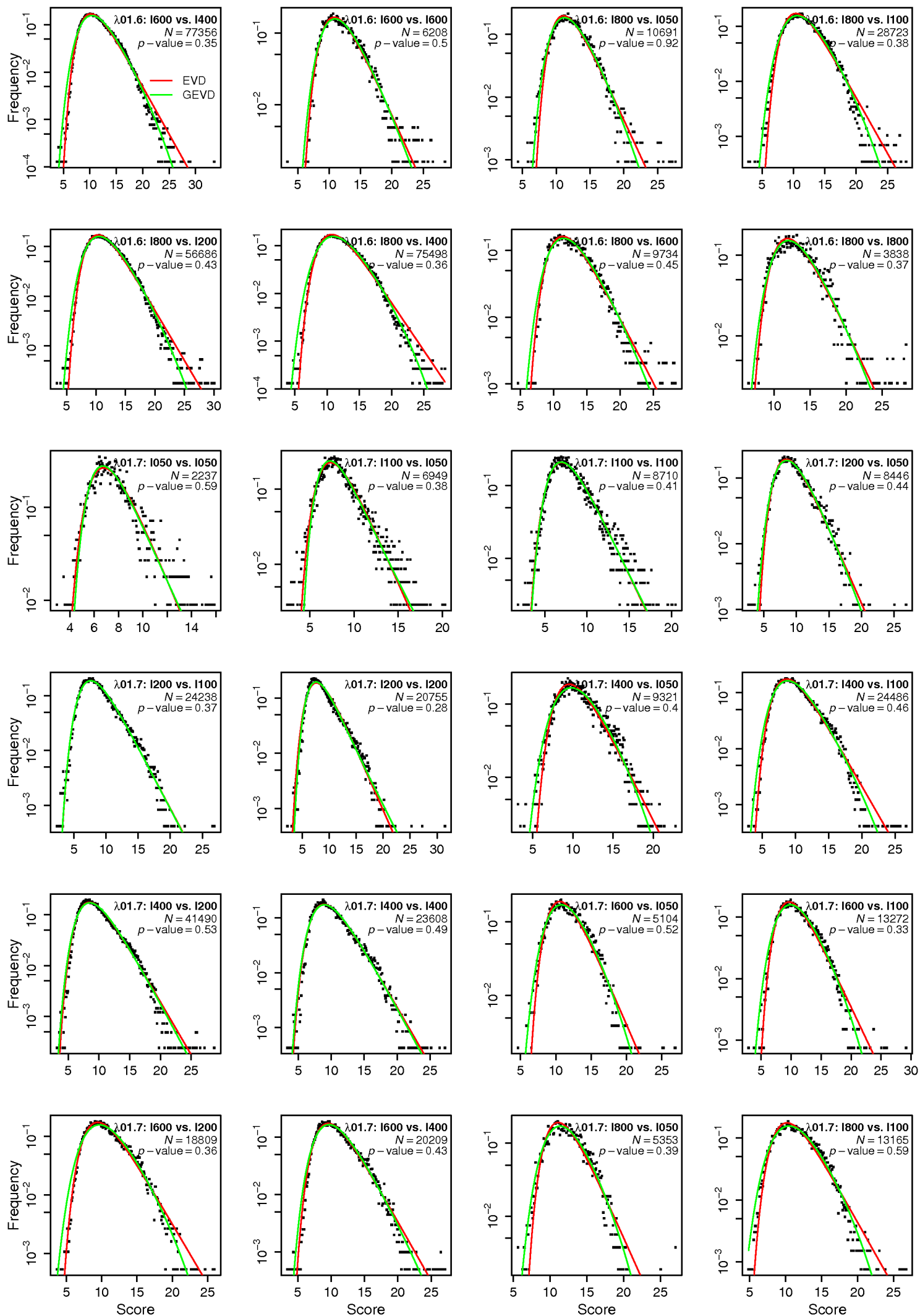

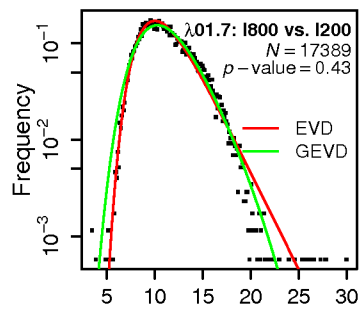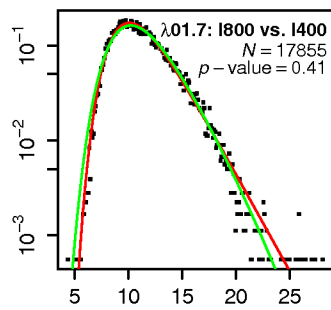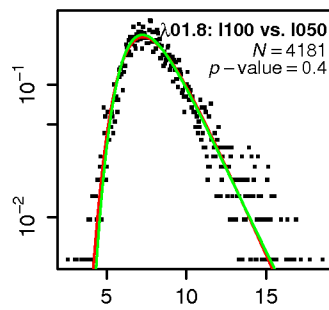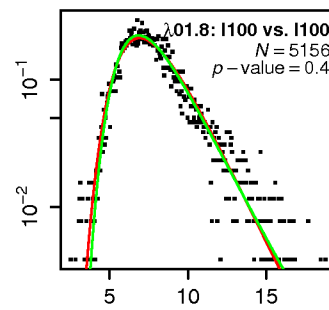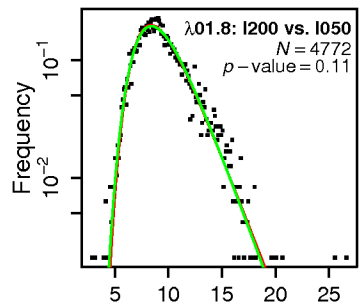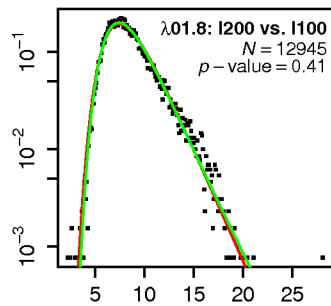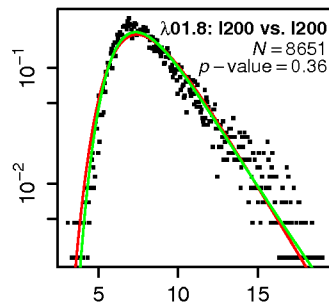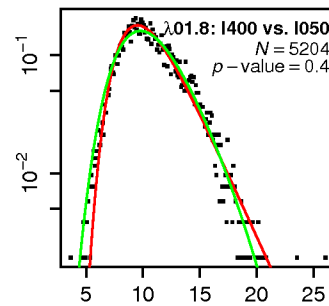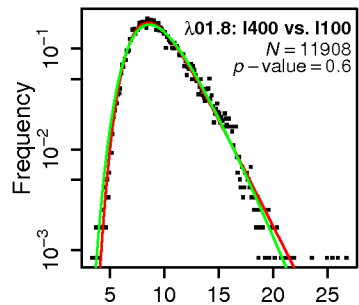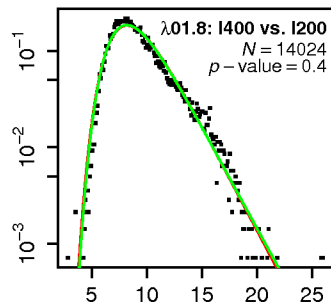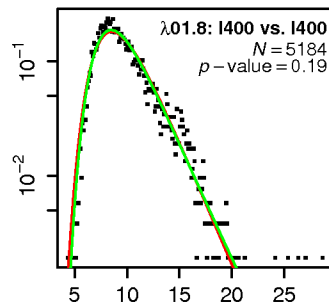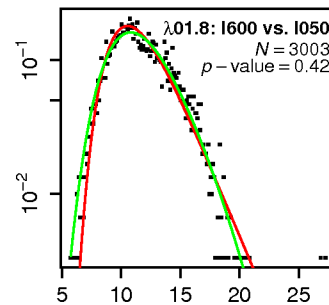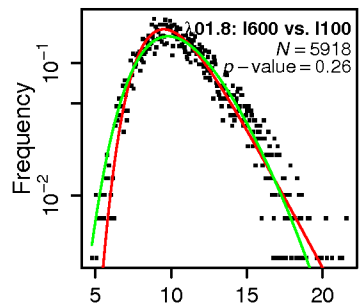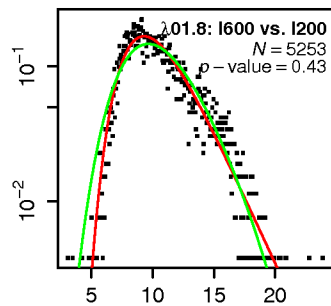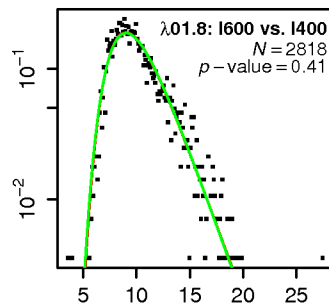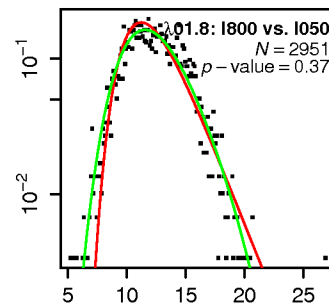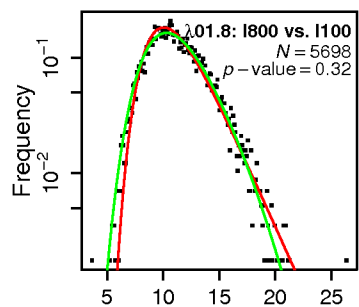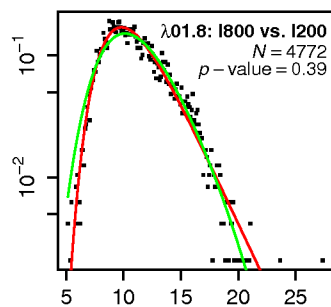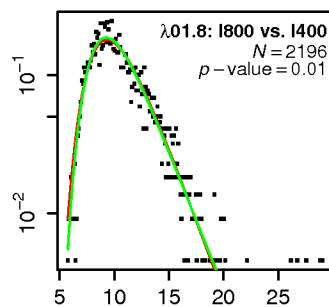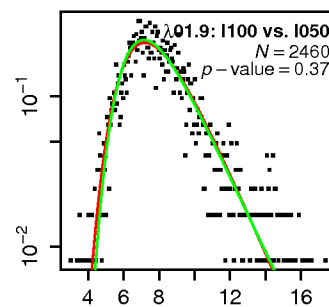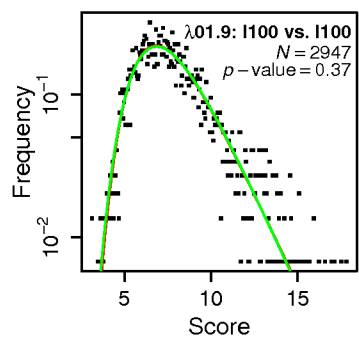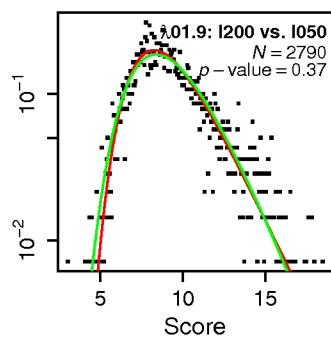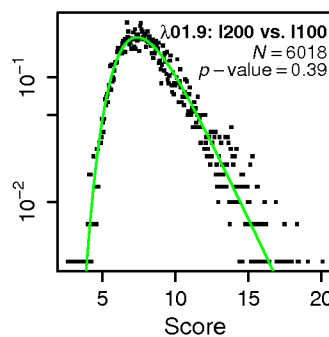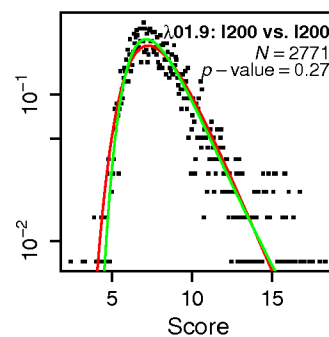

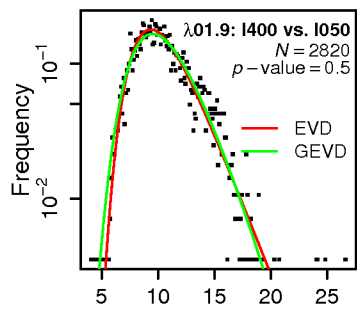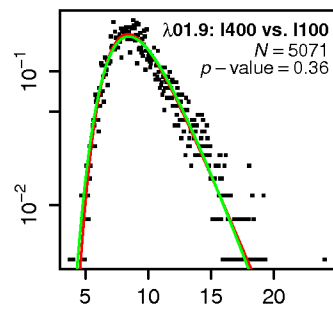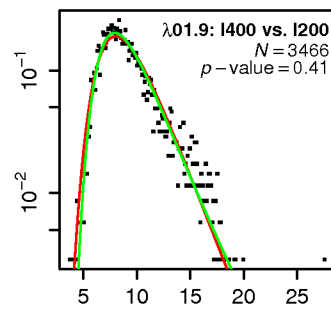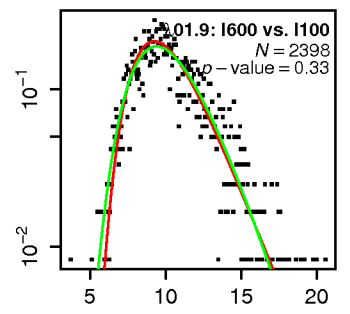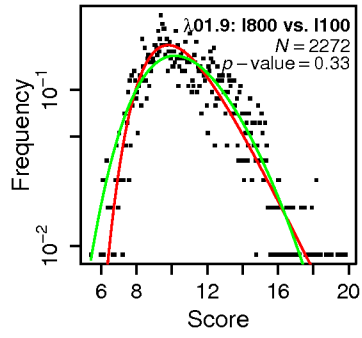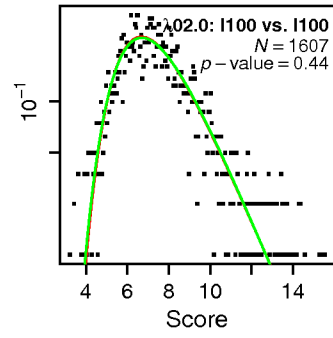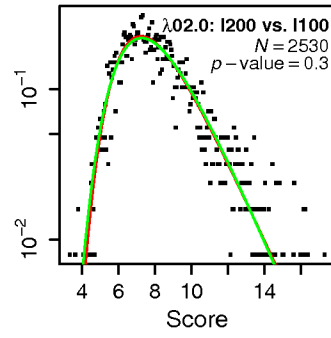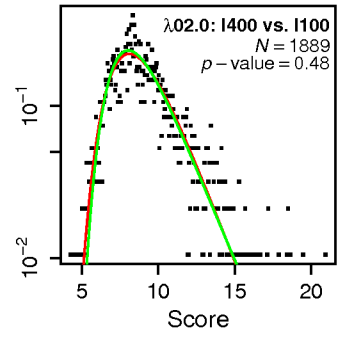

Supplement: Supplementary file 5 — Figure S9. Distributions of alignment scores obtained from aligning pairs of simulated profiles with mutual compositional similarity λ and different values of length l. (PDF 1,872 kb) [file 12859_2019_2913_MOESM5_ESM.pdf]

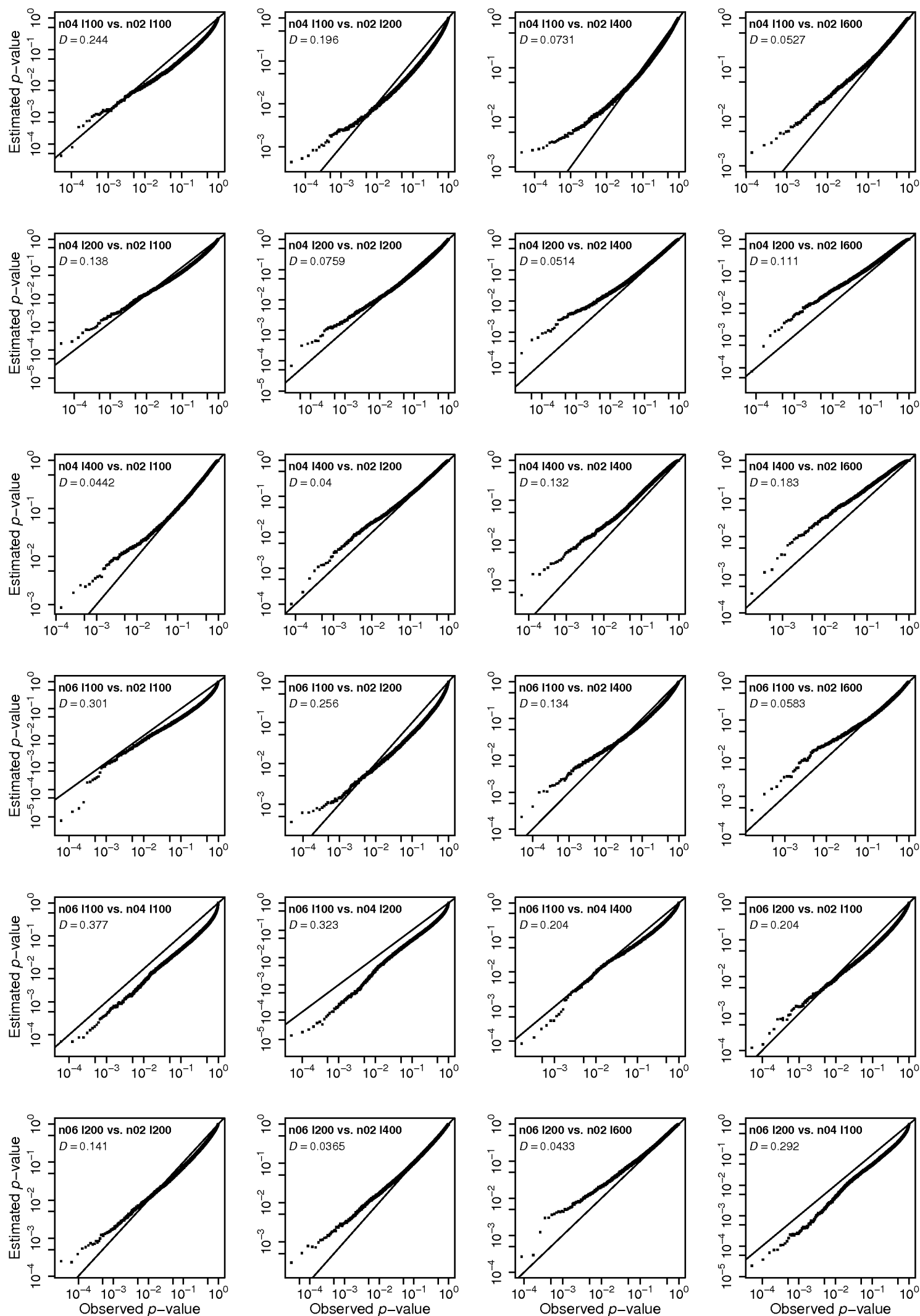

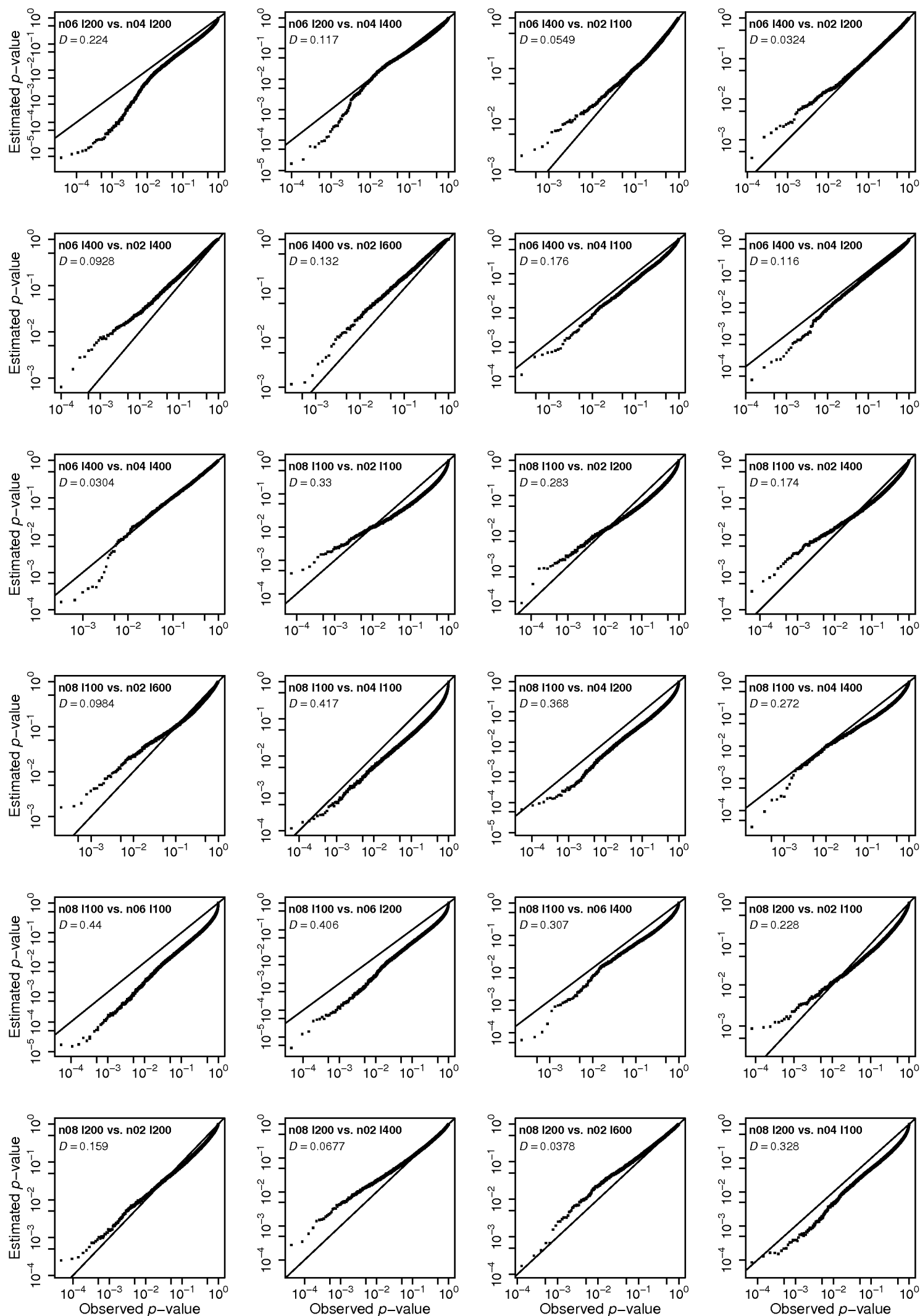

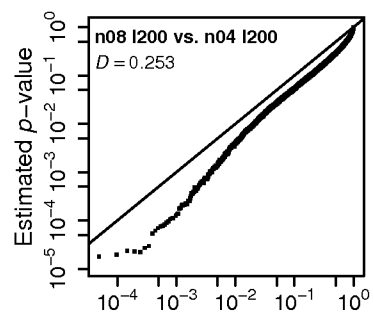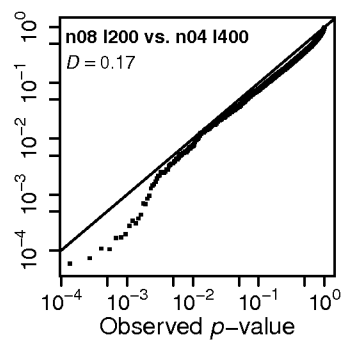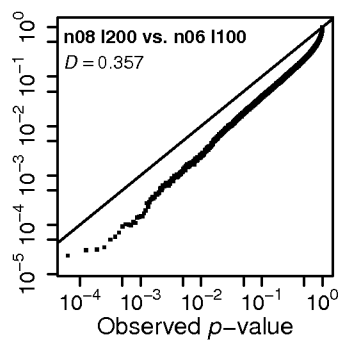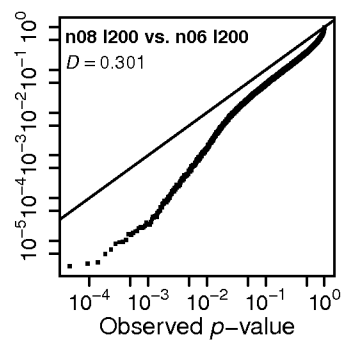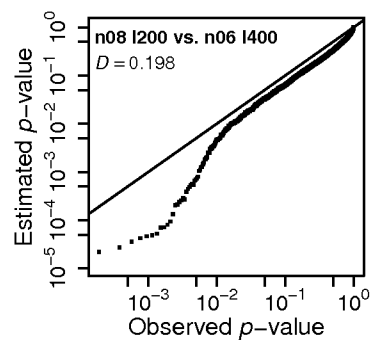

Supplement: Supplementary file 6 — Figure S10. Observed p-values corresponding to the empirical distribution function obtained for real unrelated profiles against estimated p-values using predicted values of the statistical parameters. (PDF 322 kb) [file 12859_2019_2913_MOESM6_ESM.pdf]

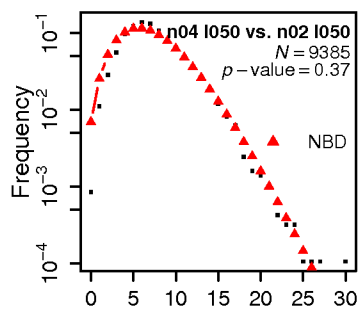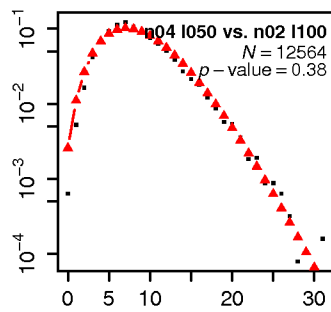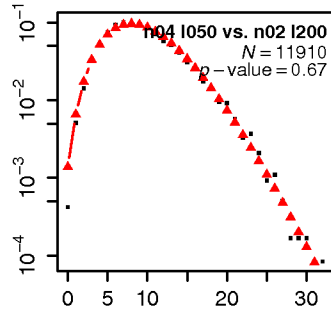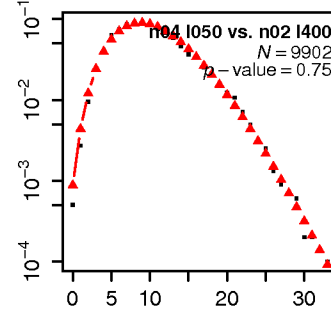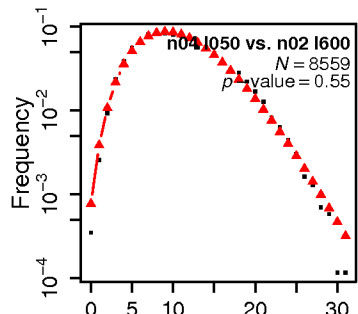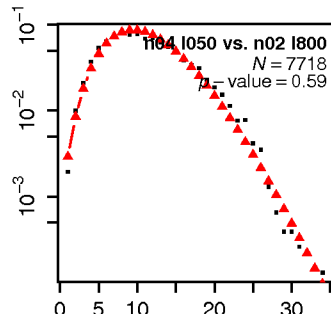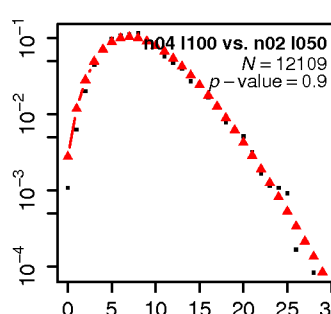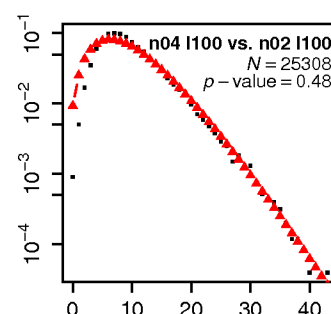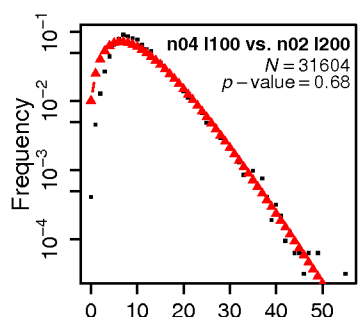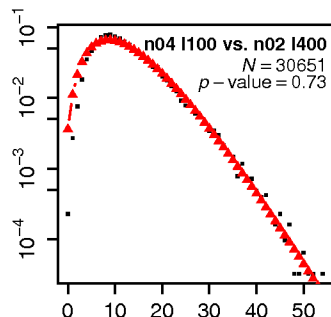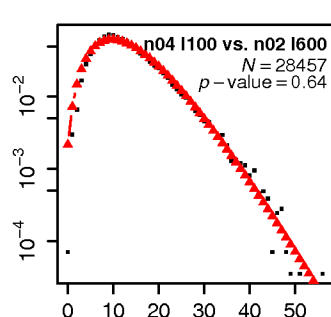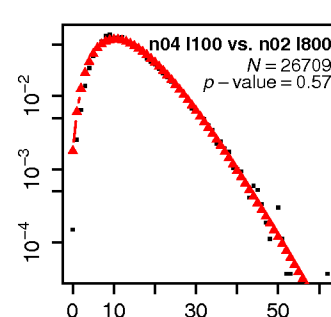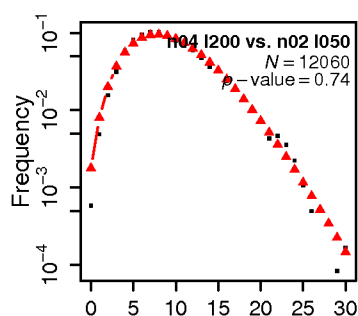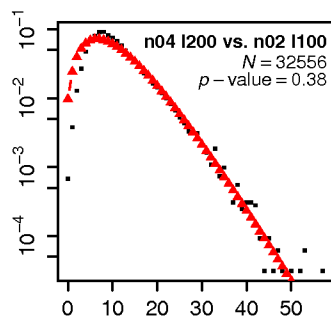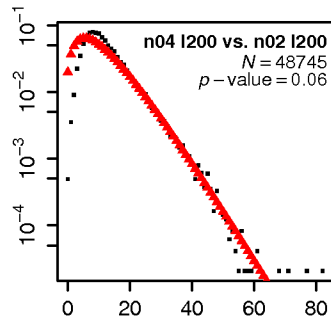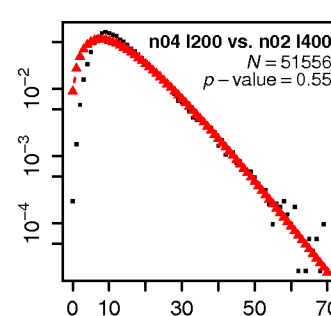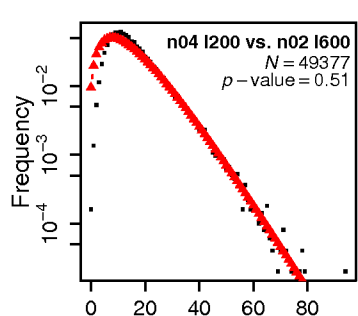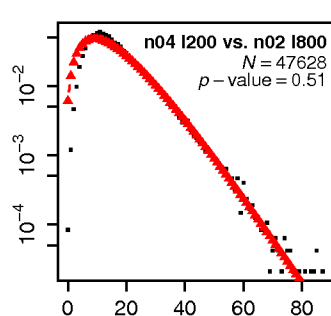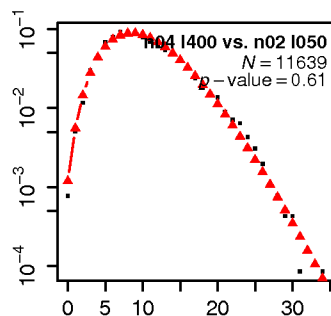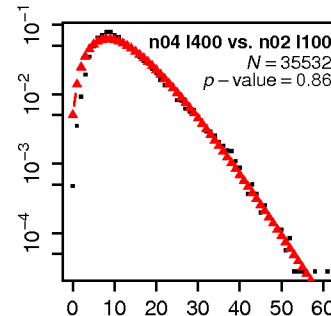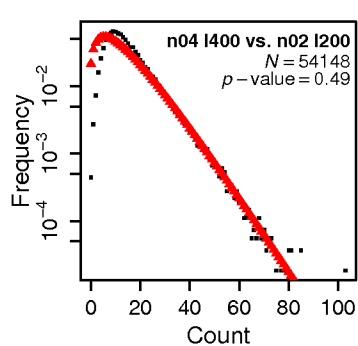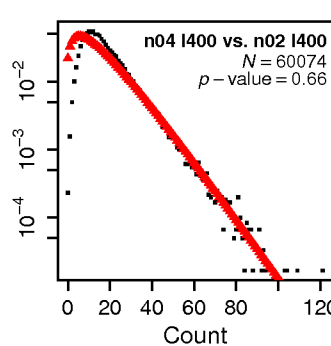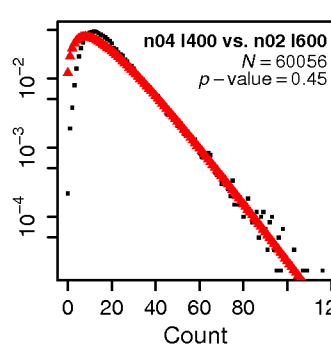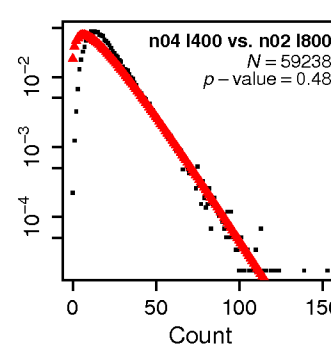

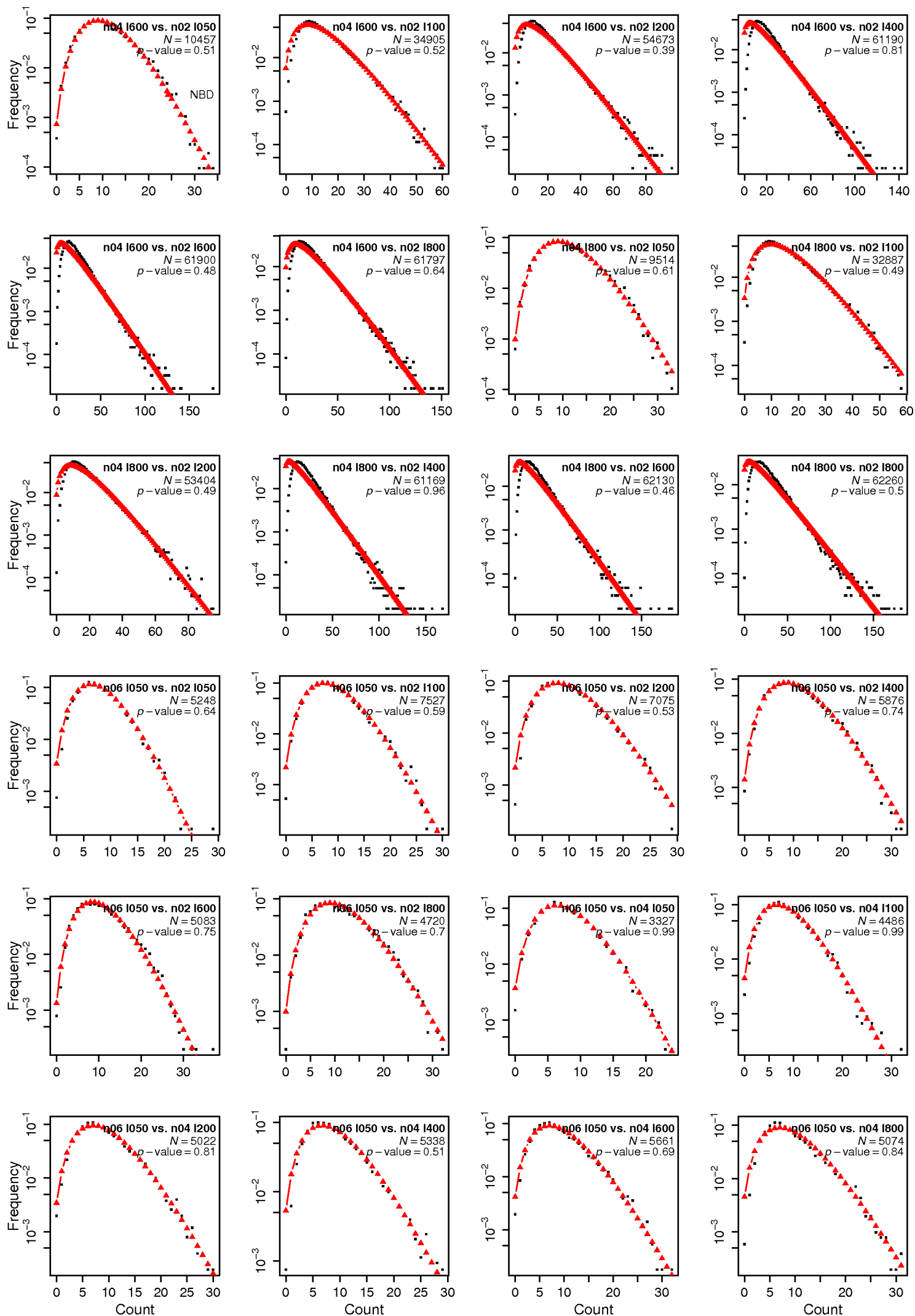

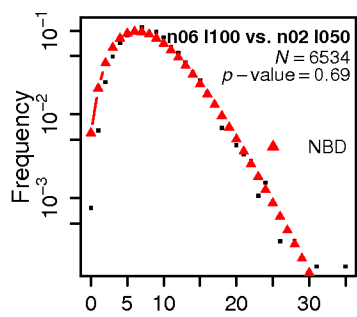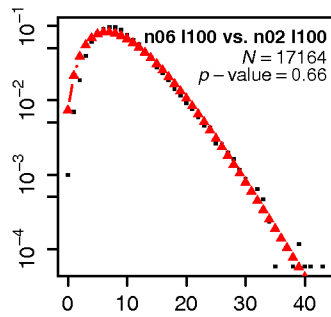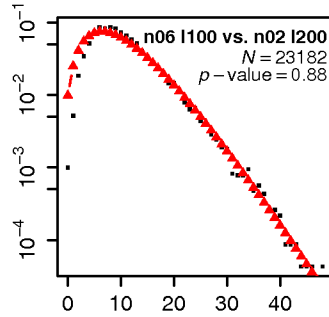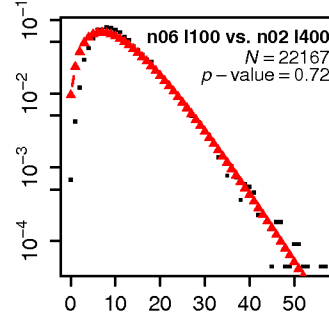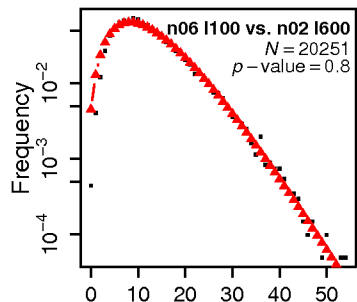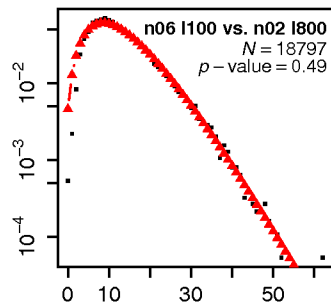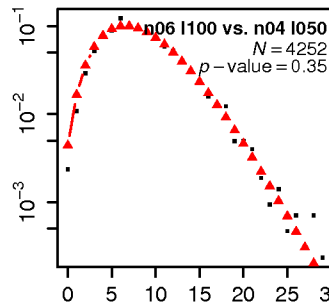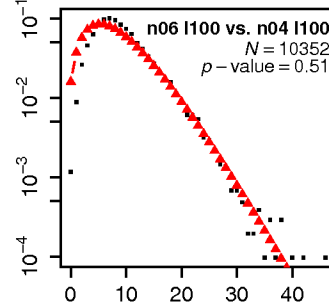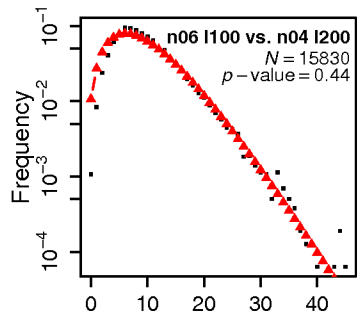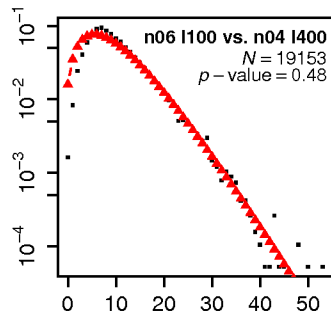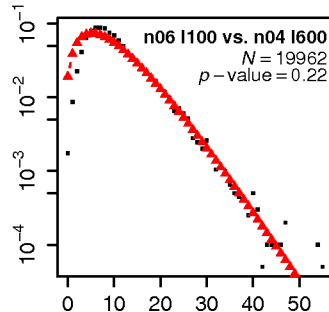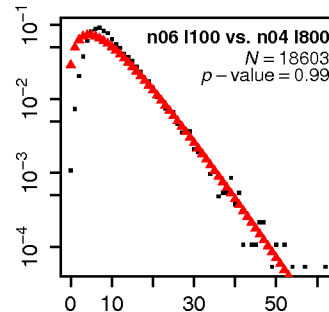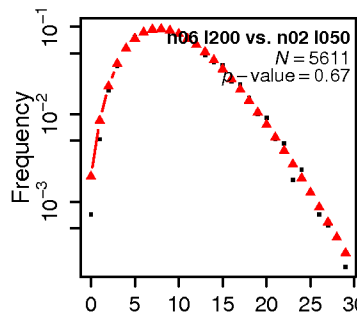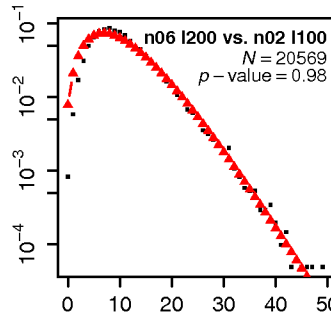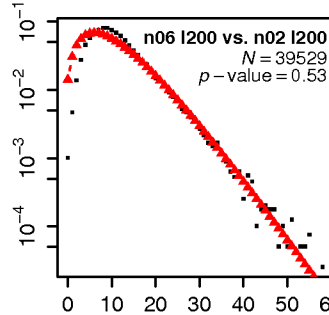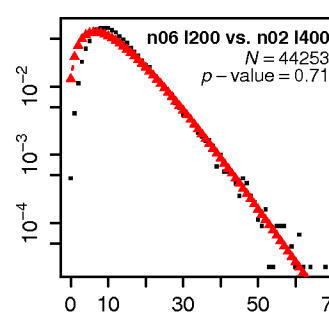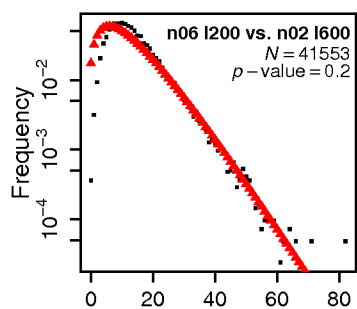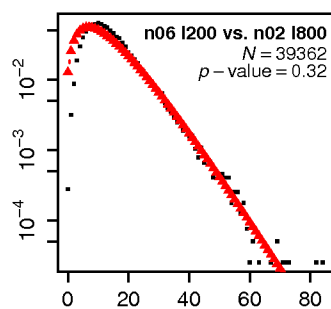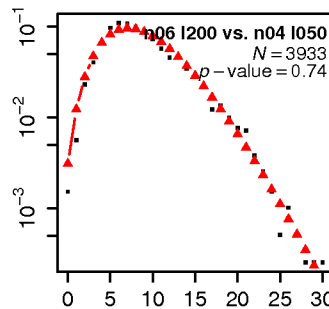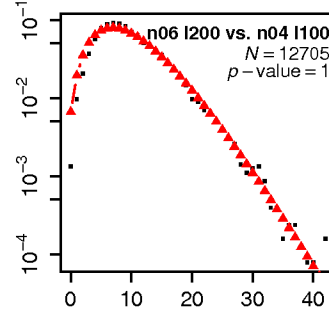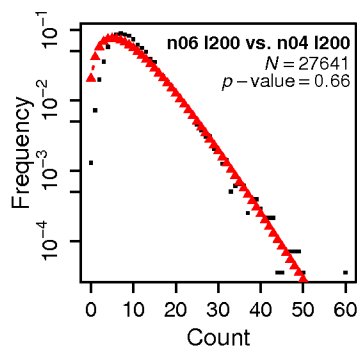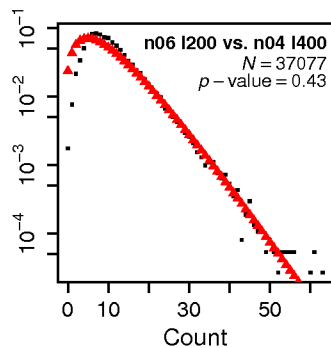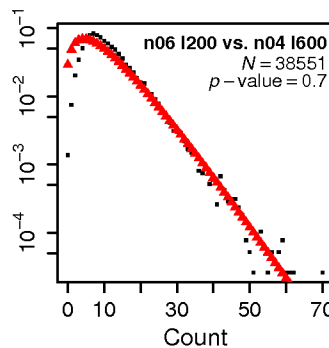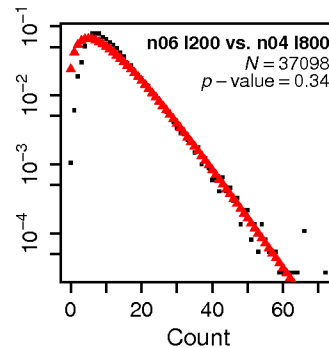

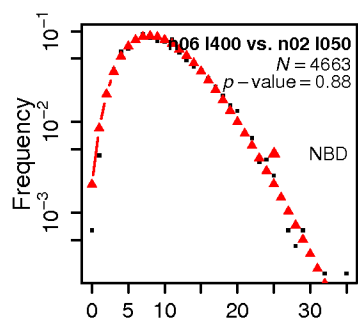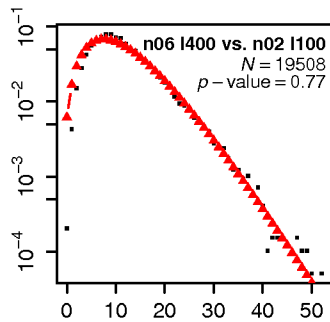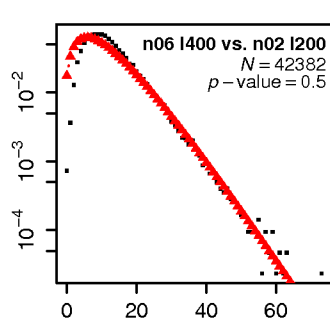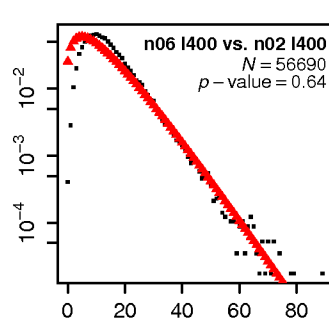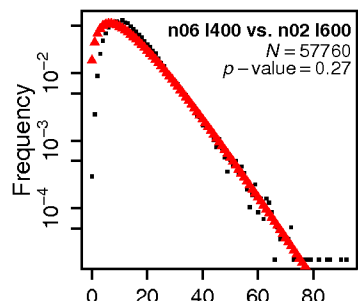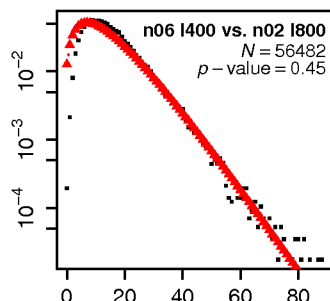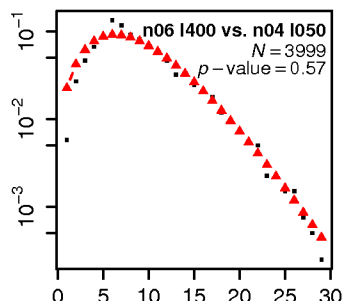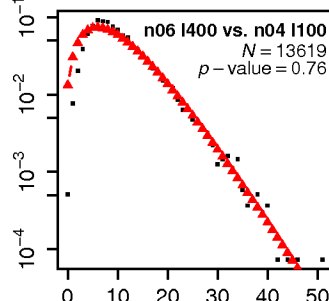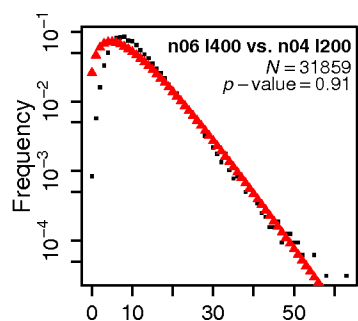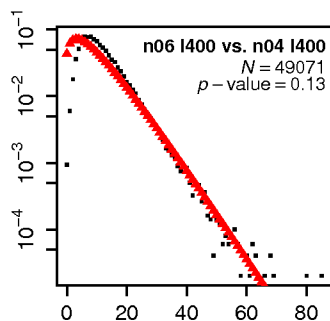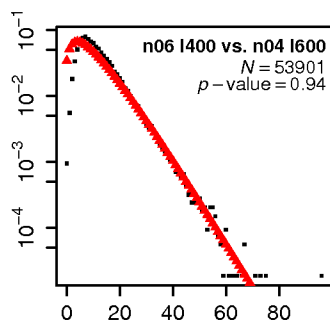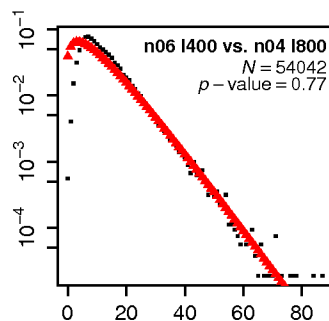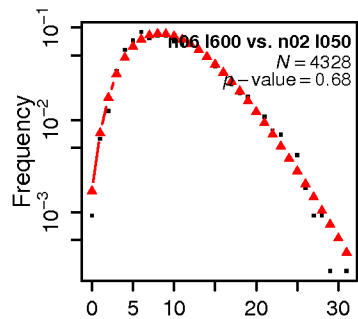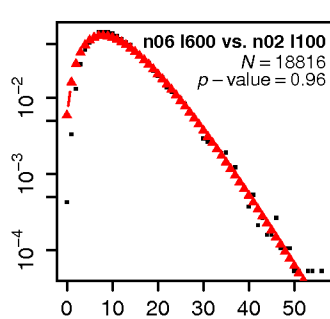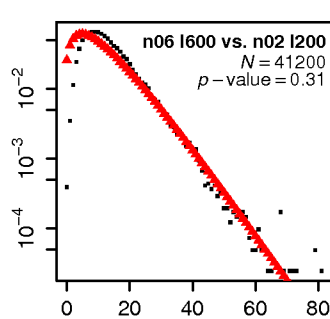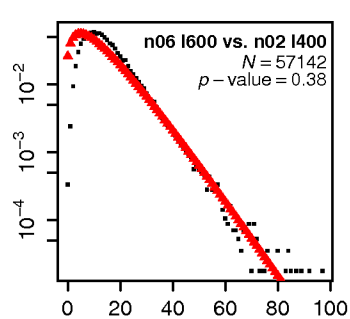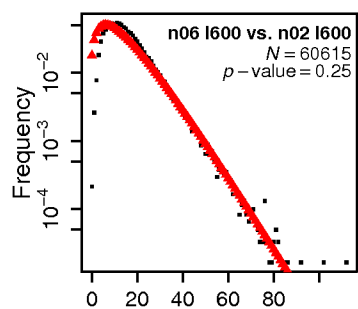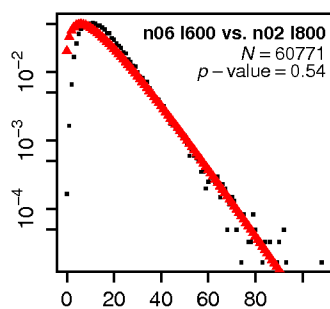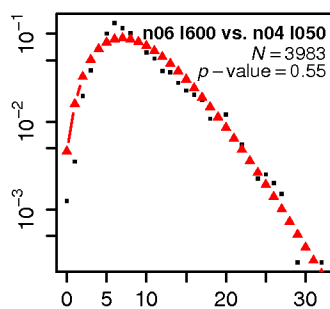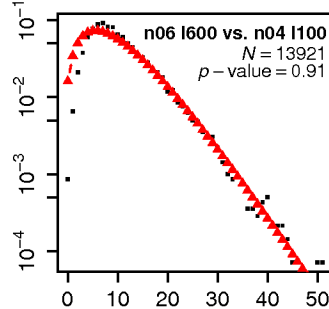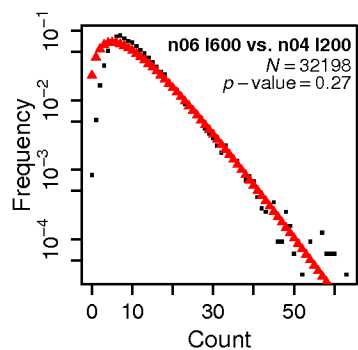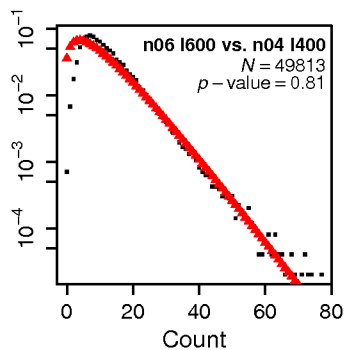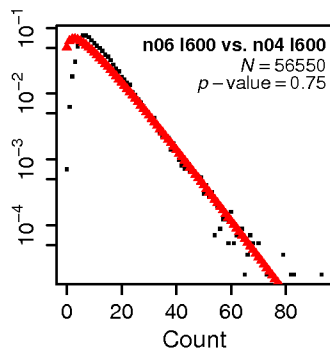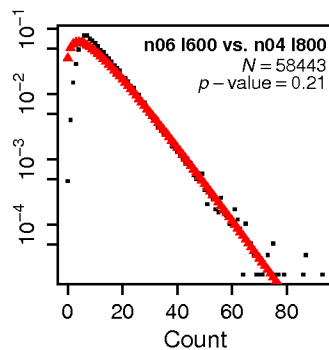

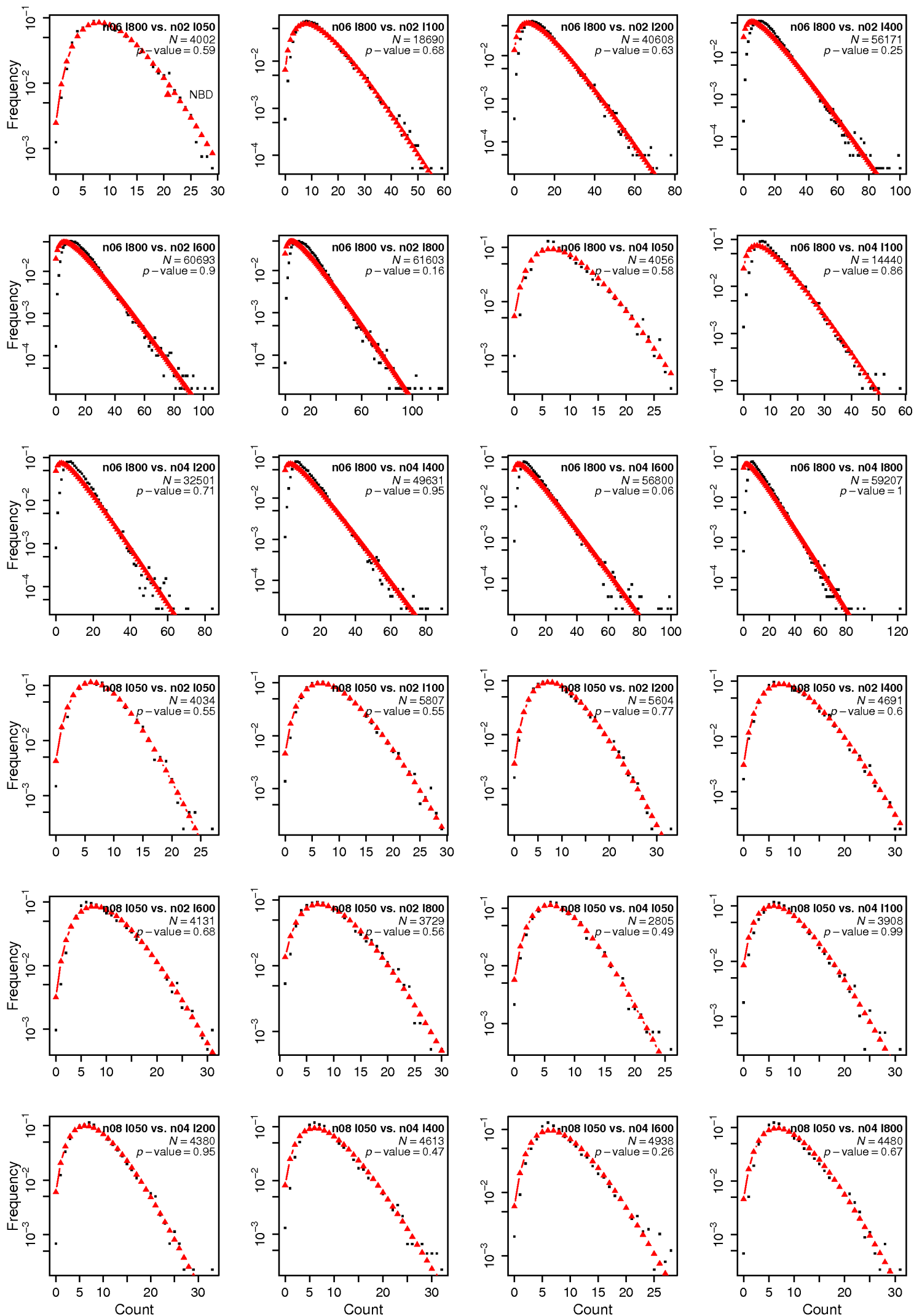

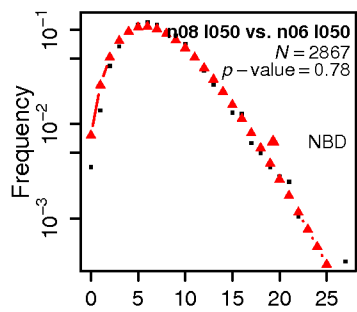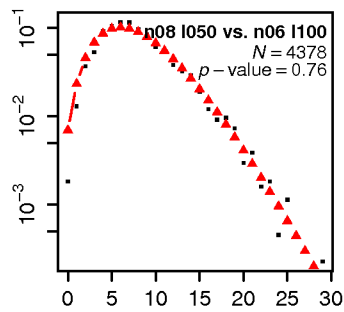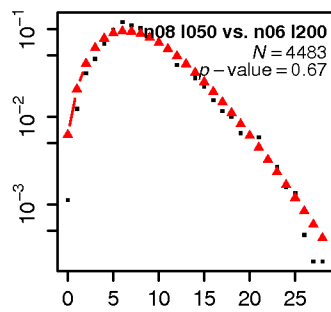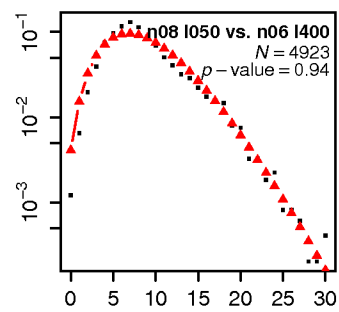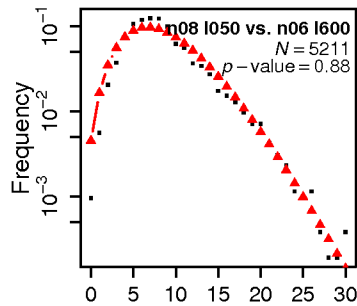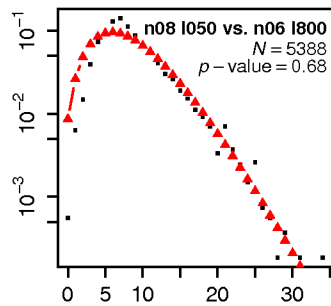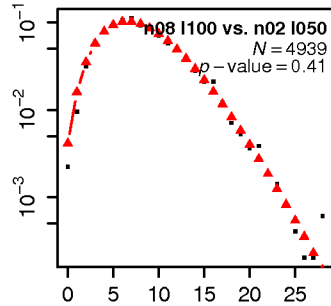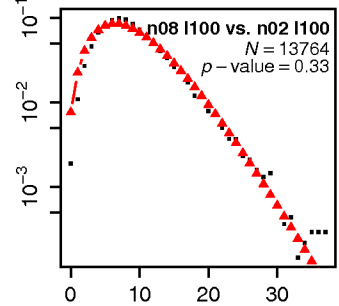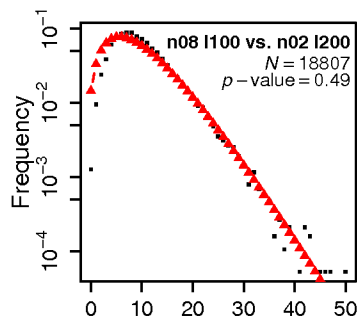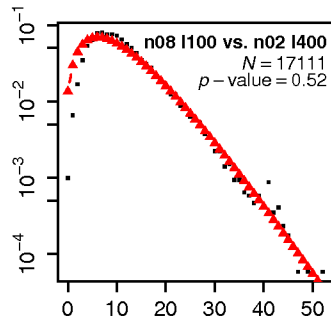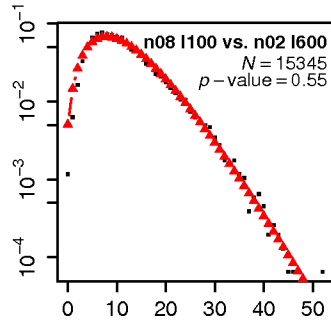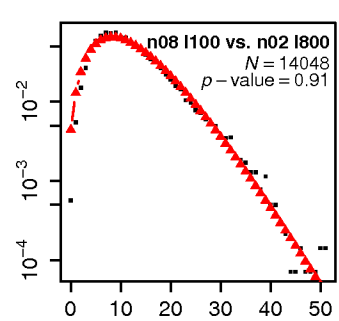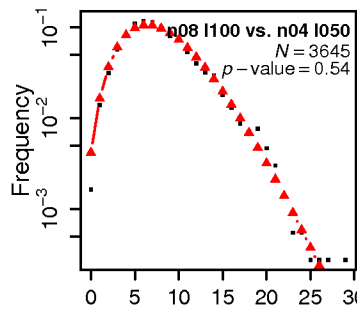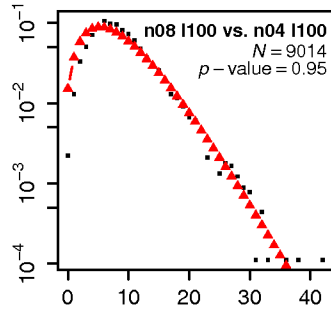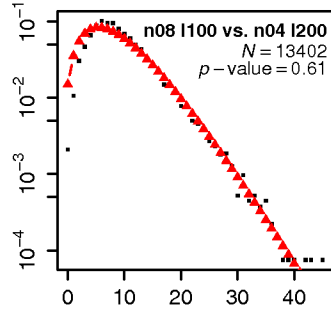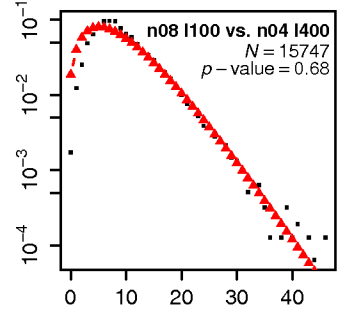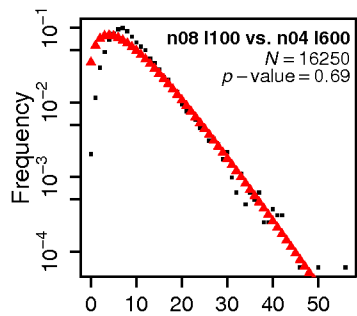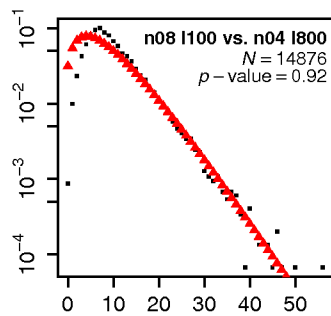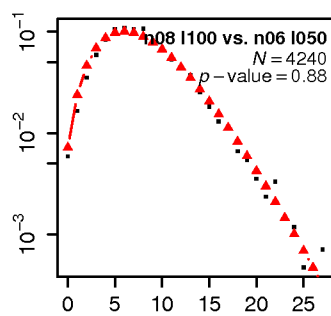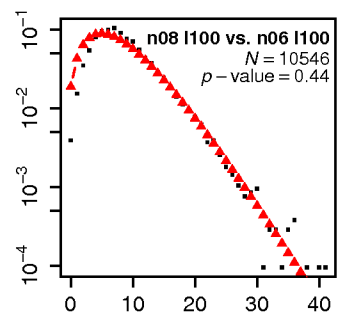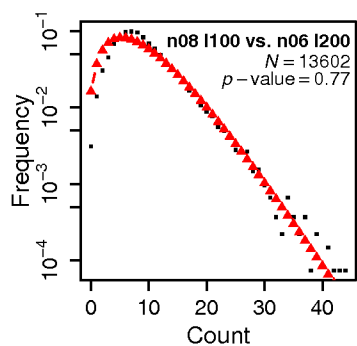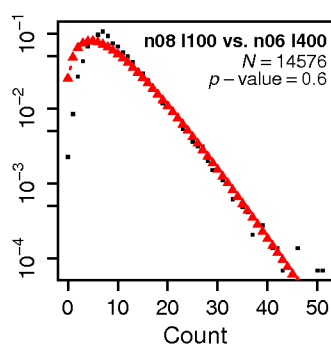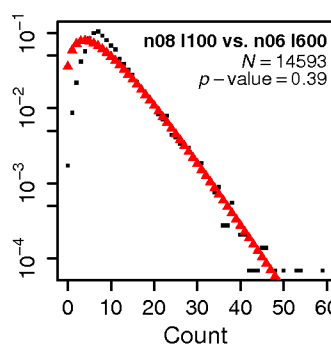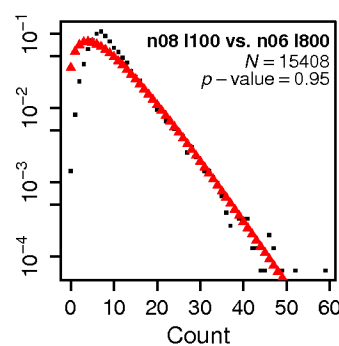

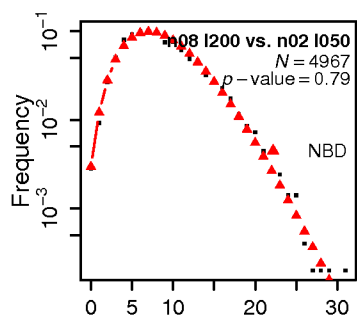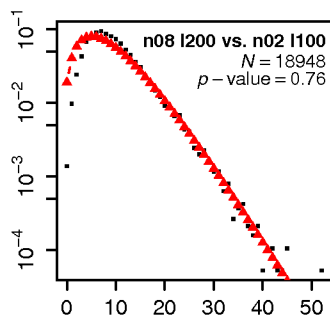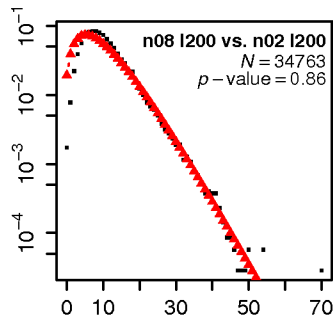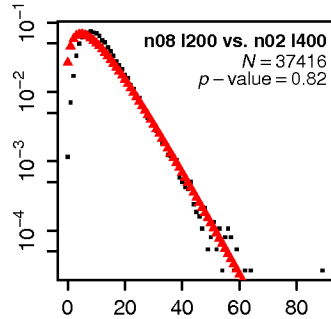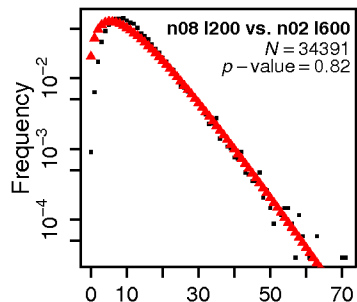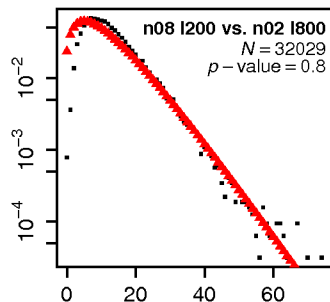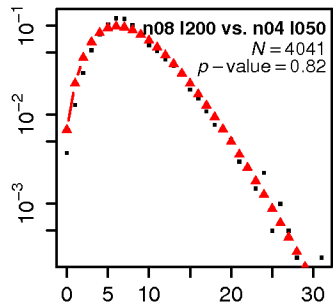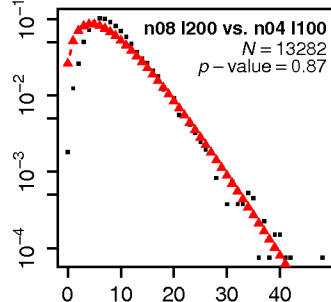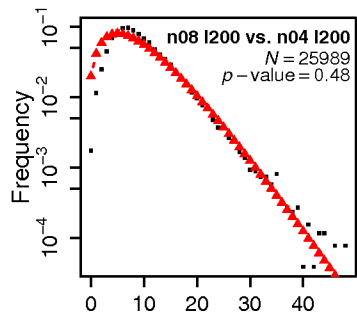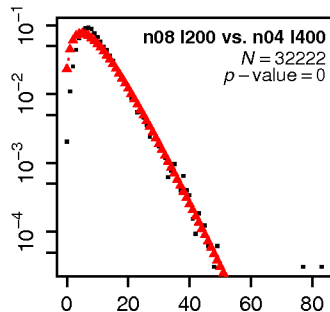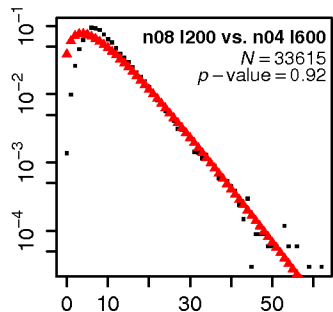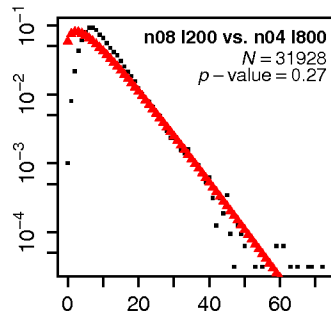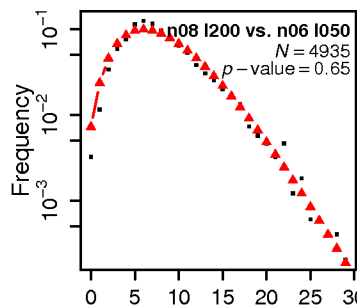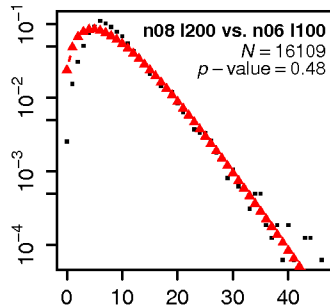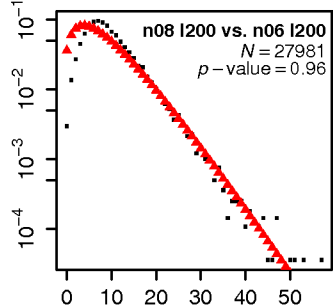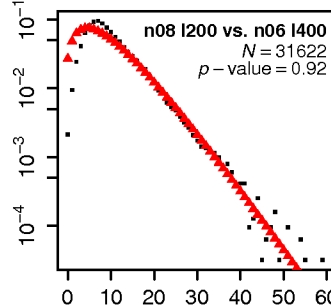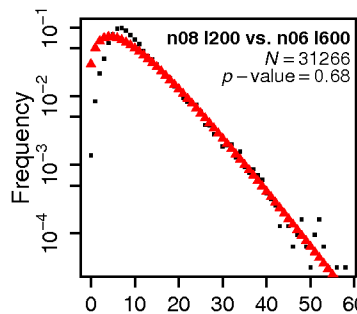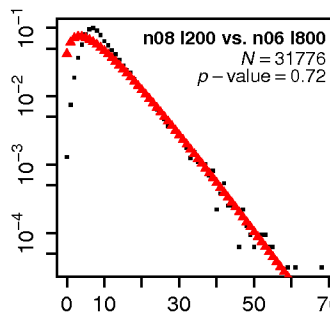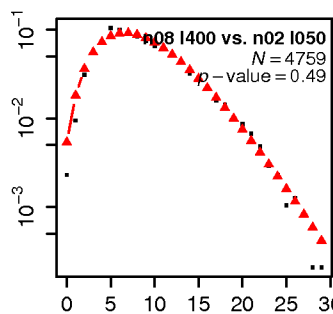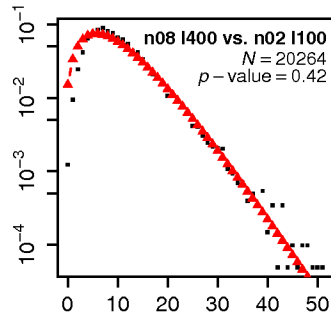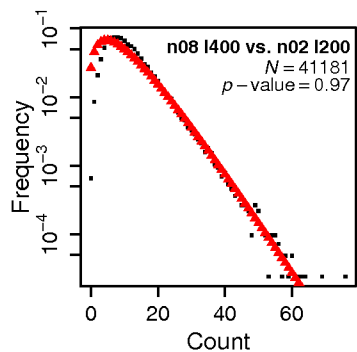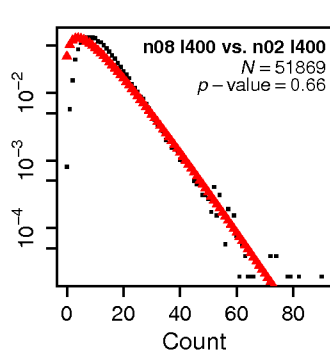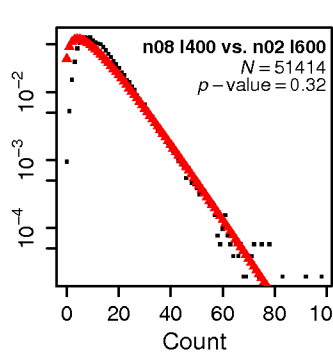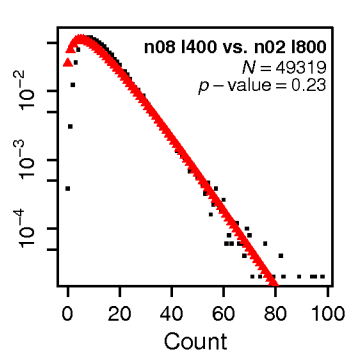

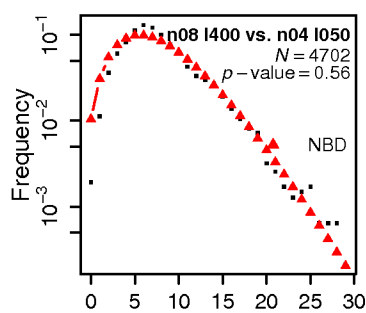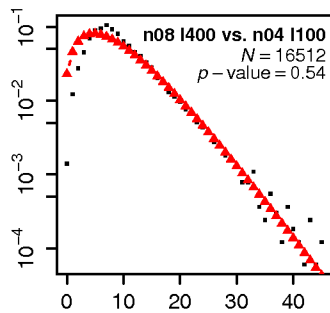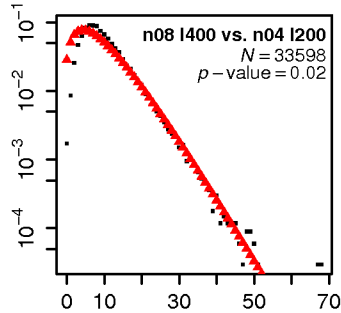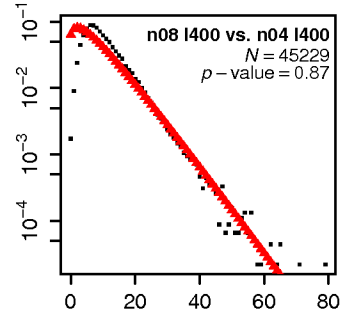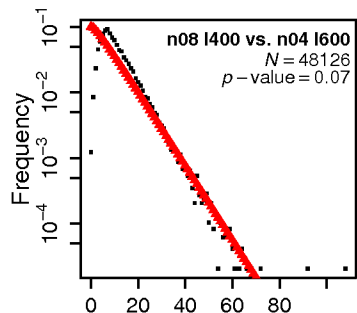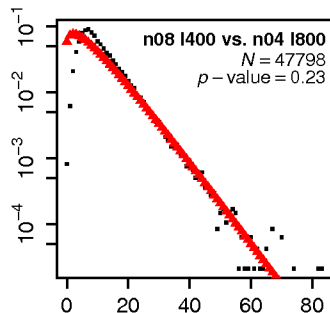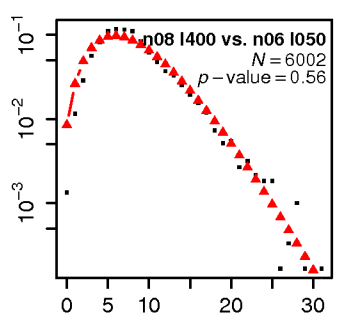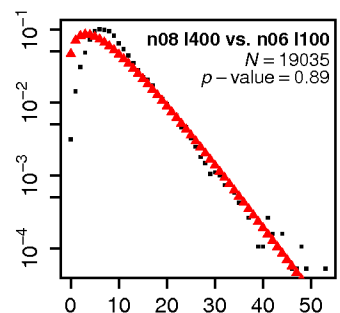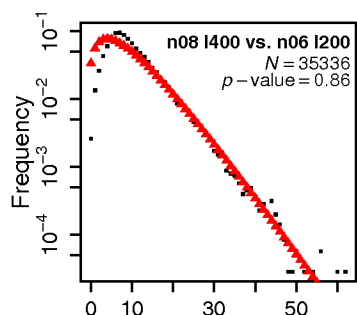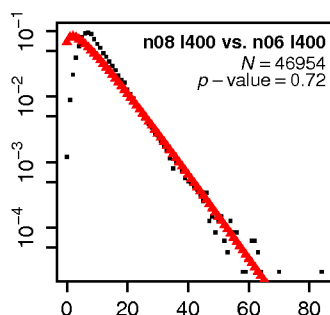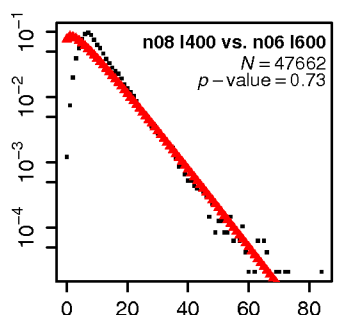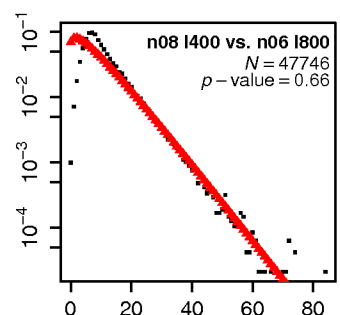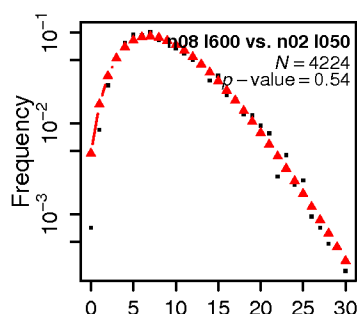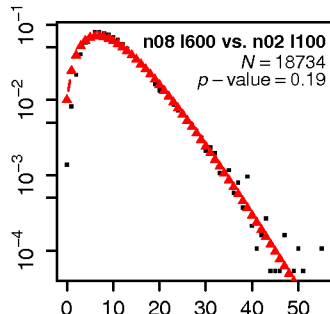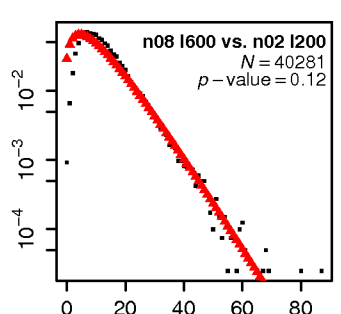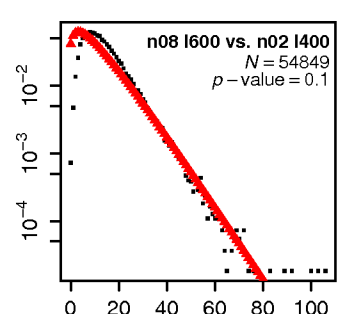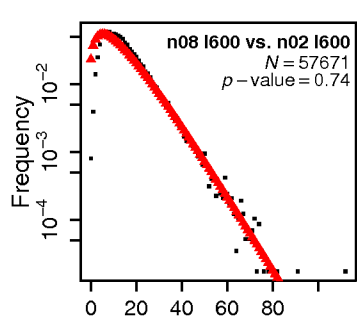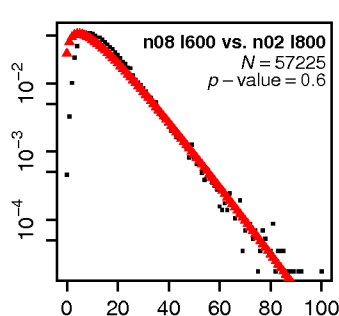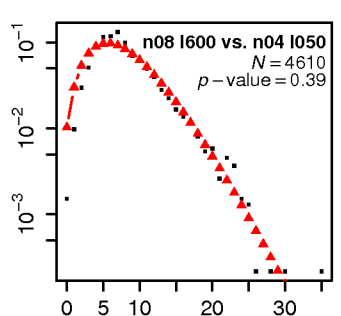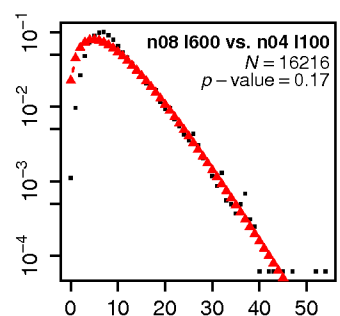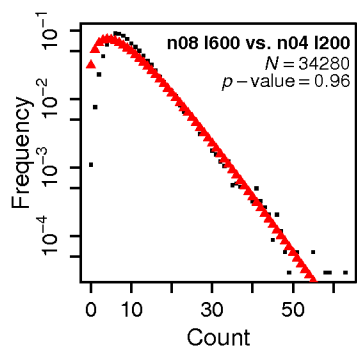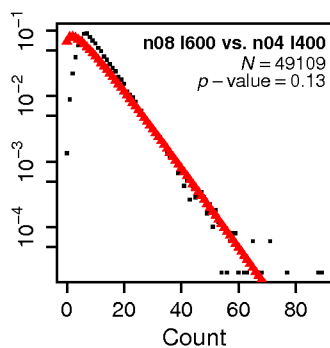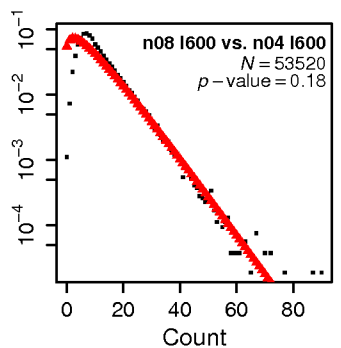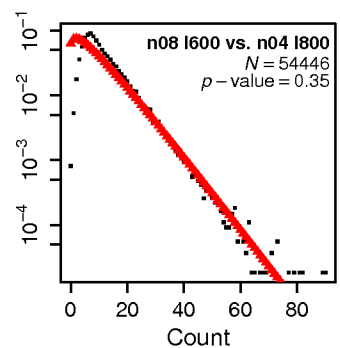

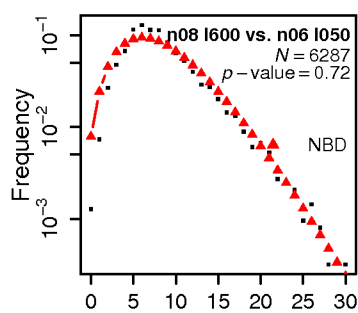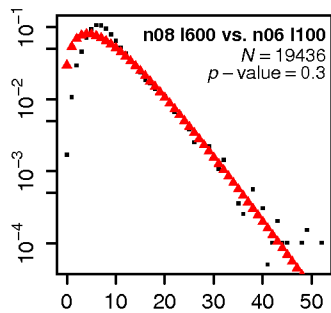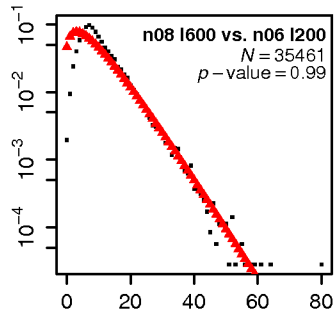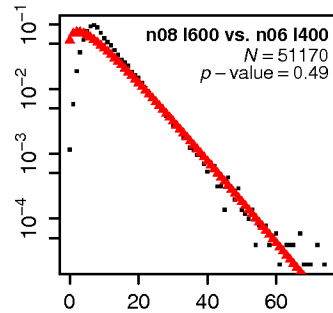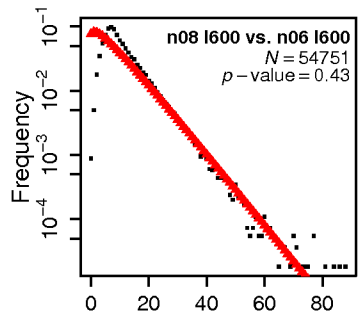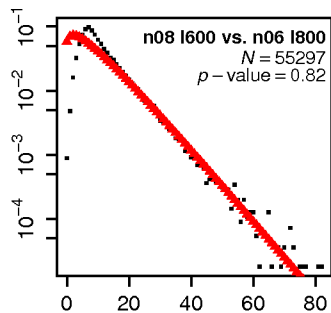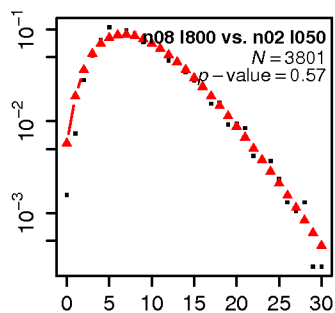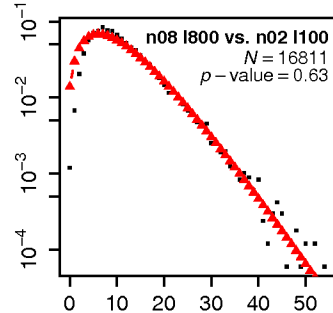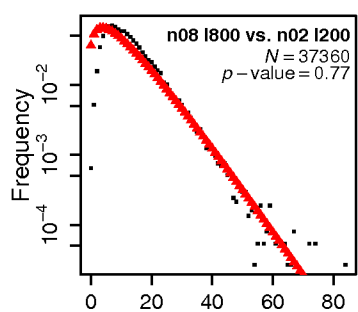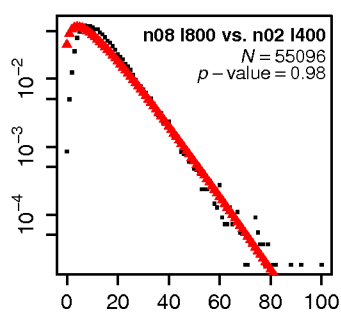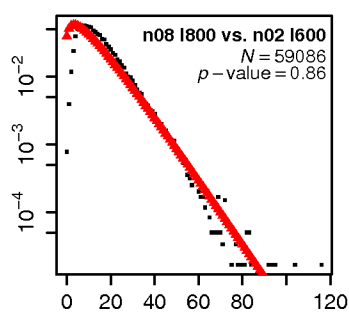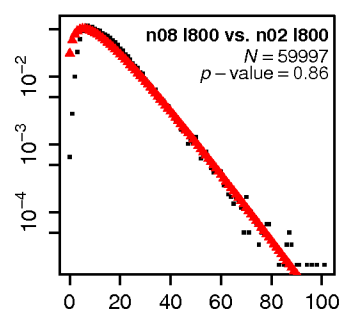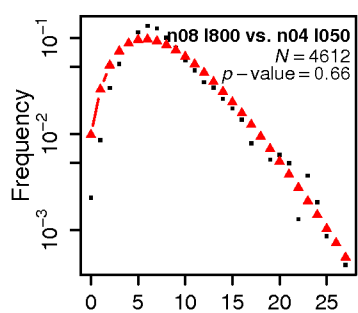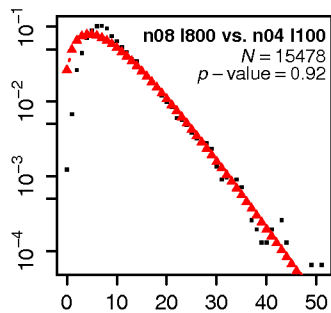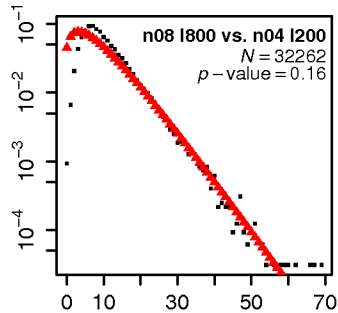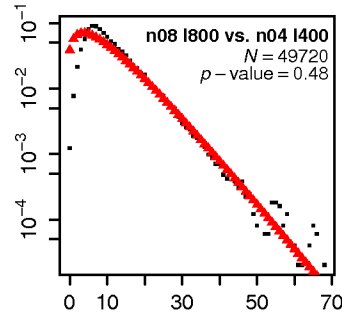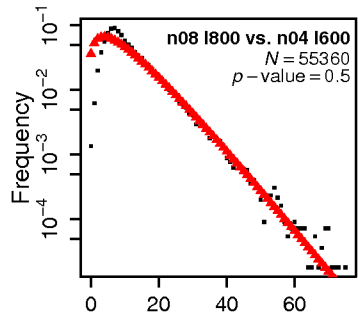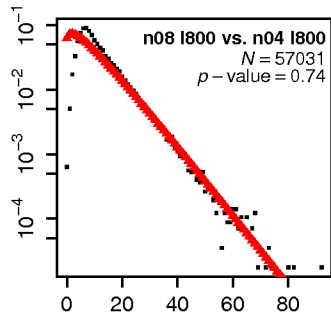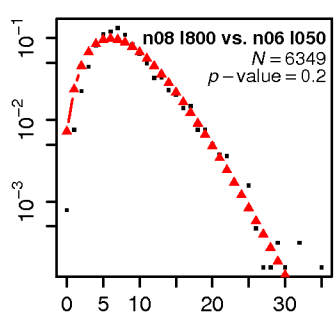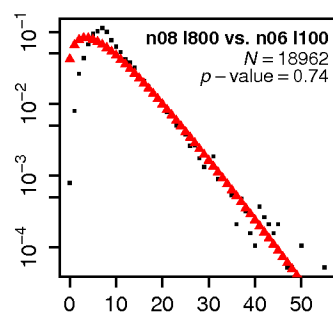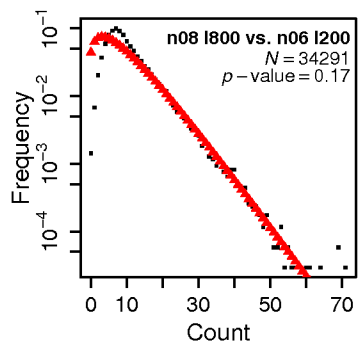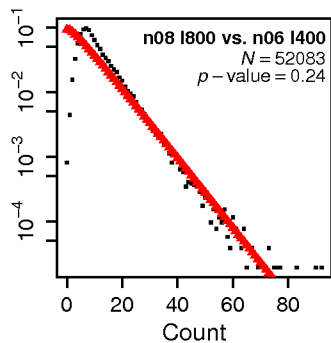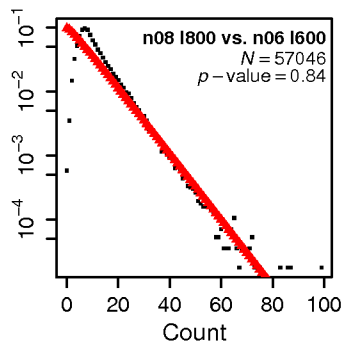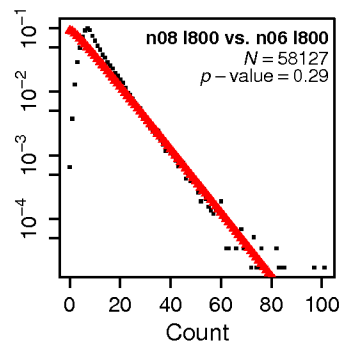

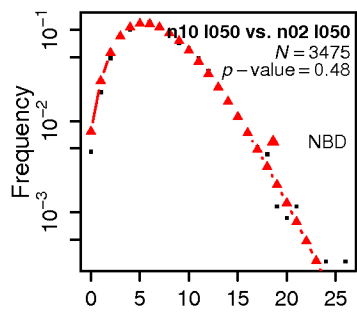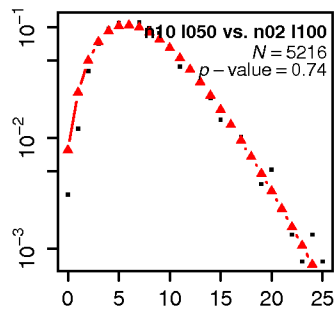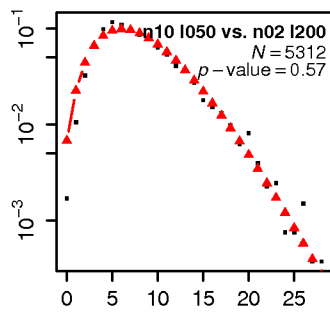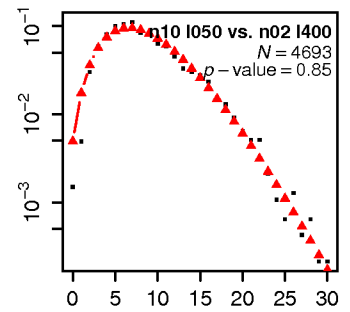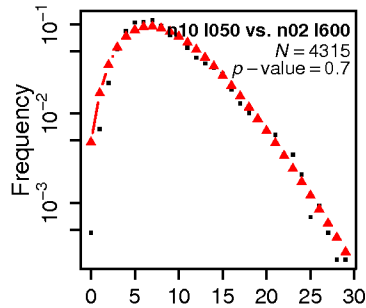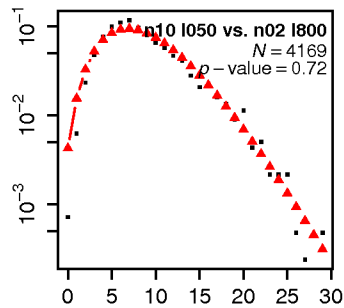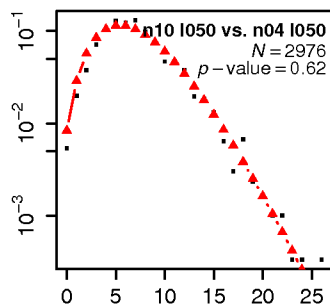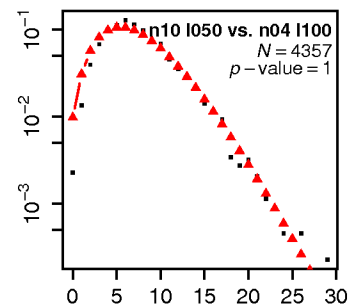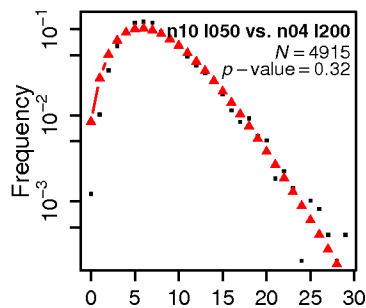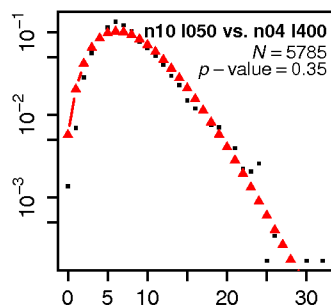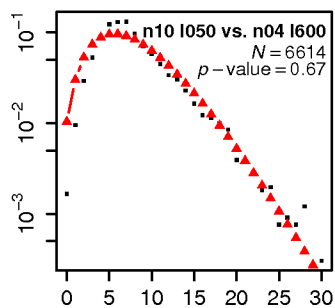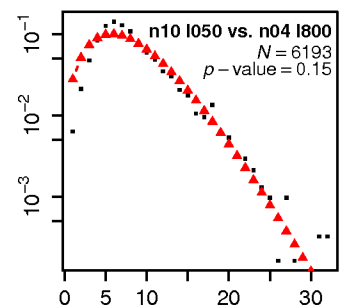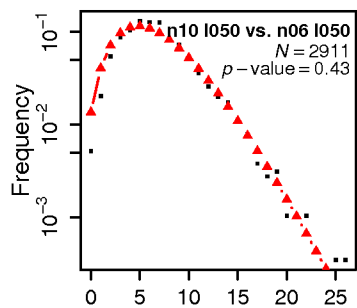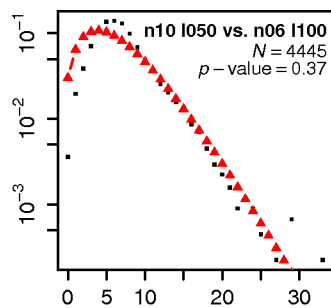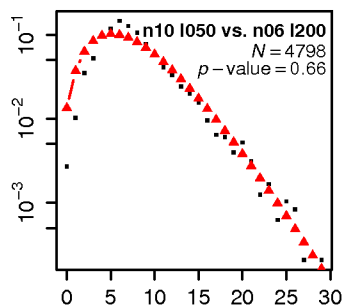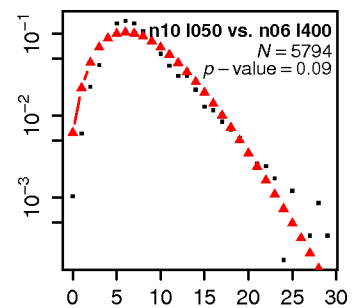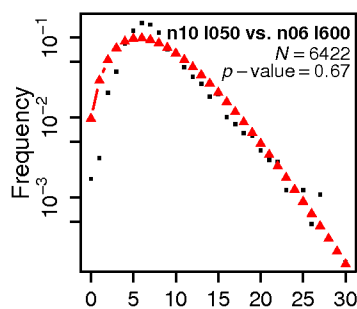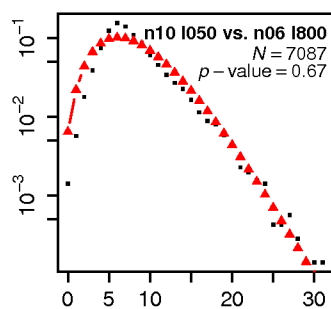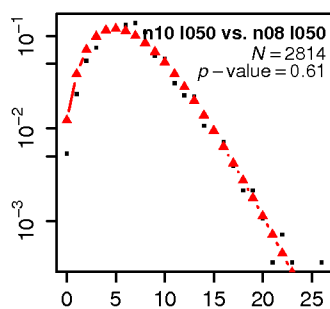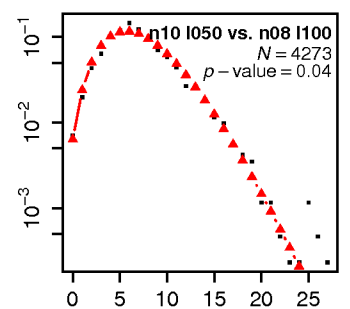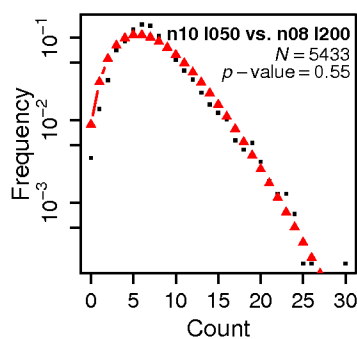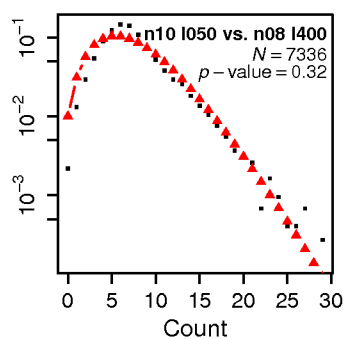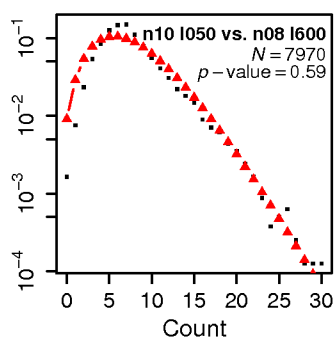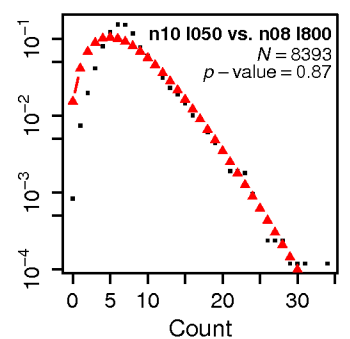

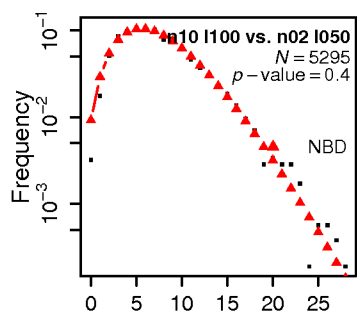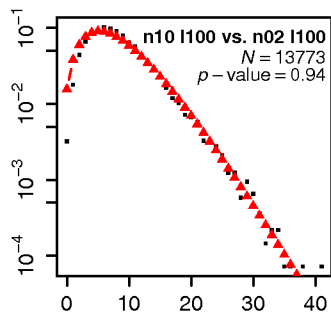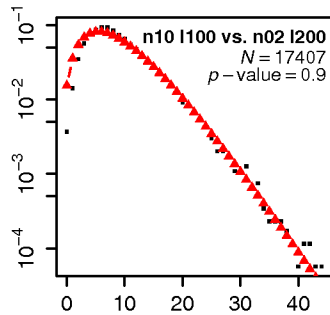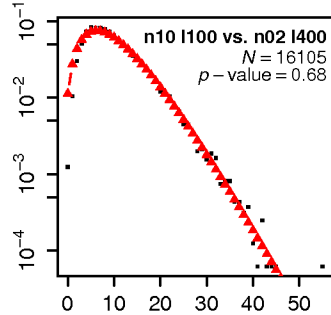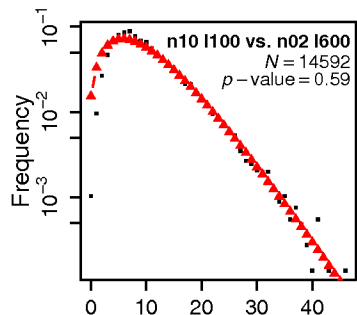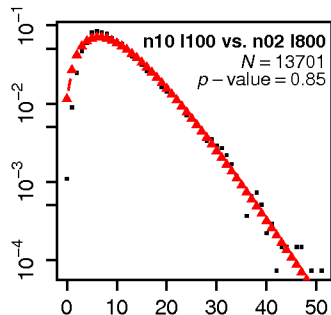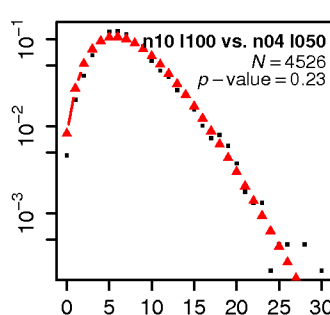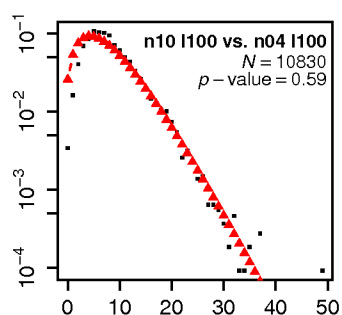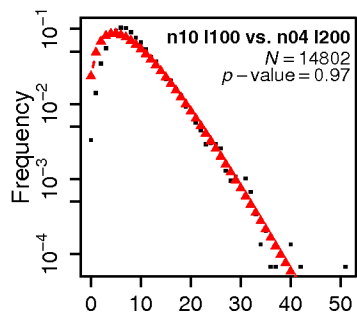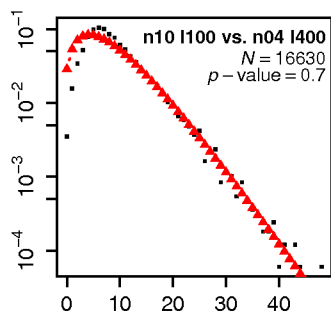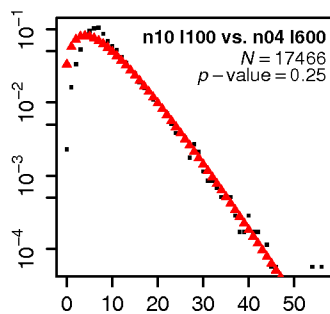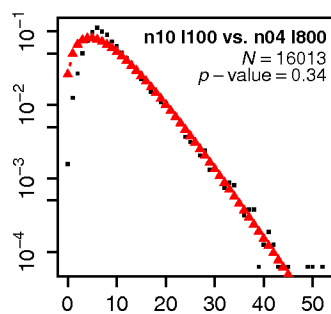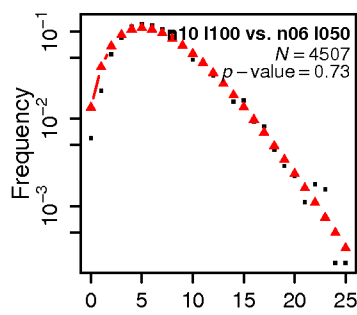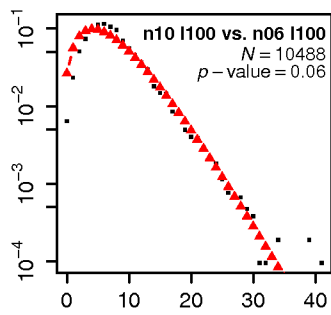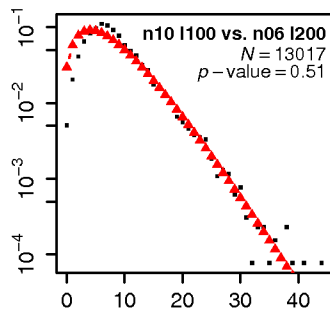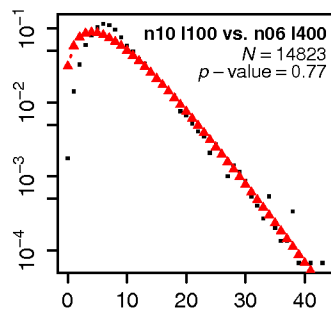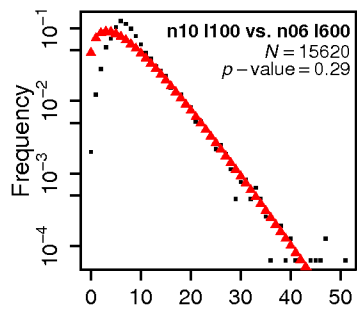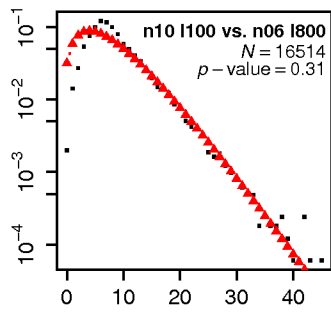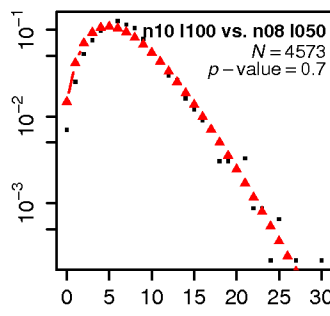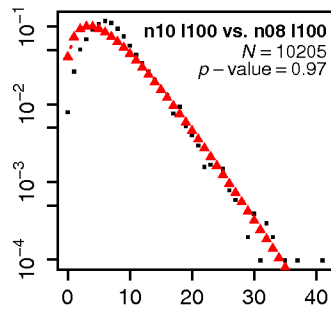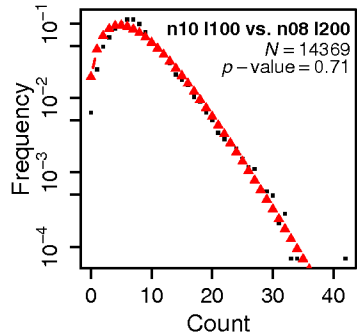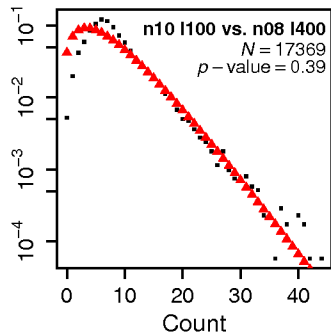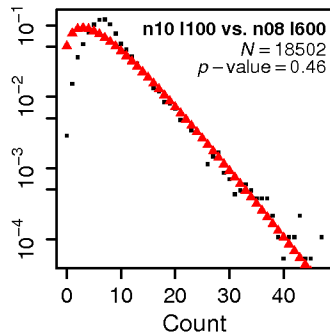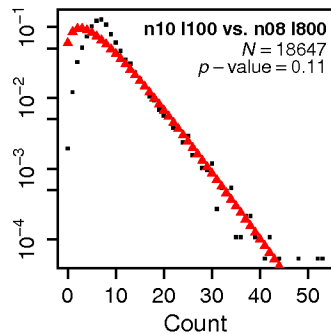

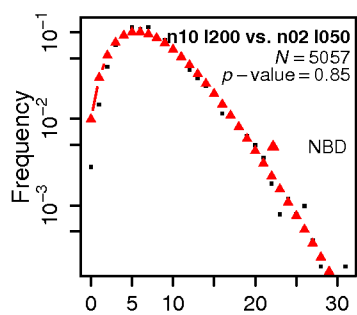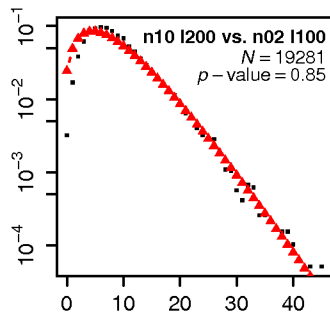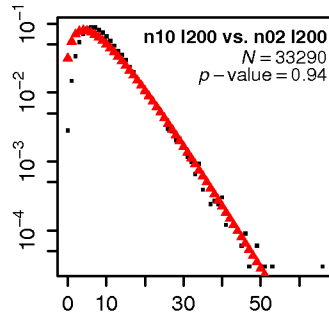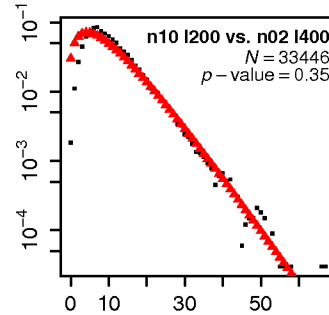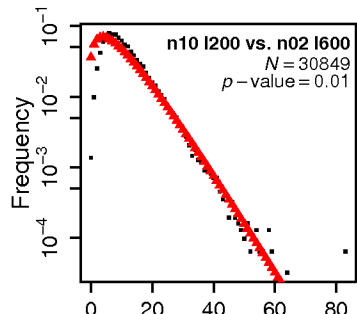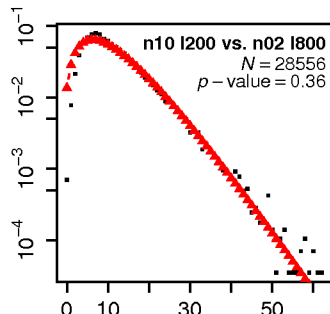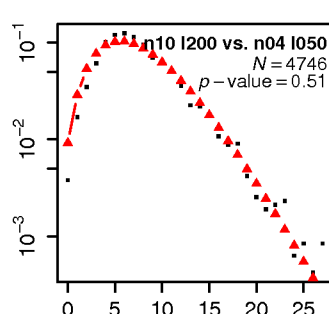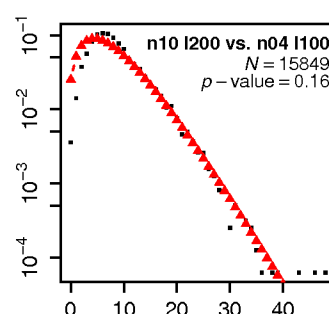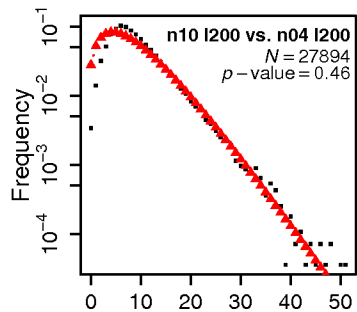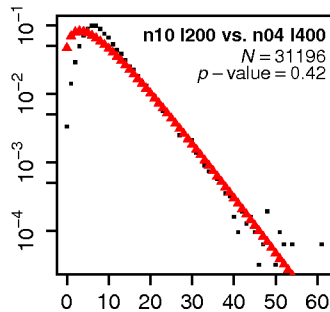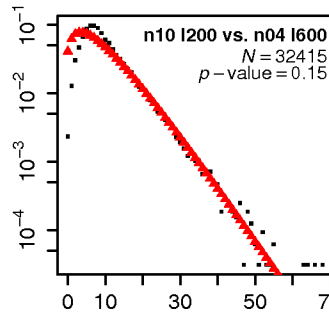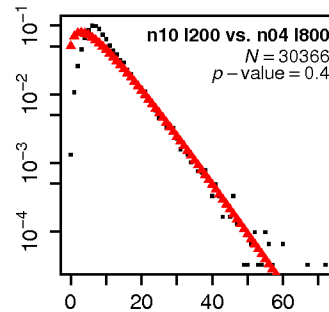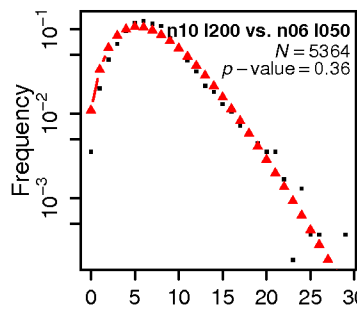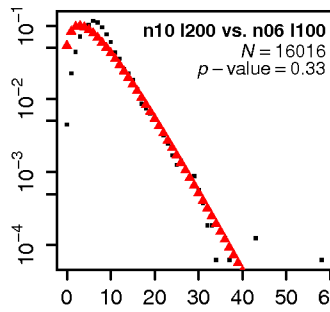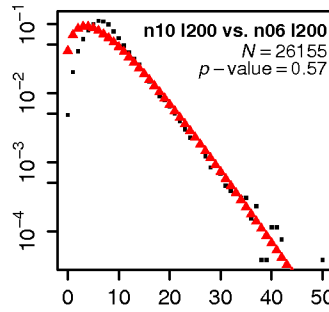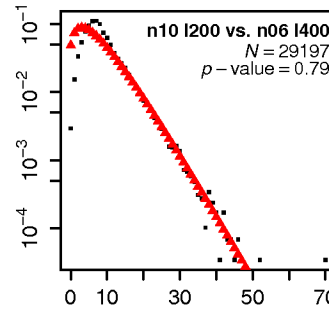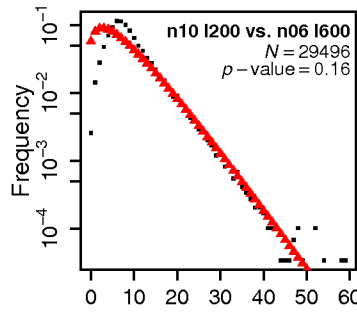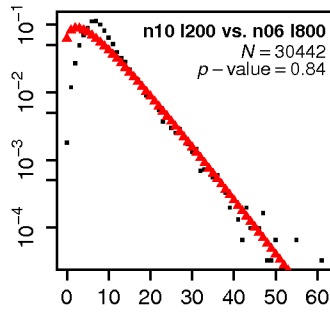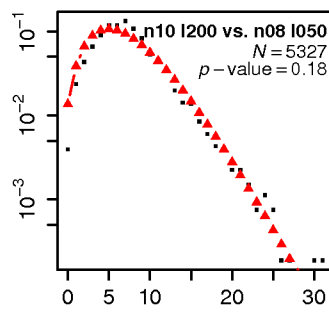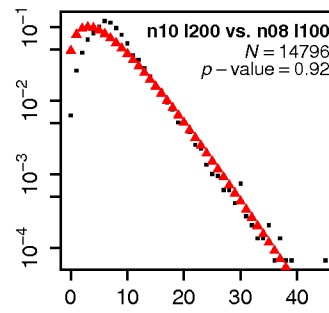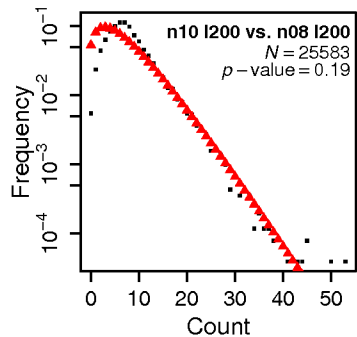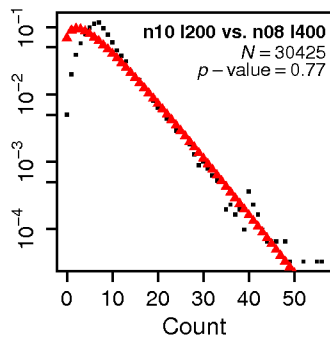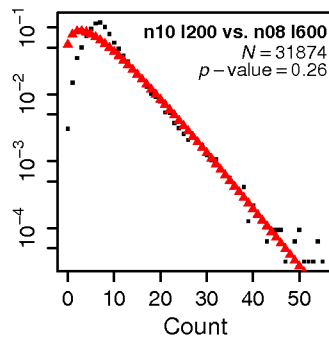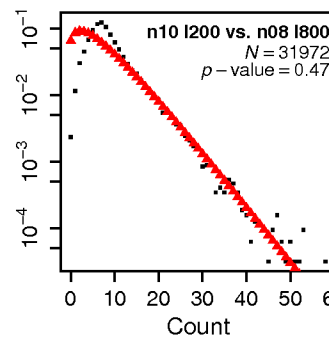

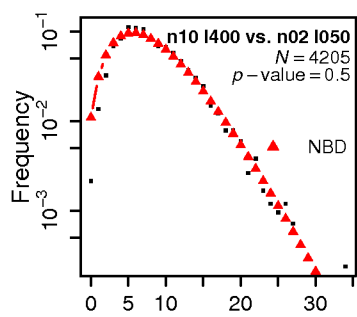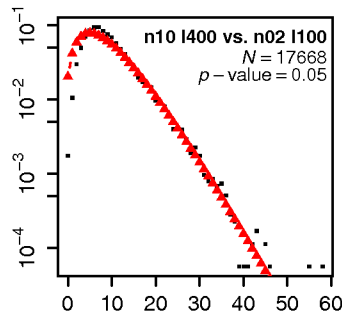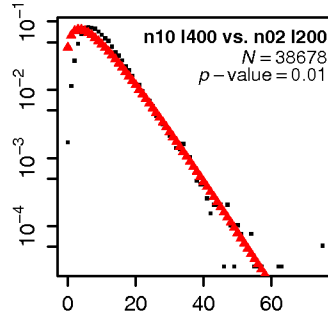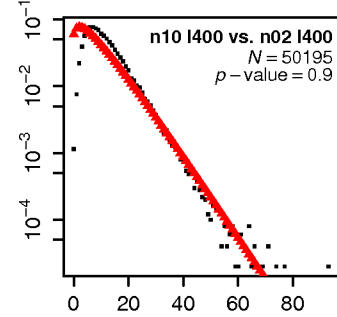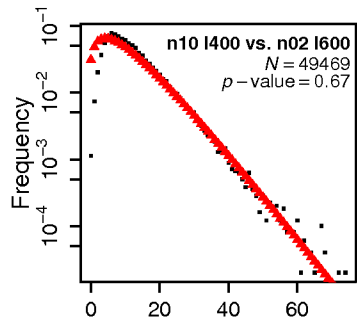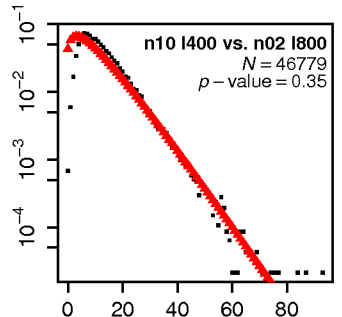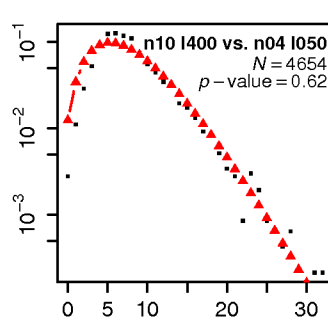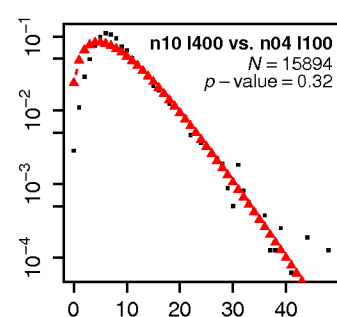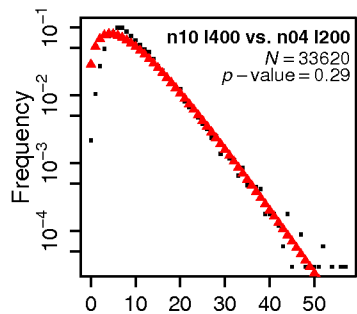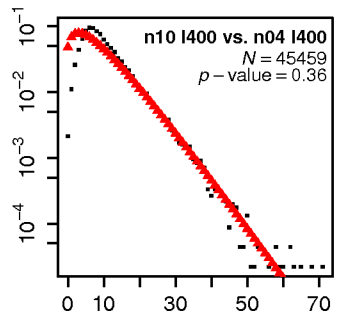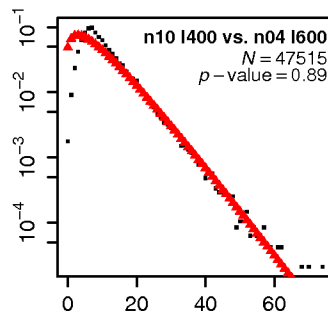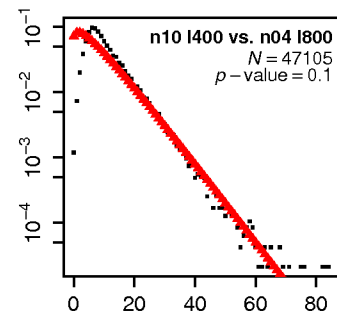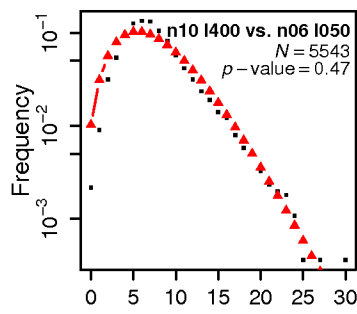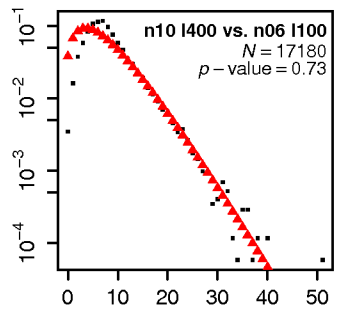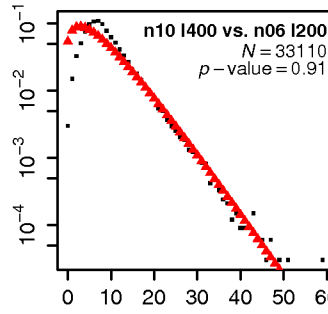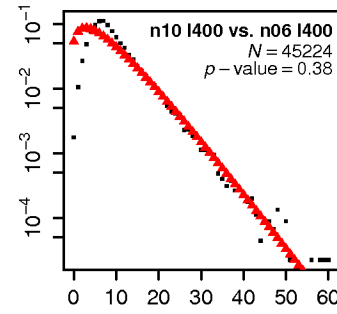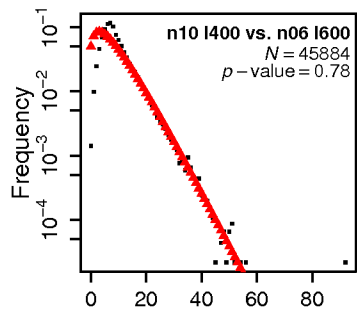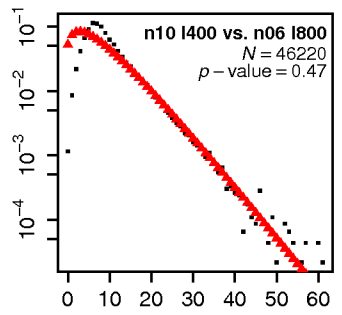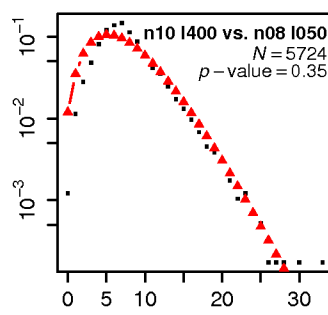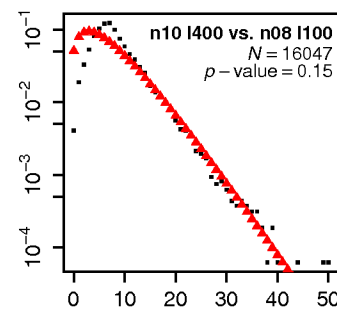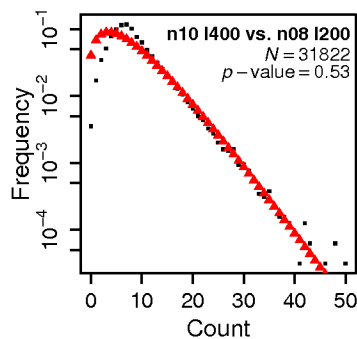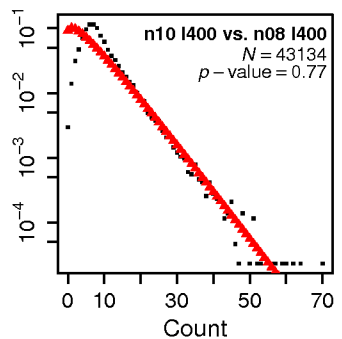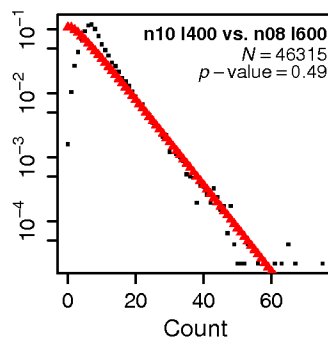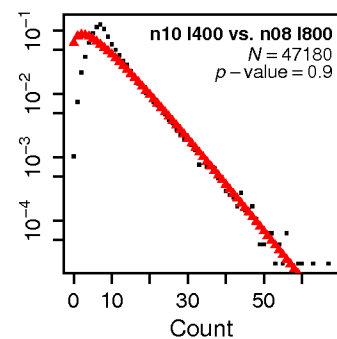

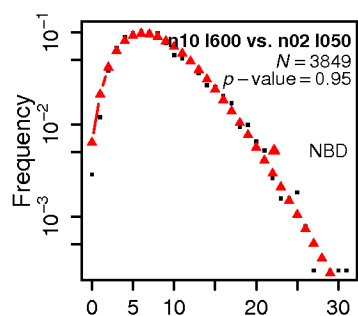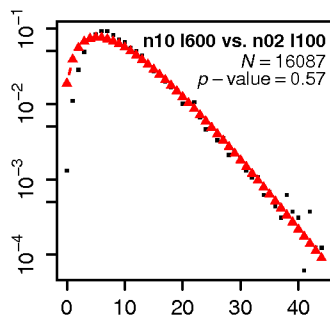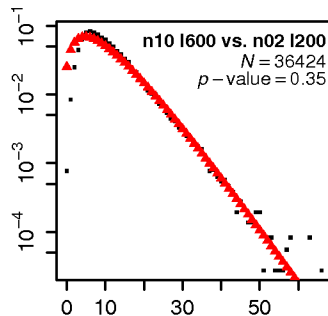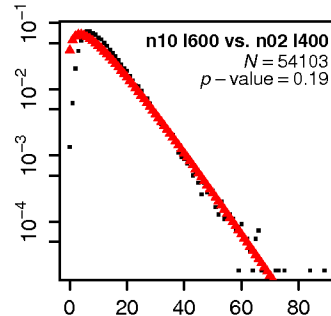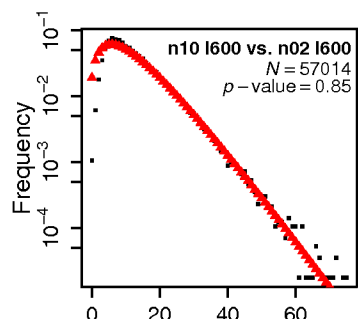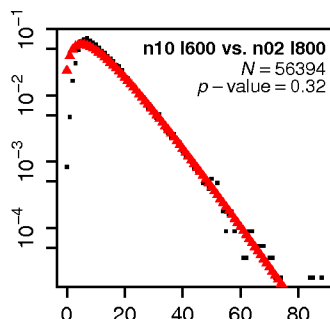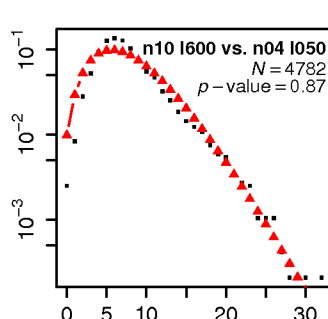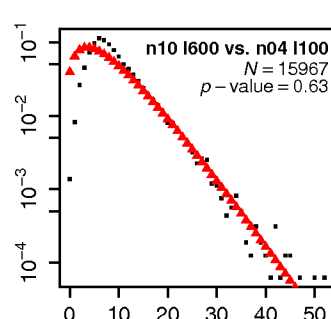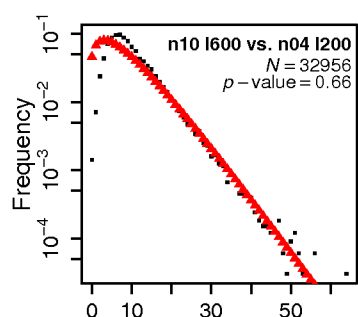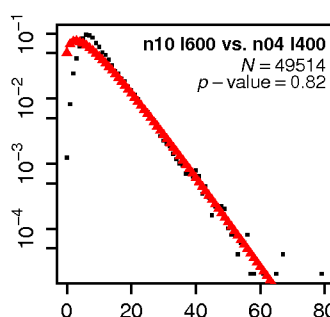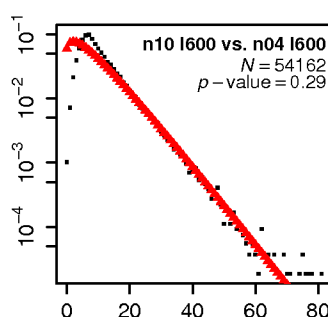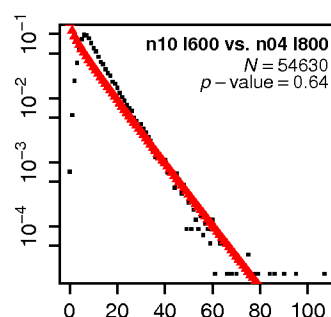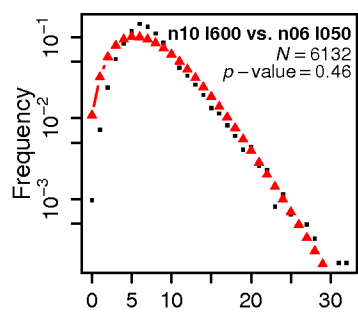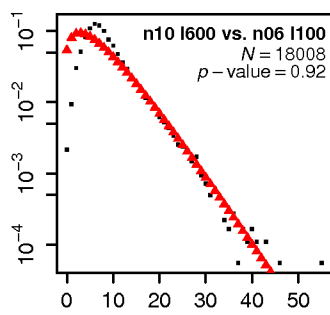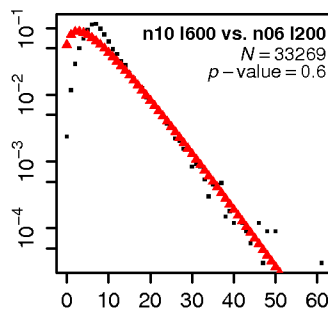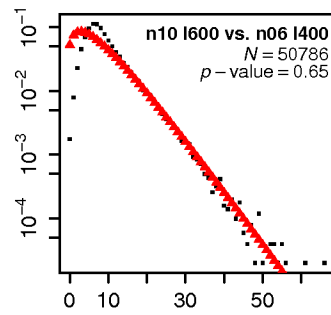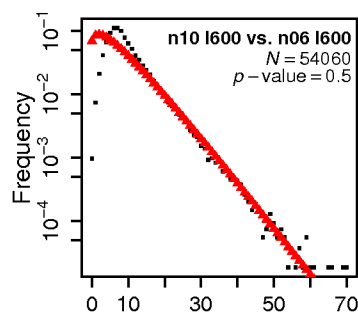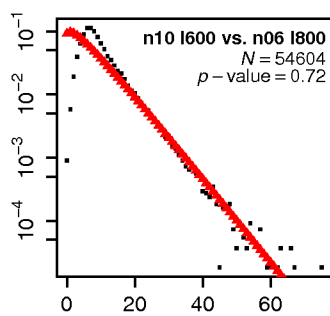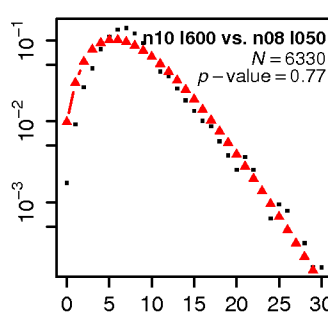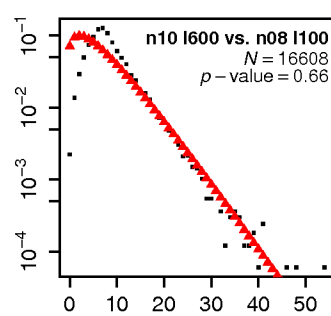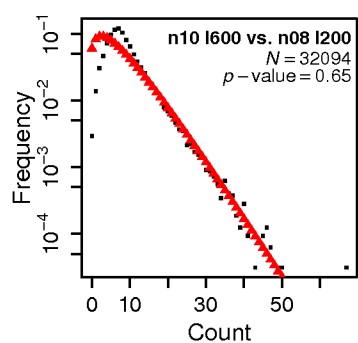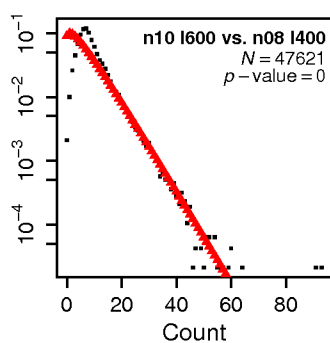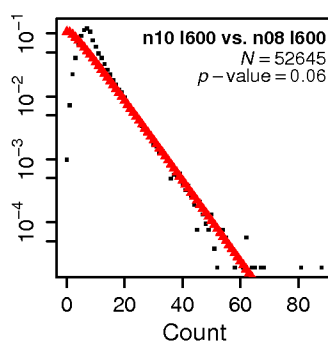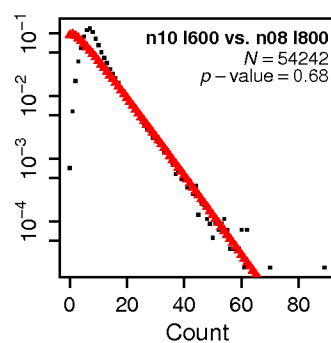

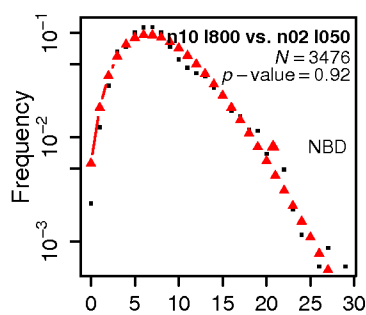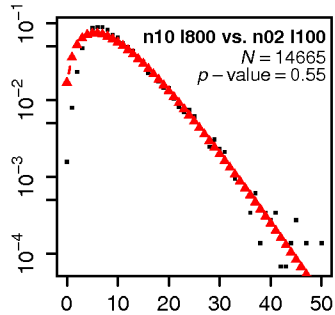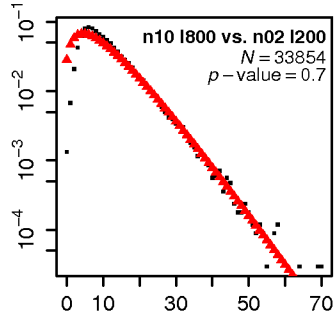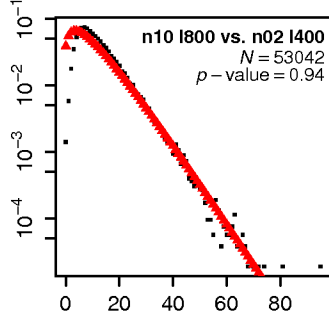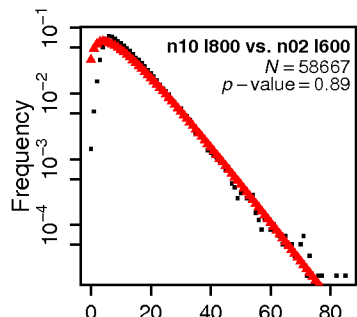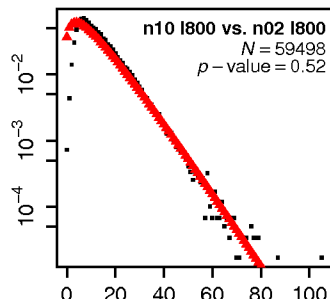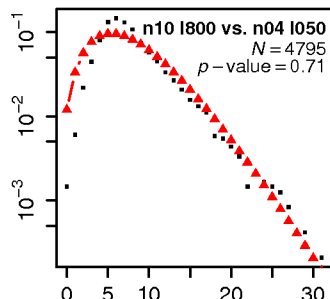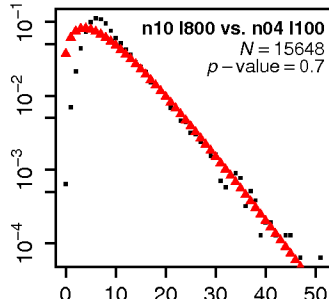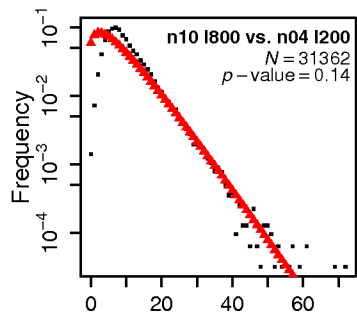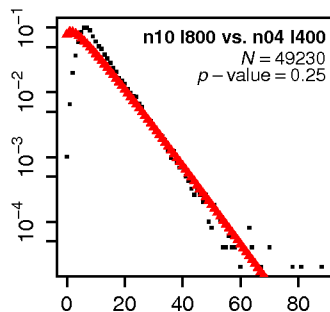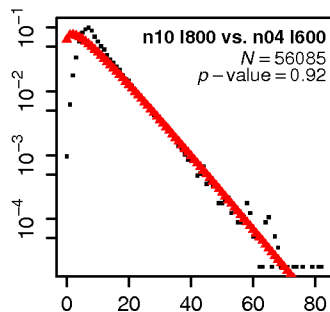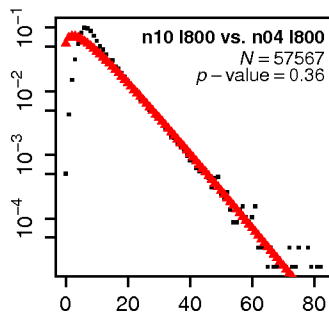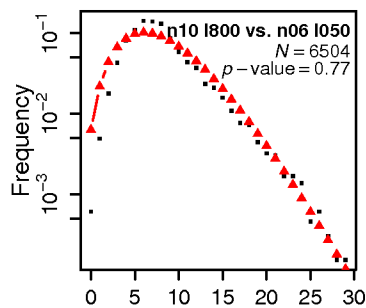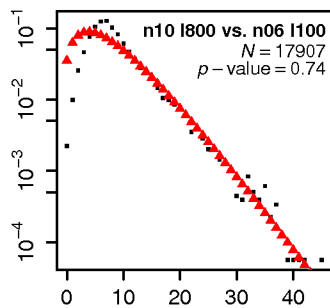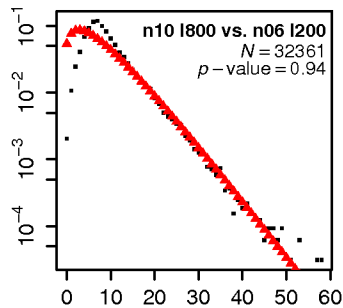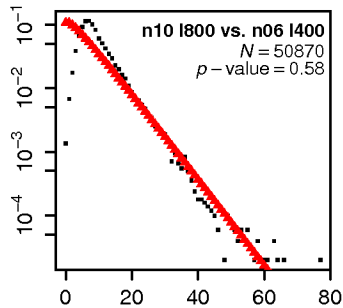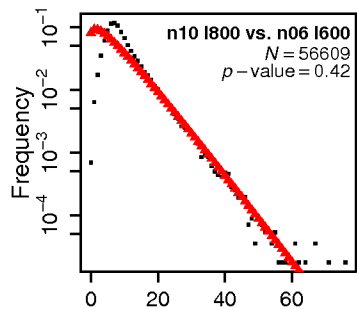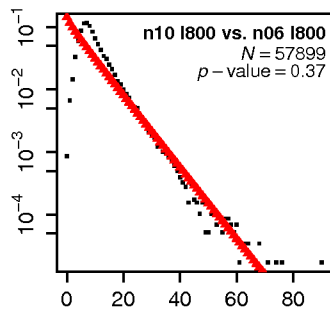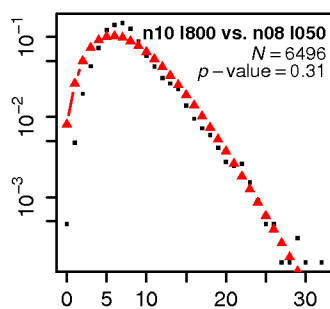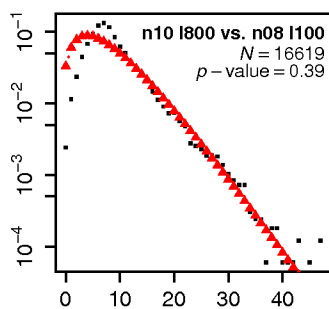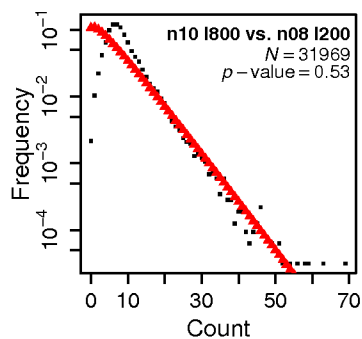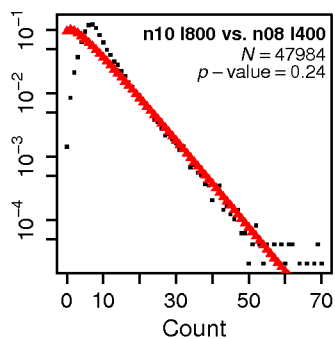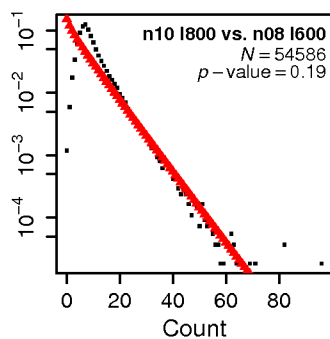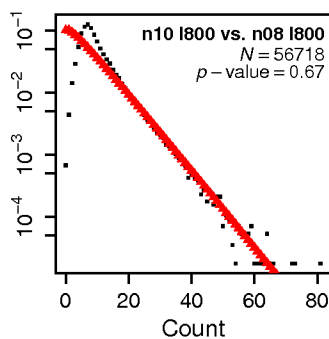

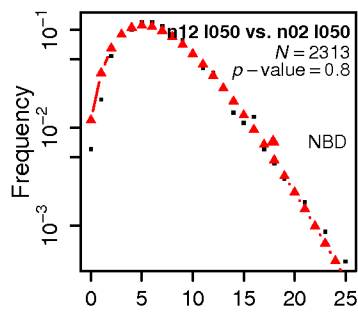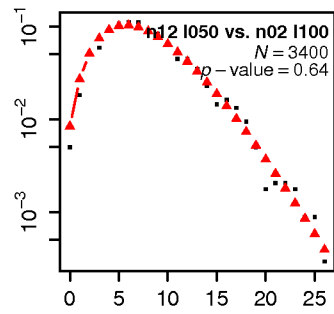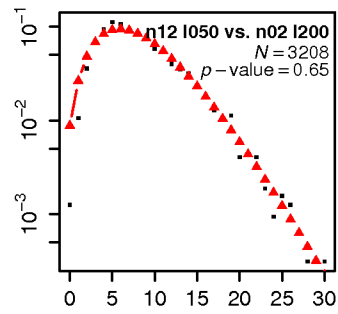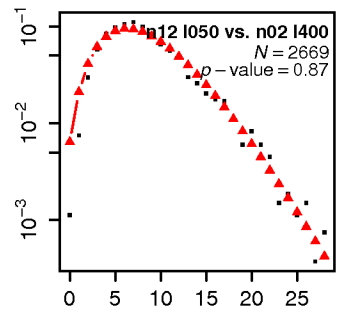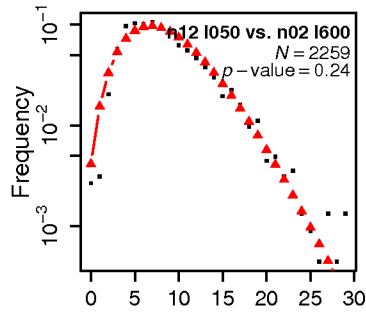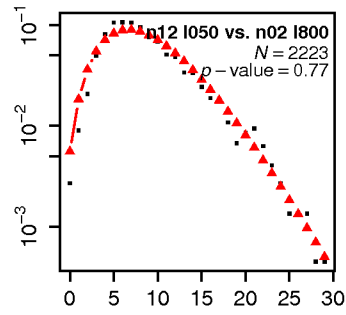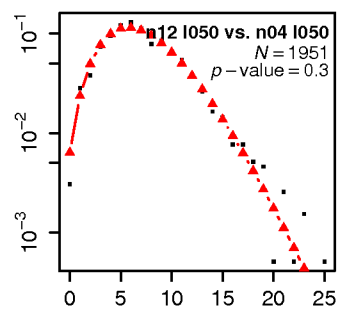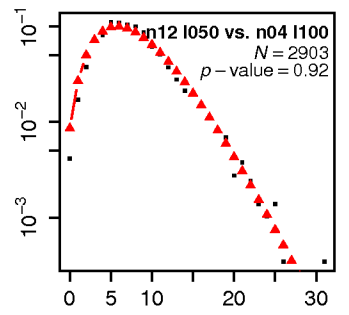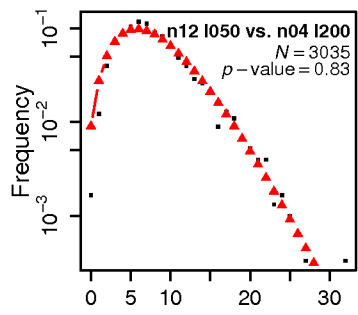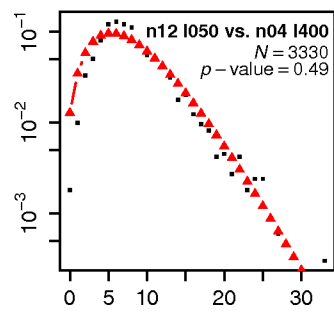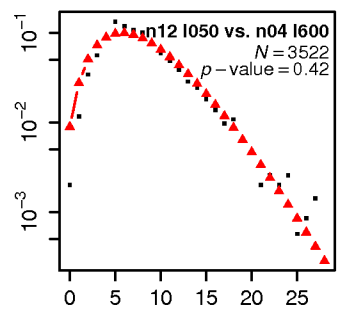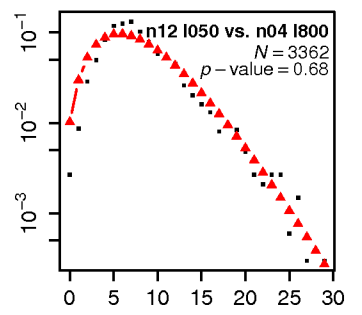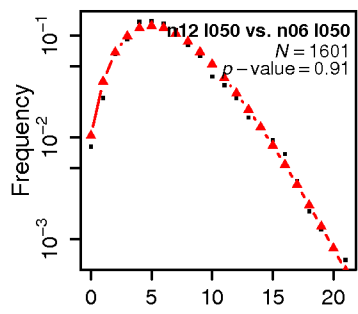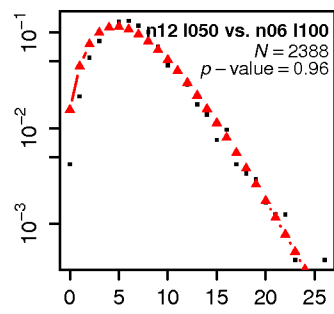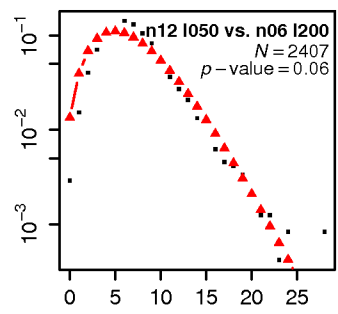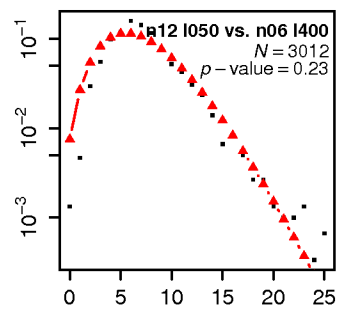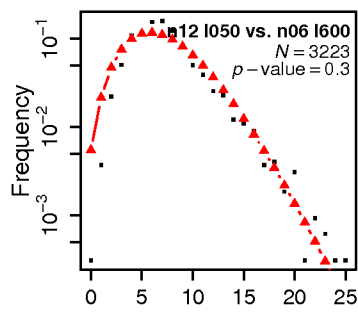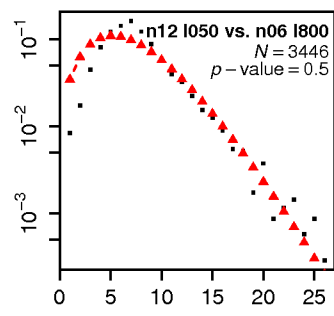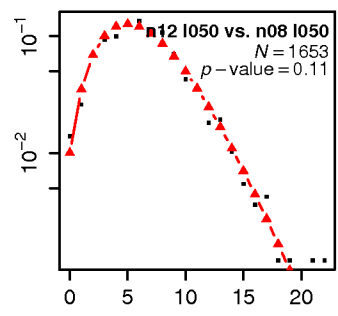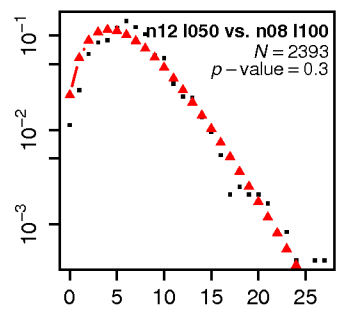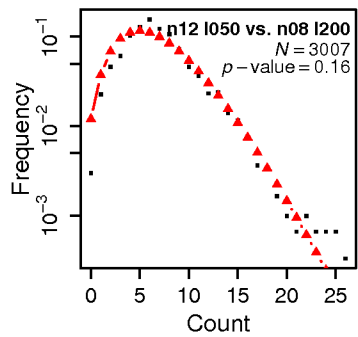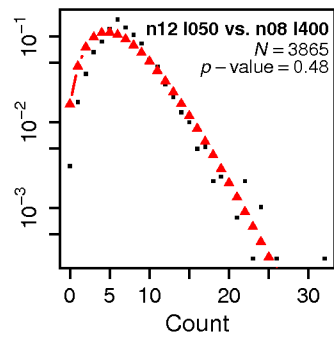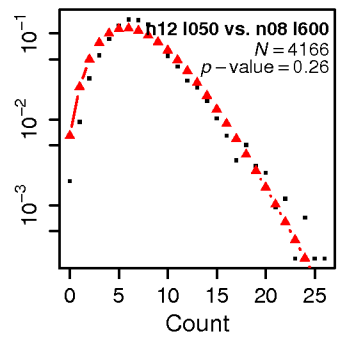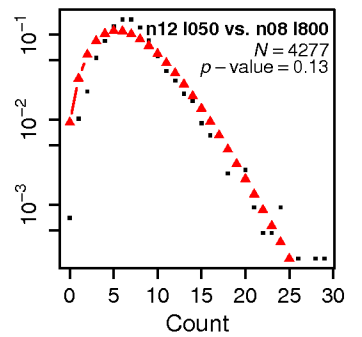

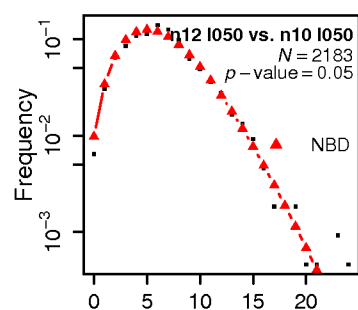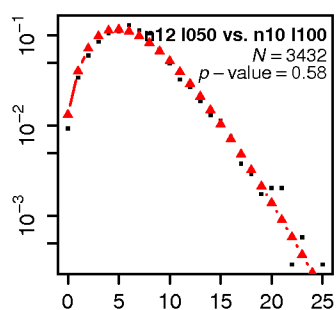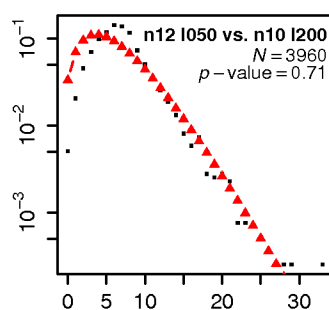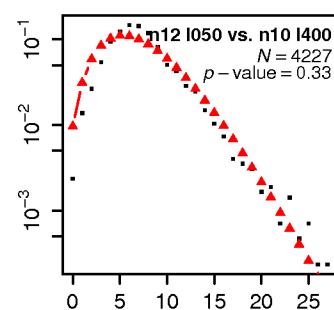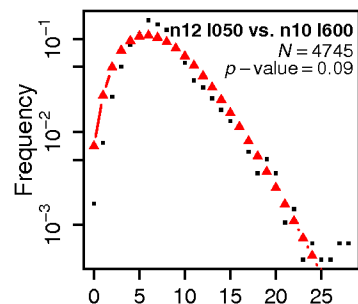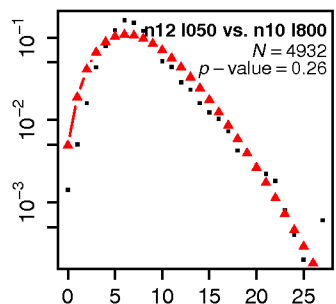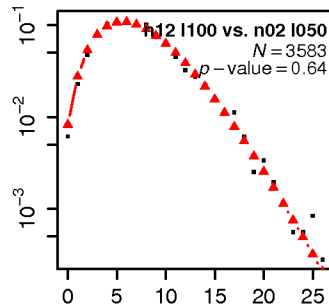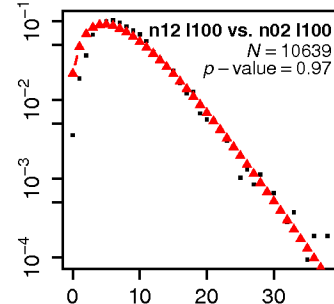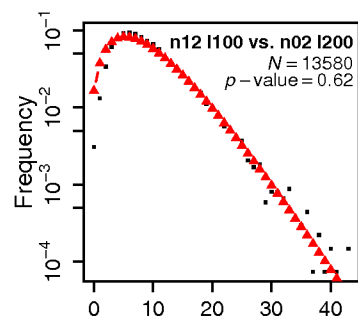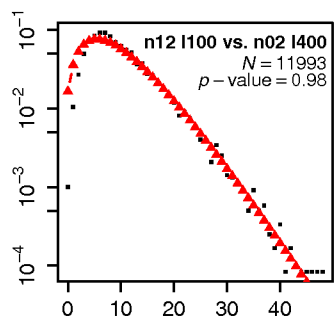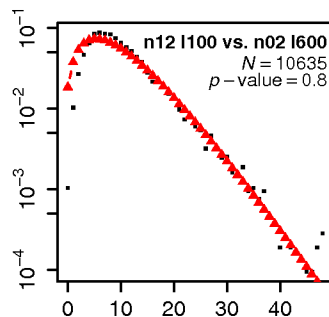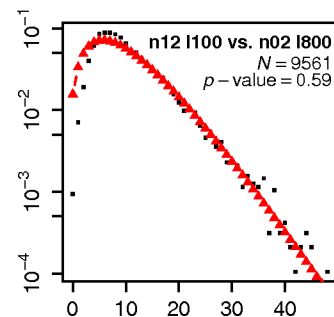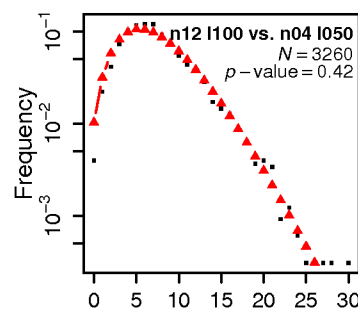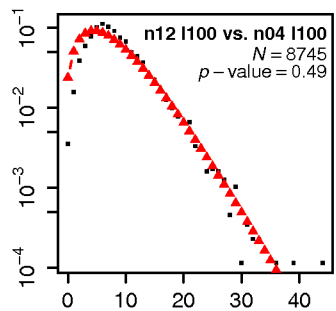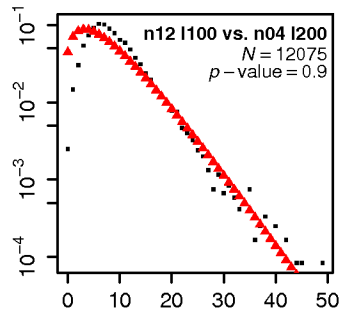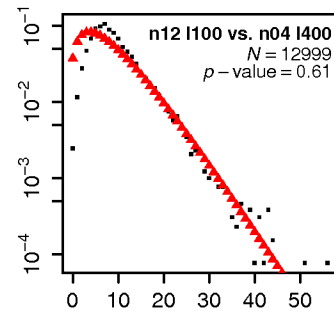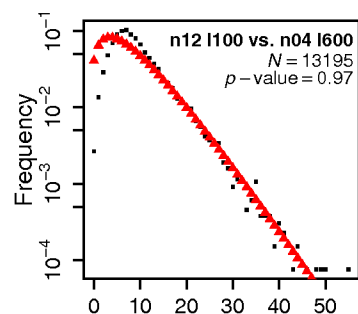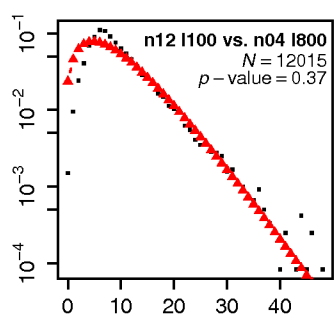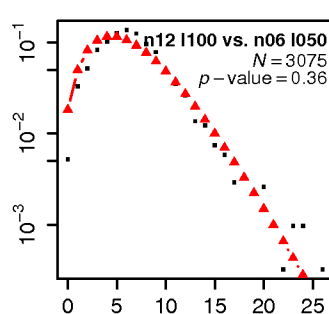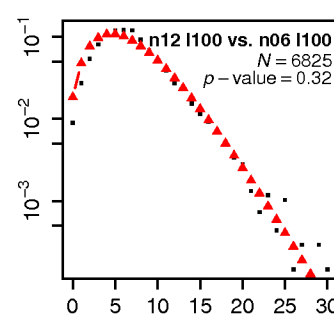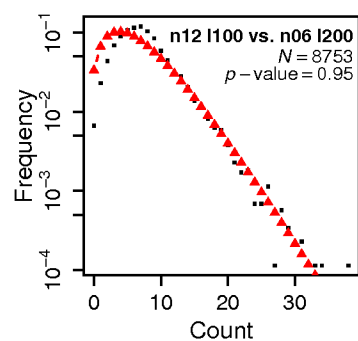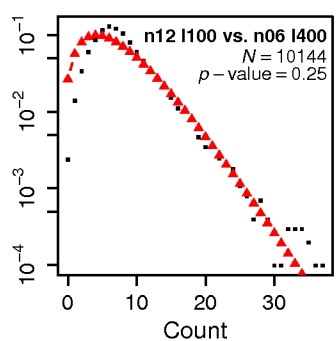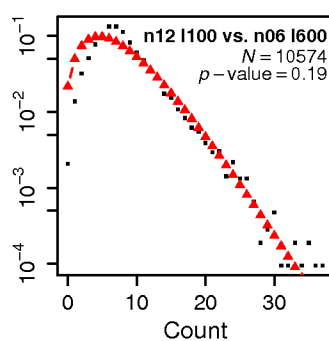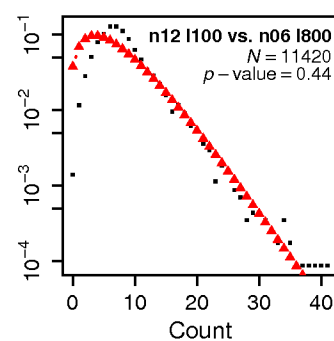

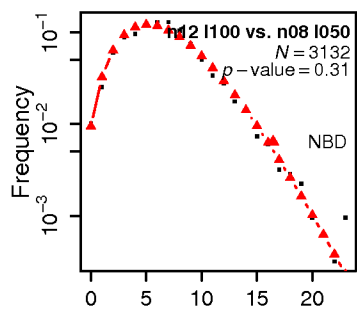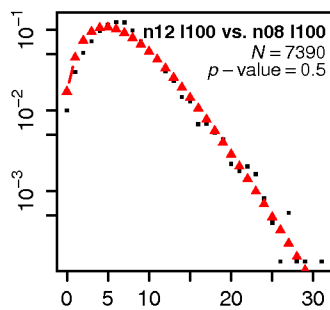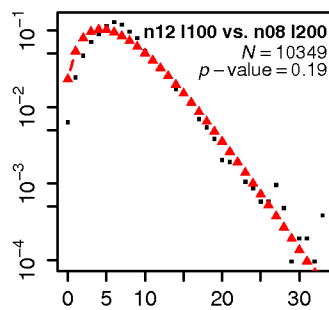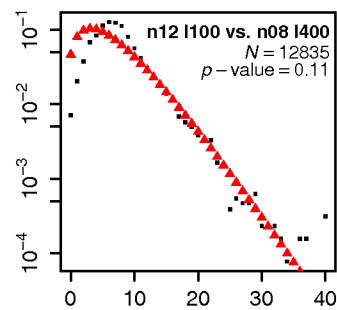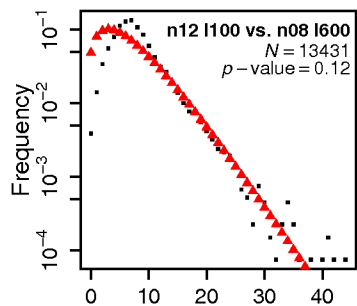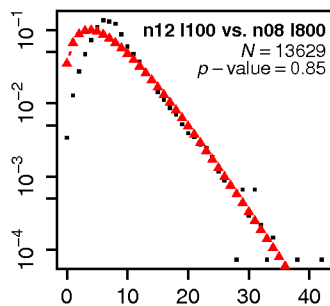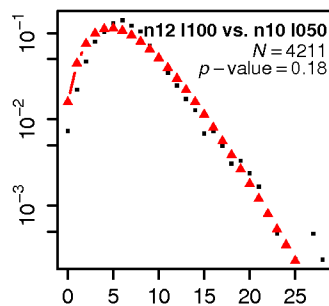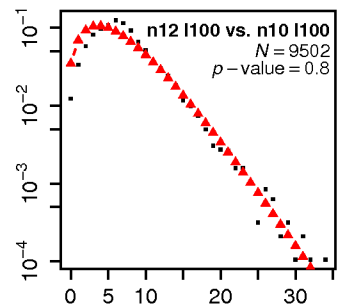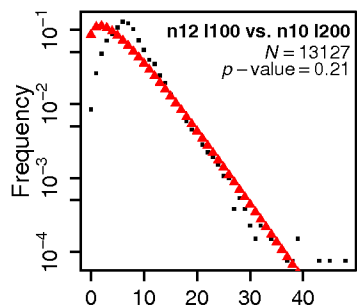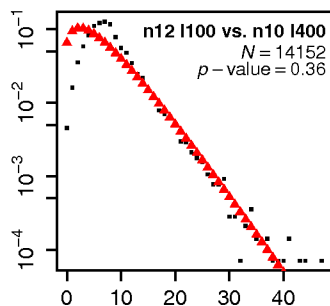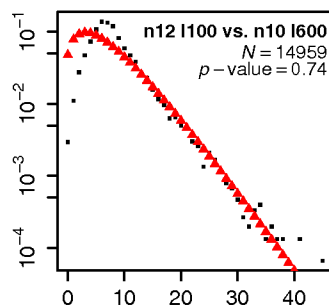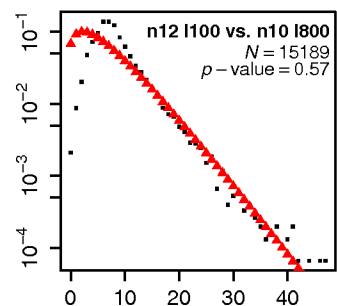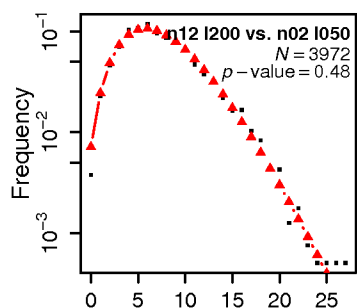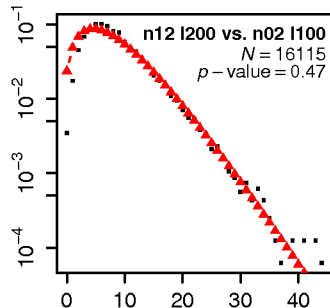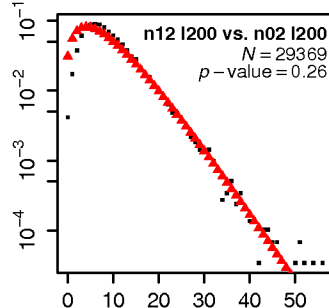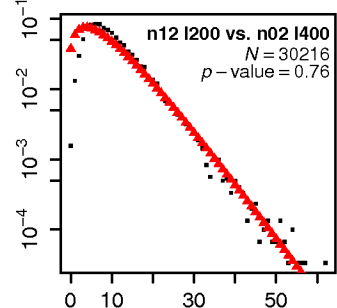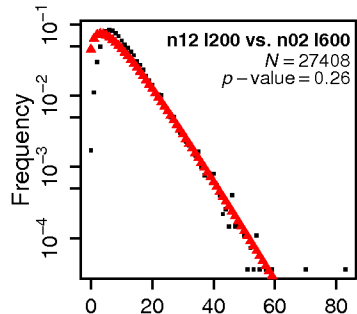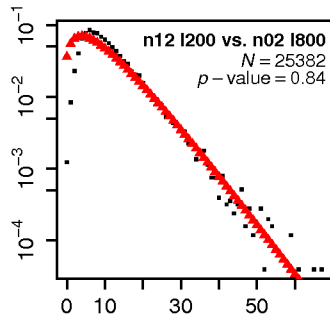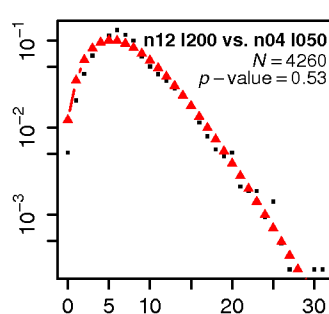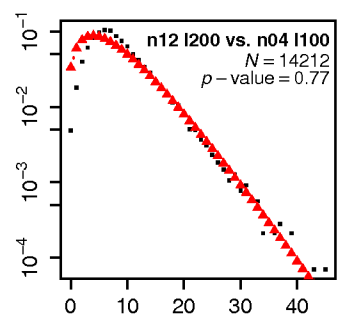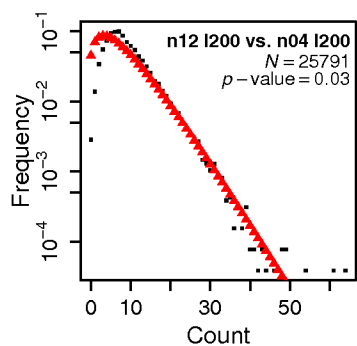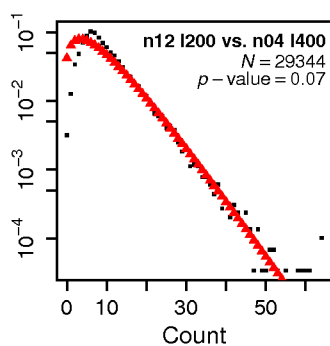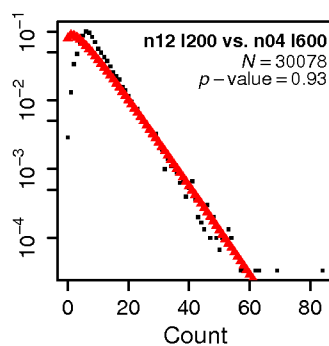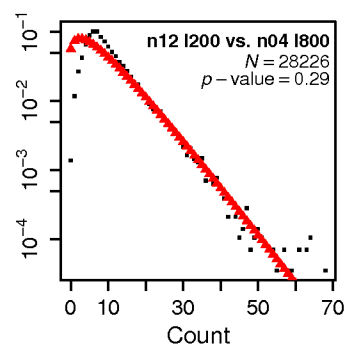

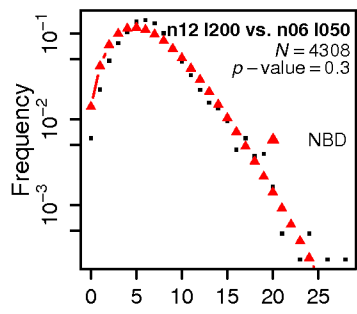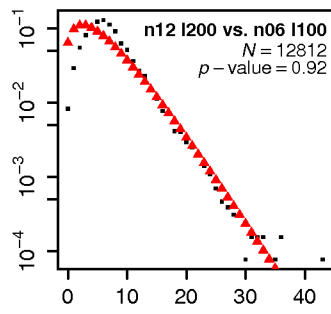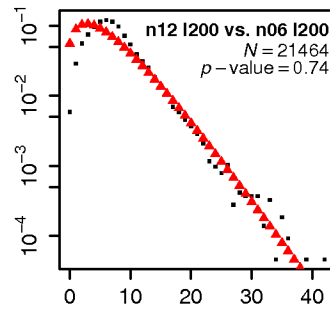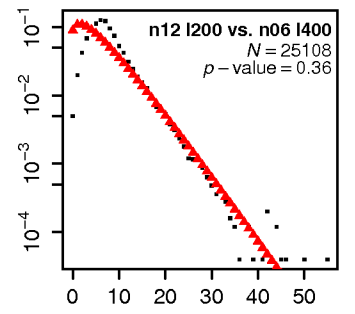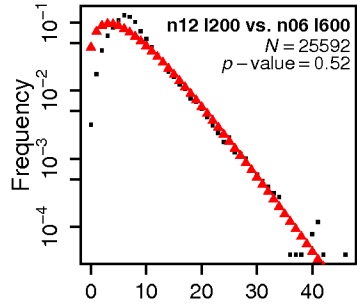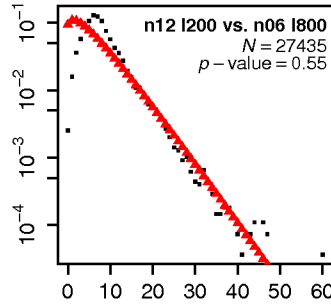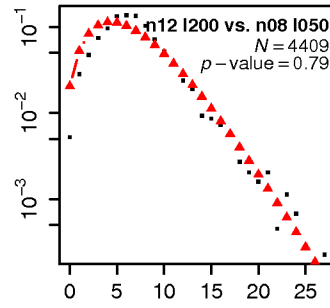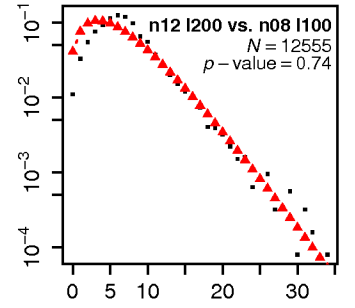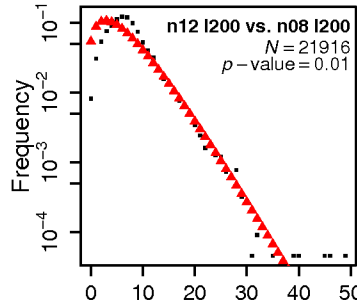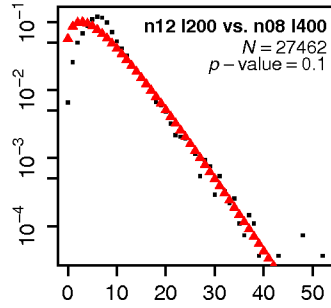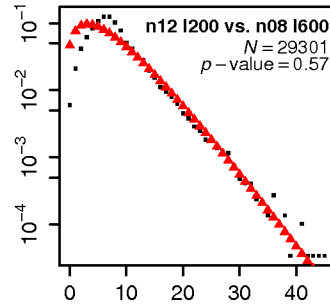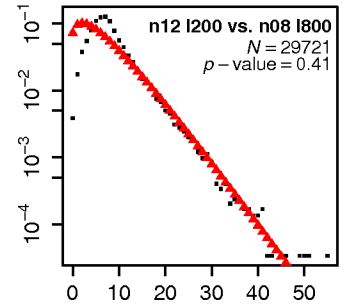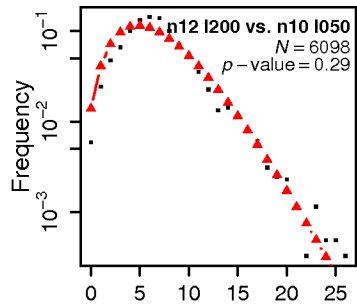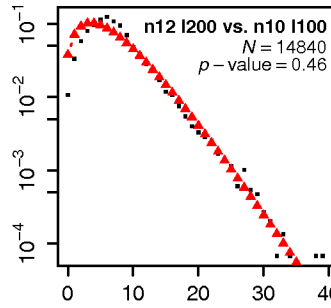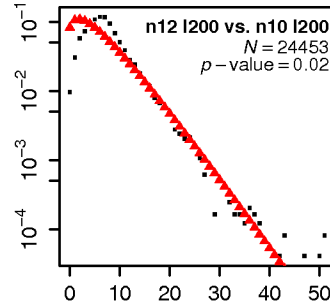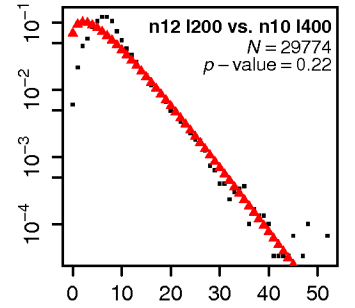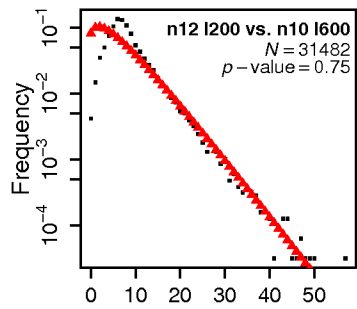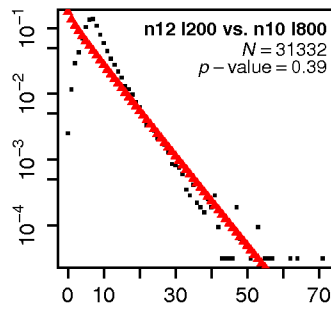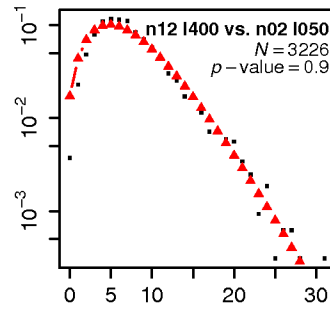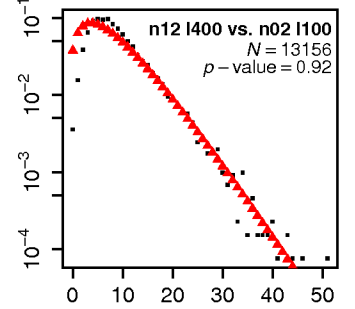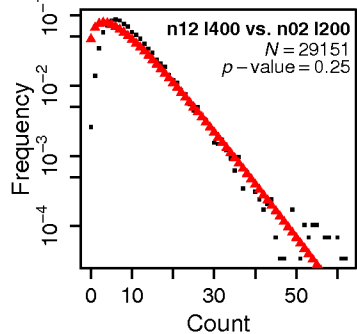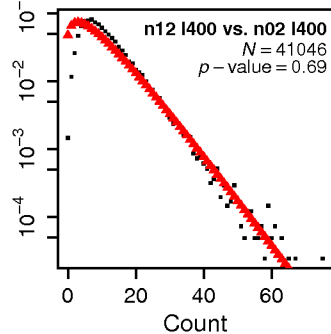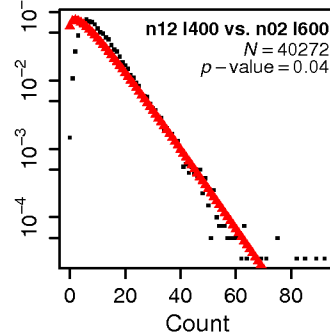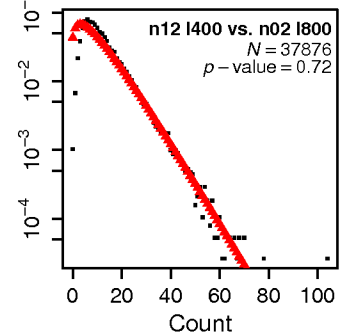

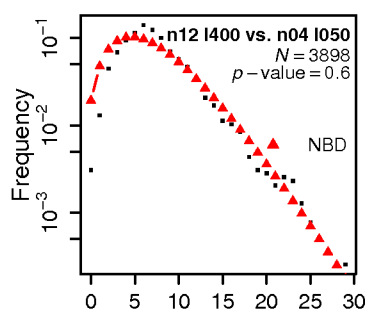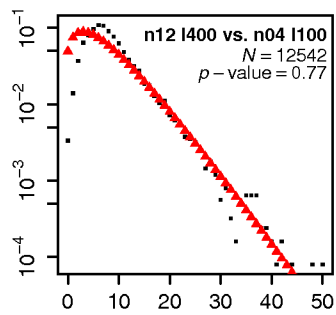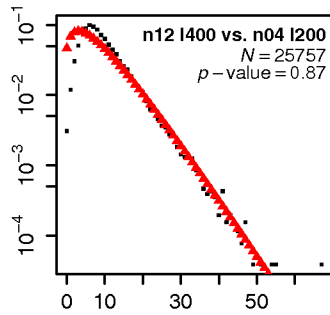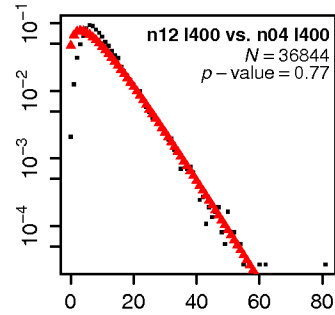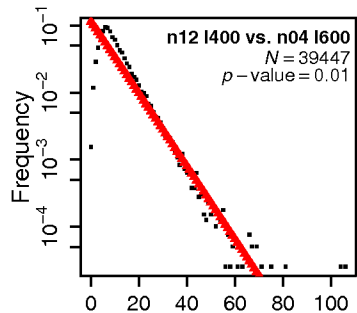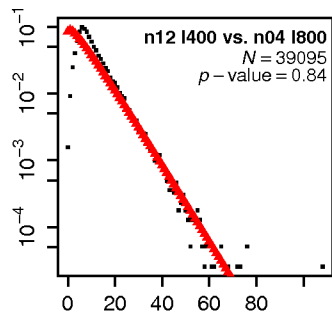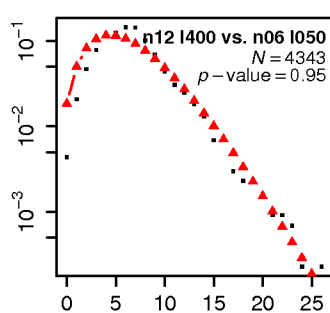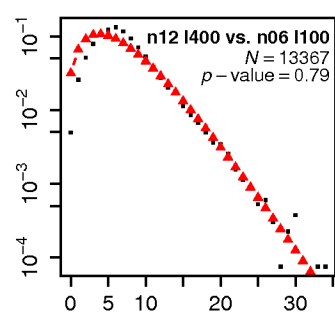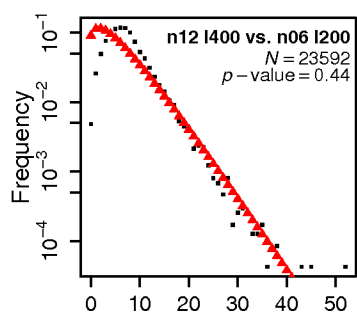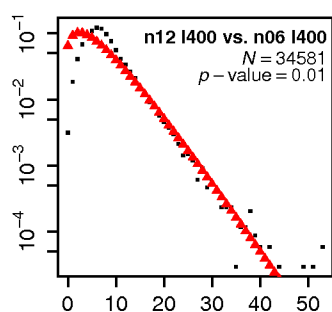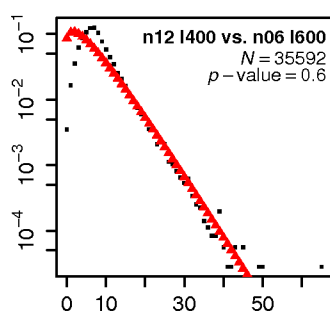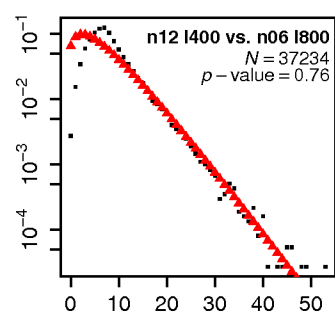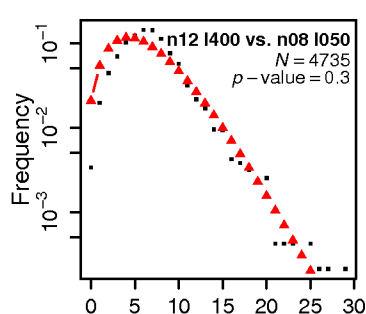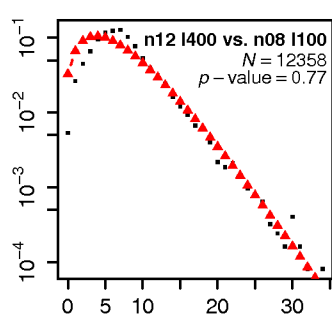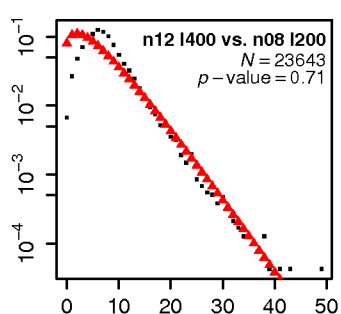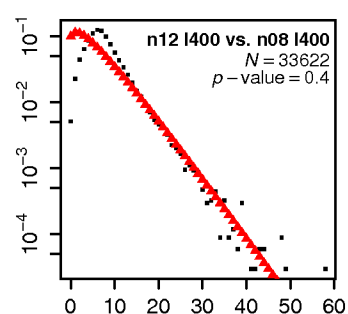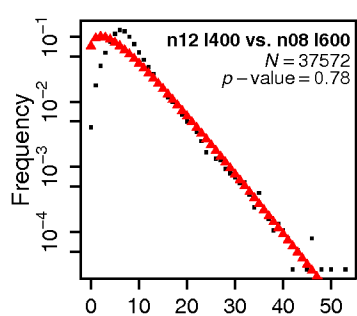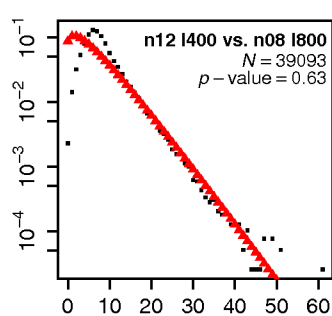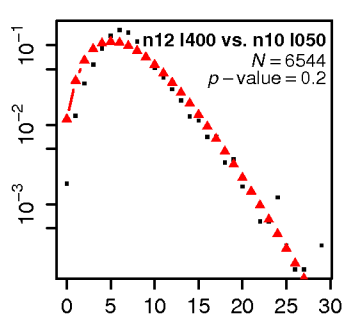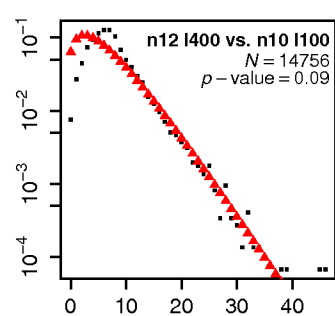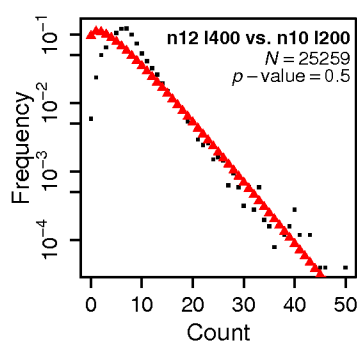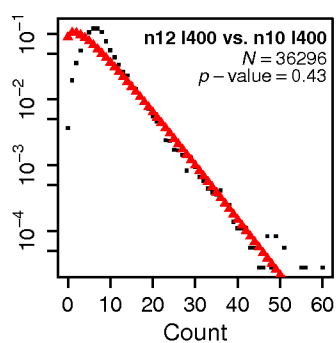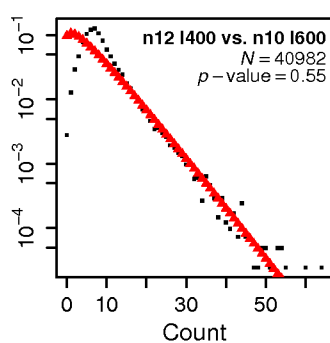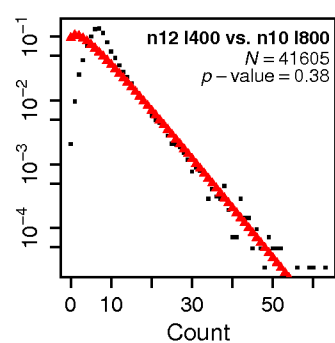

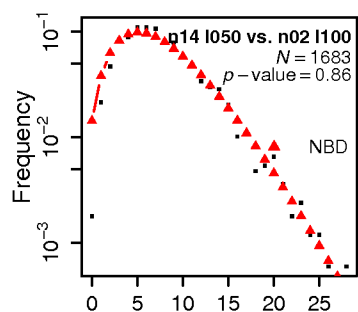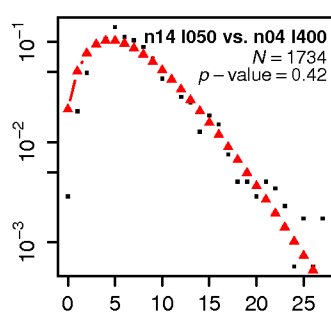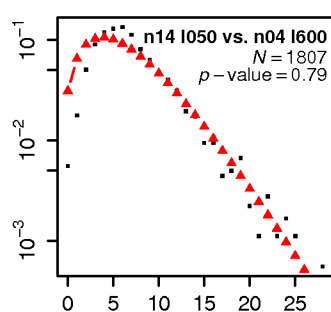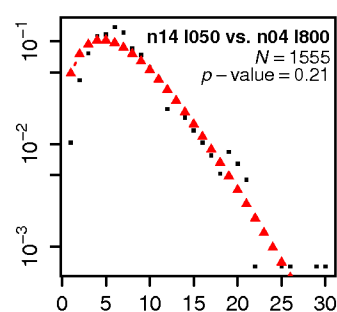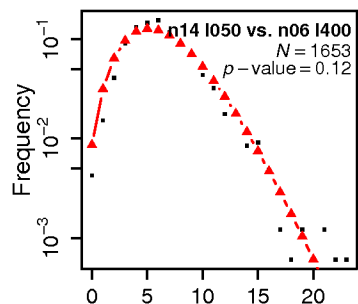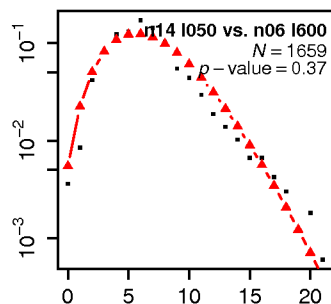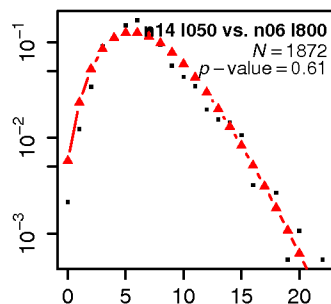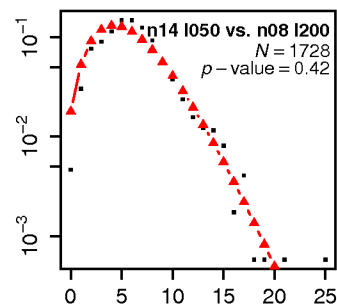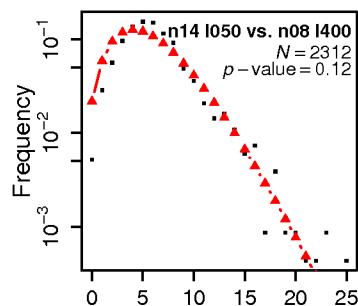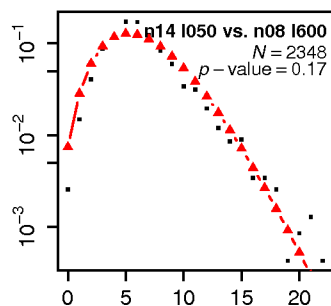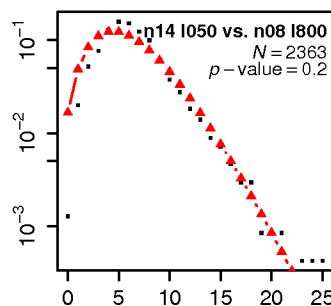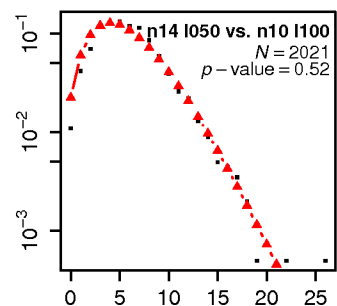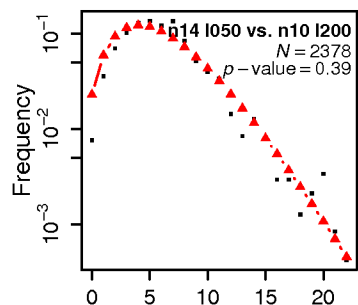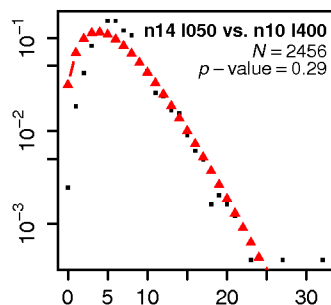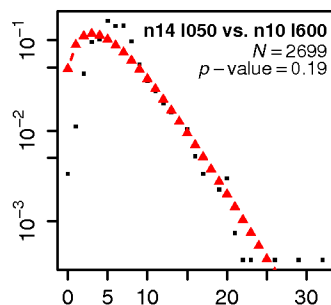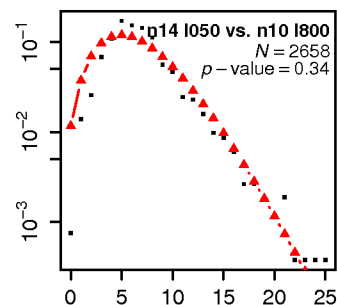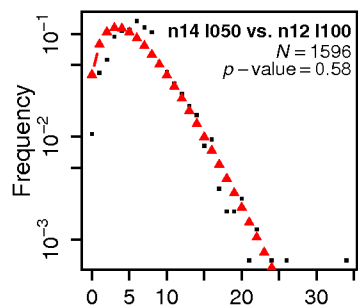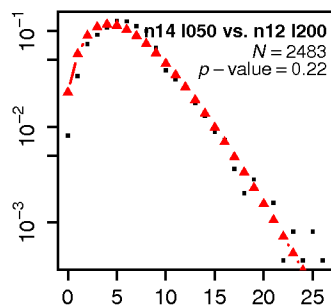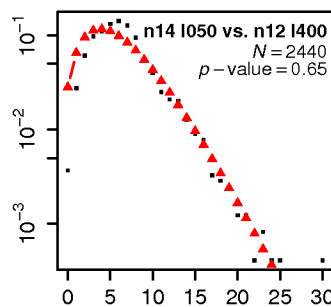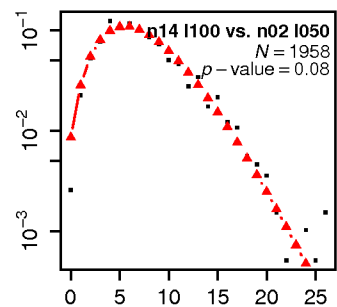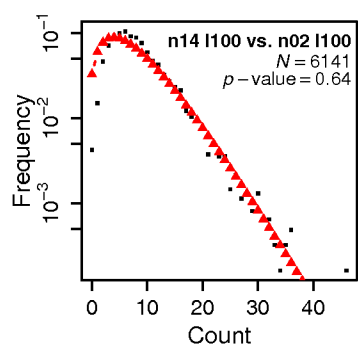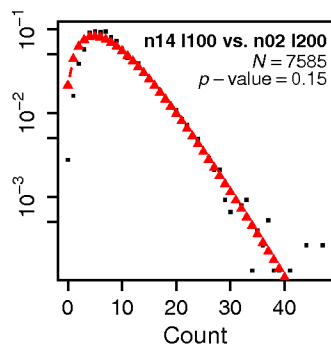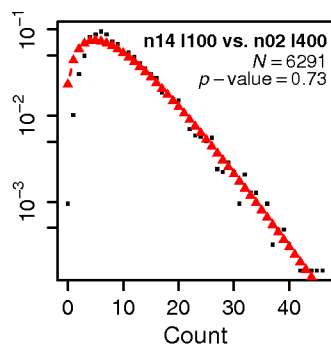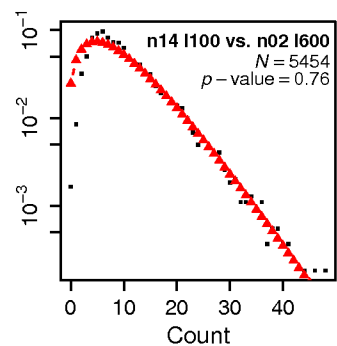

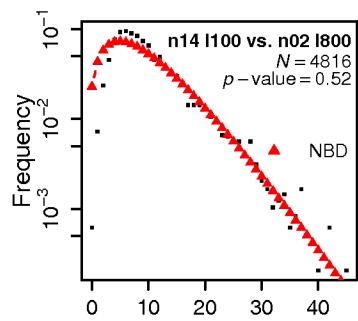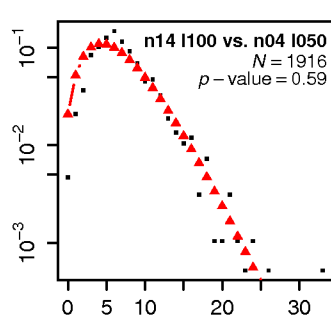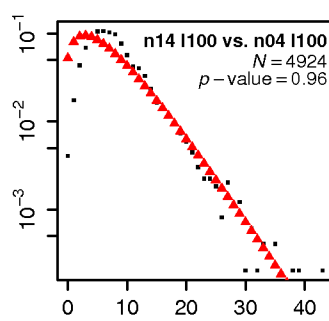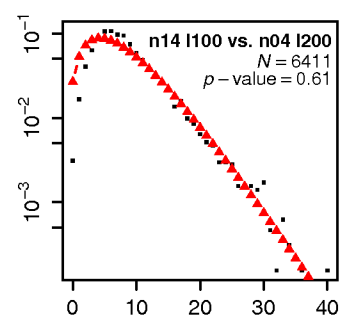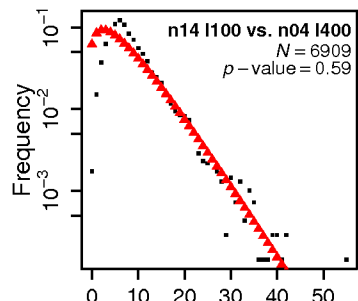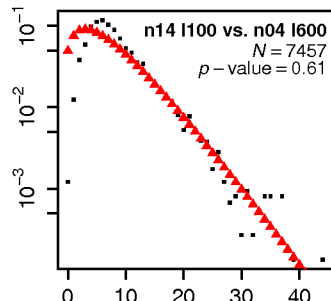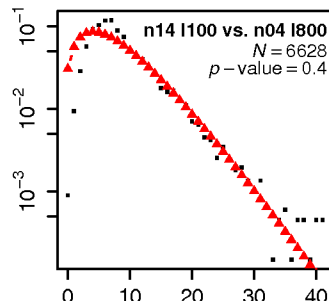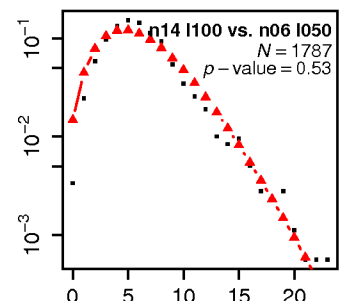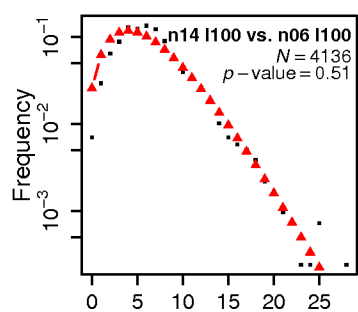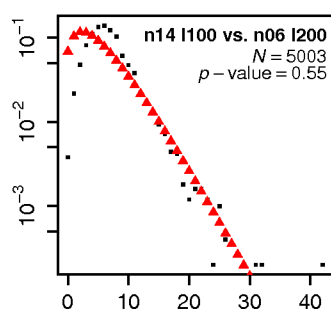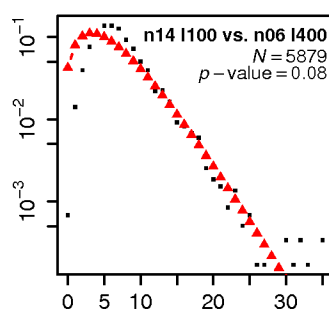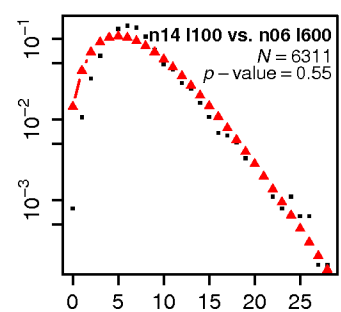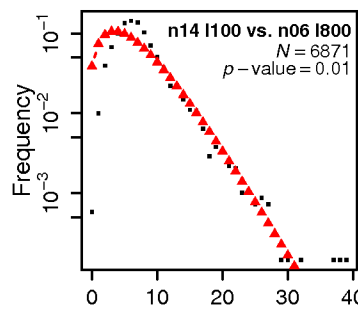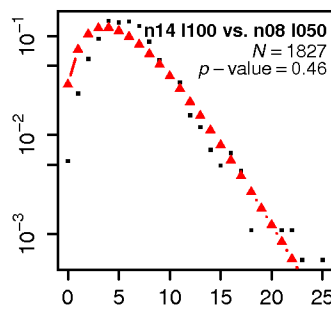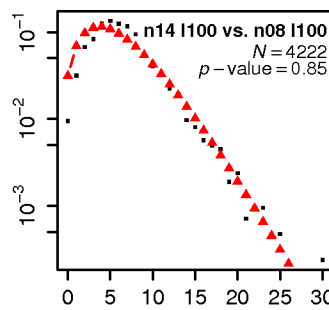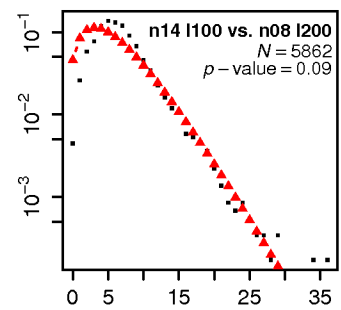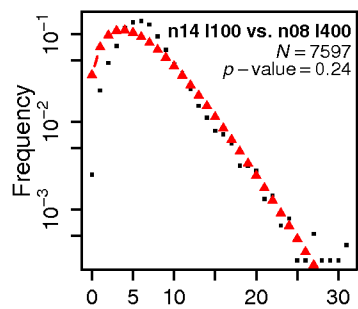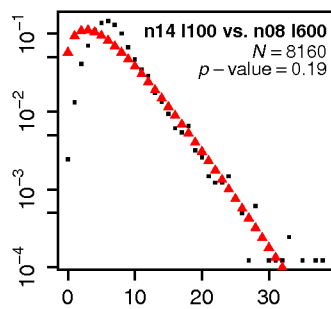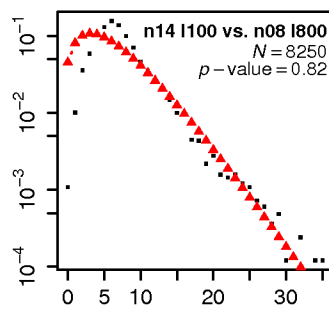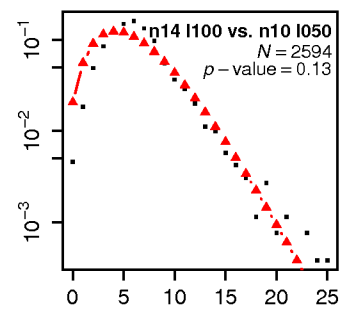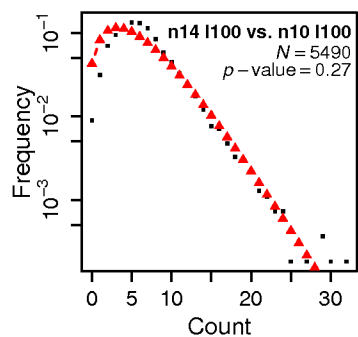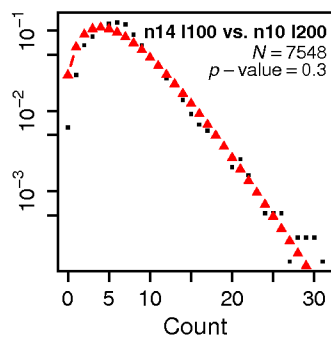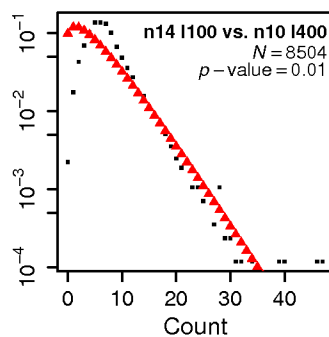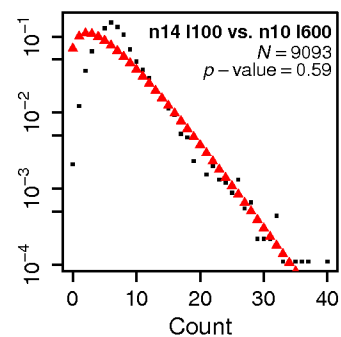

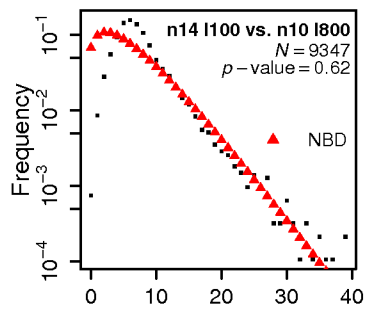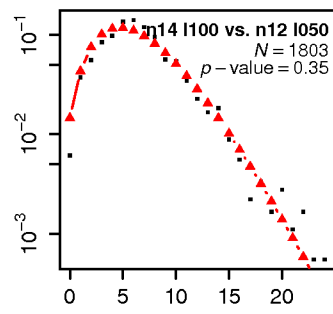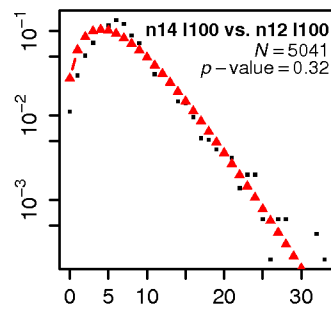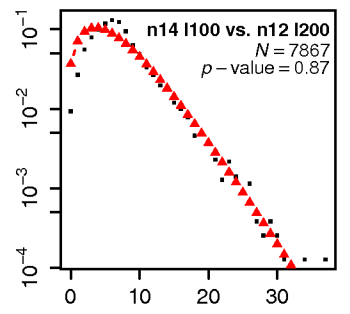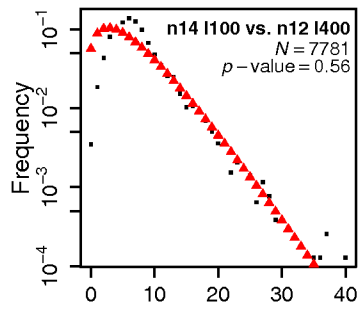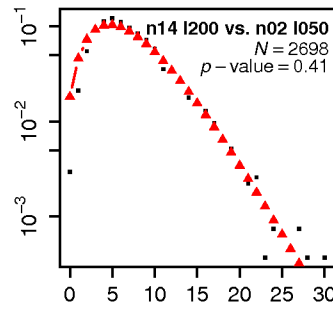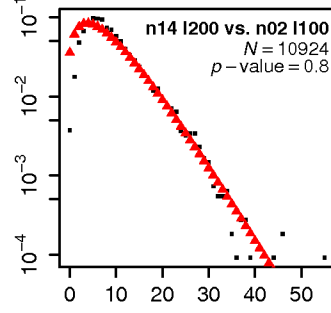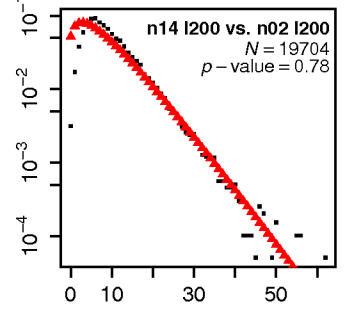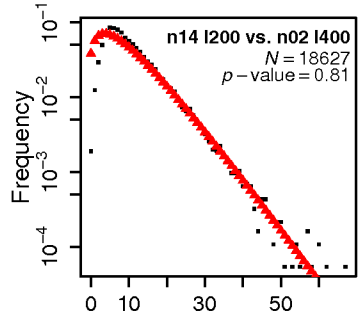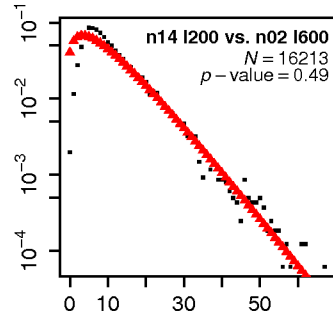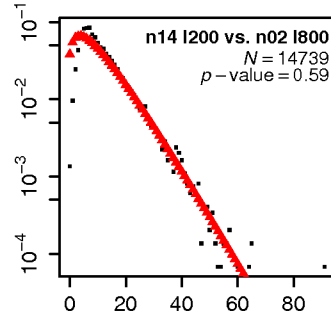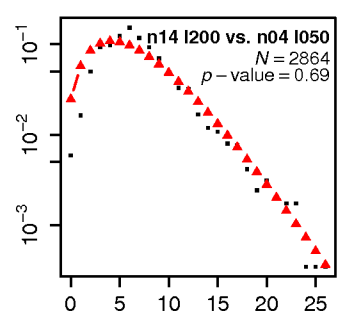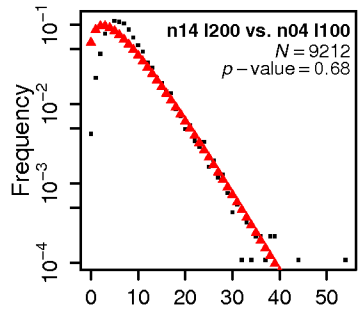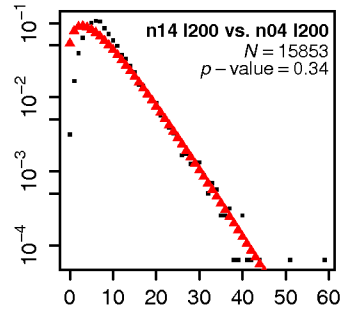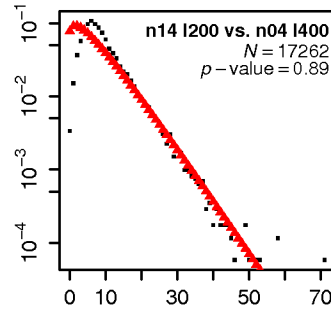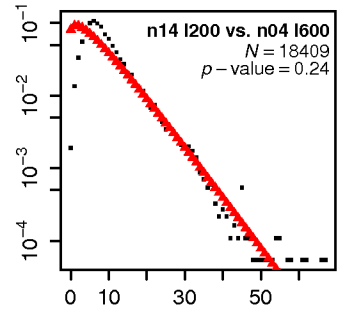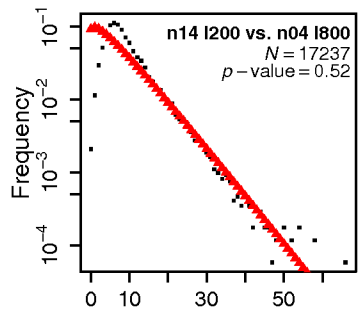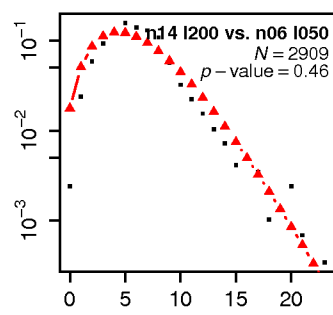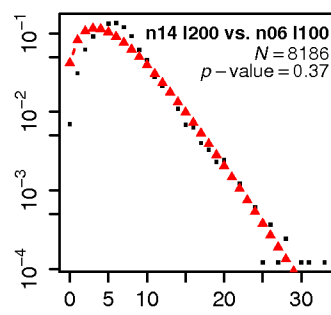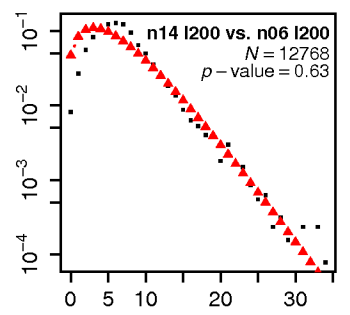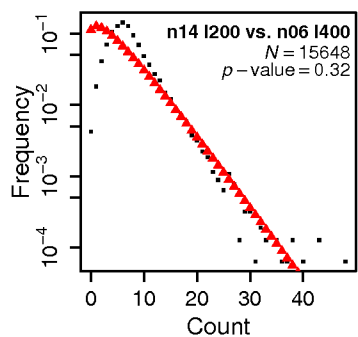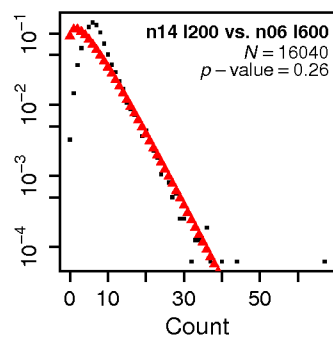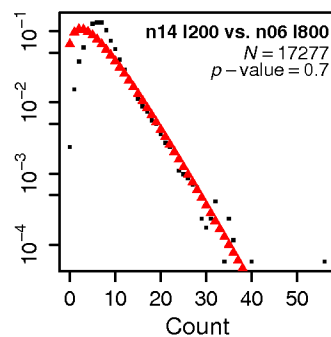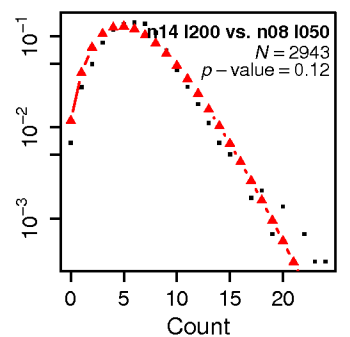

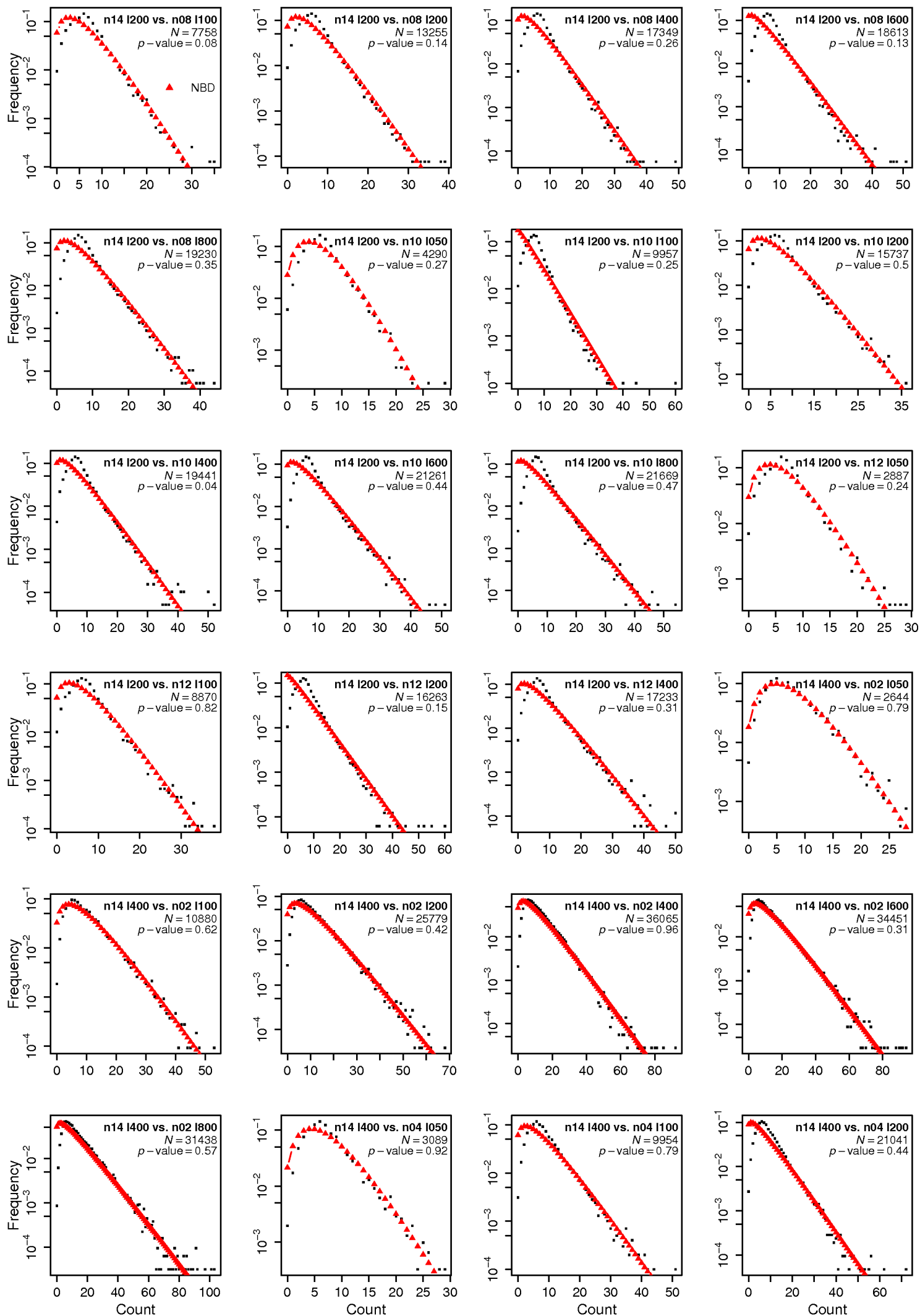

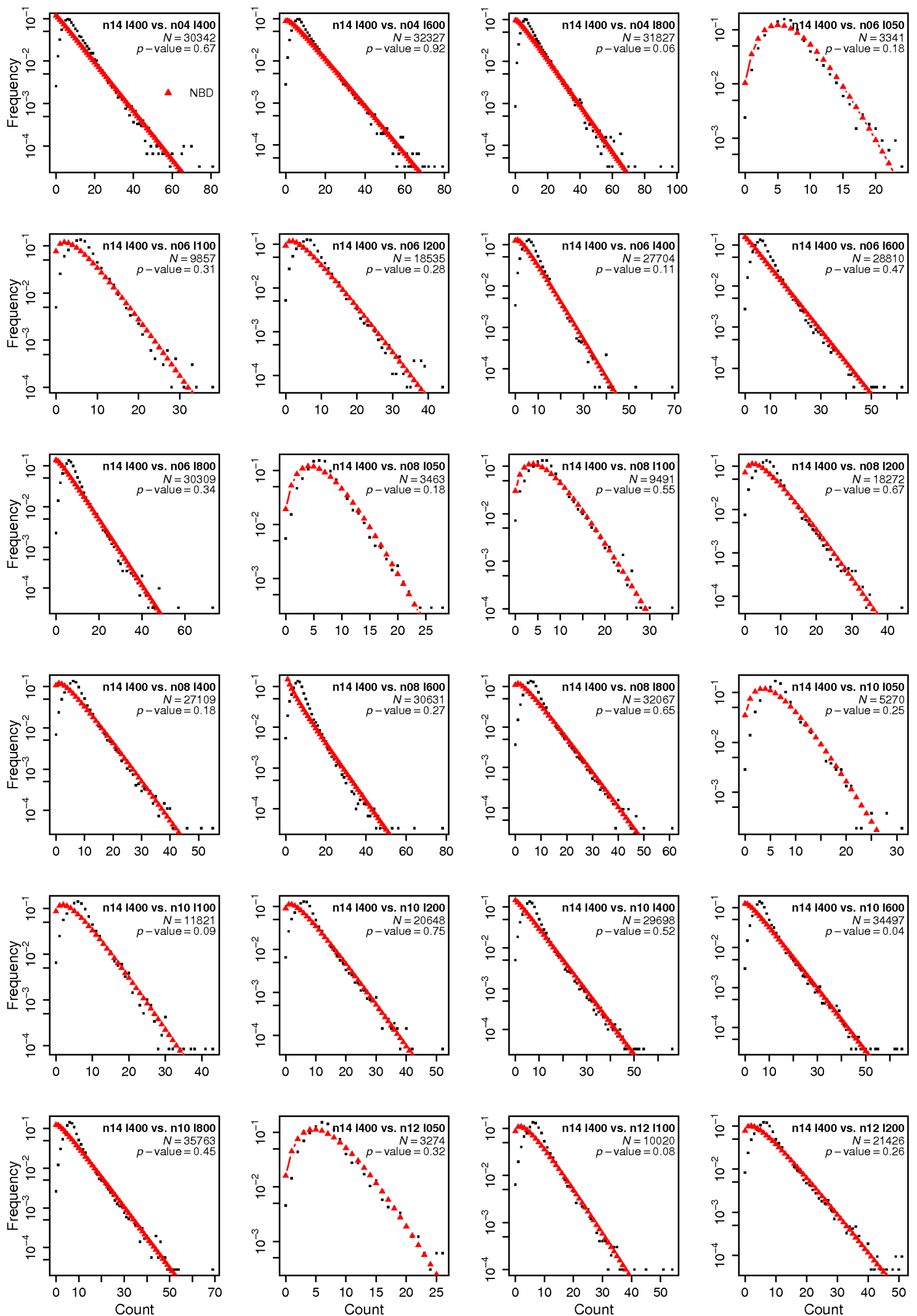

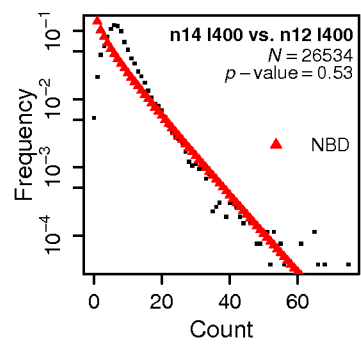

Supplement: Supplementary file 7 — Figure S13. Distributions of the number of positive substitution scores observed in alignments of simulated profiles of different values of ENO and length. (PDF 5,102 kb) [file 12859_2019_2913_MOESM7_ESM.pdf]
