# Supplementary material for: Estimating statistical significance of local protein profile-profile alignments
Source: BMC Bioinformatics. 2019 Aug 13;20:419. doi: 10.1186/s12859-019-2913-3 (PMC6693267; doi:10.1186/s12859-019-2913-3)
Supplement: Supplementary file 8 — Table S1. Goodness of fit of the EVD to the distribution of alignment scores of real unrelated profiles. (PDF 35 kb) [file 12859_2019_2913_MOESM8_ESM.pdf]

Table S1. Goodness of fit of the EVD to the distribution of alignment scores of real unrelated profiles

Distribution	N	Location		Scale		AD_{up}	p-value
		Estimate	SE	Estimate	SE		
n04 l100 vs. n02 l100	19203	9.538	0.0138	1.484	0.0114	94.68	0.44
n04 l100 vs. n02 l200	24532	10.189	0.0126	1.547	0.0106	121.90	0.35
n04 l100 vs. n02 l400	22970	10.780	0.0132	1.580	0.0111	121.66	0.29
n04 l100 vs. n02 l600	7283	11.145	0.0230	1.561	0.0194	57.92	0.40
n04 l200 vs. n02 l100	23329	10.058	0.0128	1.542	0.0108	107.91	0.40
n04 l200 vs. n02 l200	35788	10.497	0.0103	1.594	0.0091	133.56	0.41
n04 l200 vs. n02 l400	38364	11.084	0.0100	1.608	0.0088	141.05	0.39
n04 l200 vs. n02 l600	12703	11.502	0.0178	1.634	0.0156	186.39	0.12
n04 l400 vs. n02 l100	7494	10.581	0.0225	1.600	0.0198	60.81	0.42
n04 l400 vs. n02 l200	12701	11.072	0.0172	1.616	0.0155	65.18	0.59
n04 l400 vs. n02 l400	15501	11.599	0.0155	1.632	0.0142	84.38	0.41
n04 l400 vs. n02 l600	5328	12.029	0.0267	1.664	0.0247	113.48	0.19
n06 l100 vs. n02 l100	16150	9.455	0.0150	1.475	0.0124	584.59	0.06
n06 l100 vs. n02 l200	20905	10.071	0.0132	1.534	0.0113	109.87	0.32
n06 l100 vs. n02 l400	19787	10.707	0.0138	1.578	0.0120	99.87	0.45
n06 l100 vs. n02 l600	6261	11.069	0.0252	1.577	0.0211	42.41	0.59
n06 l100 vs. n04 l100	16502	10.117	0.0155	1.653	0.0139	139.25	0.27
n06 l100 vs. n04 l200	19622	10.665	0.0152	1.782	0.0137	313.21	0.22
n06 l100 vs. n04 l400	6255	11.224	0.0264	1.764	0.0240	135.75	0.18
n06 l200 vs. n02 l100	19190	9.946	0.0140	1.550	0.0120	87.57	0.45
n06 l200 vs. n02 l200	31126	10.315	0.0108	1.574	0.0096	126.71	0.41
n06 l200 vs. n02 l400	33869	10.898	0.0104	1.587	0.0093	126.55	0.43
n06 l200 vs. n02 l600	11427	11.310	0.0186	1.658	0.0167	69.15	0.44
n06 l200 vs. n04 l100	18987	10.646	0.0152	1.763	0.0138	218.55	0.23
n06 l200 vs. n04 l200	27195	10.950	0.0128	1.864	0.0124	1552.07	0.02
n06 l200 vs. n04 l400	10192	11.440	0.0204	1.794	0.0193	1232.97	0.01
n06 l400 vs. n02 l100	4263	10.343	0.0294	1.565	0.0257	45.61	0.39
n06 l400 vs. n02 l200	7680	10.739	0.0222	1.585	0.0195	48.39	0.77
n06 l400 vs. n02 l400	9985	11.182	0.0188	1.559	0.0168	59.44	0.56
n06 l400 vs. n02 l600	3513	11.677	0.0328	1.639	0.0298	49.25	0.30
n06 l400 vs. n04 l100	4277	11.138	0.0345	1.860	0.0306	71.65	0.34
n06 l400 vs. n04 l200	7028	11.367	0.0250	1.883	0.0246	316.05	0.14
n06 l400 vs. n04 l400	3030	11.867	0.0377	1.886	0.0376	267.59	0.04
n08 l100 vs. n02 l100	13800	9.298	0.0155	1.420	0.0128	85.25	0.34
n08 l100 vs. n02 l200	17912	9.888	0.0137	1.472	0.0117	92.20	0.42
n08 l100 vs. n02 l400	16600	10.553	0.0149	1.560	0.0129	84.19	0.41
n08 l100 vs. n02 l600	5075	10.932	0.0278	1.566	0.0234	48.98	0.32
n08 l100 vs. n04 l100	16271	10.026	0.0158	1.618	0.0136	74.61	0.52
n08 l100 vs. n04 l200	19168	10.611	0.0150	1.766	0.0138	76.14	0.66
n08 l100 vs. n04 l400	5946	11.223	0.0273	1.750	0.0242	519.38	0.02
n08 l100 vs. n06 l100	18426	10.476	0.0156	1.870	0.0150	334.87	0.11

n08 l100 vs. n06 l200	20915	11.035	0.0152	1.937	0.0146	776.73	0.04
n08 l100 vs. n06 l400	4534	11.557	0.0325	1.842	0.0295	339.67	0.08
n08 l200 vs. n02 l100	12430	9.687	0.0169	1.514	0.0145	80.53	0.40
n08 l200 vs. n02 l200	21336	9.955	0.0125	1.535	0.0114	90.22	0.38
n08 l200 vs. n02 l400	23261	10.549	0.0120	1.514	0.0107	84.86	0.65
n08 l200 vs. n02 l600	7785	10.933	0.0214	1.567	0.0191	242.85	0.13
n08 l200 vs. n04 l100	13999	10.420	0.0171	1.727	0.0158	71.27	0.57
n08 l200 vs. n04 l200	20448	10.666	0.0145	1.836	0.0140	757.47	0.07
n08 l200 vs. n04 l400	7413	11.224	0.0240	1.788	0.0225	595.70	0.03
n08 l200 vs. n06 l100	15798	10.855	0.0175	1.942	0.0169	207.80	0.17
n08 l200 vs. n06 l200	21528	11.122	0.0151	2.032	0.0153	2389.34	0.03
n08 l200 vs. n06 l400	5274	11.569	0.0302	2.016	0.0305	1186.85	0.02

Distribution represents distributions obtained from aligning reference profiles of ENO n and length l against reference profiles with different values of n and l. The table reports the estimates and their standard errors (SE) for the location and scale parameters of the EVD for each distribution of alignment scores. N is the number of alignment scores. AD_{up} is the supremum class upper-tail Anderson-Darling statistic. The p-value of statistic AD_{up} was computed by Monte Carlo simulation with 100 samples.
