# Supplementary material for: Estimating statistical significance of local protein profile-profile alignments
Source: BMC Bioinformatics. 2019 Aug 13;20:419. doi: 10.1186/s12859-019-2913-3 (PMC6693267; doi:10.1186/s12859-019-2913-3)
Supplement: Supplementary file 9 — Table S2. Goodness of fit of the EVD to the distribution of alignment scores of real unrelated profiles. (PDF 35 kb) [file 12859_2019_2913_MOESM9_ESM.pdf]

Table S2. Goodness of fit of the EVD to the distribution of alignment scores of real unrelated profiles

Distribution	N	Location		Scale		AD_{up}	p-value
		Estimate	SE	Estimate	SE		
λ01.0: l100 vs. l100	5677	10.516	0.0300	1.905	0.0274	42.77	0.61
λ01.0: l200 vs. l100	8794	11.331	0.0256	2.046	0.0237	53.27	0.52
λ01.0: l200 vs. l200	4356	11.869	0.0376	2.348	0.0396	101.45	0.14
λ01.0: l400 vs. l100	2648	11.737	0.0447	1.844	0.0384	18.86	0.97
λ01.0: l400 vs. l200	2986	12.369	0.0438	2.034	0.0405	341.33	0.01
λ01.1: l100 vs. l100	20706	9.885	0.0142	1.644	0.0123	407.24	0.08
λ01.1: l200 vs. l100	42426	10.495	0.0099	1.686	0.0088	169.39	0.30
λ01.1: l200 vs. l200	27478	10.882	0.0125	1.739	0.0114	1434.53	0.06
λ01.1: l400 vs. l100	20357	11.148	0.0142	1.627	0.0122	284.93	0.16
λ01.1: l400 vs. l200	30058	11.560	0.0115	1.656	0.0103	2515.66	0.02
λ01.1: l400 vs. l400	6499	12.200	0.0248	1.684	0.0227	121.40	0.18
λ01.1: l600 vs. l100	4946	11.605	0.0277	1.536	0.0232	45.50	0.34
λ01.1: l600 vs. l200	7448	12.092	0.0233	1.641	0.0204	61.81	0.40
λ01.1: l600 vs. l400	2304	12.786	0.0405	1.628	0.0366	25.10	0.70
λ01.2: l100 vs. l100	35242	9.728	0.0104	1.581	0.0090	966.95	0.04
λ01.2: l200 vs. l100	78322	10.265	0.0071	1.632	0.0063	283.73	0.28
λ01.2: l200 vs. l200	55282	10.509	0.0084	1.666	0.0077	485.65	0.13
λ01.2: l400 vs. l100	33747	10.775	0.0106	1.584	0.0092	280.38	0.22
λ01.2: l400 vs. l200	55192	11.024	0.0081	1.573	0.0072	471.59	0.12
λ01.2: l400 vs. l400	11454	11.522	0.0171	1.526	0.0154	128.46	0.28
λ01.2: l600 vs. l100	7003	11.068	0.0231	1.502	0.0190	50.60	0.63
λ01.2: l600 vs. l200	12349	11.398	0.0168	1.531	0.0148	499.55	0.03
λ01.2: l600 vs. l400	3586	11.968	0.0289	1.502	0.0272	43.11	0.30
λ01.3: l100 vs. l100	24728	9.700	0.0122	1.530	0.0104	105.36	0.37
λ01.3: l200 vs. l100	61615	10.203	0.0081	1.649	0.0071	128.21	0.74
λ01.3: l200 vs. l200	44754	10.398	0.0093	1.677	0.0086	115.37	0.63
λ01.3: l400 vs. l100	22477	10.590	0.0132	1.626	0.0116	89.03	0.52
λ01.3: l400 vs. l200	37284	10.708	0.0097	1.591	0.0089	118.01	0.57
λ01.3: l400 vs. l400	6810	11.090	0.0221	1.550	0.0204	517.80	0.04
λ01.3: l600 vs. l100	4095	10.697	0.0282	1.497	0.0250	37.10	0.53
λ01.3: l600 vs. l200	7858	10.846	0.0206	1.492	0.0181	218.35	0.15
λ01.3: l600 vs. l400	1943	11.371	0.0407	1.552	0.0383	93.40	0.15
λ01.4: l100 vs. l100	8956	9.656	0.0193	1.466	0.0165	67.07	0.39
λ01.4: l200 vs. l100	24123	10.145	0.0128	1.626	0.0112	191.29	0.17
λ01.4: l200 vs. l200	18049	10.369	0.0145	1.694	0.0137	185.44	0.18
λ01.4: l400 vs. l100	8821	10.485	0.0209	1.648	0.0188	69.08	0.31
λ01.4: l400 vs. l200	14253	10.557	0.0158	1.646	0.0150	49.60	0.89
λ01.4: l400 vs. l400	2350	10.832	0.0363	1.543	0.0346	20.65	0.88
λ01.4: l600 vs. l200	2639	10.401	0.0353	1.510	0.0317	38.73	0.40
λ01.5: l100 vs. l100	2673	9.640	0.0349	1.400	0.0286	74.54	0.15
λ01.5: l200 vs. l100	7003	10.106	0.0232	1.621	0.0208	690.94	0.04

λ01.5: l200 vs. l200	5140	10.267	0.0280	1.753	0.0265	136.12	0.17
λ01.5: l400 vs. l100	2674	10.362	0.0390	1.675	0.0347	32.14	0.51
λ01.5: l400 vs. l200	4217	10.332	0.0290	1.674	0.0281	43.61	0.36
λ01.6: l200 vs. l100	2032	10.205	0.0438	1.656	0.0393	45.17	0.27

Distribution represents distributions obtained from aligning pairs of real profiles with mutual compositional similarity λ and different values of length l. The table reports the estimates and their standard errors (SE) for the location and scale parameters of the EVD for each distribution of alignment scores. N is the number of alignment scores. AD_{up} is the upper-tail Anderson-Darling statistic. The p-value of statistic AD_{up} was computed by Monte Carlo simulation with 100 samples.
