# Supplementary material for: Estimating statistical significance of local protein profile-profile alignments
Source: BMC Bioinformatics. 2019 Aug 13;20:419. doi: 10.1186/s12859-019-2913-3 (PMC6693267; doi:10.1186/s12859-019-2913-3)
Supplement: Supplementary file 10 — Table S3. Goodness of fit of the EVD to the distribution of alignment scores of profiles generated using S=1012 seed profiles with s=9 and r=0.03. (PDF 65 kb) [file 12859_2019_2913_MOESM10_ESM.pdf]

Table S3. Goodness of fit of the EVD to the distribution of alignment scores of profiles generated using $S = 1012$ seed profiles with $s = 9$ and $r = 0.03$

Distribution	N	Location		Scale		AD_{up}	p-value
		Estimate	SE	Estimate	SE		
n04 l050 vs. n02 l050	9385	7.991	0.0158	1.169	0.0128	46.98	0.87
n04 l050 vs. n02 l100	12564	8.884	0.0141	1.270	0.0121	121.68	0.30
n04 l050 vs. n02 l200	11910	9.892	0.0151	1.319	0.0129	74.14	0.48
n04 l050 vs. n02 l400	9902	11.026	0.0163	1.332	0.0143	51.98	0.72
n04 l050 vs. n02 l600	8559	11.720	0.0181	1.380	0.0159	103.34	0.18
n04 l050 vs. n02 l800	7718	12.172	0.0200	1.370	0.0166	59.29	0.41
n04 l100 vs. n02 l050	12109	8.810	0.0142	1.245	0.0121	76.72	0.43
n04 l100 vs. n02 l100	25308	9.182	0.0108	1.378	0.0093	93.91	0.55
n04 l100 vs. n02 l200	31604	9.999	0.0098	1.444	0.0088	107.39	0.53
n04 l100 vs. n02 l400	30651	11.030	0.0104	1.507	0.0093	94.43	0.67
n04 l100 vs. n02 l600	28457	11.696	0.0110	1.555	0.0099	105.69	0.45
n04 l100 vs. n02 l800	26709	12.146	0.0114	1.571	0.0104	98.53	0.53
n04 l200 vs. n02 l050	12060	9.668	0.0151	1.306	0.0127	65.68	0.48
n04 l200 vs. n02 l100	32556	9.891	0.0099	1.455	0.0087	104.77	0.60
n04 l200 vs. n02 l200	48745	10.572	0.0082	1.522	0.0075	114.34	0.65
n04 l200 vs. n02 l400	51556	11.643	0.0081	1.577	0.0076	100.87	0.85
n04 l200 vs. n02 l600	49377	12.340	0.0085	1.608	0.0079	154.16	0.44
n04 l200 vs. n02 l800	47628	12.785	0.0087	1.639	0.0082	142.37	0.47
n04 l400 vs. n02 l050	11639	10.509	0.0161	1.403	0.0139	62.21	0.58
n04 l400 vs. n02 l100	35532	10.830	0.0097	1.546	0.0089	93.10	0.77
n04 l400 vs. n02 l200	54148	11.659	0.0081	1.619	0.0076	117.13	0.77
n04 l400 vs. n02 l400	60074	12.800	0.0080	1.700	0.0076	117.55	0.87
n04 l400 vs. n02 l600	60056	13.527	0.0081	1.740	0.0077	120.87	0.76
n04 l400 vs. n02 l800	59238	14.026	0.0083	1.773	0.0079	170.53	0.31
n04 l600 vs. n02 l050	10457	11.106	0.0179	1.462	0.0153	83.12	0.40
n04 l600 vs. n02 l100	34905	11.473	0.0100	1.599	0.0093	113.94	0.49
n04 l600 vs. n02 l200	54673	12.424	0.0083	1.704	0.0080	162.57	0.44
n04 l600 vs. n02 l400	61190	13.627	0.0082	1.775	0.0078	168.90	0.48
n04 l600 vs. n02 l600	61900	14.414	0.0084	1.841	0.0081	155.49	0.50
n04 l600 vs. n02 l800	61797	14.939	0.0085	1.860	0.0081	156.66	0.47
n04 l800 vs. n02 l050	9514	11.596	0.0183	1.464	0.0160	54.90	0.68
n04 l800 vs. n02 l100	32887	11.910	0.0105	1.650	0.0099	113.34	0.46
n04 l800 vs. n02 l200	53404	12.892	0.0086	1.741	0.0082	83.79	0.96
n04 l800 vs. n02 l400	61169	14.155	0.0084	1.836	0.0081	168.23	0.33
n04 l800 vs. n02 l600	62130	14.976	0.0085	1.895	0.0083	167.36	0.44
n04 l800 vs. n02 l800	62260	15.518	0.0086	1.935	0.0085	173.21	0.43
n06 l050 vs. n02 l050	5248	7.475	0.0196	1.147	0.0169	42.37	0.65
n06 l050 vs. n02 l100	7527	8.224	0.0179	1.230	0.0152	44.54	0.74
n06 l050 vs. n02 l200	7075	9.340	0.0191	1.331	0.0170	53.13	0.50
n06 l050 vs. n02 l400	5876	10.451	0.0218	1.376	0.0193	44.14	0.55
n06 l050 vs. n02 l600	5083	11.182	0.0237	1.408	0.0212	25.88	0.98

continues ...

(continued)

Distribution	N	Location		Scale		AD_{up}	p-value
		Estimate	SE	Estimate	SE		
n06 l050 vs. n02 l800	4720	11.679	0.0249	1.429	0.0223	31.56	0.80
n06 l050 vs. n04 l050	3327	7.330	0.0272	1.361	0.0258	78.44	0.20
n06 l050 vs. n04 l100	4486	8.108	0.0263	1.565	0.0256	32.67	0.69
n06 l050 vs. n04 l200	5022	9.001	0.0258	1.671	0.0259	26.11	0.97
n06 l050 vs. n04 l400	5338	9.973	0.0270	1.803	0.0270	42.27	0.59
n06 l050 vs. n04 l600	5661	10.698	0.0275	1.831	0.0263	53.93	0.40
n06 l050 vs. n04 l800	5074	11.240	0.0297	1.871	0.0284	44.03	0.46
n06 l100 vs. n02 l050	6534	8.290	0.0188	1.263	0.0168	53.32	0.45
n06 l100 vs. n02 l100	17164	8.395	0.0121	1.353	0.0112	72.48	0.61
n06 l100 vs. n02 l200	23182	9.093	0.0110	1.429	0.0102	139.24	0.27
n06 l100 vs. n02 l400	22167	10.077	0.0114	1.481	0.0108	59.19	0.95
n06 l100 vs. n02 l600	20251	10.758	0.0125	1.551	0.0118	83.31	0.55
n06 l100 vs. n02 l800	18797	11.166	0.0134	1.597	0.0126	83.42	0.48
n06 l100 vs. n04 l050	4252	8.251	0.0261	1.530	0.0257	29.25	0.87
n06 l100 vs. n04 l100	10352	8.172	0.0172	1.595	0.0173	67.86	0.45
n06 l100 vs. n04 l200	15830	8.676	0.0144	1.685	0.0148	416.98	0.07
n06 l100 vs. n04 l400	19153	9.445	0.0140	1.834	0.0147	208.91	0.21
n06 l100 vs. n04 l600	19962	10.149	0.0143	1.902	0.0148	79.43	0.53
n06 l100 vs. n04 l800	18603	10.573	0.0153	1.991	0.0161	115.67	0.40
n06 l200 vs. n02 l050	5611	9.281	0.0215	1.273	0.0181	35.83	0.85
n06 l200 vs. n02 l100	20569	9.099	0.0115	1.375	0.0104	66.75	0.78
n06 l200 vs. n02 l200	39529	9.449	0.0081	1.416	0.0078	79.07	0.95
n06 l200 vs. n02 l400	44253	10.336	0.0078	1.433	0.0074	130.43	0.50
n06 l200 vs. n02 l600	41553	10.975	0.0083	1.485	0.0080	126.21	0.53
n06 l200 vs. n02 l800	39362	11.383	0.0087	1.513	0.0083	97.91	0.78
n06 l200 vs. n04 l050	3933	9.326	0.0292	1.628	0.0283	26.99	0.93
n06 l200 vs. n04 l100	12705	8.838	0.0163	1.702	0.0167	78.43	0.45
n06 l200 vs. n04 l200	27641	8.873	0.0103	1.635	0.0110	182.51	0.22
n06 l200 vs. n04 l400	37077	9.634	0.0092	1.707	0.0099	495.88	0.06
n06 l200 vs. n04 l600	38551	10.294	0.0095	1.788	0.0101	109.67	0.62
n06 l200 vs. n04 l800	37098	10.687	0.0100	1.836	0.0106	187.49	0.40
n06 l400 vs. n02 l050	4663	10.302	0.0244	1.395	0.0219	40.15	0.52
n06 l400 vs. n02 l100	19508	10.031	0.0125	1.510	0.0118	76.71	0.72
n06 l400 vs. n02 l200	42382	10.414	0.0083	1.513	0.0081	219.80	0.24
n06 l400 vs. n02 l400	56690	11.192	0.0071	1.501	0.0069	139.78	0.50
n06 l400 vs. n02 l600	57760	11.794	0.0072	1.531	0.0070	160.85	0.53
n06 l400 vs. n02 l800	56482	12.227	0.0074	1.540	0.0071	157.09	0.40
n06 l400 vs. n04 l050	3999	10.651	0.0313	1.773	0.0305	39.79	0.41
n06 l400 vs. n04 l100	13619	9.926	0.0174	1.899	0.0179	80.61	0.36
n06 l400 vs. n04 l200	31859	9.868	0.0105	1.809	0.0113	115.26	0.42
n06 l400 vs. n04 l400	49071	10.552	0.0085	1.819	0.0092	154.31	0.44
n06 l400 vs. n04 l600	53901	11.243	0.0086	1.910	0.0091	116.21	0.72
n06 l400 vs. n04 l800	54042	11.630	0.0087	1.938	0.0092	141.35	0.56

continues ...

(continued)

Distribution	N	Location		Scale		AD_{up}	p-value
		Estimate	SE	Estimate	SE		
n06 l600 vs. n02 l050	4328	10.926	0.0264	1.439	0.0235	43.27	0.43
n06 l600 vs. n02 l100	18816	10.583	0.0129	1.591	0.0127	92.96	0.36
n06 l600 vs. n02 l200	41200	11.025	0.0087	1.577	0.0085	144.60	0.35
n06 l600 vs. n02 l400	57142	11.833	0.0073	1.552	0.0071	126.20	0.74
n06 l600 vs. n02 l600	60615	12.425	0.0071	1.567	0.0070	180.46	0.42
n06 l600 vs. n02 l800	60771	12.867	0.0073	1.599	0.0071	116.30	0.85
n06 l600 vs. n04 l050	3983	11.320	0.0337	1.874	0.0322	42.04	0.38
n06 l600 vs. n04 l100	13921	10.496	0.0180	1.993	0.0186	85.61	0.36
n06 l600 vs. n04 l200	32198	10.477	0.0110	1.933	0.0121	125.86	0.44
n06 l600 vs. n04 l400	49813	11.248	0.0088	1.896	0.0095	128.03	0.55
n06 l600 vs. n04 l600	56550	11.975	0.0087	1.983	0.0093	155.55	0.42
n06 l600 vs. n04 l800	58443	12.367	0.0086	2.004	0.0092	154.18	0.44
n06 l800 vs. n02 l050	4002	11.329	0.0283	1.532	0.0261	35.24	0.66
n06 l800 vs. n02 l100	18690	10.919	0.0134	1.588	0.0126	100.81	0.39
n06 l800 vs. n02 l200	40608	11.428	0.0090	1.614	0.0088	138.31	0.40
n06 l800 vs. n02 l400	56171	12.274	0.0075	1.576	0.0073	227.58	0.38
n06 l800 vs. n02 l600	60693	12.877	0.0073	1.587	0.0070	183.24	0.36
n06 l800 vs. n02 l800	61603	13.302	0.0073	1.616	0.0071	151.42	0.51
n06 l800 vs. n04 l050	4056	11.625	0.0330	1.809	0.0306	46.73	0.42
n06 l800 vs. n04 l100	14440	10.842	0.0181	2.030	0.0185	88.13	0.43
n06 l800 vs. n04 l200	32501	10.883	0.0112	1.945	0.0120	131.54	0.36
n06 l800 vs. n04 l400	49631	11.751	0.0091	1.927	0.0096	158.04	0.38
n06 l800 vs. n04 l600	56800	12.490	0.0088	1.947	0.0090	160.15	0.49
n06 l800 vs. n04 l800	59207	12.886	0.0087	1.955	0.0088	177.25	0.32
n08 l050 vs. n02 l050	4034	7.201	0.0224	1.139	0.0193	80.26	0.17
n08 l050 vs. n02 l100	5807	7.964	0.0199	1.277	0.0181	101.60	0.17
n08 l050 vs. n02 l200	5604	9.042	0.0222	1.423	0.0206	40.69	0.63
n08 l050 vs. n02 l400	4691	10.158	0.0242	1.467	0.0233	23.99	0.99
n08 l050 vs. n02 l600	4131	10.926	0.0271	1.514	0.0256	38.56	0.55
n08 l050 vs. n02 l800	3729	11.464	0.0294	1.516	0.0267	33.32	0.62
n08 l050 vs. n04 l050	2805	7.253	0.0292	1.446	0.0303	238.04	0.06
n08 l050 vs. n04 l100	3908	7.924	0.0276	1.596	0.0282	38.60	0.46
n08 l050 vs. n04 l200	4380	8.793	0.0295	1.825	0.0306	58.83	0.40
n08 l050 vs. n04 l400	4613	9.810	0.0304	1.913	0.0309	26.62	0.90
n08 l050 vs. n04 l600	4938	10.643	0.0310	1.964	0.0305	47.87	0.44
n08 l050 vs. n04 l800	4480	11.218	0.0345	2.004	0.0323	36.44	0.72
n08 l050 vs. n06 l050	2867	7.219	0.0326	1.706	0.0358	63.81	0.20
n08 l050 vs. n06 l100	4378	8.085	0.0296	1.874	0.0315	344.21	0.04
n08 l050 vs. n06 l200	4483	9.209	0.0308	1.909	0.0314	82.44	0.31
n08 l050 vs. n06 l400	4923	10.575	0.0313	1.983	0.0308	33.59	0.81
n08 l050 vs. n06 l600	5211	11.304	0.0320	2.084	0.0315	43.13	0.68
n08 l050 vs. n06 l800	5388	11.727	0.0309	1.964	0.0288	42.38	0.64
n08 l100 vs. n02 l050	4939	7.854	0.0214	1.220	0.0186	28.59	0.94

continues . . .

(continued)

Distribution	N	Location		Scale		AD_{up}	p-value
		Estimate	SE	Estimate	SE		
n08 l100 vs. n02 l100	13764	7.921	0.0134	1.352	0.0125	78.09	0.42
n08 l100 vs. n02 l200	18807	8.573	0.0120	1.420	0.0113	64.61	0.80
n08 l100 vs. n02 l400	17111	9.583	0.0131	1.496	0.0125	68.64	0.73
n08 l100 vs. n02 l600	15345	10.257	0.0143	1.564	0.0137	66.08	0.70
n08 l100 vs. n02 l800	14048	10.697	0.0152	1.576	0.0144	77.44	0.49
n08 l100 vs. n04 l050	3645	7.974	0.0285	1.575	0.0287	26.85	0.80
n08 l100 vs. n04 l100	9014	7.787	0.0188	1.682	0.0197	40.64	0.90
n08 l100 vs. n04 l200	13402	8.302	0.0157	1.734	0.0167	63.10	0.56
n08 l100 vs. n04 l400	15747	9.174	0.0157	1.883	0.0166	159.78	0.25
n08 l100 vs. n04 l600	16250	9.876	0.0163	1.966	0.0170	63.75	0.75
n08 l100 vs. n04 l800	14876	10.328	0.0178	2.023	0.0182	63.20	0.69
n08 l100 vs. n06 l050	4240	7.868	0.0291	1.818	0.0310	44.62	0.52
n08 l100 vs. n06 l100	10546	7.746	0.0193	1.910	0.0207	115.68	0.30
n08 l100 vs. n06 l200	13602	8.436	0.0173	1.948	0.0186	65.08	0.59
n08 l100 vs. n06 l400	14576	9.677	0.0185	2.079	0.0188	92.80	0.41
n08 l100 vs. n06 l600	14593	10.373	0.0193	2.139	0.0193	80.58	0.39
n08 l100 vs. n06 l800	15408	10.858	0.0192	2.150	0.0188	90.24	0.33
n08 l200 vs. n02 l050	4967	8.733	0.0238	1.366	0.0208	29.81	0.98
n08 l200 vs. n02 l100	18948	8.499	0.0118	1.417	0.0112	89.05	0.39
n08 l200 vs. n02 l200	34763	8.917	0.0087	1.453	0.0086	69.73	0.95
n08 l200 vs. n02 l400	37416	9.827	0.0086	1.509	0.0086	103.36	0.63
n08 l200 vs. n02 l600	34391	10.487	0.0094	1.586	0.0094	209.34	0.25
n08 l200 vs. n02 l800	32029	10.920	0.0099	1.630	0.0100	102.23	0.62
n08 l200 vs. n04 l050	4041	8.944	0.0306	1.775	0.0306	41.40	0.48
n08 l200 vs. n04 l100	13282	8.400	0.0162	1.787	0.0173	77.29	0.40
n08 l200 vs. n04 l200	25989	8.532	0.0110	1.765	0.0123	85.37	0.70
n08 l200 vs. n04 l400	32222	9.426	0.0106	1.882	0.0118	108.45	0.49
n08 l200 vs. n04 l600	33615	10.113	0.0112	2.013	0.0123	132.39	0.36
n08 l200 vs. n04 l800	31928	10.523	0.0117	2.053	0.0128	129.09	0.40
n08 l200 vs. n06 l050	4935	9.000	0.0298	1.961	0.0308	38.87	0.61
n08 l200 vs. n06 l100	16109	8.384	0.0164	2.054	0.0182	62.15	0.69
n08 l200 vs. n06 l200	27981	8.497	0.0117	1.973	0.0133	82.85	0.70
n08 l200 vs. n06 l400	31622	9.716	0.0123	2.160	0.0136	125.51	0.40
n08 l200 vs. n06 l600	31266	10.516	0.0131	2.274	0.0143	129.64	0.39
n08 l200 vs. n06 l800	31776	11.023	0.0135	2.268	0.0140	132.48	0.37
n08 l400 vs. n02 l050	4759	9.471	0.0246	1.480	0.0233	43.55	0.41
n08 l400 vs. n02 l100	20264	9.303	0.0119	1.512	0.0117	67.76	0.81
n08 l400 vs. n02 l200	41181	9.775	0.0084	1.559	0.0085	120.26	0.61
n08 l400 vs. n02 l400	51869	10.597	0.0074	1.554	0.0075	161.31	0.46
n08 l400 vs. n02 l600	51414	11.226	0.0078	1.589	0.0077	158.86	0.35
n08 l400 vs. n02 l800	49319	11.678	0.0080	1.615	0.0080	139.36	0.46
n08 l400 vs. n04 l050	4702	9.908	0.0313	1.979	0.0316	49.91	0.31
n08 l400 vs. n04 l100	16512	9.215	0.0153	1.912	0.0166	92.36	0.38

continues ...

(continued)

Distribution	N	Location		Scale		AD_{up}	p-value
		Estimate	SE	Estimate	SE		
n08 l400 vs. n04 l200	33598	9.358	0.0101	1.842	0.0113	99.69	0.64
n08 l400 vs. n04 l400	45229	10.269	0.0093	1.937	0.0102	143.98	0.39
n08 l400 vs. n04 l600	48126	11.031	0.0096	2.031	0.0103	164.95	0.36
n08 l400 vs. n04 l800	47798	11.443	0.0098	2.040	0.0103	164.60	0.37
n08 l400 vs. n06 l050	6002	10.070	0.0292	2.074	0.0293	52.38	0.35
n08 l400 vs. n06 l100	19035	9.404	0.0161	2.131	0.0171	88.04	0.46
n08 l400 vs. n06 l200	35336	9.431	0.0111	2.084	0.0124	124.22	0.45
n08 l400 vs. n06 l400	46954	10.518	0.0104	2.212	0.0113	163.31	0.36
n08 l400 vs. n06 l600	47662	11.364	0.0108	2.278	0.0116	154.73	0.40
n08 l400 vs. n06 l800	47746	11.972	0.0110	2.231	0.0112	168.82	0.38
n08 l600 vs. n02 l050	4224	10.122	0.0269	1.519	0.0253	43.06	0.49
n08 l600 vs. n02 l100	18734	9.863	0.0127	1.570	0.0126	121.09	0.34
n08 l600 vs. n02 l200	40281	10.355	0.0086	1.597	0.0088	109.31	0.67
n08 l600 vs. n02 l400	54849	11.177	0.0074	1.582	0.0074	126.00	0.66
n08 l600 vs. n02 l600	57671	11.773	0.0073	1.606	0.0074	177.35	0.36
n08 l600 vs. n02 l800	57225	12.198	0.0075	1.617	0.0074	123.81	0.72
n08 l600 vs. n04 l050	4610	10.661	0.0314	1.958	0.0316	49.60	0.46
n08 l600 vs. n04 l100	16216	9.868	0.0163	1.994	0.0174	89.02	0.45
n08 l600 vs. n04 l200	34280	9.990	0.0106	1.912	0.0115	133.36	0.43
n08 l600 vs. n04 l400	49109	10.875	0.0092	1.962	0.0098	127.79	0.55
n08 l600 vs. n04 l600	53520	11.657	0.0092	2.037	0.0097	169.16	0.40
n08 l600 vs. n04 l800	54446	12.062	0.0093	2.040	0.0096	142.83	0.54
n08 l600 vs. n06 l050	6287	10.726	0.0282	2.025	0.0278	60.22	0.42
n08 l600 vs. n06 l100	19436	10.160	0.0163	2.146	0.0169	92.49	0.46
n08 l600 vs. n06 l200	35461	10.176	0.0114	2.093	0.0123	127.38	0.41
n08 l600 vs. n06 l400	51170	11.191	0.0101	2.171	0.0106	168.20	0.47
n08 l600 vs. n06 l600	54751	11.959	0.0100	2.220	0.0104	176.75	0.36
n08 l600 vs. n06 l800	55297	12.583	0.0101	2.163	0.0100	177.39	0.34
n08 l800 vs. n02 l050	3801	10.679	0.0295	1.588	0.0280	34.45	0.59
n08 l800 vs. n02 l100	16811	10.381	0.0141	1.661	0.0141	71.51	0.54
n08 l800 vs. n02 l200	37360	10.828	0.0094	1.671	0.0095	124.57	0.52
n08 l800 vs. n02 l400	55096	11.590	0.0076	1.615	0.0076	164.28	0.47
n08 l800 vs. n02 l600	59086	12.205	0.0074	1.653	0.0075	148.63	0.49
n08 l800 vs. n02 l800	59997	12.637	0.0074	1.655	0.0074	146.00	0.57
n08 l800 vs. n04 l050	4612	11.134	0.0326	1.946	0.0310	50.91	0.37
n08 l800 vs. n04 l100	15478	10.425	0.0175	2.060	0.0183	92.33	0.33
n08 l800 vs. n04 l200	32262	10.510	0.0114	1.990	0.0123	121.43	0.36
n08 l800 vs. n04 l400	49720	11.339	0.0094	2.011	0.0100	145.59	0.46
n08 l800 vs. n04 l600	55360	12.157	0.0092	2.040	0.0095	153.94	0.46
n08 l800 vs. n04 l800	57031	12.548	0.0093	2.063	0.0095	169.54	0.34
n08 l800 vs. n06 l050	6349	11.273	0.0287	2.032	0.0277	51.11	0.44
n08 l800 vs. n06 l100	18962	10.773	0.0173	2.192	0.0174	90.46	0.45
n08 l800 vs. n06 l200	34291	10.773	0.0122	2.142	0.0128	126.49	0.42

continues . . .

(continued)

Distribution	N	Location		Scale		AD_{up}	p-value
		Estimate	SE	Estimate	SE		
n08 l800 vs. n06 l400	52083	11.700	0.0103	2.177	0.0104	164.65	0.40
n08 l800 vs. n06 l600	57046	12.476	0.0101	2.238	0.0103	155.28	0.46
n08 l800 vs. n06 l800	58127	13.114	0.0101	2.167	0.0097	176.38	0.41
n10 l050 vs. n02 l050	3475	7.052	0.0250	1.238	0.0228	169.52	0.11
n10 l050 vs. n02 l100	5216	7.780	0.0221	1.350	0.0202	73.14	0.30
n10 l050 vs. n02 l200	5312	8.989	0.0245	1.612	0.0243	51.55	0.42
n10 l050 vs. n02 l400	4693	10.189	0.0260	1.557	0.0246	44.19	0.43
n10 l050 vs. n02 l600	4315	10.994	0.0276	1.569	0.0259	46.58	0.42
n10 l050 vs. n02 l800	4169	11.440	0.0269	1.527	0.0256	47.34	0.35
n10 l050 vs. n04 l050	2976	7.150	0.0321	1.622	0.0329	34.74	0.47
n10 l050 vs. n04 l100	4357	8.024	0.0289	1.811	0.0305	38.84	0.54
n10 l050 vs. n04 l200	4915	8.890	0.0289	1.818	0.0284	40.73	0.57
n10 l050 vs. n04 l400	5785	10.077	0.0280	1.915	0.0274	56.36	0.34
n10 l050 vs. n04 l600	6614	10.720	0.0264	1.905	0.0254	59.31	0.46
n10 l050 vs. n04 l800	6193	11.318	0.0279	1.854	0.0253	55.96	0.33
n10 l050 vs. n06 l050	2911	7.252	0.0350	1.836	0.0380	28.84	0.65
n10 l050 vs. n06 l100	4445	8.217	0.0312	1.927	0.0316	43.67	0.48
n10 l050 vs. n06 l200	4798	9.381	0.0310	1.943	0.0307	45.84	0.39
n10 l050 vs. n06 l400	5794	10.736	0.0284	1.901	0.0271	57.39	0.41
n10 l050 vs. n06 l600	6422	11.370	0.0274	1.950	0.0265	61.48	0.38
n10 l050 vs. n06 l800	7087	11.724	0.0259	1.861	0.0237	63.72	0.45
n10 l050 vs. n08 l050	2814	7.269	0.0379	1.937	0.0406	34.26	0.41
n10 l050 vs. n08 l100	4273	8.047	0.0335	2.039	0.0343	46.74	0.41
n10 l050 vs. n08 l200	5433	9.167	0.0310	2.036	0.0300	52.57	0.32
n10 l050 vs. n08 l400	7336	10.277	0.0273	2.106	0.0269	57.35	0.51
n10 l050 vs. n08 l600	7970	10.891	0.0258	2.046	0.0249	66.33	0.47
n10 l050 vs. n08 l800	8393	11.466	0.0252	2.020	0.0239	70.57	0.35
n10 l100 vs. n02 l050	5295	7.502	0.0219	1.337	0.0199	28.84	0.94
n10 l100 vs. n02 l100	13773	7.570	0.0136	1.390	0.0129	74.80	0.44
n10 l100 vs. n02 l200	17407	8.448	0.0130	1.579	0.0132	64.00	0.74
n10 l100 vs. n02 l400	16105	9.492	0.0146	1.659	0.0143	85.24	0.46
n10 l100 vs. n02 l600	14592	10.270	0.0157	1.722	0.0156	89.02	0.37
n10 l100 vs. n02 l800	13701	10.721	0.0165	1.702	0.0158	83.21	0.33
n10 l100 vs. n04 l050	4526	7.671	0.0287	1.753	0.0286	46.25	0.35
n10 l100 vs. n04 l100	10830	7.598	0.0178	1.765	0.0189	68.87	0.45
n10 l100 vs. n04 l200	14802	8.270	0.0159	1.888	0.0173	55.68	0.80
n10 l100 vs. n04 l400	16630	9.283	0.0162	2.012	0.0173	78.93	0.49
n10 l100 vs. n04 l600	17466	10.107	0.0169	2.068	0.0171	92.78	0.37
n10 l100 vs. n04 l800	16013	10.595	0.0180	2.092	0.0180	94.11	0.46
n10 l100 vs. n06 l050	4507	7.774	0.0312	1.997	0.0330	66.61	0.28
n10 l100 vs. n06 l100	10488	7.711	0.0204	2.021	0.0219	200.67	0.20
n10 l100 vs. n06 l200	13017	8.529	0.0192	2.087	0.0202	56.90	0.79
n10 l100 vs. n06 l400	14823	9.935	0.0197	2.166	0.0193	89.03	0.36

continues ...

(continued)

Distribution	N	Location		Scale		AD_{up}	p-value
		Estimate	SE	Estimate	SE		
n10 l100 vs. n06 l600	15620	10.671	0.0200	2.222	0.0192	91.11	0.43
n10 l100 vs. n06 l800	16514	11.145	0.0192	2.131	0.0178	87.98	0.44
n10 l100 vs. n08 l050	4573	7.670	0.0332	2.123	0.0347	139.04	0.11
n10 l100 vs. n08 l100	10205	7.553	0.0211	2.081	0.0229	70.24	0.35
n10 l100 vs. n08 l200	14369	8.379	0.0190	2.211	0.0205	76.82	0.40
n10 l100 vs. n08 l400	17369	9.570	0.0183	2.246	0.0187	82.71	0.41
n10 l100 vs. n08 l600	18502	10.343	0.0179	2.192	0.0175	99.69	0.43
n10 l100 vs. n08 l800	18647	10.945	0.0182	2.194	0.0174	96.79	0.51
n10 l200 vs. n02 l050	5057	8.294	0.0237	1.490	0.0229	46.21	0.39
n10 l200 vs. n02 l100	19281	8.094	0.0115	1.453	0.0115	63.07	0.80
n10 l200 vs. n02 l200	33290	8.586	0.0090	1.547	0.0094	83.38	0.85
n10 l200 vs. n02 l400	33446	9.579	0.0094	1.602	0.0097	90.34	0.77
n10 l200 vs. n02 l600	30849	10.329	0.0104	1.706	0.0107	130.88	0.39
n10 l200 vs. n02 l800	28556	10.774	0.0111	1.761	0.0115	114.07	0.44
n10 l200 vs. n04 l050	4746	8.628	0.0294	1.923	0.0309	43.67	0.53
n10 l200 vs. n04 l100	15849	8.051	0.0148	1.860	0.0166	78.41	0.55
n10 l200 vs. n04 l200	27894	8.359	0.0110	1.850	0.0125	93.42	0.59
n10 l200 vs. n04 l400	31196	9.452	0.0115	2.028	0.0129	105.28	0.54
n10 l200 vs. n04 l600	32415	10.307	0.0121	2.129	0.0132	131.12	0.39
n10 l200 vs. n04 l800	30366	10.734	0.0129	2.168	0.0138	112.22	0.49
n10 l200 vs. n06 l050	5364	8.771	0.0306	2.075	0.0311	57.78	0.31
n10 l200 vs. n06 l100	16016	8.189	0.0170	2.137	0.0189	139.40	0.30
n10 l200 vs. n06 l200	26155	8.360	0.0128	2.081	0.0145	91.85	0.52
n10 l200 vs. n06 l400	29197	9.739	0.0136	2.245	0.0145	117.36	0.46
n10 l200 vs. n06 l600	29496	10.588	0.0144	2.360	0.0152	122.51	0.35
n10 l200 vs. n06 l800	30442	11.145	0.0145	2.310	0.0144	133.01	0.42
n10 l200 vs. n08 l050	5327	8.741	0.0319	2.180	0.0330	196.92	0.08
n10 l200 vs. n08 l100	14796	8.069	0.0180	2.155	0.0198	82.90	0.36
n10 l200 vs. n08 l200	25583	8.400	0.0141	2.287	0.0161	81.34	0.75
n10 l200 vs. n08 l400	30425	9.635	0.0139	2.353	0.0150	125.46	0.40
n10 l200 vs. n08 l600	31874	10.472	0.0138	2.306	0.0142	134.10	0.43
n10 l200 vs. n08 l800	31972	11.124	0.0140	2.329	0.0143	112.54	0.47
n10 l400 vs. n02 l050	4205	9.401	0.0287	1.616	0.0270	44.09	0.39
n10 l400 vs. n02 l100	17668	8.988	0.0130	1.602	0.0134	90.82	0.49
n10 l400 vs. n02 l200	38678	9.382	0.0088	1.640	0.0093	133.67	0.48
n10 l400 vs. n02 l400	50195	10.199	0.0078	1.614	0.0080	140.80	0.48
n10 l400 vs. n02 l600	49469	10.867	0.0081	1.670	0.0083	168.46	0.32
n10 l400 vs. n02 l800	46779	11.319	0.0085	1.707	0.0087	147.07	0.34
n10 l400 vs. n04 l050	4654	9.932	0.0322	2.035	0.0327	50.95	0.32
n10 l400 vs. n04 l100	15894	9.148	0.0169	2.077	0.0184	93.97	0.38
n10 l400 vs. n04 l200	33620	9.190	0.0106	1.960	0.0120	94.39	0.73
n10 l400 vs. n04 l400	45459	10.113	0.0097	2.049	0.0107	155.40	0.33
n10 l400 vs. n04 l600	47515	10.975	0.0101	2.124	0.0108	154.53	0.37

continues ...

(continued)

Distribution	N	Location		Scale		AD_{up}	p-value
		Estimate	SE	Estimate	SE		
n10 l400 vs. n04 l800	47105	11.409	0.0103	2.106	0.0107	155.19	0.30
n10 l400 vs. n06 l050	5543	10.109	0.0311	2.091	0.0306	43.14	0.65
n10 l400 vs. n06 l100	17180	9.316	0.0177	2.202	0.0185	83.21	0.48
n10 l400 vs. n06 l200	33110	9.191	0.0120	2.181	0.0134	121.08	0.39
n10 l400 vs. n06 l400	45224	10.233	0.0111	2.268	0.0118	150.62	0.45
n10 l400 vs. n06 l600	45884	11.136	0.0114	2.311	0.0119	164.52	0.34
n10 l400 vs. n06 l800	46220	11.749	0.0116	2.260	0.0114	167.64	0.40
n10 l400 vs. n08 l050	5724	10.037	0.0320	2.174	0.0312	52.08	0.44
n10 l400 vs. n08 l100	16047	9.244	0.0190	2.255	0.0195	94.03	0.33
n10 l400 vs. n08 l200	31822	9.343	0.0133	2.346	0.0147	125.53	0.38
n10 l400 vs. n08 l400	43134	10.398	0.0121	2.409	0.0128	156.62	0.39
n10 l400 vs. n08 l600	46315	11.147	0.0117	2.339	0.0119	158.30	0.41
n10 l400 vs. n08 l800	47180	11.755	0.0118	2.358	0.0118	163.70	0.35
n10 l600 vs. n02 l050	3849	10.129	0.0301	1.630	0.0284	46.42	0.42
n10 l600 vs. n02 l100	16087	9.650	0.0146	1.734	0.0151	80.45	0.41
n10 l600 vs. n02 l200	36424	10.086	0.0097	1.744	0.0101	142.17	0.33
n10 l600 vs. n02 l400	54103	10.788	0.0077	1.671	0.0079	158.61	0.44
n10 l600 vs. n02 l600	57014	11.418	0.0077	1.712	0.0079	171.29	0.34
n10 l600 vs. n02 l800	56394	11.853	0.0078	1.705	0.0079	141.02	0.55
n10 l600 vs. n04 l050	4782	10.692	0.0321	1.973	0.0311	47.76	0.33
n10 l600 vs. n04 l100	15967	9.903	0.0179	2.161	0.0189	96.39	0.32
n10 l600 vs. n04 l200	32956	9.954	0.0115	2.057	0.0127	126.95	0.40
n10 l600 vs. n04 l400	49514	10.807	0.0096	2.070	0.0103	142.47	0.56
n10 l600 vs. n04 l600	54162	11.651	0.0097	2.111	0.0100	143.89	0.43
n10 l600 vs. n04 l800	54630	12.074	0.0097	2.126	0.0100	168.34	0.30
n10 l600 vs. n06 l050	6132	10.861	0.0296	2.028	0.0279	51.41	0.45
n10 l600 vs. n06 l100	18008	10.159	0.0182	2.214	0.0179	92.37	0.44
n10 l600 vs. n06 l200	33269	10.051	0.0127	2.207	0.0133	122.16	0.46
n10 l600 vs. n06 l400	50786	10.952	0.0106	2.237	0.0108	163.67	0.29
n10 l600 vs. n06 l600	54060	11.766	0.0106	2.296	0.0108	178.15	0.35
n10 l600 vs. n06 l800	54604	12.428	0.0105	2.199	0.0102	179.27	0.36
n10 l600 vs. n08 l050	6330	10.851	0.0300	2.079	0.0283	41.84	0.63
n10 l600 vs. n08 l100	16608	10.118	0.0192	2.246	0.0189	85.50	0.39
n10 l600 vs. n08 l200	32094	10.285	0.0139	2.351	0.0144	130.98	0.37
n10 l600 vs. n08 l400	47621	11.202	0.0117	2.378	0.0119	164.09	0.36
n10 l600 vs. n08 l600	52645	11.913	0.0110	2.314	0.0110	157.38	0.42
n10 l600 vs. n08 l800	54242	12.510	0.0110	2.323	0.0108	160.48	0.38
n10 l800 vs. n02 l050	3476	10.603	0.0329	1.702	0.0313	43.71	0.36
n10 l800 vs. n02 l100	14665	10.087	0.0157	1.787	0.0163	89.41	0.41
n10 l800 vs. n02 l200	33854	10.554	0.0103	1.808	0.0109	135.04	0.33
n10 l800 vs. n02 l400	53042	11.204	0.0079	1.699	0.0082	165.53	0.40
n10 l800 vs. n02 l600	58667	11.805	0.0076	1.700	0.0077	169.37	0.46
n10 l800 vs. n02 l800	59498	12.224	0.0076	1.712	0.0077	176.53	0.42

continues ...

(continued)

Distribution	N	Location		Scale		AD_{up}	p-value
		Estimate	SE	Estimate	SE		
n10 l800 vs. n04 l050	4795	11.278	0.0341	2.040	0.0318	53.65	0.27
n10 l800 vs. n04 l100	15648	10.460	0.0188	2.208	0.0194	97.30	0.40
n10 l800 vs. n04 l200	31362	10.516	0.0124	2.138	0.0134	132.00	0.44
n10 l800 vs. n04 l400	49230	11.302	0.0099	2.095	0.0104	163.70	0.37
n10 l800 vs. n04 l600	56085	12.153	0.0097	2.102	0.0097	182.03	0.40
n10 l800 vs. n04 l800	57567	12.568	0.0096	2.101	0.0095	177.88	0.39
n10 l800 vs. n06 l050	6504	11.378	0.0282	1.961	0.0261	52.64	0.41
n10 l800 vs. n06 l100	17907	10.689	0.0187	2.251	0.0182	93.23	0.41
n10 l800 vs. n06 l200	32361	10.661	0.0134	2.255	0.0137	130.46	0.41
n10 l800 vs. n06 l400	50870	11.511	0.0109	2.256	0.0109	171.33	0.42
n10 l800 vs. n06 l600	56609	12.320	0.0106	2.308	0.0106	183.86	0.42
n10 l800 vs. n06 l800	57899	12.921	0.0103	2.159	0.0096	181.87	0.40
n10 l800 vs. n08 l050	6496	11.406	0.0298	2.111	0.0283	48.25	0.59
n10 l800 vs. n08 l100	16619	10.695	0.0197	2.251	0.0188	95.58	0.42
n10 l800 vs. n08 l200	31969	10.910	0.0145	2.376	0.0144	135.63	0.35
n10 l800 vs. n08 l400	47984	11.835	0.0119	2.380	0.0118	168.73	0.42
n10 l800 vs. n08 l600	54586	12.492	0.0110	2.304	0.0107	179.87	0.38
n10 l800 vs. n08 l800	56718	13.085	0.0109	2.278	0.0103	184.18	0.36
n12 l050 vs. n02 l050	2313	6.679	0.0297	1.201	0.0269	69.66	0.16
n12 l050 vs. n02 l100	3400	7.443	0.0260	1.312	0.0244	74.07	0.22
n12 l050 vs. n02 l200	3208	8.634	0.0304	1.522	0.0294	33.12	0.52
n12 l050 vs. n02 l400	2669	9.848	0.0329	1.518	0.0319	25.45	0.76
n12 l050 vs. n02 l600	2259	10.648	0.0350	1.438	0.0326	31.03	0.44
n12 l050 vs. n02 l800	2223	11.224	0.0372	1.463	0.0332	26.89	0.62
n12 l050 vs. n04 l050	1951	6.868	0.0375	1.532	0.0384	58.34	0.19
n12 l050 vs. n04 l100	2903	7.601	0.0332	1.690	0.0350	44.17	0.30
n12 l050 vs. n04 l200	3035	8.540	0.0355	1.850	0.0371	33.23	0.44
n12 l050 vs. n04 l400	3330	9.611	0.0374	1.888	0.0353	35.66	0.45
n12 l050 vs. n04 l600	3522	10.438	0.0367	1.932	0.0353	43.46	0.40
n12 l050 vs. n04 l800	3362	11.036	0.0377	1.953	0.0366	41.16	0.31
n12 l050 vs. n06 l050	1601	7.042	0.0484	1.856	0.0517	21.90	0.62
n12 l050 vs. n06 l100	2388	7.975	0.0417	1.819	0.0407	30.51	0.53
n12 l050 vs. n06 l200	2407	9.133	0.0410	1.791	0.0396	30.62	0.43
n12 l050 vs. n06 l400	3012	10.436	0.0392	1.832	0.0362	38.45	0.37
n12 l050 vs. n06 l600	3223	11.163	0.0372	1.878	0.0361	35.62	0.53
n12 l050 vs. n06 l800	3446	11.445	0.0350	1.756	0.0321	39.15	0.51
n12 l050 vs. n08 l050	1653	6.933	0.0481	1.866	0.0510	32.79	0.42
n12 l050 vs. n08 l100	2393	7.756	0.0425	1.931	0.0435	28.37	0.59
n12 l050 vs. n08 l200	3007	8.860	0.0401	1.962	0.0389	38.17	0.38
n12 l050 vs. n08 l400	3865	9.907	0.0364	1.995	0.0348	43.27	0.45
n12 l050 vs. n08 l600	4166	10.586	0.0340	1.958	0.0330	48.56	0.40
n12 l050 vs. n08 l800	4277	11.095	0.0347	1.960	0.0324	48.95	0.37
n12 l050 vs. n10 l050	2183	7.082	0.0435	1.984	0.0473	18.80	0.96

continues ...

(continued)

Distribution	N	Location		Scale		AD_{up}	p-value
		Estimate	SE	Estimate	SE		
n12 l050 vs. n10 l100	3432	7.519	0.0384	2.116	0.0398	42.23	0.33
n12 l050 vs. n10 l200	3960	8.682	0.0371	2.073	0.0359	43.51	0.34
n12 l050 vs. n10 l400	4227	9.956	0.0352	2.037	0.0340	46.58	0.38
n12 l050 vs. n10 l600	4745	10.794	0.0344	2.058	0.0323	47.66	0.51
n12 l050 vs. n10 l800	4932	11.360	0.0333	1.975	0.0302	52.60	0.37
n12 l100 vs. n02 l050	3583	7.359	0.0259	1.311	0.0237	61.25	0.29
n12 l100 vs. n02 l100	10639	7.316	0.0151	1.358	0.0144	112.68	0.24
n12 l100 vs. n02 l200	13580	8.105	0.0144	1.530	0.0145	70.58	0.51
n12 l100 vs. n02 l400	11993	9.180	0.0163	1.592	0.0159	70.98	0.40
n12 l100 vs. n02 l600	10635	9.920	0.0180	1.683	0.0178	73.39	0.43
n12 l100 vs. n02 l800	9561	10.450	0.0199	1.705	0.0189	74.09	0.38
n12 l100 vs. n04 l050	3260	7.555	0.0322	1.755	0.0342	38.65	0.40
n12 l100 vs. n04 l100	8745	7.335	0.0193	1.710	0.0203	127.24	0.20
n12 l100 vs. n04 l200	12075	7.973	0.0166	1.750	0.0177	111.99	0.28
n12 l100 vs. n04 l400	12999	9.012	0.0179	1.930	0.0187	80.35	0.38
n12 l100 vs. n04 l600	13195	9.809	0.0188	2.007	0.0191	85.33	0.40
n12 l100 vs. n04 l800	12015	10.326	0.0206	2.050	0.0204	76.60	0.41
n12 l100 vs. n06 l050	3075	7.703	0.0361	1.865	0.0370	38.89	0.32
n12 l100 vs. n06 l100	6825	7.505	0.0254	1.972	0.0263	59.54	0.42
n12 l100 vs. n06 l200	8753	8.288	0.0224	1.971	0.0232	68.71	0.36
n12 l100 vs. n06 l400	10144	9.650	0.0225	2.020	0.0217	71.68	0.37
n12 l100 vs. n06 l600	10574	10.271	0.0234	2.129	0.0224	79.60	0.51
n12 l100 vs. n06 l800	11420	10.698	0.0220	2.014	0.0202	81.80	0.38
n12 l100 vs. n08 l050	3132	7.591	0.0377	1.994	0.0395	29.37	0.70
n12 l100 vs. n08 l100	7390	7.368	0.0248	2.061	0.0267	53.60	0.50
n12 l100 vs. n08 l200	10349	8.154	0.0218	2.102	0.0228	73.44	0.48
n12 l100 vs. n08 l400	12835	9.185	0.0210	2.146	0.0206	84.90	0.32
n12 l100 vs. n08 l600	13431	9.974	0.0204	2.100	0.0196	87.67	0.36
n12 l100 vs. n08 l800	13629	10.594	0.0203	2.083	0.0193	89.00	0.35
n12 l100 vs. n10 l050	4211	7.833	0.0345	2.037	0.0343	42.47	0.44
n12 l100 vs. n10 l100	9502	7.314	0.0228	2.120	0.0241	70.36	0.45
n12 l100 vs. n10 l200	13127	7.995	0.0200	2.219	0.0215	80.48	0.42
n12 l100 vs. n10 l400	14152	9.258	0.0204	2.222	0.0204	91.61	0.39
n12 l100 vs. n10 l600	14959	10.175	0.0204	2.198	0.0194	88.98	0.37
n12 l100 vs. n10 l800	15189	10.728	0.0207	2.201	0.0191	96.43	0.45
n12 l200 vs. n02 l050	3972	8.276	0.0264	1.465	0.0254	40.39	0.48
n12 l200 vs. n02 l100	16115	7.906	0.0128	1.462	0.0127	77.58	0.46
n12 l200 vs. n02 l200	29369	8.333	0.0096	1.535	0.0100	99.90	0.59
n12 l200 vs. n02 l400	30216	9.320	0.0099	1.582	0.0100	120.67	0.44
n12 l200 vs. n02 l600	27408	10.081	0.0109	1.675	0.0111	109.16	0.42
n12 l200 vs. n02 l800	25382	10.524	0.0118	1.723	0.0119	105.39	0.52
n12 l200 vs. n04 l050	4260	8.650	0.0297	1.802	0.0305	42.37	0.42
n12 l200 vs. n04 l100	14212	8.004	0.0156	1.809	0.0170	61.87	0.68

continues ...

(continued)

Distribution	N	Location		Scale		AD_{up}	p-value
		Estimate	SE	Estimate	SE		
n12 l200 vs. n04 l200	25791	8.221	0.0115	1.819	0.0127	96.91	0.52
n12 l200 vs. n04 l400	29344	9.342	0.0119	1.975	0.0128	91.94	0.63
n12 l200 vs. n04 l600	30078	10.176	0.0121	1.995	0.0127	125.62	0.42
n12 l200 vs. n04 l800	28226	10.616	0.0129	2.032	0.0133	122.60	0.42
n12 l200 vs. n06 l050	4308	8.784	0.0325	1.906	0.0316	46.22	0.44
n12 l200 vs. n06 l100	12812	8.186	0.0194	2.036	0.0197	82.68	0.37
n12 l200 vs. n06 l200	21464	8.180	0.0144	2.044	0.0155	103.83	0.31
n12 l200 vs. n06 l400	25108	9.523	0.0143	2.074	0.0143	115.24	0.39
n12 l200 vs. n06 l600	25592	10.278	0.0148	2.157	0.0147	108.06	0.36
n12 l200 vs. n06 l800	27435	10.796	0.0142	2.090	0.0136	124.21	0.34
n12 l200 vs. n08 l050	4409	8.774	0.0328	1.984	0.0328	42.18	0.38
n12 l200 vs. n08 l100	12555	8.056	0.0198	2.130	0.0210	79.75	0.31
n12 l200 vs. n08 l200	21916	8.275	0.0151	2.174	0.0163	110.42	0.30
n12 l200 vs. n08 l400	27462	9.412	0.0141	2.153	0.0142	121.97	0.43
n12 l200 vs. n08 l600	29301	10.181	0.0135	2.095	0.0133	130.38	0.42
n12 l200 vs. n08 l800	29721	10.770	0.0136	2.090	0.0131	126.30	0.38
n12 l200 vs. n10 l050	6098	8.991	0.0288	2.014	0.0280	56.19	0.34
n12 l200 vs. n10 l100	14840	8.268	0.0190	2.162	0.0195	89.33	0.33
n12 l200 vs. n10 l200	24453	8.401	0.0151	2.271	0.0161	115.41	0.39
n12 l200 vs. n10 l400	29774	9.468	0.0140	2.269	0.0144	121.27	0.34
n12 l200 vs. n10 l600	31482	10.444	0.0140	2.239	0.0137	136.24	0.26
n12 l200 vs. n10 l800	31332	11.041	0.0142	2.211	0.0135	129.82	0.45
n12 l400 vs. n02 l050	3226	8.919	0.0315	1.548	0.0294	27.43	0.84
n12 l400 vs. n02 l100	13156	8.662	0.0152	1.617	0.0156	75.09	0.32
n12 l400 vs. n02 l200	29151	8.994	0.0102	1.642	0.0107	98.22	0.56
n12 l400 vs. n02 l400	41046	9.701	0.0085	1.609	0.0088	126.58	0.46
n12 l400 vs. n02 l600	40272	10.323	0.0089	1.639	0.0090	148.51	0.40
n12 l400 vs. n02 l800	37876	10.818	0.0094	1.675	0.0094	135.90	0.38
n12 l400 vs. n04 l050	3898	9.472	0.0349	1.996	0.0351	45.69	0.38
n12 l400 vs. n04 l100	12542	8.840	0.0179	1.923	0.0190	79.18	0.36
n12 l400 vs. n04 l200	25757	8.916	0.0119	1.876	0.0131	102.06	0.44
n12 l400 vs. n04 l400	36844	9.767	0.0106	1.981	0.0115	141.12	0.39
n12 l400 vs. n04 l600	39447	10.549	0.0108	2.013	0.0111	141.07	0.33
n12 l400 vs. n04 l800	39095	10.990	0.0111	2.054	0.0114	140.98	0.46
n12 l400 vs. n06 l050	4343	9.514	0.0336	1.940	0.0319	44.21	0.47
n12 l400 vs. n06 l100	13367	8.902	0.0195	2.029	0.0190	69.54	0.51
n12 l400 vs. n06 l200	23592	8.770	0.0140	2.048	0.0147	103.39	0.44
n12 l400 vs. n06 l400	34581	9.708	0.0123	2.117	0.0124	139.20	0.34
n12 l400 vs. n06 l600	35592	10.505	0.0125	2.167	0.0125	134.51	0.46
n12 l400 vs. n06 l800	37234	11.079	0.0122	2.081	0.0117	151.14	0.38
n12 l400 vs. n08 l050	4735	9.454	0.0336	2.071	0.0327	42.71	0.45
n12 l400 vs. n08 l100	12358	8.919	0.0211	2.146	0.0210	82.62	0.35
n12 l400 vs. n08 l200	23643	8.998	0.0150	2.175	0.0155	110.03	0.39

continues . . .

(continued)

Distribution	N	Location Estimate	Location SE	Scale Estimate	Scale SE	AD_{up}	p-value
n12 l400 vs. n08 l400	33622	9.907	0.0132	2.215	0.0132	136.14	0.36
n12 l400 vs. n08 l600	37572	10.562	0.0123	2.152	0.0121	144.45	0.36
n12 l400 vs. n08 l800	39093	11.061	0.0123	2.185	0.0120	117.59	0.58
n12 l400 vs. n10 l050	6544	9.842	0.0295	2.020	0.0268	59.57	0.40
n12 l400 vs. n10 l100	14756	9.236	0.0196	2.165	0.0194	85.83	0.44
n12 l400 vs. n10 l200	25259	9.328	0.0151	2.242	0.0155	111.70	0.39
n12 l400 vs. n10 l400	36296	9.978	0.0130	2.298	0.0132	145.49	0.33
n12 l400 vs. n10 l600	40982	10.777	0.0126	2.287	0.0122	151.73	0.34
n12 l400 vs. n10 l800	41605	11.338	0.0125	2.274	0.0121	154.29	0.27
n14 l050 vs. n02 l100	1683	7.468	0.0367	1.266	0.0332	22.45	0.62
n14 l050 vs. n04 l400	1734	9.671	0.0451	1.591	0.0410	27.87	0.44
n14 l050 vs. n04 l600	1807	10.342	0.0442	1.542	0.0388	31.60	0.46
n14 l050 vs. n04 l800	1555	10.860	0.0465	1.496	0.0404	27.35	0.39
n14 l050 vs. n06 l400	1653	10.292	0.0415	1.504	0.0404	28.06	0.35
n14 l050 vs. n06 l600	1659	10.938	0.0441	1.585	0.0423	28.96	0.33
n14 l050 vs. n06 l800	1872	11.292	0.0433	1.572	0.0391	32.84	0.44
n14 l050 vs. n08 l200	1728	9.021	0.0463	1.657	0.0429	24.41	0.58
n14 l050 vs. n08 l400	2312	9.745	0.0408	1.606	0.0358	23.81	0.74
n14 l050 vs. n08 l600	2348	10.332	0.0387	1.582	0.0351	34.23	0.40
n14 l050 vs. n08 l800	2363	10.799	0.0402	1.662	0.0369	35.55	0.47
n14 l050 vs. n10 l100	2021	7.645	0.0437	1.740	0.0420	28.50	0.48
n14 l050 vs. n10 l200	2378	8.649	0.0417	1.710	0.0376	31.49	0.47
n14 l050 vs. n10 l400	2456	9.844	0.0396	1.701	0.0370	28.45	0.61
n14 l050 vs. n10 l600	2699	10.488	0.0371	1.592	0.0327	38.62	0.39
n14 l050 vs. n10 l800	2658	10.896	0.0377	1.657	0.0348	39.25	0.40
n14 l050 vs. n12 l100	1596	7.518	0.0474	1.644	0.0443	23.67	0.48
n14 l050 vs. n12 l200	2483	8.721	0.0401	1.623	0.0347	35.96	0.37
n14 l050 vs. n12 l400	2440	9.450	0.0395	1.588	0.0343	36.41	0.36
n14 l100 vs. n02 l050	1958	7.187	0.0357	1.339	0.0325	26.20	0.50
n14 l100 vs. n02 l100	6141	7.153	0.0200	1.418	0.0199	46.27	0.56
n14 l100 vs. n02 l200	7585	8.048	0.0197	1.591	0.0201	50.02	0.52
n14 l100 vs. n02 l400	6291	9.271	0.0222	1.571	0.0215	45.87	0.62
n14 l100 vs. n02 l600	5454	10.135	0.0255	1.617	0.0236	48.38	0.41
n14 l100 vs. n02 l800	4816	10.643	0.0275	1.650	0.0255	43.29	0.49
n14 l100 vs. n04 l050	1916	7.564	0.0435	1.704	0.0425	15.34	0.98
n14 l100 vs. n04 l100	4924	7.237	0.0270	1.797	0.0284	43.26	0.49
n14 l100 vs. n04 l200	6411	8.042	0.0235	1.759	0.0242	43.48	0.69
n14 l100 vs. n04 l400	6909	9.214	0.0248	1.848	0.0241	53.00	0.39
n14 l100 vs. n04 l600	7457	9.964	0.0251	1.890	0.0236	53.72	0.47
n14 l100 vs. n04 l800	6628	10.453	0.0269	1.879	0.0248	76.15	0.30
n14 l100 vs. n06 l050	1787	7.744	0.0483	1.838	0.0474	19.00	0.84
n14 l100 vs. n06 l100	4136	7.628	0.0335	1.914	0.0323	421.86	0.02
n14 l100 vs. n06 l200	5003	8.500	0.0304	1.891	0.0289	119.95	0.18

continues . . .

(continued)

Distribution	N	Location		Scale		AD_{up}	p-value
		Estimate	SE	Estimate	SE		
n14 l100 vs. n06 l400	5879	9.845	0.0286	1.842	0.0257	51.04	0.50
n14 l100 vs. n06 l600	6311	10.479	0.0289	1.965	0.0266	42.72	0.70
n14 l100 vs. n06 l800	6871	10.775	0.0270	1.877	0.0243	43.95	0.70
n14 l100 vs. n08 l050	1827	7.711	0.0503	1.936	0.0495	25.16	0.46
n14 l100 vs. n08 l100	4222	7.511	0.0338	1.954	0.0325	62.39	0.34
n14 l100 vs. n08 l200	5862	8.299	0.0296	2.018	0.0286	41.22	0.67
n14 l100 vs. n08 l400	7597	9.407	0.0257	1.924	0.0238	49.38	0.59
n14 l100 vs. n08 l600	8160	10.076	0.0247	1.871	0.0222	58.77	0.47
n14 l100 vs. n08 l800	8250	10.621	0.0245	1.913	0.0227	69.34	0.39
n14 l100 vs. n10 l050	2594	7.905	0.0431	1.844	0.0387	35.97	0.42
n14 l100 vs. n10 l100	5490	7.436	0.0306	2.082	0.0307	43.83	0.47
n14 l100 vs. n10 l200	7548	8.149	0.0265	2.059	0.0257	92.76	0.18
n14 l100 vs. n10 l400	8504	9.426	0.0254	2.052	0.0240	49.68	0.65
n14 l100 vs. n10 l600	9093	10.259	0.0246	1.988	0.0223	47.16	0.85
n14 l100 vs. n10 l800	9347	10.782	0.0243	1.975	0.0219	62.01	0.44
n14 l100 vs. n12 l050	1803	7.543	0.0496	1.888	0.0482	20.46	0.71
n14 l100 vs. n12 l100	5041	7.204	0.0305	2.025	0.0314	49.13	0.40
n14 l100 vs. n12 l200	7867	8.184	0.0249	1.924	0.0234	67.36	0.41
n14 l100 vs. n12 l400	7781	9.220	0.0252	1.888	0.0230	57.41	0.46
n14 l200 vs. n02 l050	2698	7.902	0.0344	1.514	0.0313	33.28	0.52
n14 l200 vs. n02 l100	10924	7.661	0.0153	1.499	0.0159	66.36	0.47
n14 l200 vs. n02 l200	19704	8.134	0.0117	1.568	0.0125	82.19	0.57
n14 l200 vs. n02 l400	18627	9.249	0.0126	1.616	0.0131	85.31	0.46
n14 l200 vs. n02 l600	16213	10.088	0.0148	1.718	0.0147	85.69	0.43
n14 l200 vs. n02 l800	14739	10.586	0.0159	1.758	0.0158	86.98	0.38
n14 l200 vs. n04 l050	2864	8.404	0.0398	1.929	0.0393	38.99	0.38
n14 l200 vs. n04 l100	9212	7.792	0.0205	1.940	0.0226	55.21	0.54
n14 l200 vs. n04 l200	15853	8.010	0.0150	1.906	0.0171	70.27	0.61
n14 l200 vs. n04 l400	17262	9.274	0.0158	2.002	0.0169	76.81	0.55
n14 l200 vs. n04 l600	18409	10.147	0.0164	2.075	0.0168	95.01	0.30
n14 l200 vs. n04 l800	17237	10.614	0.0170	2.084	0.0174	93.09	0.43
n14 l200 vs. n06 l050	2909	8.573	0.0420	1.976	0.0396	78.15	0.18
n14 l200 vs. n06 l100	8186	7.906	0.0245	2.057	0.0249	235.03	0.09
n14 l200 vs. n06 l200	12768	8.077	0.0194	2.080	0.0203	59.21	0.71
n14 l200 vs. n06 l400	15648	9.533	0.0182	2.042	0.0177	81.04	0.35
n14 l200 vs. n06 l600	16040	10.329	0.0191	2.137	0.0182	83.42	0.49
n14 l200 vs. n06 l800	17277	10.736	0.0179	2.036	0.0166	89.41	0.39
n14 l200 vs. n08 l050	2943	8.543	0.0423	2.001	0.0398	203.80	0.04
n14 l200 vs. n08 l100	7758	7.872	0.0258	2.076	0.0257	50.48	0.57
n14 l200 vs. n08 l200	13255	8.208	0.0202	2.204	0.0211	78.45	0.33
n14 l200 vs. n08 l400	17349	9.322	0.0179	2.172	0.0180	85.39	0.47
n14 l200 vs. n08 l600	18613	10.085	0.0172	2.118	0.0169	97.58	0.39
n14 l200 vs. n08 l800	19230	10.681	0.0170	2.101	0.0164	90.66	0.46

continues . . .

(continued)

Distribution	N	Location		Scale		AD_{up}	p-value
		Estimate	SE	Estimate	SE		
n14 l200 vs. n10 l050	4290	8.723	0.0367	2.076	0.0342	47.46	0.31
n14 l200 vs. n10 l100	9957	8.129	0.0242	2.217	0.0243	58.92	0.41
n14 l200 vs. n10 l200	15737	8.296	0.0190	2.273	0.0200	83.16	0.45
n14 l200 vs. n10 l400	19441	9.376	0.0178	2.282	0.0179	100.60	0.39
n14 l200 vs. n10 l600	21261	10.347	0.0170	2.201	0.0163	107.63	0.40
n14 l200 vs. n10 l800	21669	10.950	0.0172	2.201	0.0161	100.53	0.42
n14 l200 vs. n12 l050	2887	8.534	0.0441	2.069	0.0417	36.21	0.39
n14 l200 vs. n12 l100	8870	7.857	0.0247	2.169	0.0253	68.93	0.43
n14 l200 vs. n12 l200	16263	8.313	0.0182	2.171	0.0187	79.65	0.55
n14 l200 vs. n12 l400	17233	9.298	0.0181	2.164	0.0180	76.58	0.63
n14 l400 vs. n02 l050	2644	9.085	0.0366	1.674	0.0355	35.09	0.36
n14 l400 vs. n02 l100	10880	8.769	0.0172	1.665	0.0176	50.59	0.72
n14 l400 vs. n02 l200	25779	9.007	0.0109	1.684	0.0117	107.84	0.45
n14 l400 vs. n02 l400	36065	9.774	0.0091	1.655	0.0097	130.36	0.39
n14 l400 vs. n02 l600	34451	10.480	0.0097	1.725	0.0103	140.22	0.43
n14 l400 vs. n02 l800	31438	11.034	0.0105	1.745	0.0109	124.12	0.47
n14 l400 vs. n04 l050	3089	9.683	0.0420	2.038	0.0396	40.51	0.42
n14 l400 vs. n04 l100	9954	8.992	0.0216	2.103	0.0234	76.62	0.33
n14 l400 vs. n04 l200	21041	8.856	0.0138	2.048	0.0160	99.45	0.41
n14 l400 vs. n04 l400	30342	9.732	0.0121	2.106	0.0135	128.60	0.34
n14 l400 vs. n04 l600	32327	10.625	0.0125	2.171	0.0134	127.46	0.31
n14 l400 vs. n04 l800	31827	11.106	0.0127	2.140	0.0132	125.74	0.40
n14 l400 vs. n06 l050	3341	9.725	0.0389	1.943	0.0362	42.64	0.42
n14 l400 vs. n06 l100	9857	9.081	0.0237	2.130	0.0233	57.37	0.59
n14 l400 vs. n06 l200	18535	8.721	0.0166	2.161	0.0175	93.06	0.41
n14 l400 vs. n06 l400	27704	9.615	0.0139	2.185	0.0144	116.43	0.45
n14 l400 vs. n06 l600	28810	10.547	0.0142	2.246	0.0145	125.96	0.35
n14 l400 vs. n06 l800	30309	11.136	0.0136	2.114	0.0131	131.83	0.41
n14 l400 vs. n08 l050	3463	9.696	0.0392	2.053	0.0379	33.72	0.63
n14 l400 vs. n08 l100	9491	9.041	0.0244	2.105	0.0233	71.35	0.37
n14 l400 vs. n08 l200	18272	9.009	0.0180	2.307	0.0188	102.74	0.39
n14 l400 vs. n08 l400	27109	9.857	0.0149	2.282	0.0152	120.63	0.42
n14 l400 vs. n08 l600	30631	10.498	0.0136	2.187	0.0136	133.33	0.43
n14 l400 vs. n08 l800	32067	11.061	0.0136	2.211	0.0134	129.98	0.44
n14 l400 vs. n10 l050	5270	10.029	0.0326	2.089	0.0313	54.53	0.37
n14 l400 vs. n10 l100	11821	9.414	0.0225	2.186	0.0218	76.85	0.37
n14 l400 vs. n10 l200	20648	9.360	0.0177	2.402	0.0184	109.08	0.40
n14 l400 vs. n10 l400	29698	9.959	0.0148	2.391	0.0152	125.87	0.38
n14 l400 vs. n10 l600	34497	10.763	0.0137	2.322	0.0136	136.12	0.34
n14 l400 vs. n10 l800	35763	11.392	0.0136	2.317	0.0133	148.52	0.27
n14 l400 vs. n12 l050	3274	9.867	0.0409	1.974	0.0370	41.57	0.27
n14 l400 vs. n12 l100	10020	9.182	0.0241	2.161	0.0235	73.78	0.47
n14 l400 vs. n12 l200	21426	9.427	0.0166	2.253	0.0169	106.54	0.41

continues . . .

(continued)

Distribution	N	Location		Scale		AD_{up}	p-value
		Estimate	SE	Estimate	SE		
n14 l400 vs. n12 l400	26534	9.935	0.0151	2.293	0.0154	112.44	0.39

Distribution represents distributions obtained from aligning simulated profiles of ENO n and length l against simulated profiles with different values of n and l. The table reports the estimates and their standard errors (SE) for the location and scale parameters of the EVD for each distribution of alignment scores. N is the number of alignment scores. AD_{up} is the upper-tail Anderson-Darling statistic. The p-value of statistic AD_{up} was computed by Monte Carlo simulation with 100 samples.
