# Supplementary material for: Estimating statistical significance of local protein profile-profile alignments
Source: BMC Bioinformatics. 2019 Aug 13;20:419. doi: 10.1186/s12859-019-2913-3 (PMC6693267; doi:10.1186/s12859-019-2913-3)
Supplement: Supplementary file 11 — Table S4. Goodness of fit of the EVD to the distribution of alignment scores of profiles generated using S=1012 seed profiles with s=9 and r=0.03. (PDF 44 kb) [file 12859_2019_2913_MOESM11_ESM.pdf]

Table S4. Goodness of fit of the EVD to the distribution of alignment scores of profiles generated using $S = 1012$ seed profiles with $s = 9$ and $r = 0.03$

Distribution	N	Location		Scale		AD_{up}	p-value
		Estimate	SE	Estimate	SE		
λ01.0: l400 vs. l200	2019	10.333	0.0554	2.258	0.0546	29.34	0.40
λ01.0: l400 vs. l400	1619	10.505	0.0594	2.127	0.0574	27.00	0.35
λ01.1: l050 vs. l050	5958	7.927	0.0225	1.417	0.0197	333.11	0.08
λ01.1: l100 vs. l050	15583	8.764	0.0141	1.432	0.0123	81.26	0.43
λ01.1: l100 vs. l100	20432	8.980	0.0129	1.471	0.0110	93.88	0.43
λ01.1: l200 vs. l050	15440	9.745	0.0146	1.415	0.0121	86.76	0.35
λ01.1: l200 vs. l100	59947	9.744	0.0078	1.531	0.0067	169.23	0.42
λ01.1: l200 vs. l200	56449	10.204	0.0083	1.609	0.0073	150.74	0.49
λ01.1: l400 vs. l050	14077	10.767	0.0154	1.453	0.0130	74.79	0.57
λ01.1: l400 vs. l100	67195	10.736	0.0076	1.589	0.0066	171.52	0.42
λ01.1: l400 vs. l200	137355	11.145	0.0057	1.713	0.0049	228.95	0.58
λ01.1: l400 vs. l400	95305	11.788	0.0077	1.907	0.0065	218.29	0.39
λ01.1: l600 vs. l050	12215	11.478	0.0165	1.459	0.0140	69.83	0.59
λ01.1: l600 vs. l100	63144	11.505	0.0075	1.603	0.0069	162.35	0.44
λ01.1: l600 vs. l200	127300	12.077	0.0057	1.698	0.0051	257.81	0.33
λ01.1: l600 vs. l400	178145	12.754	0.0055	1.891	0.0048	285.90	0.41
λ01.1: l600 vs. l600	86397	13.798	0.0074	1.902	0.0070	162.04	0.60
λ01.1: l800 vs. l050	11665	11.956	0.0170	1.459	0.0144	73.60	0.40
λ01.1: l800 vs. l100	60683	11.940	0.0078	1.635	0.0072	173.62	0.30
λ01.1: l800 vs. l200	125502	12.511	0.0058	1.736	0.0053	196.16	0.75
λ01.1: l800 vs. l400	180114	13.240	0.0055	1.929	0.0049	319.71	0.33
λ01.1: l800 vs. l600	177805	14.258	0.0053	1.952	0.0050	312.19	0.37
λ01.1: l800 vs. l800	92685	14.694	0.0076	2.018	0.0072	219.46	0.43
λ01.2: l050 vs. l050	12076	7.681	0.0156	1.418	0.0139	467.32	0.07
λ01.2: l100 vs. l050	33668	8.424	0.0097	1.474	0.0086	135.49	0.40
λ01.2: l100 vs. l100	39512	8.320	0.0093	1.563	0.0085	586.12	0.11
λ01.2: l200 vs. l050	33228	9.396	0.0102	1.527	0.0090	117.20	0.55
λ01.2: l200 vs. l100	108196	8.878	0.0057	1.614	0.0053	224.58	0.39
λ01.2: l200 vs. l200	105325	9.040	0.0057	1.634	0.0055	211.74	0.53
λ01.2: l400 vs. l050	29566	10.387	0.0110	1.560	0.0097	100.45	0.59
λ01.2: l400 vs. l100	106716	9.813	0.0059	1.672	0.0056	219.76	0.46
λ01.2: l400 vs. l200	249372	9.861	0.0038	1.680	0.0037	329.89	0.36
λ01.2: l400 vs. l400	185836	10.467	0.0045	1.700	0.0043	262.43	0.59
λ01.2: l600 vs. l050	23886	11.118	0.0120	1.559	0.0108	100.49	0.53
λ01.2: l600 vs. l100	86549	10.541	0.0065	1.690	0.0062	172.76	0.54
λ01.2: l600 vs. l200	201135	10.624	0.0041	1.676	0.0041	331.60	0.42
λ01.2: l600 vs. l400	312854	11.182	0.0034	1.673	0.0033	366.52	0.46
λ01.2: l600 vs. l600	138781	11.852	0.0049	1.665	0.0049	165.10	0.84
λ01.2: l800 vs. l050	21722	11.589	0.0127	1.569	0.0114	98.26	0.44
λ01.2: l800 vs. l100	81870	10.963	0.0068	1.708	0.0065	190.16	0.51
λ01.2: l800 vs. l200	193705	11.058	0.0043	1.714	0.0043	287.66	0.42

continues . . .

(continued)

Distribution	N	Location		Scale		AD_{up}	p-value
		Estimate	SE	Estimate	SE		
λ01.2: l800 vs. l400	310309	11.614	0.0034	1.701	0.0033	388.25	0.33
λ01.2: l800 vs. l600	280856	12.248	0.0035	1.676	0.0035	275.84	0.77
λ01.2: l800 vs. l800	144517	12.637	0.0050	1.692	0.0049	263.31	0.42
λ01.3: l050 vs. l050	11938	7.338	0.0152	1.415	0.0140	1374.97	0.05
λ01.3: l100 vs. l050	35867	8.057	0.0097	1.599	0.0092	129.12	0.44
λ01.3: l100 vs. l100	44375	7.883	0.0088	1.672	0.0087	160.35	0.34
λ01.3: l200 vs. l050	38958	9.042	0.0099	1.718	0.0095	97.95	0.75
λ01.3: l200 vs. l100	127224	8.473	0.0054	1.773	0.0055	223.04	0.54
λ01.3: l200 vs. l200	118503	8.651	0.0055	1.797	0.0058	146.95	0.88
λ01.3: l400 vs. l050	38837	10.102	0.0104	1.791	0.0099	144.61	0.37
λ01.3: l400 vs. l100	132484	9.479	0.0056	1.891	0.0057	258.20	0.34
λ01.3: l400 vs. l200	273073	9.582	0.0038	1.916	0.0041	355.93	0.47
λ01.3: l400 vs. l400	188500	10.293	0.0048	1.987	0.0051	303.34	0.43
λ01.3: l600 vs. l050	31000	10.876	0.0116	1.804	0.0111	132.67	0.43
λ01.3: l600 vs. l100	106721	10.268	0.0065	1.957	0.0066	234.60	0.38
λ01.3: l600 vs. l200	219665	10.435	0.0044	1.992	0.0047	337.59	0.39
λ01.3: l600 vs. l400	321068	11.169	0.0038	2.049	0.0040	409.65	0.40
λ01.3: l600 vs. l600	149802	12.000	0.0056	2.067	0.0059	287.20	0.35
λ01.3: l800 vs. l050	29304	11.389	0.0122	1.822	0.0115	133.93	0.45
λ01.3: l800 vs. l100	101864	10.748	0.0068	1.997	0.0069	183.36	0.60
λ01.3: l800 vs. l200	210566	10.892	0.0046	2.034	0.0049	333.45	0.45
λ01.3: l800 vs. l400	319138	11.633	0.0039	2.064	0.0040	405.27	0.47
λ01.3: l800 vs. l600	313706	12.470	0.0040	2.071	0.0041	408.65	0.50
λ01.3: l800 vs. l800	166949	12.946	0.0055	2.078	0.0056	308.92	0.35
λ01.4: l050 vs. l050	9178	7.055	0.0173	1.439	0.0163	72.36	0.40
λ01.4: l100 vs. l050	29942	7.836	0.0109	1.671	0.0106	707.46	0.03
λ01.4: l100 vs. l100	38554	7.665	0.0099	1.779	0.0100	285.60	0.21
λ01.4: l200 vs. l050	36547	8.842	0.0105	1.785	0.0102	94.65	0.82
λ01.4: l200 vs. l100	114993	8.308	0.0059	1.884	0.0061	188.86	0.61
λ01.4: l200 vs. l200	108701	8.470	0.0059	1.905	0.0065	167.23	0.70
λ01.4: l400 vs. l050	40361	9.994	0.0107	1.914	0.0104	140.32	0.41
λ01.4: l400 vs. l100	126174	9.353	0.0061	2.016	0.0063	254.99	0.31
λ01.4: l400 vs. l200	254341	9.435	0.0041	2.041	0.0045	364.51	0.55
λ01.4: l400 vs. l400	169077	10.215	0.0053	2.127	0.0057	309.47	0.35
λ01.4: l600 vs. l050	32718	10.795	0.0120	1.930	0.0116	131.55	0.30
λ01.4: l600 vs. l100	101320	10.131	0.0070	2.068	0.0071	241.25	0.37
λ01.4: l600 vs. l200	210221	10.245	0.0047	2.112	0.0051	345.75	0.43
λ01.4: l600 vs. l400	296542	10.984	0.0041	2.163	0.0044	396.62	0.42
λ01.4: l600 vs. l600	131994	11.701	0.0063	2.191	0.0067	273.96	0.40
λ01.4: l800 vs. l050	32671	11.307	0.0122	1.926	0.0115	137.04	0.37
λ01.4: l800 vs. l100	97149	10.648	0.0073	2.096	0.0073	239.38	0.27
λ01.4: l800 vs. l200	201394	10.758	0.0050	2.154	0.0053	333.43	0.35
λ01.4: l800 vs. l400	289293	11.500	0.0043	2.189	0.0045	402.53	0.39

continues . . .

(continued)

Distribution	N	Location		Scale		AD_{up}	p-value
		Estimate	SE	Estimate	SE		
λ01.4: l800 vs. l600	260461	12.229	0.0046	2.194	0.0047	367.04	0.42
λ01.4: l800 vs. l800	129107	12.739	0.0066	2.166	0.0065	275.02	0.38
λ01.5: l050 vs. l050	6239	6.963	0.0204	1.450	0.0202	80.97	0.28
λ01.5: l100 vs. l050	19787	7.625	0.0131	1.646	0.0128	266.51	0.14
λ01.5: l100 vs. l100	25868	7.422	0.0119	1.784	0.0123	223.03	0.22
λ01.5: l200 vs. l050	25144	8.691	0.0130	1.832	0.0126	251.42	0.19
λ01.5: l200 vs. l100	76647	8.071	0.0073	1.913	0.0077	159.60	0.58
λ01.5: l200 vs. l200	70451	8.248	0.0077	1.986	0.0084	177.08	0.43
λ01.5: l400 vs. l050	28209	9.855	0.0131	1.957	0.0127	103.10	0.56
λ01.5: l400 vs. l100	83924	9.179	0.0076	2.046	0.0078	203.08	0.32
λ01.5: l400 vs. l200	164245	9.267	0.0054	2.110	0.0058	300.92	0.45
λ01.5: l400 vs. l400	117009	10.031	0.0068	2.197	0.0071	263.58	0.40
λ01.5: l600 vs. l050	20821	10.712	0.0152	1.950	0.0147	102.92	0.45
λ01.5: l600 vs. l100	59538	9.983	0.0093	2.102	0.0094	174.30	0.37
λ01.5: l600 vs. l200	119719	10.118	0.0066	2.153	0.0069	254.01	0.33
λ01.5: l600 vs. l400	177765	10.765	0.0056	2.196	0.0057	328.42	0.33
λ01.5: l600 vs. l600	49440	11.449	0.0106	2.240	0.0111	131.74	0.54
λ01.5: l800 vs. l050	21563	11.221	0.0150	1.907	0.0140	104.84	0.39
λ01.5: l800 vs. l100	60316	10.552	0.0096	2.116	0.0094	182.54	0.42
λ01.5: l800 vs. l200	121911	10.742	0.0068	2.170	0.0068	267.84	0.33
λ01.5: l800 vs. l400	187728	11.367	0.0055	2.199	0.0055	337.93	0.40
λ01.5: l800 vs. l600	101848	12.069	0.0077	2.251	0.0077	234.47	0.33
λ01.5: l800 vs. l800	52605	12.666	0.0109	2.217	0.0104	173.83	0.40
λ01.6: l050 vs. l050	3754	6.834	0.0253	1.404	0.0252	51.65	0.37
λ01.6: l100 vs. l050	12055	7.447	0.0166	1.651	0.0165	69.24	0.51
λ01.6: l100 vs. l100	15074	7.166	0.0153	1.755	0.0158	84.83	0.36
λ01.6: l200 vs. l050	14637	8.513	0.0174	1.875	0.0169	75.56	0.55
λ01.6: l200 vs. l100	44147	7.835	0.0097	1.942	0.0103	142.04	0.38
λ01.6: l200 vs. l200	40764	7.918	0.0101	2.013	0.0112	113.01	0.51
λ01.6: l400 vs. l050	16380	9.651	0.0172	1.963	0.0167	85.46	0.42
λ01.6: l400 vs. l100	46974	8.930	0.0103	2.089	0.0106	149.41	0.36
λ01.6: l400 vs. l200	94741	8.859	0.0071	2.107	0.0076	218.34	0.40
λ01.6: l400 vs. l400	66910	9.451	0.0086	2.174	0.0093	187.62	0.40
λ01.6: l600 vs. l050	10342	10.568	0.0209	1.847	0.0196	60.60	0.46
λ01.6: l600 vs. l100	28504	9.794	0.0137	2.118	0.0137	118.36	0.39
λ01.6: l600 vs. l200	56773	9.769	0.0094	2.127	0.0098	168.90	0.40
λ01.6: l600 vs. l400	77356	10.183	0.0082	2.166	0.0086	203.35	0.35
λ01.6: l600 vs. l600	6208	10.813	0.0292	2.225	0.0313	49.79	0.50
λ01.6: l800 vs. l050	10691	11.078	0.0207	1.854	0.0193	45.04	0.92
λ01.6: l800 vs. l100	28723	10.373	0.0141	2.132	0.0136	123.88	0.38
λ01.6: l800 vs. l200	56686	10.342	0.0097	2.145	0.0099	162.48	0.43
λ01.6: l800 vs. l400	75498	10.727	0.0085	2.166	0.0086	209.52	0.36
λ01.6: l800 vs. l600	9734	11.362	0.0239	2.255	0.0252	63.27	0.45

continues ...

(continued)

Distribution	N	Location		Scale		AD_{up}	p-value
		Estimate	SE	Estimate	SE		
λ01.6: l800 vs. l800	3838	11.859	0.0397	2.283	0.0401	56.91	0.37
λ01.7: l050 vs. l050	2237	6.764	0.0320	1.389	0.0323	27.67	0.59
λ01.7: l100 vs. l050	6949	7.348	0.0218	1.630	0.0214	47.86	0.38
λ01.7: l100 vs. l100	8710	6.975	0.0201	1.760	0.0209	59.23	0.41
λ01.7: l200 vs. l050	8446	8.432	0.0227	1.916	0.0229	59.63	0.44
λ01.7: l200 vs. l100	24238	7.607	0.0129	1.944	0.0139	106.02	0.37
λ01.7: l200 vs. l200	20755	7.616	0.0135	1.969	0.0154	134.23	0.28
λ01.7: l400 vs. l050	9321	9.557	0.0230	1.981	0.0223	74.31	0.40
λ01.7: l400 vs. l100	24486	8.682	0.0141	2.093	0.0148	106.35	0.46
λ01.7: l400 vs. l200	41490	8.465	0.0102	2.091	0.0115	129.44	0.53
λ01.7: l400 vs. l400	23608	8.922	0.0135	2.079	0.0152	96.08	0.49
λ01.7: l600 vs. l050	5104	10.555	0.0308	1.959	0.0297	44.03	0.52
λ01.7: l600 vs. l100	13272	9.663	0.0199	2.102	0.0199	76.34	0.33
λ01.7: l600 vs. l200	18809	9.436	0.0161	2.112	0.0170	95.13	0.36
λ01.7: l600 vs. l400	20209	9.565	0.0151	2.108	0.0165	92.61	0.43
λ01.7: l800 vs. l050	5353	11.105	0.0298	1.924	0.0285	53.90	0.39
λ01.7: l800 vs. l100	13165	10.186	0.0203	2.095	0.0198	75.15	0.59
λ01.7: l800 vs. l200	17389	10.003	0.0174	2.159	0.0180	85.75	0.43
λ01.7: l800 vs. l400	17855	10.072	0.0165	2.127	0.0176	85.76	0.41
λ01.8: l100 vs. l050	4181	7.293	0.0271	1.611	0.0275	43.57	0.40
λ01.8: l100 vs. l100	5156	6.917	0.0256	1.727	0.0267	48.05	0.40
λ01.8: l200 vs. l050	4772	8.372	0.0296	1.858	0.0295	176.91	0.11
λ01.8: l200 vs. l100	12945	7.461	0.0171	1.898	0.0186	80.61	0.41
λ01.8: l200 vs. l200	8651	7.414	0.0200	1.893	0.0230	68.74	0.36
λ01.8: l400 vs. l050	5204	9.511	0.0314	2.047	0.0310	51.80	0.40
λ01.8: l400 vs. l100	11908	8.520	0.0195	2.034	0.0206	61.39	0.60
λ01.8: l400 vs. l200	14024	8.205	0.0167	2.002	0.0190	80.30	0.40
λ01.8: l400 vs. l400	5184	8.540	0.0271	2.018	0.0318	180.18	0.19
λ01.8: l600 vs. l050	3003	10.546	0.0416	2.056	0.0408	39.47	0.42
λ01.8: l600 vs. l100	5918	9.510	0.0291	2.040	0.0289	57.86	0.26
λ01.8: l600 vs. l200	5253	9.296	0.0309	2.202	0.0339	52.40	0.43
λ01.8: l600 vs. l400	2818	8.998	0.0369	1.941	0.0410	37.46	0.41
λ01.8: l800 vs. l050	2951	11.225	0.0432	1.987	0.0392	42.13	0.37
λ01.8: l800 vs. l100	5698	10.050	0.0296	2.000	0.0287	57.74	0.32
λ01.8: l800 vs. l200	4772	9.823	0.0327	2.169	0.0347	50.20	0.39
λ01.8: l800 vs. l400	2196	9.321	0.0433	2.044	0.0489	444.57	0.01
λ01.9: l100 vs. l050	2460	7.193	0.0355	1.615	0.0359	34.30	0.37
λ01.9: l100 vs. l100	2947	6.840	0.0323	1.683	0.0344	34.53	0.37
λ01.9: l200 vs. l050	2790	8.252	0.0379	1.849	0.0386	39.12	0.37
λ01.9: l200 vs. l100	6018	7.371	0.0238	1.756	0.0250	53.54	0.39
λ01.9: l200 vs. l200	2771	7.252	0.0327	1.709	0.0366	38.82	0.27
λ01.9: l400 vs. l050	2820	9.363	0.0418	2.062	0.0426	31.58	0.50
λ01.9: l400 vs. l100	5071	8.381	0.0284	1.951	0.0304	53.23	0.36

continues . . .

(continued)

Distribution	N	Location		Scale		AD_{up}	p-value
		Estimate	SE	Estimate	SE		
λ01.9: l400 vs. l200	3466	8.035	0.0325	1.941	0.0372	39.72	0.41
λ01.9: l600 vs. l100	2398	9.251	0.0412	1.819	0.0404	33.42	0.33
λ01.9: l800 vs. l100	2272	9.793	0.0427	1.907	0.0440	34.41	0.33
λ02.0: l100 vs. l100	1607	6.683	0.0410	1.523	0.0419	25.62	0.44
λ02.0: l200 vs. l100	2530	7.219	0.0344	1.644	0.0362	35.67	0.30
λ02.0: l400 vs. l100	1889	8.099	0.0416	1.676	0.0423	25.73	0.48

Distribution represents distributions obtained from aligning pairs of simulated profiles with mutual compositional similarity λ and different values of length l. The table reports the estimates and their standard errors (SE) for the location and scale parameters of the EVD for each distribution of alignment scores. N is the number of alignment scores. AD_{up} is the upper-tail Anderson-Darling statistic. The p-value of statistic AD_{up} was computed by Monte Carlo simulation with 100 samples.
