# Supplementary material for: Estimating statistical significance of local protein profile-profile alignments
Source: BMC Bioinformatics. 2019 Aug 13;20:419. doi: 10.1186/s12859-019-2913-3 (PMC6693267; doi:10.1186/s12859-019-2913-3)
Supplement: Supplementary file 12 — Table S5. Goodness of fit of the NBD to the distribution of the number of positive substitution scores observed in alignments of simulated profiles. (PDF 67 kb) [file 12859_2019_2913_MOESM12_ESM.pdf]

Table S5. Goodness of fit of the negative binomial distribution (NBD) to the distribution of the number of positive substitution scores observed in alignments of simulated profiles

Distribution	N	Shape		Probability		AD_{up}	p-value
		Estimate	SE	Estimate	SE		
n04 l050 vs. n02 l050	9385	7.544	0.1317	0.483	0.0045	171.76	0.37
n04 l050 vs. n02 l100	12564	8.568	0.2398	0.501	0.0071	158.38	0.38
n04 l050 vs. n02 l200	11910	9.553	0.2921	0.498	0.0077	93.07	0.67
n04 l050 vs. n02 l400	9902	9.537	0.3077	0.522	0.0082	74.29	0.75
n04 l050 vs. n02 l600	8559	9.328	0.2542	0.536	0.0069	81.27	0.55
n04 l050 vs. n02 l800	7718	10.241	0.2877	0.520	0.0071	75.53	0.59
n04 l100 vs. n02 l050	12109	8.646	0.2236	0.494	0.0066	77.84	0.90
n04 l100 vs. n02 l100	25308	3.841	0.0381	0.704	0.0022	125.55	0.48
n04 l100 vs. n02 l200	31604	3.272	0.0271	0.754	0.0016	125.59	0.68
n04 l100 vs. n02 l400	30651	4.133	0.0393	0.744	0.0019	119.38	0.73
n04 l100 vs. n02 l600	28457	4.514	0.0479	0.743	0.0021	124.14	0.64
n04 l100 vs. n02 l800	26709	4.521	0.0495	0.754	0.0021	138.84	0.57
n04 l200 vs. n02 l050	12060	8.649	0.2418	0.519	0.0071	78.82	0.74
n04 l200 vs. n02 l100	32556	3.319	0.0276	0.752	0.0017	261.14	0.38
n04 l200 vs. n02 l200	48745	2.246	0.0137	0.825	0.0010	913.89	0.06
n04 l200 vs. n02 l400	51556	2.615	0.0164	0.834	0.0009	192.23	0.55
n04 l200 vs. n02 l600	49377	2.439	0.0153	0.852	0.0009	168.55	0.51
n04 l200 vs. n02 l800	47628	2.703	0.0178	0.849	0.0009	164.38	0.51
n04 l400 vs. n02 l050	11639	8.602	0.2458	0.543	0.0072	88.65	0.61
n04 l400 vs. n02 l100	35532	3.574	0.0310	0.773	0.0016	112.05	0.86
n04 l400 vs. n02 l200	54148	1.872	0.0107	0.872	0.0007	171.63	0.49
n04 l400 vs. n02 l400	60074	1.665	0.0089	0.899	0.0006	163.71	0.66
n04 l400 vs. n02 l600	60056	1.921	0.0106	0.900	0.0006	201.65	0.45
n04 l400 vs. n02 l800	59238	1.636	0.0088	0.914	0.0005	193.00	0.48
n04 l600 vs. n02 l050	10457	9.965	0.3429	0.516	0.0087	93.45	0.51
n04 l600 vs. n02 l100	34905	3.193	0.0262	0.802	0.0014	160.50	0.52
n04 l600 vs. n02 l200	54673	2.062	0.0121	0.878	0.0007	190.51	0.39
n04 l600 vs. n02 l400	61190	1.482	0.0077	0.918	0.0005	139.26	0.81
n04 l600 vs. n02 l600	61900	1.431	0.0074	0.928	0.0004	199.13	0.48
n04 l600 vs. n02 l800	61797	1.827	0.0099	0.922	0.0004	167.31	0.64
n04 l800 vs. n02 l050	9514	8.225	0.2127	0.570	0.0064	83.24	0.61
n04 l800 vs. n02 l100	32887	3.588	0.0323	0.796	0.0015	167.02	0.49
n04 l800 vs. n02 l200	53404	2.241	0.0137	0.880	0.0007	189.99	0.49
n04 l800 vs. n02 l400	61169	1.281	0.0066	0.930	0.0004	114.23	0.96
n04 l800 vs. n02 l600	62130	1.370	0.0071	0.936	0.0004	247.59	0.46
n04 l800 vs. n02 l800	62260	1.341	0.0069	0.942	0.0003	195.39	0.50
n06 l050 vs. n02 l050	5248	10.083	0.2973	0.431	0.0073	71.25	0.64
n06 l050 vs. n02 l100	7527	8.887	0.2992	0.498	0.0085	99.15	0.59
n06 l050 vs. n02 l200	7075	7.448	0.2185	0.562	0.0074	77.64	0.53
n06 l050 vs. n02 l400	5876	7.777	0.2890	0.571	0.0093	59.20	0.74

continues . . .

(continued)

Distribution	N	Shape		Probability		AD_{up}	p-value
		Estimate	SE	Estimate	SE		
n06 l050 vs. n02 l600	5083	8.033	0.2814	0.562	0.0088	56.41	0.75
n06 l050 vs. n02 l800	4720	8.215	0.3772	0.569	0.0114	54.54	0.70
n06 l050 vs. n04 l050	3327	9.484	0.4730	0.446	0.0125	32.73	0.99
n06 l050 vs. n04 l100	4486	6.687	0.2081	0.555	0.0079	35.25	0.99
n06 l050 vs. n04 l200	5022	6.851	0.2445	0.563	0.0090	51.42	0.81
n06 l050 vs. n04 l400	5338	5.379	0.1538	0.622	0.0069	61.54	0.51
n06 l050 vs. n04 l600	5661	5.993	0.2055	0.600	0.0084	54.55	0.69
n06 l050 vs. n04 l800	5074	5.693	0.1937	0.612	0.0083	45.21	0.84
n06 l100 vs. n02 l050	6534	5.909	0.1188	0.580	0.0051	63.98	0.69
n06 l100 vs. n02 l100	17164	4.288	0.0550	0.682	0.0029	110.89	0.66
n06 l100 vs. n02 l200	23182	3.433	0.0342	0.739	0.0020	99.95	0.88
n06 l100 vs. n02 l400	22167	3.132	0.0309	0.774	0.0019	102.22	0.72
n06 l100 vs. n02 l600	20251	3.796	0.0452	0.759	0.0023	85.97	0.80
n06 l100 vs. n02 l800	18797	3.580	0.0426	0.778	0.0022	115.80	0.49
n06 l100 vs. n04 l050	4252	6.896	0.2400	0.545	0.0088	97.50	0.35
n06 l100 vs. n04 l100	10352	3.251	0.0459	0.720	0.0030	96.89	0.51
n06 l100 vs. n04 l200	15830	3.520	0.0443	0.725	0.0027	163.66	0.44
n06 l100 vs. n04 l400	19153	2.895	0.0309	0.761	0.0021	132.18	0.48
n06 l100 vs. n04 l600	19962	2.600	0.0267	0.782	0.0019	231.71	0.22
n06 l100 vs. n04 l800	18603	2.115	0.0213	0.813	0.0017	59.46	0.99
n06 l200 vs. n02 l050	5611	8.239	0.2665	0.532	0.0082	63.12	0.67
n06 l200 vs. n02 l100	20569	3.681	0.0404	0.732	0.0023	70.01	0.98
n06 l200 vs. n02 l200	39529	2.725	0.0194	0.790	0.0013	148.42	0.53
n06 l200 vs. n02 l400	44253	2.590	0.0172	0.814	0.0011	137.11	0.71
n06 l200 vs. n02 l600	41553	2.201	0.0146	0.842	0.0010	513.08	0.20
n06 l200 vs. n02 l800	39362	2.365	0.0165	0.842	0.0010	321.68	0.32
n06 l200 vs. n04 l050	3933	7.183	0.3008	0.553	0.0106	41.73	0.74
n06 l200 vs. n04 l100	12705	4.217	0.0654	0.696	0.0034	52.43	1.00
n06 l200 vs. n04 l200	27641	2.569	0.0215	0.780	0.0016	116.11	0.66
n06 l200 vs. n04 l400	37077	2.262	0.0162	0.809	0.0012	192.79	0.43
n06 l200 vs. n04 l600	38551	2.021	0.0139	0.827	0.0011	134.11	0.70
n06 l200 vs. n04 l800	37098	2.109	0.0151	0.830	0.0011	222.97	0.34
n06 l400 vs. n02 l050	4663	7.285	0.2462	0.572	0.0084	41.75	0.88
n06 l400 vs. n02 l100	19508	3.626	0.0416	0.754	0.0023	90.74	0.77
n06 l400 vs. n02 l200	42382	2.309	0.0154	0.827	0.0011	163.99	0.50
n06 l400 vs. n02 l400	56690	1.875	0.0103	0.859	0.0008	155.72	0.64
n06 l400 vs. n02 l600	57760	2.159	0.0122	0.856	0.0008	355.32	0.27
n06 l400 vs. n02 l800	56482	2.243	0.0130	0.858	0.0008	209.55	0.45
n06 l400 vs. n04 l050	3999	4.904	0.1397	0.634	0.0069	53.32	0.57
n06 l400 vs. n04 l100	13619	3.015	0.0393	0.762	0.0025	77.78	0.76
n06 l400 vs. n04 l200	31859	2.190	0.0166	0.812	0.0013	111.63	0.91
n06 l400 vs. n04 l400	49071	1.673	0.0098	0.847	0.0009	722.85	0.13

continues ...

(continued)

Distribution	N	Shape		Probability		AD_{up}	p-value
		Estimate	SE	Estimate	SE		
n06 l400 vs. n04 l600	53901	1.773	0.0102	0.851	0.0008	116.11	0.94
n06 l400 vs. n04 l800	54042	1.625	0.0092	0.864	0.0008	144.82	0.77
n06 l600 vs. n02 l050	4328	7.265	0.3298	0.584	0.0112	56.93	0.68
n06 l600 vs. n02 l100	18816	3.537	0.0411	0.766	0.0022	67.07	0.96
n06 l600 vs. n02 l200	41200	1.914	0.0124	0.852	0.0009	288.45	0.31
n06 l600 vs. n02 l400	57142	1.769	0.0097	0.872	0.0007	197.50	0.38
n06 l600 vs. n02 l600	60615	1.934	0.0106	0.875	0.0007	382.13	0.25
n06 l600 vs. n02 l800	60771	1.814	0.0097	0.884	0.0006	192.01	0.54
n06 l600 vs. n04 l050	3983	5.591	0.2246	0.618	0.0098	50.51	0.55
n06 l600 vs. n04 l100	13921	2.767	0.0344	0.775	0.0024	62.09	0.91
n06 l600 vs. n04 l200	32198	2.226	0.0171	0.817	0.0013	231.48	0.27
n06 l600 vs. n04 l400	49813	1.735	0.0104	0.854	0.0009	133.63	0.81
n06 l600 vs. n04 l600	56550	1.433	0.0078	0.875	0.0007	149.71	0.75
n06 l600 vs. n04 l800	58443	1.653	0.0091	0.867	0.0007	408.77	0.21
n06 l800 vs. n02 l050	4002	6.342	0.2493	0.612	0.0096	55.77	0.59
n06 l800 vs. n02 l100	18690	3.283	0.0379	0.784	0.0021	93.52	0.68
n06 l800 vs. n02 l200	40608	2.351	0.0164	0.840	0.0010	134.73	0.63
n06 l800 vs. n02 l400	56171	1.799	0.0100	0.878	0.0007	439.98	0.25
n06 l800 vs. n02 l600	60693	1.818	0.0099	0.885	0.0006	130.15	0.90
n06 l800 vs. n02 l800	61603	1.632	0.0086	0.896	0.0006	632.69	0.16
n06 l800 vs. n04 l050	4056	5.702	0.1979	0.601	0.0086	53.34	0.58
n06 l800 vs. n04 l100	14440	2.305	0.0265	0.801	0.0020	71.63	0.86
n06 l800 vs. n04 l200	32501	1.582	0.0112	0.852	0.0010	122.65	0.71
n06 l800 vs. n04 l400	49631	1.488	0.0087	0.869	0.0008	112.39	0.95
n06 l800 vs. n04 l600	56800	1.432	0.0078	0.879	0.0007	1488.34	0.06
n06 l800 vs. n04 l800	59207	1.350	0.0071	0.885	0.0006	97.98	1.00
n08 l050 vs. n02 l050	4034	9.129	0.3333	0.450	0.0092	64.98	0.55
n08 l050 vs. n02 l100	5807	6.480	0.1916	0.564	0.0074	64.18	0.55
n08 l050 vs. n02 l200	5604	7.035	0.2550	0.564	0.0091	53.78	0.77
n08 l050 vs. n02 l400	4691	6.335	0.2371	0.599	0.0092	51.89	0.60
n08 l050 vs. n02 l600	4131	5.875	0.2286	0.625	0.0094	49.95	0.68
n08 l050 vs. n02 l800	3729	5.671	0.1865	0.626	0.0079	48.46	0.56
n08 l050 vs. n04 l050	2805	7.739	0.3062	0.487	0.0101	59.50	0.49
n08 l050 vs. n04 l100	3908	5.252	0.1514	0.597	0.0072	34.31	0.99
n08 l050 vs. n04 l200	4380	5.975	0.1904	0.575	0.0080	38.07	0.95
n08 l050 vs. n04 l400	4613	4.932	0.1369	0.623	0.0068	73.61	0.47
n08 l050 vs. n04 l600	4938	5.657	0.1744	0.595	0.0077	105.91	0.26
n08 l050 vs. n04 l800	4480	6.387	0.2430	0.569	0.0096	53.48	0.67
n08 l050 vs. n06 l050	2867	6.398	0.2481	0.534	0.0099	44.92	0.78
n08 l050 vs. n06 l100	4378	5.865	0.1843	0.571	0.0079	49.34	0.76
n08 l050 vs. n06 l200	4483	5.510	0.1734	0.602	0.0078	55.76	0.67
n08 l050 vs. n06 l400	4923	6.526	0.2396	0.569	0.0092	38.25	0.94

continues ...

(continued)

Distribution	N	Shape		Probability		AD_{up}	p-value
		Estimate	SE	Estimate	SE		
n08 l050 vs. n06 l600	5211	6.403	0.2107	0.570	0.0083	44.26	0.88
n08 l050 vs. n06 l800	5388	4.878	0.1072	0.623	0.0054	55.88	0.68
n08 l100 vs. n02 l050	4939	7.404	0.2472	0.524	0.0085	97.33	0.41
n08 l100 vs. n02 l100	13764	4.406	0.0670	0.669	0.0035	168.74	0.33
n08 l100 vs. n02 l200	18807	3.065	0.0328	0.749	0.0022	138.02	0.49
n08 l100 vs. n02 l400	17111	2.796	0.0315	0.786	0.0021	104.97	0.52
n08 l100 vs. n02 l600	15345	3.873	0.0559	0.744	0.0029	91.14	0.55
n08 l100 vs. n02 l800	14048	3.890	0.0587	0.752	0.0030	63.59	0.91
n08 l100 vs. n04 l050	3645	7.852	0.3624	0.502	0.0117	74.47	0.54
n08 l100 vs. n04 l100	9014	3.500	0.0561	0.698	0.0036	48.37	0.95
n08 l100 vs. n04 l200	13402	3.261	0.0431	0.725	0.0028	90.05	0.61
n08 l100 vs. n04 l400	15747	2.856	0.0334	0.752	0.0024	87.27	0.68
n08 l100 vs. n04 l600	16250	2.098	0.0226	0.800	0.0019	88.40	0.69
n08 l100 vs. n04 l800	14876	2.175	0.0242	0.797	0.0020	68.11	0.92
n08 l100 vs. n06 l050	4240	5.751	0.1898	0.576	0.0083	41.65	0.88
n08 l100 vs. n06 l100	10546	3.193	0.0467	0.712	0.0032	114.60	0.44
n08 l100 vs. n06 l200	13602	3.102	0.0407	0.735	0.0027	79.40	0.77
n08 l100 vs. n06 l400	14576	2.454	0.0286	0.777	0.0022	81.38	0.60
n08 l100 vs. n06 l600	14593	2.049	0.0227	0.804	0.0020	129.98	0.39
n08 l100 vs. n06 l800	15408	2.051	0.0223	0.805	0.0019	64.53	0.95
n08 l200 vs. n02 l050	4967	7.710	0.3468	0.530	0.0114	52.77	0.79
n08 l200 vs. n02 l100	18948	2.819	0.0292	0.755	0.0021	85.65	0.76
n08 l200 vs. n02 l200	34763	2.582	0.0196	0.784	0.0014	100.22	0.86
n08 l200 vs. n02 l400	37416	2.072	0.0144	0.827	0.0011	110.95	0.82
n08 l200 vs. n02 l600	34391	2.190	0.0164	0.832	0.0012	107.03	0.82
n08 l200 vs. n02 l800	32029	2.017	0.0152	0.845	0.0011	108.98	0.80
n08 l200 vs. n04 l050	4041	5.641	0.1892	0.588	0.0084	43.00	0.82
n08 l200 vs. n04 l100	13282	2.624	0.0315	0.751	0.0025	65.28	0.87
n08 l200 vs. n04 l200	25989	2.789	0.0252	0.755	0.0018	145.64	0.48
n08 l200 vs. n04 l400	32222	2.444	0.0195	0.786	0.0015	31485.90	0.00
n08 l200 vs. n04 l600	33615	1.900	0.0140	0.820	0.0012	103.08	0.92
n08 l200 vs. n04 l800	31928	1.528	0.0110	0.844	0.0011	291.95	0.27
n08 l200 vs. n06 l050	4935	5.529	0.1665	0.590	0.0075	52.83	0.65
n08 l200 vs. n06 l100	16109	2.712	0.0311	0.749	0.0023	154.33	0.48
n08 l200 vs. n06 l200	27981	2.123	0.0170	0.791	0.0015	83.42	0.96
n08 l200 vs. n06 l400	31622	2.250	0.0177	0.799	0.0014	101.35	0.92
n08 l200 vs. n06 l600	31266	2.114	0.0165	0.811	0.0013	120.79	0.68
n08 l200 vs. n06 l800	31776	1.772	0.0132	0.833	0.0012	116.57	0.72
n08 l400 vs. n02 l050	4759	5.479	0.1775	0.615	0.0079	58.68	0.49
n08 l400 vs. n02 l100	20264	2.886	0.0305	0.766	0.0020	137.52	0.42
n08 l400 vs. n02 l200	41181	2.040	0.0137	0.831	0.0011	97.72	0.97
n08 l400 vs. n02 l400	51869	1.709	0.0099	0.861	0.0008	146.82	0.66

continues . . .

(continued)

Distribution	N	Shape		Probability		AD_{up}	p-value
		Estimate	SE	Estimate	SE		
n08 l400 vs. n02 l600	51414	1.720	0.0101	0.868	0.0008	413.23	0.32
n08 l400 vs. n02 l800	49319	1.791	0.0108	0.871	0.0008	533.55	0.23
n08 l400 vs. n04 l050	4702	4.789	0.1256	0.614	0.0065	63.22	0.56
n08 l400 vs. n04 l100	16512	2.613	0.0293	0.764	0.0022	90.43	0.54
n08 l400 vs. n04 l200	33598	2.259	0.0170	0.793	0.0014	2541.33	0.02
n08 l400 vs. n04 l400	45229	1.428	0.0086	0.854	0.0009	118.28	0.87
n08 l400 vs. n04 l600	48126	1.104	0.0062	0.876	0.0008	1127.89	0.07
n08 l400 vs. n04 l800	47798	1.438	0.0085	0.861	0.0008	416.41	0.23
n08 l400 vs. n06 l050	6002	5.078	0.1235	0.609	0.0060	78.48	0.56
n08 l400 vs. n06 l100	19035	1.907	0.0184	0.801	0.0018	86.61	0.89
n08 l400 vs. n06 l200	35336	2.055	0.0150	0.809	0.0013	110.91	0.86
n08 l400 vs. n06 l400	46954	1.389	0.0082	0.856	0.0009	141.53	0.72
n08 l400 vs. n06 l600	47662	1.271	0.0073	0.867	0.0008	142.12	0.73
n08 l400 vs. n06 l800	47746	1.318	0.0077	0.869	0.0008	151.27	0.66
n08 l600 vs. n02 l050	4224	5.714	0.2001	0.609	0.0086	54.37	0.54
n08 l600 vs. n02 l100	18734	3.208	0.0374	0.762	0.0023	253.24	0.19
n08 l600 vs. n02 l200	40281	1.895	0.0127	0.846	0.0010	507.06	0.12
n08 l600 vs. n02 l400	54849	1.508	0.0083	0.878	0.0007	1190.55	0.10
n08 l600 vs. n02 l600	57671	1.827	0.0103	0.873	0.0007	161.43	0.74
n08 l600 vs. n02 l800	57225	1.663	0.0092	0.885	0.0007	175.24	0.60
n08 l600 vs. n04 l050	4610	4.766	0.1215	0.617	0.0063	71.10	0.39
n08 l600 vs. n04 l100	16216	2.604	0.0296	0.768	0.0022	286.66	0.17
n08 l600 vs. n04 l200	34280	2.093	0.0154	0.810	0.0013	89.32	0.96
n08 l600 vs. n04 l400	49109	1.345	0.0077	0.864	0.0008	1174.07	0.13
n08 l600 vs. n04 l600	53520	1.441	0.0081	0.866	0.0008	488.18	0.18
n08 l600 vs. n04 l800	54446	1.332	0.0073	0.874	0.0007	357.94	0.35
n08 l600 vs. n06 l050	6287	4.964	0.1204	0.623	0.0059	56.89	0.72
n08 l600 vs. n06 l100	19436	2.285	0.0231	0.786	0.0019	182.07	0.30
n08 l600 vs. n06 l200	35461	1.718	0.0120	0.833	0.0011	84.06	0.99
n08 l600 vs. n06 l400	51170	1.479	0.0084	0.856	0.0008	178.12	0.49
n08 l600 vs. n06 l600	54751	1.229	0.0066	0.877	0.0007	211.17	0.43
n08 l600 vs. n06 l800	55297	1.371	0.0075	0.874	0.0007	141.65	0.82
n08 l800 vs. n02 l050	3801	5.056	0.1722	0.639	0.0081	51.37	0.57
n08 l800 vs. n02 l100	16811	2.740	0.0318	0.790	0.0021	89.38	0.63
n08 l800 vs. n02 l200	37360	1.689	0.0115	0.860	0.0009	122.92	0.77
n08 l800 vs. n02 l400	55096	1.649	0.0092	0.876	0.0007	99.37	0.98
n08 l800 vs. n02 l600	59086	1.461	0.0078	0.891	0.0006	139.42	0.86
n08 l800 vs. n02 l800	59997	1.772	0.0098	0.884	0.0006	143.05	0.86
n08 l800 vs. n04 l050	4612	4.748	0.1322	0.623	0.0068	56.12	0.66
n08 l800 vs. n04 l100	15478	2.394	0.0271	0.781	0.0021	66.69	0.92
n08 l800 vs. n04 l200	32262	1.739	0.0127	0.833	0.0012	316.40	0.16
n08 l800 vs. n04 l400	49720	1.751	0.0106	0.844	0.0009	182.89	0.48

continues . . .

(continued)

Distribution	N	Shape		Probability		AD_{up}	p-value
		Estimate	SE	Estimate	SE		
n08 l800 vs. n04 l600	55360	1.712	0.0099	0.856	0.0008	198.69	0.50
n08 l800 vs. n04 l800	57031	1.309	0.0070	0.879	0.0007	165.87	0.74
n08 l800 vs. n06 l050	6349	5.543	0.1254	0.589	0.0057	246.43	0.20
n08 l800 vs. n06 l100	18962	1.946	0.0188	0.805	0.0017	91.55	0.74
n08 l800 vs. n06 l200	34291	1.719	0.0123	0.837	0.0011	396.68	0.17
n08 l800 vs. n06 l400	52083	1.117	0.0061	0.881	0.0007	341.47	0.24
n08 l800 vs. n06 l600	57046	1.073	0.0055	0.887	0.0007	145.46	0.84
n08 l800 vs. n06 l800	58127	1.086	0.0056	0.890	0.0006	285.86	0.29
n10 l050 vs. n02 l050	3475	7.628	0.2846	0.472	0.0095	73.90	0.48
n10 l050 vs. n02 l100	5216	5.930	0.1694	0.559	0.0072	57.38	0.74
n10 l050 vs. n02 l200	5312	5.675	0.1761	0.585	0.0077	60.14	0.57
n10 l050 vs. n02 l400	4693	6.078	0.2012	0.583	0.0083	44.48	0.85
n10 l050 vs. n02 l600	4315	6.046	0.2241	0.587	0.0092	48.46	0.70
n10 l050 vs. n02 l800	4169	6.186	0.2381	0.586	0.0096	43.14	0.72
n10 l050 vs. n04 l050	2976	6.870	0.2671	0.502	0.0099	52.54	0.62
n10 l050 vs. n04 l100	4357	5.580	0.1533	0.563	0.0070	35.70	1.00
n10 l050 vs. n04 l200	4915	5.533	0.1523	0.578	0.0069	89.42	0.32
n10 l050 vs. n04 l400	5785	6.400	0.1991	0.554	0.0079	111.71	0.35
n10 l050 vs. n04 l600	6614	4.618	0.1051	0.628	0.0056	60.35	0.67
n10 l050 vs. n04 l800	6193	5.178	0.1125	0.598	0.0054	215.43	0.15
n10 l050 vs. n06 l050	2911	5.531	0.1874	0.540	0.0087	68.59	0.43
n10 l050 vs. n06 l100	4445	3.229	0.0685	0.662	0.0051	84.87	0.37
n10 l050 vs. n06 l200	4798	4.479	0.1077	0.618	0.0060	50.46	0.66
n10 l050 vs. n06 l400	5794	6.467	0.1877	0.544	0.0074	299.33	0.09
n10 l050 vs. n06 l600	6422	4.893	0.1078	0.612	0.0055	67.61	0.67
n10 l050 vs. n06 l800	7087	6.000	0.1484	0.569	0.0063	64.74	0.67
n10 l050 vs. n08 l050	2814	6.267	0.2114	0.505	0.0087	52.42	0.61
n10 l050 vs. n08 l100	4273	7.997	0.3176	0.469	0.0100	610.51	0.04
n10 l050 vs. n08 l200	5433	5.990	0.1697	0.546	0.0072	61.80	0.55
n10 l050 vs. n08 l400	7336	5.376	0.1148	0.575	0.0054	143.09	0.32
n10 l050 vs. n08 l600	7970	5.595	0.1135	0.569	0.0052	91.79	0.59
n10 l050 vs. n08 l800	8393	4.336	0.0667	0.619	0.0038	60.14	0.87
n10 l100 vs. n02 l050	5295	5.572	0.1643	0.569	0.0074	76.29	0.40
n10 l100 vs. n02 l100	13773	3.545	0.0500	0.691	0.0032	68.15	0.94
n10 l100 vs. n02 l200	17407	3.150	0.0385	0.735	0.0025	76.49	0.90
n10 l100 vs. n02 l400	16105	3.248	0.0428	0.749	0.0026	88.21	0.68
n10 l100 vs. n02 l600	14592	2.786	0.0357	0.777	0.0024	96.83	0.59
n10 l100 vs. n02 l800	13701	3.051	0.0423	0.769	0.0026	69.24	0.85
n10 l100 vs. n04 l050	4526	5.938	0.1986	0.554	0.0085	140.84	0.23
n10 l100 vs. n04 l100	10830	2.887	0.0401	0.718	0.0030	80.17	0.59
n10 l100 vs. n04 l200	14802	2.818	0.0338	0.737	0.0025	54.68	0.97
n10 l100 vs. n04 l400	16630	2.431	0.0271	0.768	0.0022	87.92	0.70

continues ...

(continued)

Distribution	N	Shape		Probability		AD_{up}	p-value
		Estimate	SE	Estimate	SE		
n10 l100 vs. n04 l600	17466	2.202	0.0235	0.787	0.0020	261.01	0.25
n10 l100 vs. n04 l800	16013	2.471	0.0282	0.772	0.0022	179.81	0.34
n10 l100 vs. n06 l050	4507	5.052	0.1462	0.574	0.0073	51.84	0.73
n10 l100 vs. n06 l100	10488	3.059	0.0458	0.696	0.0034	484.06	0.06
n10 l100 vs. n06 l200	13017	2.657	0.0336	0.734	0.0027	116.67	0.51
n10 l100 vs. n06 l400	14823	2.493	0.0289	0.752	0.0024	78.77	0.77
n10 l100 vs. n06 l600	15620	2.038	0.0215	0.780	0.0020	172.62	0.29
n10 l100 vs. n06 l800	16514	2.454	0.0265	0.755	0.0022	158.95	0.31
n10 l100 vs. n08 l050	4573	4.788	0.1243	0.586	0.0065	50.37	0.70
n10 l100 vs. n08 l100	10205	2.487	0.0343	0.724	0.0030	49.59	0.97
n10 l100 vs. n08 l200	14369	3.432	0.0477	0.685	0.0032	87.41	0.71
n10 l100 vs. n08 l400	17369	2.201	0.0232	0.763	0.0021	140.47	0.39
n10 l100 vs. n08 l600	18502	1.932	0.0189	0.785	0.0019	116.00	0.46
n10 l100 vs. n08 l800	18647	1.817	0.0171	0.789	0.0018	340.11	0.11
n10 l200 vs. n02 l050	5057	4.999	0.1361	0.603	0.0068	45.19	0.85
n10 l200 vs. n02 l100	19281	2.690	0.0287	0.749	0.0022	88.71	0.85
n10 l200 vs. n02 l200	33290	2.205	0.0169	0.792	0.0014	98.35	0.94
n10 l200 vs. n02 l400	33446	2.025	0.0154	0.823	0.0012	189.60	0.35
n10 l200 vs. n02 l600	30849	1.812	0.0139	0.841	0.0012	2340.43	0.01
n10 l200 vs. n02 l800	28556	2.604	0.0236	0.808	0.0015	180.81	0.36
n10 l200 vs. n04 l050	4746	5.423	0.1608	0.579	0.0075	71.01	0.51
n10 l200 vs. n04 l100	15849	2.804	0.0320	0.731	0.0024	449.43	0.16
n10 l200 vs. n04 l200	27894	2.444	0.0209	0.769	0.0017	159.26	0.46
n10 l200 vs. n04 l400	31196	1.810	0.0137	0.816	0.0013	204.15	0.42
n10 l200 vs. n04 l600	32415	1.860	0.0141	0.822	0.0013	496.98	0.15
n10 l200 vs. n04 l800	30366	1.662	0.0126	0.835	0.0012	214.89	0.40
n10 l200 vs. n06 l050	5364	5.277	0.1370	0.575	0.0066	101.47	0.36
n10 l200 vs. n06 l100	16016	2.057	0.0216	0.760	0.0022	180.76	0.33
n10 l200 vs. n06 l200	26155	2.230	0.0189	0.762	0.0017	122.01	0.57
n10 l200 vs. n06 l400	29197	1.913	0.0149	0.794	0.0014	107.47	0.79
n10 l200 vs. n06 l600	29496	1.743	0.0131	0.807	0.0014	323.58	0.16
n10 l200 vs. n06 l800	30442	1.616	0.0119	0.821	0.0013	104.84	0.84
n10 l200 vs. n08 l050	5327	4.816	0.1142	0.590	0.0060	166.77	0.18
n10 l200 vs. n08 l100	14796	2.221	0.0246	0.746	0.0023	67.80	0.92
n10 l200 vs. n08 l200	25583	1.992	0.0166	0.771	0.0017	382.38	0.19
n10 l200 vs. n08 l400	30425	1.617	0.0121	0.809	0.0013	116.53	0.77
n10 l200 vs. n08 l600	31874	1.762	0.0131	0.807	0.0013	220.13	0.26
n10 l200 vs. n08 l800	31972	1.610	0.0115	0.815	0.0013	155.08	0.47
n10 l400 vs. n02 l050	4205	4.483	0.1184	0.635	0.0064	54.00	0.50
n10 l400 vs. n02 l100	17668	2.697	0.0299	0.764	0.0022	728.26	0.05
n10 l400 vs. n02 l200	38678	1.814	0.0123	0.827	0.0011	1844.28	0.01
n10 l400 vs. n02 l400	50195	1.421	0.0081	0.863	0.0008	112.55	0.90

continues . . .

(continued)

Distribution	N	Shape		Probability		AD_{up}	p-value
		Estimate	SE	Estimate	SE		
n10 l400 vs. n02 l600	49469	1.825	0.0112	0.851	0.0009	155.30	0.67
n10 l400 vs. n02 l800	46779	1.550	0.0094	0.869	0.0008	244.79	0.35
n10 l400 vs. n04 l050	4654	4.402	0.1096	0.631	0.0061	52.52	0.62
n10 l400 vs. n04 l100	15894	2.673	0.0309	0.754	0.0023	187.81	0.32
n10 l400 vs. n04 l200	33620	2.321	0.0177	0.785	0.0014	235.18	0.29
n10 l400 vs. n04 l400	45459	1.707	0.0106	0.831	0.0010	269.82	0.36
n10 l400 vs. n04 l600	47515	1.614	0.0098	0.847	0.0009	121.02	0.89
n10 l400 vs. n04 l800	47105	1.307	0.0076	0.866	0.0008	620.74	0.10
n10 l400 vs. n06 l050	5543	5.143	0.1265	0.590	0.0062	87.39	0.47
n10 l400 vs. n06 l100	17180	2.366	0.0247	0.748	0.0022	88.31	0.73
n10 l400 vs. n06 l200	33110	1.834	0.0132	0.797	0.0013	96.88	0.91
n10 l400 vs. n06 l400	45224	1.758	0.0108	0.811	0.0011	207.77	0.38
n10 l400 vs. n06 l600	45884	1.790	0.0110	0.813	0.0011	136.12	0.78
n10 l400 vs. n06 l800	46220	1.711	0.0104	0.822	0.0010	185.44	0.47
n10 l400 vs. n08 l050	5724	4.997	0.1023	0.588	0.0052	130.20	0.35
n10 l400 vs. n08 l100	16047	1.992	0.0208	0.776	0.0021	282.46	0.15
n10 l400 vs. n08 l200	31822	2.182	0.0167	0.771	0.0015	140.94	0.53
n10 l400 vs. n08 l400	43134	1.299	0.0078	0.840	0.0010	137.29	0.77
n10 l400 vs. n08 l600	46315	1.163	0.0067	0.853	0.0009	165.38	0.49
n10 l400 vs. n08 l800	47180	1.534	0.0091	0.834	0.0010	122.28	0.90
n10 l600 vs. n02 l050	3849	5.602	0.2083	0.595	0.0092	35.73	0.95
n10 l600 vs. n02 l100	16087	2.689	0.0320	0.773	0.0023	105.39	0.57
n10 l600 vs. n02 l200	36424	2.142	0.0159	0.820	0.0012	199.51	0.35
n10 l600 vs. n02 l400	54103	1.671	0.0095	0.857	0.0008	420.13	0.19
n10 l600 vs. n02 l600	57014	2.170	0.0131	0.840	0.0009	131.98	0.85
n10 l600 vs. n02 l800	56394	1.924	0.0112	0.857	0.0008	317.12	0.32
n10 l600 vs. n04 l050	4782	4.872	0.1361	0.613	0.0069	44.09	0.87
n10 l600 vs. n04 l100	15967	2.077	0.0219	0.790	0.0020	91.94	0.63
n10 l600 vs. n04 l200	32956	1.783	0.0129	0.823	0.0012	130.76	0.66
n10 l600 vs. n04 l400	49514	1.605	0.0095	0.844	0.0009	133.51	0.82
n10 l600 vs. n04 l600	54162	1.424	0.0079	0.862	0.0008	379.94	0.29
n10 l600 vs. n04 l800	54630	0.840	0.0043	0.897	0.0006	168.80	0.64
n10 l600 vs. n06 l050	6132	4.876	0.1050	0.604	0.0054	88.93	0.46
n10 l600 vs. n06 l100	18008	1.899	0.0184	0.786	0.0019	75.13	0.92
n10 l600 vs. n06 l200	33269	1.748	0.0124	0.807	0.0013	138.59	0.60
n10 l600 vs. n06 l400	50786	1.744	0.0101	0.815	0.0010	162.90	0.65
n10 l600 vs. n06 l600	54060	1.457	0.0080	0.840	0.0009	245.10	0.50
n10 l600 vs. n06 l800	54604	1.239	0.0066	0.855	0.0008	157.65	0.72
n10 l600 vs. n08 l050	6330	5.127	0.1100	0.594	0.0054	51.50	0.77
n10 l600 vs. n08 l100	16608	1.652	0.0161	0.798	0.0018	92.30	0.66
n10 l600 vs. n08 l200	32094	1.714	0.0124	0.806	0.0013	126.71	0.65
n10 l600 vs. n08 l400	47621	1.295	0.0074	0.843	0.0009	22205.90	0.00

continues . . .

(continued)

Distribution	N	Shape		Probability		AD_{up}	p-value
		Estimate	SE	Estimate	SE		
n10 l600 vs. n08 l600	52645	1.138	0.0061	0.860	0.0008	949.28	0.06
n10 l600 vs. n08 l800	54242	1.196	0.0064	0.861	0.0008	168.79	0.68
n10 l800 vs. n02 l050	3476	5.818	0.2471	0.590	0.0105	34.93	0.92
n10 l800 vs. n02 l100	14665	2.747	0.0341	0.774	0.0024	96.56	0.55
n10 l800 vs. n02 l200	33854	1.982	0.0150	0.834	0.0012	117.63	0.70
n10 l800 vs. n02 l400	53042	1.644	0.0094	0.862	0.0008	120.18	0.94
n10 l800 vs. n02 l600	58667	1.719	0.0096	0.864	0.0008	135.21	0.89
n10 l800 vs. n02 l800	59498	1.589	0.0086	0.875	0.0007	168.62	0.52
n10 l800 vs. n04 l050	4795	4.342	0.1062	0.639	0.0059	48.67	0.71
n10 l800 vs. n04 l100	15648	2.079	0.0224	0.795	0.0020	87.67	0.70
n10 l800 vs. n04 l200	31362	1.568	0.0113	0.836	0.0012	461.58	0.14
n10 l800 vs. n04 l400	49230	1.273	0.0072	0.865	0.0008	310.22	0.25
n10 l800 vs. n04 l600	56085	1.345	0.0073	0.870	0.0007	122.79	0.92
n10 l800 vs. n04 l800	57567	1.430	0.0077	0.867	0.0007	240.64	0.36
n10 l800 vs. n06 l050	6504	6.163	0.1551	0.560	0.0064	60.46	0.77
n10 l800 vs. n06 l100	17907	2.333	0.0237	0.761	0.0021	92.04	0.74
n10 l800 vs. n06 l200	32361	1.746	0.0126	0.812	0.0013	98.76	0.94
n10 l800 vs. n06 l400	50870	1.161	0.0063	0.853	0.0008	174.52	0.58
n10 l800 vs. n06 l600	56609	1.331	0.0070	0.848	0.0008	220.66	0.42
n10 l800 vs. n06 l800	57899	0.926	0.0046	0.878	0.0007	203.70	0.37
n10 l800 vs. n08 l050	6496	5.638	0.1221	0.574	0.0055	134.38	0.31
n10 l800 vs. n08 l100	16619	2.390	0.0253	0.761	0.0021	149.10	0.39
n10 l800 vs. n08 l200	31969	1.205	0.0082	0.842	0.0011	147.41	0.53
n10 l800 vs. n08 l400	47984	1.251	0.0071	0.849	0.0009	444.08	0.24
n10 l800 vs. n08 l600	54586	0.889	0.0045	0.879	0.0007	426.16	0.19
n10 l800 vs. n08 l800	56718	1.150	0.0059	0.865	0.0008	173.76	0.67
n12 l050 vs. n02 l050	2313	5.425	0.2249	0.558	0.0105	31.61	0.80
n12 l050 vs. n02 l100	3400	5.575	0.1968	0.576	0.0089	43.90	0.64
n12 l050 vs. n02 l200	3208	4.801	0.1666	0.627	0.0084	40.14	0.65
n12 l050 vs. n02 l400	2669	5.425	0.2184	0.606	0.0099	32.24	0.87
n12 l050 vs. n02 l600	2259	6.644	0.3690	0.562	0.0140	114.09	0.24
n12 l050 vs. n02 l800	2223	5.280	0.2660	0.626	0.0122	34.91	0.77
n12 l050 vs. n04 l050	1951	7.650	0.4414	0.484	0.0146	86.57	0.30
n12 l050 vs. n04 l100	2903	5.287	0.2045	0.593	0.0096	29.10	0.92
n12 l050 vs. n04 l200	3035	5.012	0.1826	0.609	0.0090	37.89	0.83
n12 l050 vs. n04 l400	3330	4.165	0.1218	0.649	0.0070	47.06	0.49
n12 l050 vs. n04 l600	3522	5.109	0.1701	0.603	0.0083	62.67	0.42
n12 l050 vs. n04 l800	3362	4.642	0.1443	0.627	0.0076	44.72	0.68
n12 l050 vs. n06 l050	1601	7.309	0.6607	0.465	0.0229	27.75	0.91
n12 l050 vs. n06 l100	2388	5.063	0.1811	0.561	0.0091	27.60	0.96
n12 l050 vs. n06 l200	2407	5.176	0.1734	0.565	0.0086	243.68	0.06
n12 l050 vs. n06 l400	3012	7.366	0.2318	0.485	0.0081	167.54	0.23

continues ...

(continued)

Distribution	N	Shape		Probability		AD_{up}	p-value
		Estimate	SE	Estimate	SE		
n12 l050 vs. n06 l600	3223	8.745	0.3036	0.449	0.0088	110.72	0.30
n12 l050 vs. n06 l800	3446	5.494	0.1321	0.558	0.0062	75.27	0.50
n12 l050 vs. n08 l050	1653	7.981	0.4881	0.438	0.0153	173.14	0.11
n12 l050 vs. n08 l100	2393	4.141	0.1319	0.596	0.0080	73.89	0.30
n12 l050 vs. n08 l200	3007	5.941	0.1862	0.525	0.0081	166.37	0.16
n12 l050 vs. n08 l400	3865	4.797	0.1143	0.576	0.0061	70.11	0.48
n12 l050 vs. n08 l600	4166	7.767	0.2315	0.478	0.0076	147.37	0.26
n12 l050 vs. n08 l800	4277	6.213	0.1537	0.529	0.0064	251.86	0.13
n12 l050 vs. n10 l050	2183	7.859	0.3719	0.445	0.0119	312.83	0.05
n12 l050 vs. n10 l100	3432	5.741	0.2037	0.529	0.0091	63.34	0.58
n12 l050 vs. n10 l200	3960	3.176	0.0694	0.658	0.0053	44.04	0.71
n12 l050 vs. n10 l400	4227	6.005	0.1592	0.538	0.0068	111.52	0.33
n12 l050 vs. n10 l600	4745	6.690	0.1859	0.524	0.0071	362.26	0.09
n12 l050 vs. n10 l800	4932	7.704	0.2763	0.498	0.0092	166.51	0.26
n12 l100 vs. n02 l050	3583	6.200	0.2473	0.539	0.0101	56.38	0.64
n12 l100 vs. n02 l100	10639	3.067	0.0454	0.714	0.0032	46.04	0.97
n12 l100 vs. n02 l200	13580	3.106	0.0423	0.733	0.0029	78.16	0.62
n12 l100 vs. n02 l400	11993	2.838	0.0396	0.764	0.0027	49.17	0.98
n12 l100 vs. n02 l600	10635	2.606	0.0380	0.784	0.0027	64.02	0.80
n12 l100 vs. n02 l800	9561	2.743	0.0422	0.781	0.0029	76.47	0.59
n12 l100 vs. n04 l050	3260	5.339	0.1922	0.576	0.0091	73.38	0.42
n12 l100 vs. n04 l100	8745	2.966	0.0458	0.717	0.0034	80.28	0.49
n12 l100 vs. n04 l200	12075	2.006	0.0242	0.789	0.0023	65.19	0.90
n12 l100 vs. n04 l400	12999	2.079	0.0246	0.794	0.0022	83.42	0.61
n12 l100 vs. n04 l600	13195	1.972	0.0232	0.803	0.0021	56.67	0.97
n12 l100 vs. n04 l800	12015	2.482	0.0327	0.779	0.0025	143.96	0.37
n12 l100 vs. n06 l050	3075	4.837	0.1430	0.563	0.0076	100.35	0.36
n12 l100 vs. n06 l100	6825	4.280	0.0902	0.607	0.0053	120.32	0.32
n12 l100 vs. n06 l200	8753	2.856	0.0423	0.696	0.0034	49.95	0.95
n12 l100 vs. n06 l400	10144	3.082	0.0427	0.692	0.0032	238.23	0.25
n12 l100 vs. n06 l600	10574	3.394	0.0469	0.677	0.0032	212.53	0.19
n12 l100 vs. n06 l800	11420	2.480	0.0303	0.733	0.0026	122.15	0.44
n12 l100 vs. n08 l050	3132	7.256	0.3191	0.474	0.0112	103.77	0.31
n12 l100 vs. n08 l100	7390	4.311	0.0896	0.611	0.0052	81.67	0.50
n12 l100 vs. n08 l200	10349	3.529	0.0532	0.656	0.0036	235.96	0.19
n12 l100 vs. n08 l400	12835	2.364	0.0276	0.728	0.0026	497.83	0.11
n12 l100 vs. n08 l600	13431	2.243	0.0250	0.741	0.0024	389.86	0.12
n12 l100 vs. n08 l800	13629	2.670	0.0300	0.717	0.0025	71.46	0.85
n12 l100 vs. n10 l050	4211	4.980	0.1334	0.565	0.0068	198.16	0.18
n12 l100 vs. n10 l100	9502	2.912	0.0447	0.684	0.0036	61.99	0.80
n12 l100 vs. n10 l200	13127	1.643	0.0181	0.777	0.0022	224.61	0.21
n12 l100 vs. n10 l400	14152	1.858	0.0198	0.770	0.0022	161.72	0.36

continues ...

(continued)

Distribution	N	Shape		Probability		AD_{up}	p-value
		Estimate	SE	Estimate	SE		
n12 l100 vs. n10 l600	14959	2.154	0.0228	0.758	0.0022	78.13	0.74
n12 l100 vs. n10 l800	15189	1.734	0.0175	0.788	0.0020	96.48	0.57
n12 l200 vs. n02 l050	3972	6.327	0.2558	0.542	0.0102	71.03	0.48
n12 l200 vs. n02 l100	16115	2.800	0.0341	0.738	0.0025	132.04	0.47
n12 l200 vs. n02 l200	29369	2.282	0.0191	0.782	0.0016	297.80	0.26
n12 l200 vs. n02 l400	30216	1.904	0.0149	0.821	0.0013	120.21	0.76
n12 l200 vs. n02 l600	27408	1.694	0.0137	0.840	0.0013	257.07	0.26
n12 l200 vs. n02 l800	25382	1.809	0.0154	0.841	0.0013	88.61	0.84
n12 l200 vs. n04 l050	4260	4.646	0.1364	0.612	0.0073	63.28	0.53
n12 l200 vs. n04 l100	14212	2.361	0.0282	0.764	0.0024	80.95	0.77
n12 l200 vs. n04 l200	25791	1.951	0.0161	0.796	0.0015	1205.51	0.03
n12 l200 vs. n04 l400	29344	1.872	0.0149	0.817	0.0014	725.11	0.07
n12 l200 vs. n04 l600	30078	1.302	0.0096	0.857	0.0011	89.21	0.93
n12 l200 vs. n04 l800	28226	1.543	0.0120	0.844	0.0012	242.41	0.29
n12 l200 vs. n06 l050	4308	5.597	0.1522	0.534	0.0070	126.71	0.30
n12 l200 vs. n06 l100	12812	2.074	0.0242	0.734	0.0026	69.14	0.92
n12 l200 vs. n06 l200	21464	2.161	0.0197	0.738	0.0020	103.35	0.74
n12 l200 vs. n06 l400	25108	1.523	0.0121	0.795	0.0015	196.03	0.36
n12 l200 vs. n06 l600	25592	2.263	0.0192	0.751	0.0018	126.81	0.52
n12 l200 vs. n06 l800	27435	1.428	0.0107	0.809	0.0014	125.83	0.55
n12 l200 vs. n08 l050	4409	4.340	0.1019	0.592	0.0059	48.46	0.79
n12 l200 vs. n08 l100	12555	2.660	0.0339	0.699	0.0029	80.27	0.74
n12 l200 vs. n08 l200	21916	2.235	0.0203	0.731	0.0020	1486.25	0.01
n12 l200 vs. n08 l400	27462	1.999	0.0162	0.761	0.0017	692.57	0.10
n12 l200 vs. n08 l600	29301	2.176	0.0174	0.756	0.0016	136.28	0.57
n12 l200 vs. n08 l800	29721	1.661	0.0123	0.795	0.0014	204.93	0.41
n12 l200 vs. n10 l050	6098	5.349	0.1204	0.551	0.0058	133.93	0.29
n12 l200 vs. n10 l100	14840	2.660	0.0327	0.708	0.0028	130.24	0.46
n12 l200 vs. n10 l200	24453	1.633	0.0136	0.783	0.0016	718.29	0.02
n12 l200 vs. n10 l400	29774	1.729	0.0130	0.787	0.0015	362.36	0.22
n12 l200 vs. n10 l600	31482	1.514	0.0108	0.810	0.0013	124.70	0.75
n12 l200 vs. n10 l800	31332	0.896	0.0060	0.857	0.0011	183.81	0.39
n12 l400 vs. n02 l050	3226	3.951	0.1230	0.642	0.0075	31.51	0.90
n12 l400 vs. n02 l100	13156	2.142	0.0261	0.784	0.0023	60.50	0.92
n12 l400 vs. n02 l200	29151	1.768	0.0139	0.825	0.0013	293.01	0.25
n12 l400 vs. n02 l400	41046	1.606	0.0105	0.850	0.0010	142.31	0.69
n12 l400 vs. n02 l600	40272	1.375	0.0088	0.868	0.0009	868.98	0.04
n12 l400 vs. n02 l800	37876	1.570	0.0107	0.865	0.0009	127.19	0.72
n12 l400 vs. n04 l050	3898	3.844	0.0963	0.642	0.0061	49.25	0.60
n12 l400 vs. n04 l100	12542	1.903	0.0228	0.794	0.0022	71.21	0.77
n12 l400 vs. n04 l200	25757	1.804	0.0151	0.818	0.0014	97.97	0.87
n12 l400 vs. n04 l400	36844	1.719	0.0120	0.832	0.0011	116.47	0.77

continues . . .

(continued)

Distribution	N	Shape Estimate	Shape SE	Probability Estimate	Probability SE	AD_{up}	p-value
n12 l400 vs. n04 l600	39447	1.015	0.0063	0.882	0.0008	4362.66	0.01
n12 l400 vs. n04 l800	39095	1.197	0.0076	0.873	0.0009	106.62	0.84
n12 l400 vs. n06 l050	4343	4.793	0.1136	0.565	0.0061	39.88	0.95
n12 l400 vs. n06 l100	13367	3.132	0.0394	0.669	0.0030	78.08	0.79
n12 l400 vs. n06 l200	23592	1.607	0.0131	0.776	0.0016	129.98	0.44
n12 l400 vs. n06 l400	34581	1.858	0.0129	0.771	0.0014	1146.09	0.01
n12 l400 vs. n06 l600	35592	1.551	0.0102	0.795	0.0013	141.35	0.60
n12 l400 vs. n06 l800	37234	1.762	0.0117	0.788	0.0013	121.09	0.76
n12 l400 vs. n08 l050	4735	4.509	0.0925	0.577	0.0053	122.05	0.30
n12 l400 vs. n08 l100	12358	2.981	0.0377	0.683	0.0030	72.69	0.77
n12 l400 vs. n08 l200	23643	1.714	0.0142	0.771	0.0017	110.04	0.71
n12 l400 vs. n08 l400	33622	1.427	0.0097	0.803	0.0013	177.87	0.40
n12 l400 vs. n08 l600	37572	1.668	0.0110	0.794	0.0013	123.47	0.78
n12 l400 vs. n08 l800	39093	1.463	0.0092	0.811	0.0011	143.07	0.63
n12 l400 vs. n10 l050	6544	5.479	0.1124	0.555	0.0053	209.60	0.20
n12 l400 vs. n10 l100	14756	1.981	0.0214	0.752	0.0023	531.43	0.09
n12 l400 vs. n10 l200	25259	1.445	0.0115	0.803	0.0015	114.09	0.50
n12 l400 vs. n10 l400	36296	1.428	0.0094	0.816	0.0012	197.30	0.43
n12 l400 vs. n10 l600	40982	1.317	0.0081	0.830	0.0010	148.05	0.55
n12 l400 vs. n10 l800	41605	1.332	0.0081	0.829	0.0010	193.52	0.38
n14 l050 vs. n02 l050	1163	3.339	0.1572	0.662	0.0112	18.83	0.92
n14 l050 vs. n02 l100	1683	4.160	0.1822	0.640	0.0106	26.30	0.86
n14 l050 vs. n02 l200	1427	4.036	0.2068	0.655	0.0122	26.49	0.74
n14 l050 vs. n02 l400	1154	4.105	0.2368	0.661	0.0135	20.86	0.80
n14 l050 vs. n02 l600	1097	4.354	0.2431	0.633	0.0136	27.47	0.64
n14 l050 vs. n02 l800	934	3.033	0.1489	0.706	0.0110	47.84	0.35
n14 l050 vs. n04 l050	934	5.938	0.4128	0.522	0.0178	33.51	0.49
n14 l050 vs. n04 l100	1374	4.023	0.2014	0.635	0.0122	24.09	0.88
n14 l050 vs. n04 l200	1487	5.715	0.4746	0.561	0.0210	62.83	0.38
n14 l050 vs. n04 l400	1734	3.665	0.1468	0.651	0.0096	48.66	0.42
n14 l050 vs. n04 l600	1807	3.108	0.1101	0.673	0.0084	30.11	0.79
n14 l050 vs. n04 l800	1555	3.792	0.1476	0.644	0.0095	74.22	0.21
n14 l050 vs. n06 l050	887	5.994	0.3691	0.465	0.0157	122.23	0.17
n14 l050 vs. n06 l100	1408	6.043	0.3447	0.485	0.0146	29.94	0.79
n14 l050 vs. n06 l200	1383	3.274	0.1226	0.625	0.0094	40.77	0.44
n14 l050 vs. n06 l400	1653	8.573	0.5057	0.426	0.0147	172.35	0.12
n14 l050 vs. n06 l600	1659	10.342	1.0643	0.396	0.0250	66.29	0.37
n14 l050 vs. n06 l800	1872	10.456	1.7322	0.390	0.0399	45.72	0.61
n14 l050 vs. n08 l050	934	5.503	0.3637	0.510	0.0170	28.63	0.70
n14 l050 vs. n08 l100	1479	5.867	0.3477	0.495	0.0152	26.16	0.89
n14 l050 vs. n08 l200	1728	6.176	0.2398	0.479	0.0100	71.51	0.42
n14 l050 vs. n08 l400	2312	5.105	0.1679	0.527	0.0085	156.70	0.12

continues ...

(continued)

Distribution	N	Shape Estimate	Shape SE	Probability Estimate	Probability SE	AD_{up}	p-value
n14 l050 vs. n08 l600	2348	9.560	0.7057	0.401	0.0180	177.34	0.17
n14 l050 vs. n08 l800	2363	5.741	0.1882	0.510	0.0085	167.40	0.20
n14 l050 vs. n10 l050	1262	5.672	0.3292	0.499	0.0149	68.02	0.25
n14 l050 vs. n10 l100	2021	5.102	0.2041	0.526	0.0103	51.40	0.52
n14 l050 vs. n10 l200	2378	4.651	0.1699	0.556	0.0094	63.74	0.39
n14 l050 vs. n10 l400	2456	3.530	0.0989	0.626	0.0070	82.30	0.29
n14 l050 vs. n10 l600	2699	2.754	0.0682	0.667	0.0060	107.69	0.19
n14 l050 vs. n10 l800	2658	6.377	0.2104	0.503	0.0085	94.34	0.34
n14 l050 vs. n12 l050	799	4.468	0.3148	0.573	0.0179	15.38	0.98
n14 l050 vs. n12 l100	1596	3.025	0.1082	0.655	0.0087	37.91	0.58
n14 l050 vs. n12 l200	2483	4.303	0.1452	0.585	0.0086	97.91	0.22
n14 l050 vs. n12 l400	2440	3.800	0.1163	0.609	0.0077	38.50	0.65
n14 l100 vs. n02 l050	1958	6.143	0.3389	0.539	0.0140	148.49	0.08
n14 l100 vs. n02 l100	6141	2.414	0.0444	0.758	0.0037	56.72	0.64
n14 l100 vs. n02 l200	7585	2.746	0.0484	0.754	0.0035	233.63	0.15
n14 l100 vs. n02 l400	6291	2.406	0.0446	0.791	0.0034	53.32	0.73
n14 l100 vs. n02 l600	5454	2.303	0.0458	0.800	0.0035	47.42	0.76
n14 l100 vs. n02 l800	4816	2.368	0.0499	0.797	0.0038	56.22	0.52
n14 l100 vs. n04 l050	1916	4.087	0.1412	0.612	0.0086	37.15	0.59
n14 l100 vs. n04 l100	4924	1.975	0.0377	0.775	0.0038	34.21	0.96
n14 l100 vs. n04 l200	6411	2.666	0.0499	0.743	0.0039	58.98	0.61
n14 l100 vs. n04 l400	6909	1.703	0.0269	0.805	0.0029	58.68	0.59
n14 l100 vs. n04 l600	7457	1.951	0.0304	0.786	0.0030	67.52	0.61
n14 l100 vs. n04 l800	6628	2.412	0.0421	0.765	0.0035	115.67	0.40
n14 l100 vs. n06 l050	1787	5.936	0.3093	0.508	0.0134	51.92	0.53
n14 l100 vs. n06 l100	4136	4.039	0.0995	0.597	0.0062	73.36	0.51
n14 l100 vs. n06 l200	5003	2.111	0.0375	0.719	0.0040	54.43	0.55
n14 l100 vs. n06 l400	5879	2.767	0.0465	0.681	0.0040	393.22	0.08
n14 l100 vs. n06 l600	6311	4.682	0.0951	0.596	0.0051	75.12	0.55
n14 l100 vs. n06 l800	6871	2.755	0.0440	0.693	0.0037	649.22	0.01
n14 l100 vs. n08 l050	1827	3.774	0.1285	0.597	0.0087	38.64	0.46
n14 l100 vs. n08 l100	4222	3.507	0.0837	0.628	0.0059	42.93	0.85
n14 l100 vs. n08 l200	5862	2.692	0.0478	0.682	0.0042	292.54	0.09
n14 l100 vs. n08 l400	7597	3.168	0.0509	0.655	0.0039	207.11	0.24
n14 l100 vs. n08 l600	8160	2.266	0.0318	0.717	0.0032	239.74	0.19
n14 l100 vs. n08 l800	8250	2.531	0.0359	0.705	0.0033	62.44	0.82
n14 l100 vs. n10 l050	2594	5.031	0.1481	0.538	0.0076	140.50	0.13
n14 l100 vs. n10 l100	5490	2.878	0.0558	0.666	0.0047	124.62	0.27
n14 l100 vs. n10 l200	7548	3.457	0.0623	0.645	0.0044	123.52	0.30
n14 l100 vs. n10 l400	8504	1.563	0.0207	0.773	0.0027	709.59	0.01
n14 l100 vs. n10 l600	9093	1.927	0.0253	0.749	0.0028	72.50	0.59
n14 l100 vs. n10 l800	9347	1.924	0.0248	0.754	0.0027	80.79	0.62

continues ...

(continued)

Distribution	N	Shape		Probability		AD_{up}	p-value
		Estimate	SE	Estimate	SE		
n14 l100 vs. n12 l050	1803	5.483	0.2498	0.537	0.0117	70.73	0.35
n14 l100 vs. n12 l100	5041	3.236	0.0723	0.671	0.0053	134.63	0.32
n14 l100 vs. n12 l200	7867	2.774	0.0460	0.697	0.0038	51.02	0.87
n14 l100 vs. n12 l400	7781	2.081	0.0314	0.749	0.0032	69.44	0.56
n14 l200 vs. n02 l050	2698	3.992	0.1331	0.633	0.0081	50.36	0.41
n14 l200 vs. n02 l100	10924	2.179	0.0299	0.783	0.0026	64.63	0.80
n14 l200 vs. n02 l200	19704	1.656	0.0159	0.832	0.0015	93.53	0.78
n14 l200 vs. n02 l400	18627	1.761	0.0178	0.843	0.0015	87.68	0.81
n14 l200 vs. n02 l600	16213	1.643	0.0177	0.858	0.0015	102.69	0.49
n14 l200 vs. n02 l800	14739	1.662	0.0187	0.860	0.0016	88.35	0.59
n14 l200 vs. n04 l050	2864	3.623	0.1057	0.640	0.0071	38.53	0.69
n14 l200 vs. n04 l100	9212	1.852	0.0256	0.782	0.0027	64.42	0.68
n14 l200 vs. n04 l200	15853	1.863	0.0198	0.793	0.0020	166.45	0.34
n14 l200 vs. n04 l400	17262	1.390	0.0137	0.841	0.0016	74.90	0.89
n14 l200 vs. n04 l600	18409	1.346	0.0127	0.845	0.0015	274.65	0.24
n14 l200 vs. n04 l800	17237	1.229	0.0118	0.856	0.0015	94.59	0.52
n14 l200 vs. n06 l050	2909	5.555	0.1836	0.516	0.0086	76.61	0.46
n14 l200 vs. n06 l100	8186	2.956	0.0460	0.658	0.0038	118.76	0.37
n14 l200 vs. n06 l200	12768	2.546	0.0309	0.698	0.0028	106.82	0.63
n14 l200 vs. n06 l400	15648	1.427	0.0138	0.783	0.0020	197.30	0.32
n14 l200 vs. n06 l600	16040	1.600	0.0154	0.775	0.0020	165.41	0.26
n14 l200 vs. n06 l800	17277	1.940	0.0186	0.754	0.0020	93.28	0.70
n14 l200 vs. n08 l050	2943	7.405	0.2550	0.451	0.0087	227.25	0.12
n14 l200 vs. n08 l100	7758	2.479	0.0371	0.682	0.0036	356.65	0.08
n14 l200 vs. n08 l200	13255	2.060	0.0229	0.720	0.0025	327.77	0.14
n14 l200 vs. n08 l400	17349	1.544	0.0144	0.769	0.0019	200.60	0.26
n14 l200 vs. n08 l600	18613	1.311	0.0116	0.795	0.0017	409.01	0.13
n14 l200 vs. n08 l800	19230	1.844	0.0166	0.757	0.0019	145.53	0.35
n14 l200 vs. n10 l050	4290	4.218	0.0872	0.572	0.0053	151.58	0.27
n14 l200 vs. n10 l100	9957	1.069	0.0129	0.806	0.0024	173.18	0.25
n14 l200 vs. n10 l200	15737	2.088	0.0222	0.730	0.0023	104.58	0.50
n14 l200 vs. n10 l400	19441	1.488	0.0132	0.787	0.0018	912.52	0.04
n14 l200 vs. n10 l600	21261	1.500	0.0128	0.794	0.0017	125.93	0.44
n14 l200 vs. n10 l800	21669	1.312	0.0108	0.811	0.0015	132.84	0.47
n14 l200 vs. n12 l050	2887	3.592	0.0958	0.625	0.0066	119.01	0.24
n14 l200 vs. n12 l100	8870	2.243	0.0326	0.732	0.0032	59.92	0.82
n14 l200 vs. n12 l200	16263	1.082	0.0104	0.826	0.0017	326.99	0.15
n14 l200 vs. n12 l400	17233	1.579	0.0156	0.799	0.0019	149.56	0.31
n14 l400 vs. n02 l050	2644	3.739	0.1261	0.657	0.0080	33.28	0.79
n14 l400 vs. n02 l100	10880	2.093	0.0290	0.806	0.0024	75.14	0.62
n14 l400 vs. n02 l200	25779	1.714	0.0149	0.849	0.0013	130.20	0.42
n14 l400 vs. n02 l400	36065	1.481	0.0104	0.875	0.0009	85.22	0.96

continues . . .

(continued)

Distribution	N	Shape		Probability		AD_{up}	p-value
		Estimate	SE	Estimate	SE		
n14 l400 vs. n02 l600	34451	1.600	0.0119	0.881	0.0009	265.75	0.31
n14 l400 vs. n02 l800	31438	1.295	0.0095	0.899	0.0008	134.19	0.57
n14 l400 vs. n04 l050	3089	3.774	0.1059	0.639	0.0069	32.27	0.92
n14 l400 vs. n04 l100	9954	1.741	0.0231	0.800	0.0024	63.31	0.79
n14 l400 vs. n04 l200	21041	1.307	0.0113	0.842	0.0014	125.62	0.44
n14 l400 vs. n04 l400	30342	1.033	0.0073	0.876	0.0010	120.86	0.67
n14 l400 vs. n04 l600	32327	1.170	0.0083	0.874	0.0010	94.78	0.92
n14 l400 vs. n04 l800	31827	1.122	0.0079	0.879	0.0009	744.86	0.06
n14 l400 vs. n06 l050	3341	7.103	0.2575	0.474	0.0093	176.48	0.18
n14 l400 vs. n06 l100	9857	1.964	0.0250	0.731	0.0028	109.15	0.31
n14 l400 vs. n06 l200	18535	1.623	0.0149	0.769	0.0019	178.46	0.28
n14 l400 vs. n06 l400	27704	1.282	0.0093	0.803	0.0014	500.71	0.11
n14 l400 vs. n06 l600	28810	1.033	0.0073	0.836	0.0012	149.28	0.47
n14 l400 vs. n06 l800	30309	1.126	0.0078	0.827	0.0012	244.81	0.34
n14 l400 vs. n08 l050	3463	4.950	0.1241	0.550	0.0065	173.16	0.18
n14 l400 vs. n08 l100	9491	3.404	0.0507	0.642	0.0036	95.71	0.55
n14 l400 vs. n08 l200	18272	1.942	0.0187	0.743	0.0021	102.62	0.67
n14 l400 vs. n08 l400	27109	1.416	0.0107	0.795	0.0015	377.87	0.18
n14 l400 vs. n08 l600	30631	0.756	0.0050	0.856	0.0011	298.94	0.27
n14 l400 vs. n08 l800	32067	1.313	0.0091	0.814	0.0013	137.65	0.65
n14 l400 vs. n10 l050	5270	3.432	0.0609	0.625	0.0045	166.29	0.25
n14 l400 vs. n10 l100	11821	1.786	0.0205	0.748	0.0025	349.97	0.09
n14 l400 vs. n10 l200	20648	1.548	0.0137	0.787	0.0017	99.71	0.75
n14 l400 vs. n10 l400	29698	1.070	0.0075	0.835	0.0012	142.47	0.52
n14 l400 vs. n10 l600	34497	1.165	0.0077	0.831	0.0011	797.49	0.04
n14 l400 vs. n10 l800	35763	1.166	0.0075	0.835	0.0011	153.85	0.45
n14 l400 vs. n12 l050	3274	4.900	0.1367	0.570	0.0071	91.72	0.32
n14 l400 vs. n12 l100	10020	1.568	0.0197	0.790	0.0024	746.46	0.08
n14 l400 vs. n12 l200	21426	1.575	0.0141	0.803	0.0017	263.01	0.26
n14 l400 vs. n12 l400	26534	0.689	0.0050	0.884	0.0010	140.24	0.53

Distribution represents distributions obtained from aligning simulated profiles of ENO n and length l against simulated profiles with different values of n and l. Profiles were simulated using $S = 1012$ source profiles with a noise level $r = 0.03$ and a fragment length $s = 9$. The table reports the estimates and the Cramér–Rao lower bound on their standard errors (SE) for the shape (the number of non-positive scores) and probability (of having a positive score) parameters of the NBD for each distribution of the number of positive scores. N is the number of alignments between simulated profiles. AD_{up} is the upper-tail Anderson-Darling statistic. The p-value of statistic AD_{up} was computed by Monte Carlo simulation with 100 samples.
